# Research Poster Abstracts

**DOI:** 10.1080/24740527.2018.1476313

**Published:** 2018-05-21

## Characteristics and social demographics of chronic pain patients referred to a multidisciplinary chronic pain clinic in BC

Curtis May^a^, Vanessa Brcic^b^ and Brenda Lau^c^

^a^Faculty of Medicine, Bkin, University of British Columbia, Vancouver, BC, Canada, c_may@shaw.ca; ^b^Faculty of Medicine, University of British Columbia, Vancouver, BC, Canada; ^c^Dept. of Anesthesiology, Pharmacology, & Therapeutics, University of British Columbia Faculty of Medicine, Vancouver, BC, Canada

**CONTACT** Curtis May c_may@shaw.ca

© 2018 Curtis May, Vanessa Brcic, and Brenda Lau. Published with license by Taylor & Francis Group, LLC.

This is an Open Access article distributed under the terms of the Creative Commons Attribution License (http://creativecommons.org/licenses/by/4.0/), which permits unrestricted use, distribution, and reproduction in any medium, provided the original work is properly cited.

**Introduction/Aim**: Community-based pain services help fill a significant service gap for patients suffering from chronic non-cancer pain. Very little is known about the patients accessing community pain services yet understanding this population is essential to develop patient-centered services. Thus, our primary objective is to describe the characteristics and socioeconomic status of patients presenting to a community-based multidisciplinary, chronic pain clinic in Vancouver, BC.

**Methods**: Retrospective cross-sectional study of self-reported data from 935 consecutive patients.

**Results**: The mean age of the population (n = 935) was 49.5 (SD, 14.9) years; 70% were female. Of 935 patients, 79.8% reported income, and of those, 45.2% made less than $20000 individual income annually. Before consultation, 73.4% of patients were taking medication for their pain, 45.6% of whom took opioids and 44.9% antidepressants; 65% previously used either an emergency department or walk-in clinic for pain management; 50.7% of patients have been in pain for 5 years or more. 63% of patients reported severe disability (>6/10) as per the Brief Pain Inventory – 30% of patients were unable to work due to disability.

**Discussion/Conclusions**: Low-income was prevalent in this community patient population limiting access to multi-dimensional pain care, the gold standard for complex chronic pain. This may lead to an over-reliance on medications such as opioids rather than biopsychsocial care aimed at restoring functioning. Funding models that support community-based care as well as early intervention and income supports for patients should be considered to reduce disability. Further study is needed to inform patient-centered service delivery.

## Family members’ perceptions of the Critical-Care Pain Observation Tool use in their loved one in the intensive care unit

Sarah Mohand-Saïd^a^, Melissa Richard-Lalonde^b^, Madalina Boitor^b^ and Céline Gélinas^b^

^a^Faculty of Medicine, McGill University, Montréal, Québec, Canada; ^b^Ingram School of Nursing, and Centre for Nursing Research, CIUSSS Centre-Ouest-Nord-Ile-Montreal – Jewish General Hospital, McGill University, Montréal, Québec, Canada

**CONTACT** Sarah Mohand-Saïd sarah.mohand-said@mail.mcgill.ca Faculty of Medicine, McGill University, Montréal, Québec, Canada

© 2018 Sarah Mohand-Saïd, Melissa Richard-Lalonde, Madalina Boitor and Céline Gélinas. Published with license by Taylor & Francis Group, LLC.

This is an Open Access article distributed under the terms of the Creative Commons Attribution License (http://creativecommons.org/licenses/by/4.0/), which permits unrestricted use, distribution, and reproduction in any medium, provided the original work is properly cited.

**Introduction/Aim**: Family participation is encouraged in pain assessment of patients unable to self-report because of their familiarity with the patient. This study described the family members’ perceptions of the 0–8 Critical-Care Pain Observation Tool (CPOT) in assessing their loved one’s pain in the intensive care unit (ICU).

**Methods**: A descriptive design was used. To be eligible, family members had to be aged ≥18 years, French or English speaking, knowing the patient ≥ 1 year, and with a loved one unable to self-report admitted to the ICU ≥ 3 days whom they had visited ≥ 3 times. They received a 15-minute training of the CPOT use and assessed their loved one’s pain at their next visit. Then, they completed a survey (1–5 scale response) about the CPOT relevance and their role in pain management.

**Results**: Ten family members (mean age = 59.6 years; 60% parent; 40% spouse) participated. They visited their loved one 2.75 hours on average per day in the ICU. They rated their loved one’s pain at rest or during a procedure with CPOT scores from 2 to 6 (median = 3.5). Most CPOT items (e.g., facial expression, body movement) were rated as relevant (medians≥4.5) for pain assessment except ventilator compliance (median = 3). They felt comfortable using the CPOT (median = 5) and expressed their willingness to be more involved in their loved one’s pain management (median = 5).

**Discussion/Conclusions**: This is the first study to introduce the CPOT to ICU family members. Findings support that family members could play a role in their loved one’s pain management.

## Cannabis use among chronic non-cancer pain patients (CNCP) in a community based academic pain clinic: demographics and pain characterization

S. Fatima Lakha^a,c^, Amol Deshpande^b^ and Angela Mailis^c,d,e^

^a^Institute of Medical Science, University of Toronto, Toronto, Ontario, Canada; ^b^Dept. of Family and Community Medicine, University of Toronto, Toronto, Ontario, Canada; ^c^Pain and Wellness Centre, Vaughan, Ontario; ^d^Dept. of Medicine, Division of Physical Medicine, University of Toronto; ^e^Toronto Rehab Institute, University Health Network, Toronto, Ontario, Canada

**CONTACT** S. Fatima Lakha sfatima.lakha@utoronto.ca

© 2018 S. Fatima Lakha, Amol Deshpande and Angela Mailis. Published with license by Taylor & Francis Group, LLC.

This is an Open Access article distributed under the terms of the Creative Commons Attribution License (http://creativecommons.org/licenses/by/4.0/), which permits unrestricted use, distribution, and reproduction in any medium, provided the original work is properly cited.

**Introduction/Aim**: Cannabis use in chronic non-cancer pain (CNCP) is highly prevalent, however, actual patterns of use still remain poorly characterized. To describe the demographics and pain characteristics of cannabis use in CNCP patients referred to the Pain and Wellness Centre (PWC), a community-based university affiliated pain clinic in Vaughan, Ontario.

**Methods**: This retrospective study reviewed the characteristics of 39 cannabis users among 110 consecutive CNCP patients referred to PWC between 2016 –2017. Data collected included: 1) demographics (age, sex, marital status, work status, country of birth, ethnicity, education); 2) pain characteristics (NRS pain rating (0–10), duration of pain, inciting event, primary pain complaint and 3) cannabis source and delivery route.

**Results**: Male/female ratio was 1:1.3; mean age 35 ± 14 years; mean pain ratings were 6.8 ± 1.3, with average pain duration of 10 ± 12 years. The largest cohort by age was 31–40 years of age with an average duration of pain of 5.2 ± 4.3years. No patients were over 65. Canadian-born patients constituted 77% of the population and 51% were unmarried. The primary delivery route was inhaled (smoked) 44%. Only 43% had a medical cannabis document and with 15% using cannabis exclusively for pain relief. Motor vehicle accidents and sports injuries accounted for the onset of pain in 41% of patients. Primary areas of pain were reported as shoulder, neck and low back (41%, 39% and 38%, respectively).

**Discussion/Conclusions**: Cannabis use in many CNCP patients may be associated with the need to manage multiple symptoms rather than being solely used as an analgesic.

## Do you understand what I am experiencing?: the role of partner empathy on pain expression in couples during experimental pain

Michelle M. Gagnon^a^, Thomas Hadjistavropoulos^b^ and Ying C. MacNab^c^

^a^Department of Psychology, University of Saskatchewan, Saskatoon, Saskatchewan, Canada; ^b^Department of Psychology, University of Regina, Regina, Saskatchewan, Canada; ^c^School of Population and Public Health, University of British Columbia, Vancouver, Canada

**CONTACT** Michelle M. Gagnon michelle.gagnon@usask.ca

© 2018 Michelle M. Gagnon, Thomas Hadjistavropoulos and Ying C. MacNab. Published with license by Taylor & Francis Group, LLC.

This is an Open Access article distributed under the terms of the Creative Commons Attribution License (http://creativecommons.org/licenses/by/4.0/), which permits unrestricted use, distribution, and reproduction in any medium, provided the original work is properly cited.

**Introduction/Aim**: We examined the role of empathy in predicting pain responses in couples with and without a partner with chronic pain during an experimental pain task. Mixed evidence exists regarding the influence of empathy on pain in dyads, and the relationship between empathy and non-verbal pain expression has not been examined. Using data from a larger investigation, we examined whether partners of an individual with chronic pain (ICP) reported lower empathy than partners of non-ICPs, and whether empathy predicted pain-related facial expression and pain ratings of partners completing a pain induction task.

**Methods**: Couples with (*n* = 66) and without (*n* = 65) an ICP completed a measure of empathy and relationship questionnaires (not included in this study). One partner completed a pain task (pain target, PT), while the other partner observed (pain observer, PO). Pain intensity and perceived pain intensity ratings were requested during the task and facial expressions were video-recorded. In couples with an ICP, the ICP was the PT. Pain-related facial expression was quantified using the Facial Action Coding System (FACS).

**Results**: There were no significant differences between partners with and without an ICP in empathy ratings. PO empathy significantly predicted PT FACS scores, but was not associated with PT self-reported pain scores.

**Discussion/Conclusions**: Partner empathy appears to influence the non-verbal pain expression, but not the self-reported pain scores, of individuals completing an experimental paint ask. This is consistent with previous findings highlighting the disparate influence of interpersonal variables on facets of pain expression.

## Differences between cannabis users and abstainers among chronic non-cancer patients referred to a community based pain clinic in Ontario

S. Fatima Lakha^a^, Peter Pennefather^b^, Amol Deshpande^c^ and Angela Mailis^d,e,f^

^a^Institute of Medical Sciences, University of Toronto, Toronto, Ontario, Canada; ^b^Leslie Dan Faculty of Pharmacy, University of Toronto, Toronto, Ontario, Canada; ^c^Dept. of Family and Community Medicine, University of Toronto, Toronto, Ontario, Canada; ^d^Pain and Wellness Centre, Vaughan, Ontario; ^e^Dept. of Medicine, Division of Physical Medicine, UofToronto; ^f^Toronto Rehab Institute, University Health Network, Toronto, Ontario, Canada

**CONTACT** S. Fatima Lakha sfatima.lakha@utoronto.ca

© 2018 S. Fatima Lakha, Peter Pennefather, Amol Deshpande and Angela Mailis. Published with license by Taylor & Francis Group, LLC.

This is an Open Access article distributed under the terms of the Creative Commons Attribution License (http://creativecommons.org/licenses/by/4.0/), which permits unrestricted use, distribution, and reproduction in any medium, provided the original work is properly cited.

**Introduction/Aim**: To compare various attributes between chronic non-cancer pain (CNCP) patients who use cannabis versus those who do not at the Pain and Wellness Centre (PWC), a community-based academic pain clinic in Vaughan, Ontario.

**Methods**: This retrospective study was conducted on 110 consecutive CNCP patients assessed during 2016 –2017. The study population was classified into Cannabis Users (CU, n = 39) and Non-Cannabis Users (NCU, n = 71). Data collected included: a) demographics; b) pain characteristics; and c) emotional and functional status obtained by validated instruments.

**Results**: Male/female ratio for CU was 1:1.3 compared to 1:1.6 for NCU. Mean age was 35 ± 14 vs 51 ± 17 (p < 0.0001), respectively. Foreign-born CU constituted 23% vs 39% NCU and 51% vs 24% (p < 0.05) were unmarried. One third of both groups was unemployed due to pain, though 36% of CU and 18% of NCU were employed full-time. The commonest pain site was low back with car accidents cited as the most frequent cause in both groups. Average pain duration in months was 9.3 ± 12 (CU) vs 5.8 ± 6 (NCU) (p < 0.05); no differences were found in average NRS rating (6.8 ± 1.4). Only 43% of CU received a medical document for cannabis with a minority (15%) using cannabis exclusively for pain relief. No clinically meaningful differences were detected on emotional or functional status variables between CU and NCU.

**Discussion/Conclusions**: Non-cannabis users were more likely to be older, foreign born, married, with shorter pain duration than cannabis users. The relevance to our clinical experience in a community with an older ethnic population will be discussed.

## In-hospital opioid trajectories, but not pain intensity trajectories, are associated with pain disability 6 months following hepatic resection

M. Gabrielle Pagé^a^, Paul J Karanicolas^b^, Sean Cleary^b^, Alice C Wei^b^, Paul McHardy^c^, Salima S. J. Ladak^c^, Nour Ayach^d^, Stuart A. McCluskey^d^, Coimbatore Srinivas^d^, Joel Katz^e^, Natalie Coburn^b^, Julie Hallet^b^, Calvin H. L. Law^b^ and Hance Clarke^c^

^a^Centre de recherche, Centre hospitalier de l’Université de Montréal, Montréal, Quebec, Canada; ^b^Surgery, University of Toronto, Toronto, Canada; ^c^Anaesthesia, University of Toronto, Toronto, Canada; ^d^Toronto General Hospital, Pain Research Unit, Toronto, Canada; ^e^Psychology, York University, Toronto, Canada

**CONTACT** M. Gabrielle Pagé gabrielle.page@umontreal.ca

© 2018 M. Gabrielle Pagé, Paul J Karanicolas, Sean Cleary, Alice C Wei, Paul McHardy, Salima S. J. Ladak, Nour Ayach, Stuart A. McCluskey, Coimbatore Srinivas, Joel Katz, Natalie Coburn, Julie Hallet, Calvin H. L. Law, and Hance Clarke Published with license by Taylor & Francis Group, LLC.

This is an Open Access article distributed under the terms of the Creative Commons Attribution License (http://creativecommons.org/licenses/by/4.0/), which permits unrestricted use, distribution, and reproduction in any medium, provided the original work is properly cited.

**Introduction/Aim:** The study objectives were to (1) identify subgroup trajectories of (a) cumulative opioid consumption and (b) pain intensity over the first 72 hours after open hepatic resection and (2) examine the association between opioid and pain trajectories and 6-month pain-related disability.

**Methods:** A total of 153 participants undergoing open hepatectomy completed self-report measures pre-operatively, several times daily over the first 3 post-operative days and 6 months later. Growth mixture modeling was used to identify opioid and pain trajectories. General linear and logistic regression models were used to examine the association of trajectory memberships with 6-month pain disability and chronic post-surgical pain (CPSP), respectively.

**Results:** For both opioid and pain trajectory analyses a 5-trajectory model with a quadratic term best fit the data. Patient trajectories differed in terms of initial levels of opioid consumption and pain intensity and rates of change. No significant baseline predictors of trajectory memberships were found. Opioid and pain intensity trajectories did not significantly predict status of CPSP at 6 months. Patients in the two highest opioid consumption trajectories (Trajectory-4: n = 39; Trajectory-5: n = 16) had significantly higher pain disability scores at six months compared to patients in trajectories 1–3 (all *p *< 0.05).

**Discussion/Conclusions:** Results support the importance of examining homogeneous subgroups of pain and opioid consumption in the days following surgery. Understanding modifiable risk factors for belonging to opioid trajectory memberships might help identify patients at risk of high opioid consumption and poor outcomes and facilitate strategies to minimize opioid use and improve long-term outcomes.

## Investigating links between distress-promoting parent behaviours and infant pain-related distress during 12-month vaccinations

Shaylea Badovinac^a^, Hannah Gennis^a^, Rebecca Pillai Riddell^a^ and Hartley Garfield^b^

^a^Clinical-Developmental Psychology, York University, Toronto, Canada; ^b^Pediatrics, University of Toronto, Toronto, Canada

**CONTACT** Rebecca Pillai Riddell rpr@yorku.ca

© 2018 Shaylea Badovinac, Hannah Gennis, Rebecca Pillai Riddell, and Hartley Garfield. Published with license by Taylor & Francis Group, LLC.

This is an Open Access article distributed under the terms of the Creative Commons Attribution License (http://creativecommons.org/licenses/by/4.0/), which permits unrestricted use, distribution, and reproduction in any medium, provided the original work is properly cited.

**Introduction/Aim**: Caregivers play a critical role in managing infants’ pain-related distress. It has previously been shown that distress-promoting parent behaviours are associated with greater infant pain-related distress during 12-month vaccinations (Pillai Riddell et al., in press).

**Objective**: Our objective was to examine relationships between distress-promoting parent behaviours and physiological indicators associated with infant pain-related distress (i.e., infant heart rate).

**Methods**: The study included parent-infant dyads (*n* = 55) from the 12-month wave of an ongoing longitudinal study (the OUCH Cardio Cohort). Dyads were videotaped and connected to equipment that recorded their heart rate. Videotapes were coded for parent distress-promoting behaviours post-needle (Pillai Riddell, Gennis, et al., under review). Heart rate was averaged over 30-second epochs at 1, 2, and 3 minutes post-needle using the MindWare analysis system (HRV Analysis 3.1.3.).

**Results**: Bonferroni-corrected bivariate correlations revealed associations between total distress-promoting behaviours and infant heart rate at 2 (*r = *0.36, *p *= 0.006) and 3 minutes (*r *= 0.36, *p *= 0.007) post-needle.

**Discussion/Conclusions**: This study extends previous findings on the relationship between caregiver behaviours and infant pain-related distress. Our results indicate that distress-promoting parent behaviours during vaccination are associated with regulatory physiological reactions but do not seem to be related to initial or peak reactivity.

References1.Pillai Riddell
R, Gennis
H, Tablon
P, Greenberg
S, Garfield
H.
Developing a measure of distress-promoting parent behaviours during infant vaccination: Assessing reliability and validity. Can J Pain. in press.10.1080/24740527.2018.1471325PMC873061035005373

## Pain intensity and satisfaction with pain management of patients in a newly built single-patient room intensive care unit

Melissa Richard-Lalonde^a^, Darina M. Tsoller^b^ and Céline Gélinas^a,b^

^a^Ingram School of Nursing, Montreal, McGill University, Montréal, Canada; ^b^Centre for Nursing Research, CIUSSS Centre-Ouest-Ile-Montréal – Jewish General Hospital, Montréal, Canada

**CONTACT** Céline Gélinas celine.gelinas@mcgill.ca

© 2018 Melissa Richard-Lalonde, Darina M. Tsoller, and Céline Gélinas. Published with license by Taylor & Francis Group, LLC.

This is an Open Access article distributed under the terms of the Creative Commons Attribution License (http://creativecommons.org/licenses/by/4.0/), which permits unrestricted use, distribution, and reproduction in any medium, provided the original work is properly cited.

**Introduction/Aim**: In January 2016, the adult Intensive Care Unit (ICU) of a McGill University affiliated hospital in Montreal moved into a newly built setting with single-patient rooms. This study aimed to describe pain intensity and satisfaction with pain management of patients who were hospitalized in the new ICU 6–12 months post-move.

**Methods**: A descriptive study design was used. Adult patients who were in the ICU ≥24 hours and who could self-report were eligible. An adapted version of the Patient Outcome Questionnaire developed by the American Pain Society Quality of Care Committee was used.

**Results**: Eighty-three patients participated. They had a mean age of 65.27 years (SD, 11.46), and the majority were males (63.9%). The median ICU length of stay was: 3.50 days (min-max = 1–42). A proportion of 23.2% of patients reported severe pain (≥7/10) in the last 24 hours. Regarding their satisfaction with pain management, only 17.3% of them were dissatisfied, and 13.6% were dissatisfied with the way the nurse or physician responded to their pain reports. More than half of patients (53.4%) reported that they waited ≤ 10 minutes for their medication, and 18.9% waited ≥ 30 minutes. Of the 28.7% of patients who requested a change in their pain treatment, 62.5% waited <1h, while 16.7% waited >9h.

**Discussion/Conclusions**: ICU patients experience severe pain. While the majority were satisfied with pain management, some patients waited long periods of time to receive appropriate treatment. Clinicians have to walk long distances in this large unit, which may contribute to longer waiting times.

## Adolescent idiopathic scoliosis: same diagnosis but different pain characteristics

D. D. Ocay^a^, M. L. Ma^b^, N. Saran^c^, S. Marchand^d^, J. A. Ouellet^c^ and C. E. Ferland^e^

^a^Physiology, McGill University, Montreal, Canada; ^b^Experimental Surgery, McGill University, Montreal, Canada; ^c^Orthopedic Surgery, McGill University, Montreal, Canada; ^d^Surgery, Université de Sherbrooke, Sherbrooke, Canada; ^e^Anesthesia, McGill University, Montreal, Canada

**CONTACT** Don Daniel Ocay don.ocay@mail.mcgill.ca

© 2018 D. D. Ocay, M. L. Ma, N. Saran, S. Marchand, J. A. Ouellet, and C. E. Ferland. Published with license by Taylor & Francis Group, LLC.

This is an Open Access article distributed under the terms of the Creative Commons Attribution License (http://creativecommons.org/licenses/by/4.0/), which permits unrestricted use, distribution, and reproduction in any medium, provided the original work is properly cited.

**Introduction/Aim**: Limited data exist on chronic pain and its effect on the quality of life of patients with Adolescent Idiopathic Scoliosis (AIS). The purpose of this study was to evaluate the characteristics of the somatosensory functioning and to describe various domains of pain related to the quality of life of these patients.

**Methods**: Patients aged between 10 and 21 years diagnosed with AIS with a history of back pain for more than three months were recruited, completed questionnaires and underwent quantitative sensory testing (QST). Self-report measures included pain intensity, type and location, anxiety and depressive symptoms, disability, and sleep quality. QST measures included mechanical and thermal pain thresholds, temporal summation and conditioned pain modulation efficiency. Inferential analysis was conducted.

**Results**: One hundred and two AIS patients were recruited and reported an average pain of moderate intensity (5/10) located mainly at the thoracic level. Self-report questionnaires revealed that 51% of our cohort had mild to severe functional disability, 74.5% had poor sleep quality, and 21.6% of the patient’s pain had a neuropathic component. In addition, we observed that 9.8% were hypersensitive to touch, 50% were hypersensitive to pressure pain, 45% had a suboptimal or inefficient CPM, and 12.7% demonstrated thermal temporal summation.

**Discussion/Conclusions**: Patients with the same diagnosis, pain intensity and pain location are not experiencing pain similarly, suggesting that patients should not be treated in the same way for pain. Therefore, there is a clinical need for individualized pain management.

## Core behavioural cues in infant clinical pain assessment

Miranda G. DiLorenzo^a^, David B. Flora^b^, Rebecca Pillai Riddell^b^ and Kenneth Craig^c^

^a^Psychology, York University, Toronto, Ontario, Canada; ^b^Psychology, York University, Toronto, Canada; ^c^Psychology, University of British Columbia, Vancouver, Canada

**CONTACT** Miranda DiLorenzo mgdilo@yorku.ca

© 2018 Miranda G. DiLorenzo, David B. Flora, Rebecca Pillai Riddell and Kenneth Craig. Published with license by Taylor & Francis Group, LLC.

This is an Open Access article distributed under the terms of the Creative Commons Attribution License (http://creativecommons.org/licenses/by/4.0/), which permits unrestricted use, distribution, and reproduction in any medium, provided the original work is properly cited.

**Introduction/Aim**: Behavioural cues are widely used in infant pain assessment, but there is a lack of evidence using optimal psychometric strategies to establish their validity for this purpose. We aimed to examine two widely-used coding systems, the Neonatal Facial Coding System (NFCS) and the Modified Behaviour Pain Scale (MBPS), by examining their factor structures with confirmatory factor analysis. (DiLorenzo et al., submitted).

**Methods**: The data is part of an ongoing longitudinal study (OUCH Cohort) that followed caregivers and children from infancy to preschool. The facial expressions of infants were coded using 7 facial action units on the NFCS. Facial expressions, cry, and body movements were coded using MBPS. The factor structure of NFCS and MBPS immediately after the needle and 1-minute post-needle was estimated using data from 2- (*n *= 500) and 12-month vaccinations (*n *= 548).

**Results**: There was warranted removal of weakly associated and redundant items on NFCS and MBPS. An item-reduced NFCS scale with three items had an underlying unidimensional pain factor structure that preserved the good psychometric properties of the 7-item scale. In addition, it was found that one item of the MBPS may be able to capture the construct of pain equally well as the full scale and improve its validity and reliability. Redefinition of MBPS with cry as a sole indicator was suggested.

**Discussion/Conclusions**: This analysis provides new iterations of the NFCS and MBPS that improve the construct validity and internal consistency of both scales. With less items, the revised versions also improve feasibility of use and increase potential for clinical use.

## The second year of life: parent and young child physiological convergence during vaccination

Miranda G. DiLorenzo^a^, Jordana A. Waxman^a^, Rebecca Pillai Riddell^a^ and Hartley Garfield^b^

^a^Psychology, York University, Toronto, Canada; ^b^Pediatrics, University of Toronto, Toronto, Canada

**CONTACT** Miranda G. DiLorenzo mgdilo@yorku.ca

© 2018 Miranda G. DiLorenzo, Jordana A. Waxman, Rebecca Pillai Riddell and Hartley Garfield. Published with license by Taylor & Francis Group, LLC.

This is an Open Access article distributed under the terms of the Creative Commons Attribution License (http://creativecommons.org/licenses/by/4.0/), which permits unrestricted use, distribution, and reproduction in any medium, provided the original work is properly cited.

**Introduction/Aim**: This investigation uses preliminary data to examine the relationships between physiological stress responses of caregivers and their infants during 12-, 18-, and 24-month vaccinations.

**Methods**: The data is part of a new ongoing longitudinal study (OUCH Cardio Cohort) that follows caregiver-infant dyads through the second year of life at 12-month (n = 59), 18-month (n = 34), and 24-month (n = 23) well-baby visits. Caregiver and infant physiological responses (respiratory sinus arrhythmia; RSA) were analyzed for 1-minute prior to the vaccination and 3-minutes post-vaccination. RSA values were calculated on sequential 30-s epochs for the baseline and post-vaccination periods. Pearson correlations were used to examine the convergence between caregiver and infant RSA during the vaccination.

**Results**: During 12-month vaccinations, caregiver and infant RSA were not significantly associated during baseline or post-vaccination periods. At 18-months, significant convergence was found between caregiver and infant baseline RSA (*r *= .37) and caregiver and infant RSA 3-minutes post-needle (*r *= .44). In addition, caregiver and infant RSA were associated 3-minutes post-vaccination (*r *= .51) at the 24-month vaccination.

**Discussion/Conclusions**: Our preliminary results suggest caregiver physiological response patterns converge with infant physiological regulation of pain-related distress at 18 and 24 months of age. Null findings at the 12-month vaccination suggest that convergence between caregivers’ physiological responses and infant physiological reactivity and regulation in the vaccination context does not reliably manifest until after 12 months of age.

## The role of parents in young children’s memories for postsurgical pain

Shanaya Fischer^a^, Jillian Vinall^b^ and Melanie Noel^a^

^a^Department of Psychology, University of Calgary, Calgary, Canada; ^b^Department of Anesthesia, University of Calgary, Calgary, Canada

**CONTACT** Shanaya Fischer sdfische@ucalgary.ca

© 2018 Shanaya Fischer, Jillian Vinall and Melanie Noel. Published with license by Taylor & Francis Group, LLC.

This is an Open Access article distributed under the terms of the Creative Commons Attribution License (http://creativecommons.org/licenses/by/4.0/), which permits unrestricted use, distribution, and reproduction in any medium, provided the original work is properly cited.

**Introduction/Aim**: Children who develop negatively biased memories for pain (i.e., remembering more pain than actually experienced) are at risk for worse future pain and distress, and avoidance of medical care. Factors influencing older children’s memories for pain are child and parent anxiety, and experiencing greater pain. Little is known about which factors influence *younger* children’s memories, when memories are most malleable and parents are most influential. The aim of this study was to identify child and parental factors associated with young children’s memories for postsurgical pain.

**Methods**: Participants included 66 children aged 4 to 7 years and their parents. Before their child’s tonsillectomy, parents completed questionnaires to assess their own trait anxiety and whether they prepared their child for surgery. On the day of surgery, children’s anxiety at anesthesia induction was observationally coded. The following day, children reported on their postsurgical pain intensity. One month later, children recalled their postsurgical pain intensity using the same scale previously administered.

**Results**: Experiencing more postsurgical pain (*p *= 0.002), greater parent trait anxiety (*p *= 0.02), and parents preparing their child for surgery (*p *= 0.04) were associated with more negatively biased memories for pain (*R*^2^ = 0.34). Child anxiety was not associated with memory for pain.

**Discussion/Conclusions**: These findings suggest that *parents* play the most important role in young children’s memories for postsurgical pain. Intervention efforts could focus on 3 risk factors identified from this study: (1) reducing children’s postsurgical pain, (2) reducing parental anxiety, and (3) training parents to optimally prepare their children for an upcoming surgery.

## Caregivers’ Knowledge of Secure Base Scripts is Related to Children’s Behavioural and Physiological Responses to Pain: An Exploratory Analysis

Jodi Martin^a^, Jordana Waxman^a^, Miranda DiLorenzo^a^, Rebecca Pillai Riddell^a^ and Hartley Garfield^b^

^a^Department of Psychology, York University, Toronto, Canada; ^b^Pediatrics, University of Toronto, Toronto, Canada

**CONTACT** Jodi Martin jodimart@yorku.ca

© 2018 Jodi Martin, Jordana Waxman, Miranda DiLorenzo, Rebecca Pillai Riddell and Hartley Garfield. Published with license by Taylor & Francis Group, LLC.

This is an Open Access article distributed under the terms of the Creative Commons Attribution License (http://creativecommons.org/licenses/by/4.0/), which permits unrestricted use, distribution, and reproduction in any medium, provided the original work is properly cited.

**Introduction/Aim**: Caregivers provide important scaffolding of children’s pain-related distress regulation. The success of this process depends in part on caregiver characteristics, like their ability to understand how to provide comfort and support to a distressed child, which is termed “secure base script knowledge” (SBSK). This is the first investigation to explore associations between caregivers’ SBSK and behavioural and physiological indices of children’s pain responses during immunization.

**Methods**: Eleven 24-month-old children and their caregivers provided preliminary data from an ongoing study. Child behavioural and physiological pain responses were collected for 3-minutes post-vaccination. Behavioural responses were coded using the Face Legs Activity Cry Consolability (FLACC) system at 15-second epochs; child physiological responses (respiratory sinus arrhythmia; RSA; lower values reflect adaptive response) were collected in 30-second epochs using MindWare technologies. Caregivers completed the Attachment Script Assessment, a measure of SBSK.

**Results**: Caregiver SBSK was moderately negatively correlated with children’s behavioural (Cohen’s *d *= −1.04, -.85, -.49, -.56) and physiological (Cohen’s *d *= -.68, -.02) responses during the first post-needle minute (pain-reactivity); these associations decreased in magnitude over time, reaching near-zero effect sizes by the start of the second post-needle minute (pain-regulation) (Cohen’s *d *= -.08, -.02, respectively).

**Discussion/Conclusions**: Preliminary findings suggest that children of caregivers with better knowledge of how to provide secure base support (SBSK) had children who demonstrated less behavioural pain-related distress reactivity, and physiological responses to pain that reflect adaptive management of pain reactivity. Findings suggest caregivers’ own relational characteristics influence their child’s reactivity to, but not regulation of, pain at 24 months.

## What factors are predictive of pain intensity during chest tube removal after cardiac surgery?

Ariane Ballard^a^, Melissa Richard-Lalonde^b^, Madalina Boitor^b^ and Céline Gélinas^b^

^a^Faculty of Nursing, University of Montreal, and CHU Sainte-Justine Research Center, Montréal, Québec, Canada; ^b^Ingram School of Nursing, McGill University, and Centre for Nursing Research, CIUSSS Centre-Ouest-Nord-Ile-Montréal - Jewish General Hospital, Montréal, Québec, Canada

**CONTACT** Céline Gelinas celine.gelinas@mcgill.ca Ingram School of Nursing, McGill University, and Centre for Nursing Research, CIUSSS Centre-Ouest-Nord-Ile-Montréal - Jewish General Hospital, Montréal, Québec, Canada

© 2018 Ariane Ballard, Melissa Richard-Lalonde, Madalina Boitor and Céline Gélinas. Published with license by Taylor & Francis Group, LLC.

This is an Open Access article distributed under the terms of the Creative Commons Attribution License (http://creativecommons.org/licenses/by/4.0/), which permits unrestricted use, distribution, and reproduction in any medium, provided the original work is properly cited.

**Introduction/Aim**: Chest tube removal (CTR) is one of the most painful procedure in critically ill patients, and is a standard procedure after cardiac surgery. This study aimed to identify the factors predictive of procedural pain intensity during CTR in intensive care unit (ICU) cardiac surgery patients.

**Methods**: A correlational study design was used. ICU cardiac patients were asked to report their pain intensity and pain unpleasantness before and during CTR using 0–10 Numeric Rating Scales. The 0–8 Critical-Care Pain Observation Tool (CPOT) was used to describe their behavioral responses to CTR. Socio-demographic (e.g., sex, age), number of tubes removed and opioid use were documented. Multiple linear regression was performed to identify significant predictors of pain intensity during CTR.

**Results**: A sample of 192 ICU cardiac surgery patients with a mean age of 65.1 years and 77.6% of them being males participated. Cardiac surgery included coronary artery bypass grafting (63.5%) followed by valve replacement (17.7%), a combination of both (17.2%) and other (1.6%). Only 25% of participants received opioids 1 hour prior to CTR, and a moderate level of pain intensity (5.3 ± 2.9) was reported during CTR. The regression model included five predictors explaining 55.7% of the procedural pain intensity variance (R^2^ = 0.557, F(5, 157) = 39.439, p < .001). Significant predictors were procedural pain unpleasantness (ß = .466, p < .001), procedural CPOT score (ß = .276, p < .001), preprocedural pain intensity (ß = .174, p = .004), gender (ß = .122, p = 024), and number of tubes (ß = .114, p = .037).

**Discussion/Conclusions**: Systematic pain assessment should be done prior to a painful procedure, and appropriate pain management should be provided to patients prior to CTR.

## Chronic non-cancer pain among people who use illicit drugs: prevalence, characteristics, and access to treatment

Jean-Luc Kaboré^a^, Élise Roy^b^, Lise Dassieu^b^, Didier Jutras-Aswad^c^, Julie Bruneau^d^ and Manon Choinière^e^

^a^Department of Pharmacology and Physiology, Faculty of Medicine,Université de Montréal, Montreal, Quebec, Canada; Centre de recherche du Centre hospitalier de l'Université de Montréal, Montréal, Québec, Canada; ^b^Département des sciences de la santé communautaire, Faculté de Médecine et des Sciences de la Santé, Université de Sherbrooke, Longueuil, Quebec, Canada; Chaire de recherche en toxicomanie; Longueuil, Quebec, Canada; ^c^Department of Psychiatry, Faculty of Medicine, Université de Montréal, Montreal, Quebec, Canada; Centre de recherche du Centre hospitalier de l'Université de Montréal, Montréal, Québec, Canada; ^d^Department of Family Medicine and Emergency Medicine, Faculty of Medicine, Université de Montréal, Montreal, Quebec, Canada; Centre de recherche du Centre hospitalier de l'Université de Montréal, Montréal, Québec, Canada; ^e^Department of Anesthesiology and Pain Medicine, Faculty of Medicine, Université de Montréal, Montreal, Quebec, Canada; Centre de recherche du Centre hospitalier de l'Université de Montréal; Montréal, Québec, Canada.

**CONTACT** Jean-Luc Kaboré benewende.jean.luc.kabore@umontreal.ca Centre de recherche du Centre hospitalier de l'Université de Montréal, Tour Saint-Antoine, 850 rue Saint-Denis, Montréal, (Québec), H2X 0A9.

© 2018 Jean-Luc Kaboré, Élise Roy, Lise Dassieu, Didier Jutras-Aswad, Julie Bruneau and Manon Choinière. Published with license by Taylor & Francis Group, LLC.

This is an Open Access article distributed under the terms of the Creative Commons Attribution License (http://creativecommons.org/licenses/by/4.0/), which permits unrestricted use, distribution, and reproduction in any medium, provided the original work is properly cited.

**Introduction/Aim**: The Opioid crisis led to restrictive measures which can limit access to proper pain management, especially for people who use illicit drugs. The aim of this study was to assess prevalence of chronic non-cancer pain (CNCP) and associated factors among people who use drugs (PWUD), and to document how it is managed.

**Methods**: This cross-sectional study was nested within an ongoing prospective cohort study on HIV and HCV infections among PWUD in Montreal. Multivariate logistic regression was used to identify factors associated with CNCP.

**Results**: Overall 389 participants (mean age of 44.6 ± 10.7 years, 83.8% male) completed a questionnaire between February and August 2017. Illicit drug use (last month) was as follows: cocaine (51.7%), heroin (22.6%), prescription opioids (27.3%), cannabis (51.2%). The prevalence of CNCP was 44.0% with moderate to severe intensity and interference in 80.0% and 64.7% of participants respectively. PWUD with CNCP were less likely to report cocaine use (OR = 0.6 (95% CI: 0.4 – 0.9)) but more likely to be male (OR = 2.6 (1.4 – 4.9)), moderately/severely psychologically distressed ((OR = 3.0 (1.8 – 4.9)), with fair/poor global health (OR = 2.0 (1.3 – 3.2)) and anti-HCV positive (OR = 1.8 (1.1 – 2.9)). Among CNCP participants, 18.8% used medications from other people and 19.4% used illicit drugs to relieve their pain. Among those who asked their physician for pain medication, 27.3% were denied prescription.

**Discussion/Conclusions**: Despite the high prevalence, intensity, and interference of CNCP among PWUD, a significant proportion of them have limited access to pain management. Appropriate measures are needed to improve access to optimal CNCP treatment for PWUD.

## Examining the role of healthcare utilization in the relationship between pain and disability among IBD patients

Katherine Fretz^a^, Laura Katz^b^, Alison Crawford^a^, Madelaine Gierc^a^, Valentina Mihajlovic^a^, Dean Tripp^a^ and Michael Beyak^c^

^a^Department of Psychology, Queen’s University, Kingston, Canada; ^b^Michael G DeGroote Pain Clinic, McMaster University Hospital, Hamilton, Canada; ^c^Department of Medicine, Queen’s University, Kingston, Canada

**CONTACT** Katherine Fretz 11kf23@queensu.ca

© 2018 Katherine Fretza, Laura Katzb, Alison Crawforda, Madelaine Gierca, Valentina Mihajlovica, Dean Trippa and Michael Beyakc. Published with license by Taylor & Francis Group, LLC.

This is an Open Access article distributed under the terms of the Creative Commons Attribution License (http://creativecommons.org/licenses/by/4.0/), which permits unrestricted use, distribution, and reproduction in any medium, provided the original work is properly cited.

**Introduction/Aim**: Inflammatory Bowel Disease (IBD) consists of two conditions that affect the GI tract, Crohn’s disease and ulcerative colitis. IBD patients suffer from many physical symptoms (e.g., pain) and psychological difficulties, often resulting in disability. Consequently, IBD is associated with elevated healthcare utilization. The current study aimed to examine the relationships between pain, pain-related disability, and healthcare utilization in IBD patients. It was expected that pain, disability, and healthcare utilization would be significantly, positively related, and that healthcare utilization would mediate the relationship between pain and disability given evidence for its relation to psychological constructs.

**Methods**: A sample of *N* = 299 IBD patients filled out a questionnaire including the following measures: the McGill Pain Questionnaire-Short Form, the Pain Disability Index, and healthcare utilization (i.e., the total number of visits to different healthcare providers over the past 3 months).

**Results**: Healthcare utilization was significantly, positively related to pain (*r* = 0.30, *p* < .001) and pain-related disability (*r* = 0.23, *p* < .001), and pain was significantly, positively related to pain-related disability (*r* = 0.59, *p* < .001). The relationship between pain and disability was not significantly mediated by healthcare utilization (*B* = 0.04, 95% CI contained zero).

**Discussion/Conclusions**: Healthcare utilization seems to be related to important disease-related variables among IBD patients. However, given that disability is a multi-determined biopsychosocial construct, perhaps healthcare utilization is an overly-simplistic mediator to explain significant variability in its relation to pain. These findings highlight the complexity of creating empirical models of factors such as disability.

## Breath awareness in people with chronic pain during a smartphone-based mindfulness task

Muhammad Abid Azam^a^, Vered Valeria Latman^a^, Arielle Sutton^a^, Amir Zarie^a^, Helia Ghazinejad^a^ and Joel Katz^a^

^a^Psychology, York University, Toronto, Ontario, Canada

**CONTACT** Muhammad Abid Azam abidazam@yorku.ca

© 2018 Muhammad Abid Azam, Vered Valeria Latman, Arielle Sutton, Amir Zarie, Helia Ghazinejad and Joel Katz. Published with license by Taylor & Francis Group, LLC.

This is an Open Access article distributed under the terms of the Creative Commons Attribution License (http://creativecommons.org/licenses/by/4.0/), which permits unrestricted use, distribution, and reproduction in any medium, provided the original work is properly cited.

**Introduction/Aim**: Mindful breathing is commonly used in mindfulness approaches to pain management, however, few studies have measured breath awareness during mindfulness meditation (MM) for chronic pain.

**Methods**: This study examined breath awareness during a smartphone-based MM task in 133 participants (Age_M_ = 20.5 years, SD = 3.74; Male = 45, Female = 88, Other = 1). Participants were classified into 3 groups: 1) chronic pain (CP; n = 42) if they self-reported a diagnosed CP condition, 2) depression/anxiety (DA; n = 39) if they reported severe symptoms on either the Center for Epidemiological Studies-Depression subscale (≥21) or Beck Anxiety Inventory (≥36), or 3) controls (n = 52) if not meeting criteria for DA or CP groups. Participants practiced MM for ~12 minutes using a smartphone app. The task involved attending to breathing sensations and pressing “breath” or “other” buttons on a smartphone at the sound of a tone if awareness was on breathing or another experience, respectively. Breath awareness was calculated as the percentage of “breath” responses. Pain intensity was measured before and after MM with a 0–10 numeric rating scale. Group differences in breath awareness were tested with one-way ANOVA.

**Results**: Pain intensity in CP group before (M = 2.62, SD = 2.38) and after (M = 2.07, SD = 2.40) MM did not differ (p = 0.091). ANOVA showed a significant group effect (*p *< 0.05, η^2^ = 0.11), indicating % breath response was higher in CP (M = 58.91%, SD = 0.24, *p *< 0.05) and controls (M = 57.47%, SD = 0.23, *p *< 0.05) than DA (M = 46.29%, SD = 0.24).

**Discussion/Conclusions**: Breath awareness in CP participants was equivalent to controls, suggesting a positive capacity to engage in mindful breathing despite pain. Breath awareness in CP may be useful in studying MM-induced analgesic effects.

## Preliminary results of pain phenotypes in people with Knee Osteoarthritis (KOA): application of IMMPACT recommendations

Lisa C. Carlesso^a^, François Desmeules^a^, Pascal-André Vendittoli^b^, Julio Fernandes^c^, Gil-Roch Bouillon^d^, Manon Choinière^e^, Lars Arendt-Nielsen^f^ and Ariel Desjardins Charbonneau^g^

^a^Centre de Recherche de Hôpital Maisonneuve Rosemont, École de Réadaptation, Faculté de Médecine, Université de Montréal, Montréal, Québec, Canada; ^b^CIUSSS de l’est de l’île, Département de chirurgie, Université de Montréal, Montréal, Québec, Canada; ^c^Orthopedic Research Laboratory, Hôpital du Sacré-Coeur de Montréal and Department of Surgery, Université de Montréal, Montréal, Québec, Canada; ^d^Physio Extra, Clinique MG3, Montréal, Quebec, Canada; ^e^Département d’anesthésiologie et de médecine de la douleur/Centre de recherche du Centre hospitalier de l’Université de Montréal, Université de Montréal, Montréal, Québec, Canada; ^f^Department of Health Science and Technology, School of Medicine, Center for Sensory-Motor Interaction, Aalborg University, Aalborg, Denmark; ^g^Centre de Recherche de Hôpital Maisonneuve Rosemont, Montréal, Québec, Canada

**CONTACT** Lisa C. Carlesso lisa.carlesso@umontreal.ca

© 2018 Lisa C. Carlesso, François Desmeules, Pascal-André Vendittoli, Julio Fernandes, Gil-Roch Bouillon, Manon Choinière, Lars Arendt-Nielsen and Ariel Desjardins Charbonneau. Published with license by Taylor & Francis Group, LLC.

This is an Open Access article distributed under the terms of the Creative Commons Attribution License (http://creativecommons.org/licenses/by/4.0/), which permits unrestricted use, distribution, and reproduction in any medium, provided the original work is properly cited.

**Introduction/Aim**: To 1. identify pain phenotypes (PP) in people with KOA using the PP domains recommended by the IMMPACT Group and their relationship to disease specific function, and 2. compare PPs using neurophysiological tests (NPT) versus the Central Sensitization Inventory (CSI) for assessing peripheral and central sensitization.

**Methods**: Participants with KOA were recruited from three Montreal area hospitals. Latent profile analysis (LPA) was used to determine PPs using the recommended domains of pain intensity, variability and quality, sleep, psychological factors, somatization, fatigue, neuropathic pain, and NPT. Linear regression was used to determine the relation of the PPs to disease specific function using the ADL subscale of the KOOS questionnaire. The LPA was rerun with the CSI substituted for NPT measures.

**Results**: 82 participants were included (mean age 63; BMI 33.1 kg/m^2^, 59% women). Three PPs were identified: mild, moderate, and severe groups demonstrating progressive levels of pain intensity, specific pain descriptors, poor sleep, anxiodepressive symptoms, catastrophization, somatization, fatigue, and neuropathic pain. All groups demonstrated signs of peripheral or central sensitization according to NPT results. In an adjusted model, the severe group reported significantly worse function compared to the mild group (ß −26.4 95%CI (−35.7, −17.0)). Substitution of the CSI did not replicate NPT results

**Discussion/Conclusions**: Three PPs of progressive severity using domains recommended by IMMPACT were identified with the most severe group reporting clinically important loss of function compared to the mild group. Substitution of the CSI to identify peripheral or central sensitization identified by NPT measures was not successful.

## Prospective analysis of chronic non-cancer pain patients receiving chronic methadone as analgesic. A five-years methadone registry

Jordi Perez^a^, Marc O. Martel^b^ and Yoram Shir^a^

^a^Alan Edwards Pain Management Unit, McGill University Health Centre, Montreal, Canada; ^b^Faculties of Dentistry & Medicine, McGill University, Montreal, Canada

**CONTACT** Jordi Perez Jordi.Perez@MUHC.MCGILL.CA

© 2018 Jordi Perez, Marc O. Martel and Yoram Shir. Published with license by Taylor & Francis Group, LLC.

This is an Open Access article distributed under the terms of the Creative Commons Attribution License (http://creativecommons.org/licenses/by/4.0/), which permits unrestricted use, distribution, and reproduction in any medium, provided the original work is properly cited.

**Introduction/Aim**: Although methadone is commonly used for the treatment of chronic noncancer pain (CNCP), very few longitudinal studies have been conducted to examine long-term treatment outcomes among patients prescribed methadone. A methadone registry was developed at the Alan Edwards Pain Management Unit to prospectively assess treatment outcomes among CNCP patients prescribed methadone therapy.

**Methods**: The methadone registry currently includes a total of 97 patients. Among these patients, a subset (n = 21) of new methadone users underwent baseline assessment (i.e., before initiating methadone) and were then assessed at fixed follow-up time points over a 5-year period. A subset (n = 76) of patients were already using methadone at the time of enrolment into the registry. These patients also underwent follow-up assessment procedures over a 5-year period. During each of the follow-up visits, patients completed questionnaires assessing pain intensity, pain interference, side effects, and methadone therapy satisfaction.

**Results**: Multilevel modeling analyses indicated that pain intensity and pain interference levels decreased significantly among methadone users across the 5-year period (both p’s < .05). Results indicated that roughly 65% of patients were satisfied with methadone therapy. Patients’ levels of satisfaction with methadone therapy were more strongly influenced by pain (B = -.18; < .001) than by side effects (B = -.04; < .05). Examination of data revealed that side effects associated with methadone were most often reported as being “mild”.

**Discussion/Conclusions**: Our preliminary data suggest that methadone therapy may be accompanied by treatment improvements, acceptable side effects, and generally high patient-reported satisfaction levels.

## Salivary Alpha-Amylase assessment as a proxy of pre-operative anxiety in pediatric patients

Shajenth Premachandran^a^, Ljiljana Nikolajev^b^, Jean A. Ouellet^c^ and Catherine E. Ferland^d^

^a^Anatomy and Cell Biology, McGill University, Montreal, Quebec, Canada; ^b^Shriners Hospital for Children-Canada, Montreal, Quebec, Canada; ^c^Orthopedic Surgery, McGill University, Montreal, Quebec, Canada; ^d^Anesthesia, McGill University, Montreal, Quebec, Canada

**CONTACT** Shajenth Premachandran shajenth.premachandran@mail.mcgill.ca

© 2018 Shajenth Premachandran, Ljiljana Nikolajev, Jean A. Ouellet and Catherine E. Ferland. Published with license by Taylor & Francis Group, LLC.

This is an Open Access article distributed under the terms of the Creative Commons Attribution License (http://creativecommons.org/licenses/by/4.0/), which permits unrestricted use, distribution, and reproduction in any medium, provided the original work is properly cited.

**Introduction/Aim**: Patients undergoing major surgery are subject to both physical and psychological stress, which influence their recovery. In clinical settings, patient’s stress or anxiety is evaluated using self-report measurements that are not necessarily the most reliable method of determining the physiological stress levels of a patient, due to its subjectivity and several potential biases. Finding new means to evaluate anxiety levels before and after surgery would help minimize its impact and lead to better postsurgical recovery. Salivary Alpha-Amylase (sAA) is a biomarker of the activity of the sympathetic nervous system and has been shown to increase as a result of both psychological and physical stress. The project’s goal is to identify any changes in the level of sAA during the preoperative and postoperative periods, and correlate it with the self-reported scores on the visual analog scale for anxiety (VAS-A), in pediatric patients undergoing spinal surgery. The aim of this study was to assess the reliability of salivary alpha-amylase as an accurate marker of pediatric pre-operative stress.

**Methods**: Thirty patients with adolescent idiopathic scoliosis scheduled to undergo corrective surgery were recruited and enrolled in the study. Saliva samples were collected 1 week before surgery (baseline), on the morning of surgery, 24 and 48 hours after surgery and six weeks after surgery. Samples were processed and analyzed using Salimetrics® α-Amylase kinetic enzyme assays. In parallel, patients were asked to self-report their level of anxiety at each saliva collection with the use of the visual analog scale for anxiety (VAS-A). ANOVAs were performed to identify changes over time. Correlation analyses were performed for associations between sAA levels and VAS-A scores.

**Results**: SAA concentrations did not increase in activity on the morning of surgery as hypothesized (0.729 nkat/L ± 0.406 at baseline compared to 0.700 nkat/L ± 0.430 on the morning of surgery, p > 0.05), but rather increased significantly during the 48-hour period after surgery (1.449 nkat/L ± 1.398 sAA activity, p = 0.024). Furthermore, no associations were found between sAA levels and VAS-A scores at any time point.

**Discussion/Conclusions**: AA concentrations may have increased in the 48-hour period of the post-operative period, as a result of the invasiveness of spinal surgery that puts a tremendous amount of physical stress on the body of pediatric patients. The findings suggest that sAA is not a reliable marker of pre-operative stress/anxiety in pediatric patients undergoing surgery, but rather a marker of surgical stress.

## The role of resilience, optimism, and self-compassion in the treatment of patients with acute back pain

Andrea Aternali^a^, Rebekah Wickens^a^, Arthur Woznowski-Vu^a^ and Timothy H. Wideman^a^

^a^School of Physical and Occupational Therapy Montreal, McGill University, Montreal, Canada

**CONTACT** Andrea Aternali andrea.aternali@mail.mcgill.ca

© 2018 Andrea Aternali, Rebekah Wickens, Arthur Woznowski-Vu, and Timothy H. Wideman. Published with license by Taylor & Francis Group, LLC.

This is an Open Access article distributed under the terms of the Creative Commons Attribution License (http://creativecommons.org/licenses/by/4.0/), which permits unrestricted use, distribution, and reproduction in any medium, provided the original work is properly cited.

**Introduction/Aim**: The current study aimed to elucidate the psychological factors that affect the treatment response of patients acutely suffering from back pain and work disability. Depression and catastrophizing have been previously associated with worsened treatment response; however, the role of resilience, optimism, and self-compassion have been minimally investigated.

**Methods**: Thirty-eight adults living with daily non-specific back pain (< 6 months) and enrolled in a physiotherapy or rehabilitation program reported their average pain, pain-related disability (Pain Disability Index), resilience (Connor-Davidson Resilience scale), optimism (Life Orientation Test-Revised), and self-compassion (Self-Compassion Scale). At follow-up three months after, patients reported their average pain and pain-related disability once again. Bivariate Pearson’s correlations compared patients’ psychological variables to their changes in pain and pain-related disability scores over the three-month period.

**Results**: Higher optimism and resilience were correlated with a reduction in average pain ratings and pain-related disability, respectively, *r*(37) = -.365, *p *= .024, *r*(*38*) = -.401, *p* = .013. Interestingly, self-compassion was not significantly associated with average reported pain or pain-related disability (*rs* > |.221|, *ps* > .141), although it was positively correlated with optimism (*r*(43) = .508, *p *< .001) and resilience (*r*(43) = .515, *p *< .001).

**Discussion/Conclusions**: This is the first study to indicate that patients’ optimism and resilience, but not self-compassion, predict improvements in back pain and pain-related disability. Since treatment response factors during the early stages of pain modulate the risk of chronicity, future studies may explore early interventions that address these psychological traits.

## Development and implementation of an interdisciplinary self-management for chronic pain program: patient evaluation and feedback

Matilda E. Nowakowski^a^, Graham Nishikawa^b^, Cathy Page^b^, Harsha Shanthanna^c^, Mauricio Forero^c^, Philip Chan^c^, Heather Radman^b^ and Julie Holmes^b^

^a^Department of Psychiatry and Behavioural Neurosciences/Chronic Pain Clinic, McMaster University/St. Joseph’s Healthcare Hamilton, Hamilton, Canada; ^b^Chronic Pain Clinic, St. Joseph’s Healthcare Hamilton, Hamilton, Canada; ^c^Department of Anesthesia/Chronic Pain Clinic, McMaster University/St. Joseph’s Healthcare Hamilton, Hamilton, Canada

**CONTACT** Matilda E. Nowakowski mnowakow@stjoes.ca

© 2018 Matilda E. Nowakowski, Graham Nishikawa, Cathy Page, Harsha Shanthanna, Mauricio Forero, Philip Chan, Heather Radman and Julie Holmes. Published with license by Taylor & Francis Group, LLC.

This is an Open Access article distributed under the terms of the Creative Commons Attribution License (http://creativecommons.org/licenses/by/4.0/), which permits unrestricted use, distribution, and reproduction in any medium, provided the original work is properly cited.

**Introduction/Aim**: The Chronic Pain Clinic at St. Joseph’s Healthcare Hamilton received funding in 2014 from the Ministry of Health and Long-Term Care in Ontario to implement a biopsychosocial approach to pain management and develop an interdisciplinary self-management for chronic pain program. We summarize patient feedback for the expansion of services.

**Methods**: Patients attended an 8-week self-management for chronic pain program that included both exercise therapy and cognitive-behaviour therapy interventions. Patients evaluated the impact of the program on physical, emotional and social functioning.

**Results**: A total of 53 patients (*Mean* age = 58 years) with chronic pain completed the group program. Patients presented with a variety of chronic pain conditions with lower back pain being the most common (74%). On a 7-point scale where 1 = “not at all” and 7 = “extremely”, patients rated their satisfaction with the cognitive-behavioural and exercise therapy components of the program as 6.75 (*SD* = .52) and 6.68 (*SD* = .61), respectively. On a 7-point scale where 1 = “not at all” and 7 = “extremely”, patients’ rated the degree of improvement after completing the program as greater than 5 in the following domains: range of motion and strength, posture form and awareness, managing and coping with chronic pain, coping with anxiety and stress, coping with anger and frustration, self-esteem and self-worth, independence and ability to conduct daily activities, and functioning in family and social relationships.

**Discussion/Conclusions**: Patients reported that the program helped to increase their confidence in coping and managing with their chronic pain.

## Understanding the relationship between pre-surgical pain and functioning with acute post-surgical activity: A prospective actigraphy trajectory analysis after pediatric orthopedic surgery

Brittany N. Rosenbloom^a^, Gabrielle Pagé^b^, Lisa Isaac^c^, Fiona Campbell^c^, Shima Razavi^c^, Meghan Rossi^c^, Jennifer Stinson^c,d^ and Joel Katz^a^

^a^Psychology, York University, Toronto, ON, Canada; ^b^Centre de recherche du Centre hospitalier de l’Université de Montréal, Montreal, QC, Canada; ^c^Anesthesia and Pain Medicine, The Hospital for Sick Children, University of Toronto, Toronto, ON, Canada; ^d^Faculty of Nursing, University of Toronto, Toronto, Canada

**CONTACT** Brittany Rosenbloom bnrosen@yorku.ca

© 2018 Brittany N. Rosenbloom, Gabrielle Pagé, Lisa Isaac, Fiona Campbell, Shima Razavi, Meghan Rossi, Jennifer Stinson and Joel Katz. Published with license by Taylor & Francis Group, LLC.

This is an Open Access article distributed under the terms of the Creative Commons Attribution License (http://creativecommons.org/licenses/by/4.0/), which permits unrestricted use, distribution, and reproduction in any medium, provided the original work is properly cited.

**Aims**: The fear-avoidance model of pain and activity has not been explored in youth undergoing major surgeries. The study aims are to examine (1) acute post-surgical physical activity trajectories and (2) their associated pre-surgical and in-hospital predictors (e.g., pain, anxiety, general functioning).

**Methods**: A subgroup of patients and their parents/guardians from a larger prospective, longitudinal study were included in this study if they had a typical in-hospital stay (i.e. did not go to ICU). Patients (n = 238; 41.18% male, mean age = 14.10 years, SD = 2.48) were recruited before surgery and followed daily while in hospital, and 6 and 12 months later. Data was collected by questionnaire and chart review. Physical activity was measured in hospital, continuously for four days using a non-invasive Actical (Philips Respironics) motion biosensor.

**Results**: Growth mixture modeling indicated the best fitting model comprised four different trajectories of acute post-surgical activity with a significant linear term (Akiake information criterion = 1173.69). The trajectories varied by activity level on Day 1 after surgery and in the rate of increased or maintained activity over the first four days post-surgically. Significant predictors of trajectory group membership included pre-surgical functioning and in-hospital anxiety. Activity trajectories were significantly associated with daily reported movement-evoked pain. The least active trajectories had the lowest pre-surgical functioning and highest movement-evoked pain scores.

**Discussion/Conclusion**: This study shows that youth undergoing major orthopedic surgeries have different physical activity trajectories in the days after surgery. Further research will evaluate whether these trajectories predict development of long-term pain and functional outcomes.

## Neonatal pain management practices: mothers’ evaluation of parent targeted educational tools

Ligyana Candido^a^, Denise Harrison^b^, Maria Veríssimo^a^ and Mariana Bueno^c^

^a^Maternal-Child and Psychiatric Nursing, School of Nursing of the University of São Paulo, São Paulo, Brazil; ^b^Children’s Hospital of Eastern Ontario, university of Ottawa, Ottawa, Ontario, Canada; ^c^Child Health Evaluative Sciences, The Hospital for Sick Children, Toronto, Ontario, Canada

**CONTACT** Ligyana Korki de Candido ligyanakorki@usp.br

© 2018 Ligyana Candido, Denise Harrison, Maria Veríssimo and Mariana Bueno. Published with license by Taylor & Francis Group, LLC.

This is an Open Access article distributed under the terms of the Creative Commons Attribution License (http://creativecommons.org/licenses/by/4.0/), which permits unrestricted use, distribution, and reproduction in any medium, provided the original work is properly cited.

**Introduction/Aim**: To evaluate mothers’ perception on feasibility, acceptability, and usefulness of two educational tools on neonatal pain management.

**Methods**: This is a cross-sectional study nested in a pragmatic clinical trial, where allocation into three groups was according to mothers’ preferences and availability. Mothers admitted in a rooming-in unit received the pamphlet on neonatal procedural pain management practices, and were invited to participate in daily educational sessions performed by nurses where the Portuguese version of the ‘Be Sweet to Babies’ video was presented. At hospital discharge, mothers answered a questionnaire. The study protocol was approved by local ethics review board.

**Results**: 51 mothers comprised the analyses of this sub study: 18 mothers watched the video and read the pamphlet, 17 watched the video, and 16 read the pamphlet and were included in this sub study. Most mothers were not aware of analgesic effects of breastfeeding (57%), SSC (63%), and sweet solutions (83%). 23% mothers had already breastfed and 4% provided SSC to their infants’ during painful procedures. After watching the video and/or reading the pamphlet, all mothers intended to use or advocate for the use of at least one of this analgesic strategies. Both educational tools were considered as useful, easy to understand and to apply in real scenarios, with ideal length, and recommendable to other parents.

**Discussion/Conclusions**: Mothers’ considered both educational tools as feasible, acceptable and useful. Future studies are needed to explore the effects of parental education on neonatal pain outcomes such as pain assessment and management.

## Feasibility and acceptability of a web-based and in-person self-management intervention aimed at preventing chronic pain after major lower extremity trauma (iPACT-E-Trauma)

Mélanie Bérubé^a,b^, Céline Gélinas^a,c^, Nancy Feeley^a,c^, Géraldine Martorella^d^, José Côté^e^, George-Yves Laflamme^b^, Dominique Rouleau^b^ and Manon Choinière^f^

^a^Ingram School of Nursing, McGill University, Montreal, Quebec, Canada; ^b^CIUSSS du Nord-de l’Île-de-Montréal, Hôpital du Sacré-Cœur de Montréal, Montreal, Quebec, Canada; ^c^Centre for Nursing Research, CIUSSS Centre-Ouest-Île-Montréal, Jewish General Hospital, Montreal, Montreal, Quebec, Canada; ^d^College of Nursing, Florida State University, Tallahassee, FL, USA; ^e^Faculté des sciences infirmières, Université de Montréal, Montreal, Quebec, Canada; ^f^Department of Anesthesiology, Université de Montréal, Montreal, Quebec, Canada

**CONTACT** Mélanie Bérubé melanie.berube2@mail.mcgill.ca

© 2018 Mélanie Bérubé, Céline Gélinas, Nancy Feeley, Géraldine Martorella, José Côté, George-Yves Laflamme, Dominique Rouleau and Manon Choinière. Published with license by Taylor & Francis Group, LLC.

This is an Open Access article distributed under the terms of the Creative Commons Attribution License (http://creativecommons.org/licenses/by/4.0/), which permits unrestricted use, distribution, and reproduction in any medium, provided the original work is properly cited.

**Introduction/Aim**: Transition from acute to chronic pain frequently occurs after major lower extremity trauma, and no intervention exists to prevent this transition in this specific population. We developed a 7-session hybrid, web-based and in-person, self-management intervention to prevent acute to chronic pain transition after major lower extremity trauma (iPACT-E-Trauma). The goal of this study was to assess the feasibility and acceptability of this intervention.

**Methods**: Using a descriptive design, the intervention was initiated at a level-1 trauma center. Twenty-eight patients were recruited. Feasibility assessment examined whether the intervention could be provided as planned and if participants’ could complete the intervention in at least 80%. Acceptability assessment was performed using the E-Health Acceptability Questionnaire and the Treatment Acceptability and Preference Questionnaire.

**Results**: More than 80% of participants received sessions’ components. However, 71.4% of session 2 web pages, which include a significant amount of actionable content, were accessed. Sessions were delivered according to the established timeline for ≥ 80% of participants. Session 3 needed to be provided earlier. Session duration was 30 minutes or less on average, as initially planned. More than 80% of participants attended sessions and the majority were able to apply self-management behaviors, with the exception of deep breathing relaxation exercises. Most session features were evaluated as very acceptable.

**Discussion/Conclusions**: Findings showed that the iPACT-E-Trauma intervention is feasible and was perceived as highly acceptable by participants. Further tailoring iPACT-E-Trauma to patients’ needs, providing more training time for relaxation techniques, and modifying the web platform to improve its convenience are required.

## Differential predictors of the presence and intensity of dyspareunia and genito-pelvic pain in pregnancy

Meghan A. Rossi^a^, Kayla Mooney^b^, Ron George^c^, Jill Chorney^c^, Caroline Pukall^b^, Erna Snelgrove-Clarke^d^ and Natalie O. Rosen^a,e^

^a^Department of Psychology and Neuroscience, Dalhousie University, Halifax, NS, Canada; ^b^Department of Psychology, Queen’s University, Kingston, ON, Canada; ^c^Department of Anesthesia, Pain Management, and Perioperative Medicine, Dalhousie University, Halifax, NS, Canada; ^d^School of Nursing, Dalhousie University, Halifax, NS, Canada; ^e^Department of Obstetrics and Gynaecology, IWK Health Centre, Nova Scotia, Halifax, Canada

**CONTACT** Meghan Rossi meghan.rossi@dal.ca

© 2018 Meghan A. Rossi, Kayla Mooney, Ron George, Jill Chorney, Caroline Pukall, Erna Snelgrove-Clarke and Natalie O. Rosen. Published with license by Taylor & Francis Group, LLC.

This is an Open Access article distributed under the terms of the Creative Commons Attribution License (http://creativecommons.org/licenses/by/4.0/), which permits unrestricted use, distribution, and reproduction in any medium, provided the original work is properly cited.

**Introduction/Aim**: This study examined the prevalence of genito-pelvic pain (GPP) and dyspareunia (pain during intercourse) in first time mothers 18–24 weeks pregnant, and examined predictors of these pain types.

**Methods**: Women (*N* = 403) pregnant for the first-time completed an online survey assessing dyspareunia, GPP, sexual distress, depressive symptoms, pain catastrophizing, and intensity of non-genito-pelvic pain (non-GPP). A multinomial logistic regression assessed whether these variables predicted membership to statistically derived pain groups. Linear regressions tested whether the predictors were associated with greater intensity of GPP and dyspareunia.

**Results**: 7 (1.7%) women reported GPP alone, 244 (60.5%) reported dyspareunia alone, 31 (7.7%) reported both pain types, and 121 (30.1%) reported neither pain. Using the “Neither pain” group as the reference category, greater sexual distress, depressive symptoms, and intensity of non-GPP significantly predicted having both pain types. Sexual distress and intensity of non-GPP predicted having dyspareunia only, whereas the intensity of non-GPP significantly predicted having GPP alone, χ^2^(12) = 63.16, *p* < .01. Greater non-GPP intensity was associated with greater intensity of GPP, *F*(4, 64) = 3.23, *p < *.01, whereas greater sexual distress and intensity of non-GPP were unique predictors of greater intensity of dyspareunia, *F*(4,242) = 13.94, *p* < .05.

**Discussion/Conclusions**: This study was the first to assess the prevalence and predictors of dyspareunia and GPP in pregnancy. Findings suggest that a large portion of women experience dyspareunia and exhibit differential predictors compared to those who report only GPP, highlighting the importance of assessing these pain types separately.

## Efficacy of conservative interventions for pain associated with trapeziometacarpal (thumb base) osteoarthritis: a systematic review

Tokiko Hamasaki^a,b,c^, Sylvain Laprise^d^, Patrick Harris^a,c,e,f^, Nathalie J. Bureau^a,g,h^, Nathaly Gaudreault^i,j^, Lyne Lalonde^a,k^, Daniela Ziegler^l^ and Manon Choinière^a,m^

^a^Research Center of the Centre hospitalier de l’Université de Montréal (CHUM), Montreal, Quebec, Canada; ^b^School of Rehabilitation, Faculty of Medicine, Université de Montréal (UdeM), Montreal, Quebec, Canada; ^c^Hand Center, CHUM, Montreal, Quebec, Canada; ^d^Physiotherapie MyoSynergie, Amos, Quebec, Canada; ^e^Plastic Surgery Service, Department of Surgery, CHUM, Montreal, Quebec, Canada; ^f^Department of Surgery, Faculty of Medicine, UdeM, Montreal, Quebec, Canada; ^g^Department of Radiology, Radio-Oncology and Nuclear Medicine, Faculty of Medicine, UdeM, Montreal, Quebec, Canada; ^h^Department of Radiology, CHUM, Montreal, Quebec, Canada; ^i^School of Rehabilitation, Faculty of Medicine and Health Sciences, Université de Sherbrooke, Sherbrooke, Quebec, Canada; ^j^Research Center of the Centre hospitalier universitaire de Sherbrooke, Sherbrooke, Quebec, Canada; ^k^Faculty of Pharmacy,UdeM, Montreal, Quebec, Canada; ^l^CHUM Library, Montreal, Quebec, Canada; ^m^Department of Anesthesiology and Pain Medicine, Faculty of Medicine, UdeM, Montreal, Quebec, Canada

**CONTACT** Manon Choiniére manon.choiniere@umontreal.ca

© 2018 Tokiko Hamasaki, Sylvain Laprise, Patrick Harris, Nathalie J. Bureau, Nathaly Gaudreault, Lyne Lalonde, Daniela Ziegler and Manon Choinière. Published with license by Taylor & Francis Group, LLC.

This is an Open Access article distributed under the terms of the Creative Commons Attribution License (http://creativecommons.org/licenses/by/4.0/), which permits unrestricted use, distribution, and reproduction in any medium, provided the original work is properly cited.

**Introduction/Aim**: Trapeziometacarpal osteoarthritis (TMO) is painful and affects 5–7% of adults in North America. Unfortunately, TMO management is not optimal since knowledge of evidence-based treatments is lacking. This systematic review (SR) aims to document therapeutic efficacy of all existing interventions for TMO.

**Methods**: Our protocol was based on the *Cochrane Handbook for SRs of Interventions*. Relative effect size of intervention was extracted from SRs when available, otherwise estimated from randomized controlled trials (RCTs). If RCTs were not available, non-RCTs were consulted.

**Results**: Through 16 bibliographic databases, 21 SRs, 49 RCTs, and 44steroid>hyaluronate  non-RCTs met the inclusion criteria. Relative effects (superior>inferior) and quality of evidence (VL = very low; L = low; M = moderate; H = high) of the following conservative interventions for pain were as follows: *(1) medications*: naproxen>control(C)(VL); transdermal steroids>C(L); saline injections>C(L-M); saline>steroid injections(VL), steroid>hyaluronate injections(VL); dextrose prolotherapy>steroid injections except for short-term(ST)(VL); hyaluronate>saline injections(VL); *(2) rehabilitation*: hard orthosis>C except for ST if night-time wearing(VL); soft orthosis>C(L); soft>hard orthosis(VL); hybrid>soft orthosis(VL); wrist-based>hand-based orthosis except for pain during pinch(VL); thumb>joint exercises(L); joint mobilization>C except at 1 week follow-up(VL); nerve mobilization>C(VL); exercises-joint protection>C(VL); manual therapy-exercises>C(VL); laser therapy = C(L); and *(3) others*: leech therapy>diclofenac gel(VL); acupuncture>C(VL); stinging nettle>C(VL).

**Discussion/Conclusions**: This SR allowed collating comprehensive evidence on the efficacy of a variety of conservative interventions for TMO. Inclusion of SRs, RCTs, and non-RCTs reinforced our exhaustive search of the relevant references without duplicating SRs. Superiority among saline, steroid and hyaluronate injections remains unknown. Furthermore, more research is needed to provide better evidence.

## Assessment of the role of spinal mGluR5 and anchoring proteins in nociception

Nitasha Gill^a^, Andre Laferrière^a^ and Terence J. Coderre^a^

Anesthesia, McGill University, Montreal, Quebec, Canada

**CONTACT** Nitasha Gill nitasha.gill@mail.mcgill.ca

© 2018 Nitasha Gill, Andre Laferrière and Terence J. Coderre. Published with license by Taylor & Francis Group, LLC.

This is an Open Access article distributed under the terms of the Creative Commons Attribution License (http://creativecommons.org/licenses/by/4.0/), which permits unrestricted use, distribution, and reproduction in any medium, provided the original work is properly cited.

**Introduction/Aim**: The observed increase in nuclear mGluR5 in rats with sciatic nerve injury or hind paw inflammation may be explained by the actions of proteins that affect mGluR5 trafficking. Homer proteins regulate trafficking and intracellular signaling associated with mGluR5. Induction of Homer1a has been shown to affect localization of mGluR5. Thus, acutely Homer1a acts homeostatically to prevent glutamate-induced excitotoxicity. Homer1a induction causes synaptic remodeling which may lead to the development of nociceptive hypersensitivity after nerve injury. This provides the rationale for our investigation into the modulatory role of Homer1 proteins on cell surface and nuclear mGluR5 in nociception. We hypothesize that Homer1a and Homber1b/c affect the trafficking and signaling of cell surface and nuclear mGluR5 which leads to the development of nociceptive hypersensitivity after nerve injury.

**Methods**: To determine whether Homer1a is able to affect the localization of mGluR5, a Homer1a mimic peptide, Tat-mGluR5ct, was used in naïve male Long-Evans rats. Tat-mGluR5ct was injected intrathecally. Spinal tissue was extracted 48 hours later followed by subfractionation and western blot. To assess whether Tat-mGluR5ct is able to reduce nociceptive hypersensitivity, Tat-mGluR5ct was injected intrathecally 30 minutes before 20 nmol of quisqualate was spinally administered. Sustained nociceptive behaviours were recorded for a period of 30 minutes after quisqualate injection.

**Results**: *Western blot -* 48 hours after Tat-mGluR5ct injection there was a marked decrease in nuclear mGluR5 expression relative to vehicle-injected rats. *Nociceptive testing -* Tat-mGluR5ct reduced quisqualate-induced sustained nociceptive behaviours.

**Discussion/Conclusions**: Homer1a appears to play an important role in spinal mGluR5 localization and nociceptive hypersensitivity and warrants further study.

## Circulating levels of monoamines in pediatric patients undergoing orthopedic surgery

Ljiljana Nikolajev^a^, Neil Saran^b^, Jean A. Ouellet^c^ and Catherine E. Ferland^d^

^a^Shriners Hospital for Children-Canada, Montreal, Canada; ^b^Pediatrics, Shriners Hospital for Children-Canada, McGill University, Montreal, Canada; ^c^Orthopedics, Shriners Hospital for Children-Canada, McGill University, Montreal, Canada; ^d^Anesthesia, Shriners Hospital for Children-Canada, McGill University, Montreal, Canada

**CONTACT** Ljiljana Nikolajev ljiljana.nikolajev@mail.mcgill.ca

© 2018 Ljiljana Nikolajev, Neil Saran, Jean A. Ouellet and Catherine E. Ferland. Published with license by Taylor & Francis Group, LLC.

This is an Open Access article distributed under the terms of the Creative Commons Attribution License (http://creativecommons.org/licenses/by/4.0/), which permits unrestricted use, distribution, and reproduction in any medium, provided the original work is properly cited.

**Introduction/Aim**: Spinal fusion surgery is one of the most invasive orthopaedic surgeries and the resulting postoperative pain is far too often under-treated. The role of the descending monoaminergic pathways in pain modulation has been extensively studied. Monoamine neurotransmitters, which include serotonin (5-HT) and the catecholamines dopamine (DA), epinephrine (EPI), and norepinephrine (NE), play a key role in pain modulation by regulating the release of neurotransmitters from nociceptive afferents. This study aims to enhance the current knowledge of the endogenous pain control system in a surgical context.

**Methods**: 102 patients aged between 12 and 18 years old and scheduled to undergo spinal fusion surgery, were enrolled in this prospective cohort study. At each time point throughout the study, pain was rated by the patient using a numerical rating score (0–10). Blood samples were collected and analyzed by mass spectrometry. Monoamine level differences over time were analyzed using repeated measures ANOVA.

**Results**: Variation in time of all monoamine levels were observed throughout the perioperative period (p < 0.0001). Six weeks after spinal fusion, plasma concentrations of NE, normetanephrine (NME) and DA remained drastically increased compared to baseline (p˂0.0001, p = 0.001, p = 0.0113 respectively). Interestingly, a significant correlation was found between perioperative plasma NE and NME and persistence of pain, weeks after surgery (r = 0.48, 0.50; p < .002).

**Discussion/Conclusions**: These results suggest that the addition of pharmacological interventions targeting descending monoaminergic pathways to treat postoperative pain may improve pain management after surgery.

## Usability and safety of a virtual reality distraction intervention to reduce procedural pain in children with cancer

Yalinie Kulandaivelu^a^, Kathryn A. Birnie^a^, Lindsay Jibb^b^, Petra Hroch^c^, Karyn Positano^d^, Fiona Campbell^e^, Simon Robertson^f^, Oussama Abla^d^ and Jennifer Stinson^a^

^a^Child Health Evaluative Sciences, The Hospital for Sick Children, Toronto, ON, Canada; ^b^Nursing Sciences, Faculty of Health Sciences, University of Ottawa, Ottawa, ON, Canada; ^c^Department of Anesthesia, University of Toronto, Toronto, ON, Canada; ^d^Division of Hematology/Oncology, The Hospital for Sick Children, Toronto, ON, Canada; ^e^Department of Anesthesia and Pain Medicine, The Hospital for Sick Children, University of Toronto, Toronto, ON, Canada; ^f^KindVR, Alameda, CA, USA

**CONTACT** Yalinie Kulandaivelu yalinie.kulandaivelu@sickkids.ca

© 2018 Yalinie Kulandaivelu, Kathryn A. Birnie, Lindsay Jibb, Petra Hroch, Karyn Positano, Fiona Campbell, Simon Robertson, Oussama Abla and Jennifer Stinson. Published with license by Taylor & Francis Group, LLC.

This is an Open Access article distributed under the terms of the Creative Commons Attribution License (http://creativecommons.org/licenses/by/4.0/), which permits unrestricted use, distribution, and reproduction in any medium, provided the original work is properly cited.

**Aim**: Subcutaneous port (SCP) needle insertions are distressing for youth with cancer. Virtual reality (VR) distraction offers promise for reducing needle-related pain and distress given its highly immersive and interactive audiovisual environment. The aim was to assess and refine usability (i.e., acceptability, ease of use) and safety of a custom VR intervention in youth with cancer undergoing SCP access.

**Methods**: Three iterative cycles of usability sessions were conducted with youth with cancer aged 8–18 years old either prior to or during SCP access. Participants were asked to “think aloud” while using the VR, then participated in a semi-structured interview regarding VR usability and recommendations for improvement. Observations and feedback were used to refine the VR intervention.

**Results**: Over 3 cycles of testing, 17 youth with cancer participated (M = 11.7 years old, SD = 3.51; n = 12 male.) All participants reported the VR was easy to use, enjoyed the VR intervention, and understood the game objective. The majority of participants (n = 16) were interested in using the VR intervention during subsequent SCP procedures. Refinements focused on: (1) increasing level of VR responsiveness, interaction and immersion, (2) ability for youth to be alerted to procedural steps conducted by clinical staff during SCP access, and (3) preventing hardware from touching sterile areas and excess patient movement. No adverse events were reported (e.g., nausea, dizziness).

**Discussion/Conclusions**: Youth with cancer found the VR distraction intervention to be acceptable, easy to use and safe for SCP procedures. Next steps include feasibility testing using a pilot randomized controlled trial.

## The effect of the ABCD’s of pain management on parent pain ratings: Is parent psychological distress a moderator?

Hannah Gennis^a^, Shaylea Badovinac^a^, Rebecca Pillai Riddell^a^, Joel Katz^a^, Hartley Garfield^b^ and Saul Greenberg^b^

^a^Psychology, York University, Toronto, Ontario, Canada; ^b^Pediatrics, University of Toronto, Toronto, Ontario, Canada

**CONTACT** Hannah Gennis hgennis@yorku.ca

© 2018 Hannah Gennis, Shaylea Badovinac, Rebecca Pillai Riddell, Joel Katz, Hartley Garfield and Saul Greenberg. Published with license by Taylor & Francis Group, LLC.

This is an Open Access article distributed under the terms of the Creative Commons Attribution License (http://creativecommons.org/licenses/by/4.0/), which permits unrestricted use, distribution, and reproduction in any medium, provided the original work is properly cited.

**Introduction/Aim**: The ABCD’s of pain management video (Pillai Riddell et al., 2017) has been shown to impact parent soothing and young child pain during vaccination, with parent psychological distress as a moderator (Gennis et al., under review). The current study explores whether the ABCD video has an effect on parent ratings of child pain post-needle, and whether this is also moderated by parent psychological distress.

**Methods**: Parents of 6- and 18-month-olds (n = 64 each) were randomized to a video treatment - The ABCD’s (**A**ssess anxiety, **B**elly breathe, **C**alm close cuddle, and **D**istraction) of pain management or a placebo video. Parents completed the Brief Symptom Inventory – 18 (BSI 18; Derogatis, 2001), a measure of psychological distress. They also rated their child’s pain from 0 to 10 following the child’s last needle.

**Results**: A 2 (Age: 6- vs. 18 months) X 2 (Treatment: ABCD vs. Placebo Video) ANOVA revealed no effect of Age, Treatment, or an interaction on parent pain ratings post-needle (p’s > .05). Moderation analyses revealed that at 18 months, parent psychological distress moderated the effect of treatment video on parent pain ratings, = .52, *p* = .02. Parents in the treatment group who reported low psychological distress had lower pain ratings, = ‒.97, *p* < .001.

**Discussion/Conclusions**: Parents of toddlers exposed to the ABCD video reported lower pain scores if their psychological distress was lower. These findings, together with past research, suggest that psychological distress is an important consideration when developing parent psychoeducation strategies for vaccination.

## Factors associated with pain resolution following surgical fixation of tibia fractures

Yaping Chang^a^, Jason W. Busse^b^, Mohit Bhandari^c^, Wei Qi^d^, Li Wang^e^, Diane Heels-Ansdell^f^, Qi Zhou^g^, Mei Wang^h^, Kan Lun Zhu^i^, Brad Petrisor^j^, Lin Jin^k^, Sean Alexander Kennedy^l^, Sheila Sprague^m^, Paula McKay^n^, Nicole Simunovic^o^, Shiyun Hu^p^, Yuan Zhang^q^, Lehana Thabane^r^, Gordon H Guyatt and on behalf of the TRUST Investigators^s^

^a^Department of Health Research Methods, Evidence, and Impact, McMaster University, Hamilton, Ontario, Canada, changy28@mcmaster.ca,@YapingChangMc; ^g^Department of Health Research Methods, Evidence, and Impact, McMaster University, Hamilton, Ontario, Canada, qzhou@mcmaster.ca; ^e^Department of Anesthesia, McMaster University, Hamilton, Ontario, Canada; Michael G. DeGroote Institute for Pain Research and Care, Department of Anesthesia, McMaster University, Hamilton, Ontario, Canada, lwang246@gmail.com,@lwang246; ^c^Department of Health Research Methods, Evidence, and Impact, McMaster University, Hamilton, Ontario, Canada; Division of Orthopaedic Surgery, Department of Surgery, McMaster University, Hamilton, Ontario, Canada, bhandam@mcmaster.ca; ^b^Department of Health Research Methods, Evidence, and Impact, McMaster University, Hamilton, Ontario, Canada; Department of Anesthesia, McMaster University, Hamilton, Ontario, Canada; Michael G. DeGroote Institute for Pain Research and Care, Department of Anesthesia, McMaster University, Hamilton, Ontario, Canada,bussejw@mcmaster.ca,@JasonWBusse; ^d^Outpatient Clinic, Tianjin Centres for Disease Control and Prevention, Tianjin, China; ^f^Department of Health Research Methods, Evidence, and Impact, McMaster University, Hamilton, Ontario, Canada, ansdell@mcmaster.ca; ^n^Division of Orthopaedic Surgery, Department of Surgery, McMaster University, Hamilton, Ontario, Canada, mckayp@mcmaster.ca; ^h^Department of Health Research Methods, Evidence, and Impact, McMaster University, Hamilton, Ontario, Canada, wangm59@mcmaster.ca; ^i^Department of Medicine, McMaster University, Hamilton, Ontario, Canada, zhukl@mcmaster.ca; ^j^Division of Orthopaedic Surgery, Department of Surgery, McMaster University, Hamilton, Ontario, Canada, petrisb@mcmaster.ca; ^k^Department of Health Research Methods, Evidence, and Impact, McMaster University, Hamilton, Ontario, Canada, jinl9@mcmaster.ca; ^l^Department of Diagnostic Radiology, University of Toronto, Toronto, Ontario, Canada, sean.kennedy@medportal.ca; ^m^Department of Health Research Methods, Evidence, and Impact, McMaster University, Hamilton, Ontario, Canada; Division of Orthopaedic Surgery, Department of Surgery, McMaster University, Hamilton, Ontario, Canada, sprags@mcmaster.ca; ^o^Division of Orthopaedic Surgery, Department of Surgery, McMaster University, Hamilton, Ontario, Canada, simunon@mcmaster.ca; ^p^Center for Cardio-cerebrovascular Disease Prevention and Control Research, Zhejiang Hospital, Hangzhou, Zhejiang, China; ^q^Department of Health Research Methods, Evidence, and Impact, McMaster University, Hamilton, Ontario, Canada, zhang243@mcmaster.ca; ^r^Department of Health Research Methods, Evidence, and Impact, McMaster University, Hamilton, Ontario, Canada; Centre for Evaluation of Medicines, St. Joseph’s Healthcare Hamilton, McMaster University, Hamilton, Ontario, Canada, thabanl@mcmaster.ca; ^s^Department of Health Research Methods, Evidence, and Impact, McMaster University, Hamilton, Ontario, Canada; Department of Medicine, McMaster University, Hamilton, Ontario, Canada, guyatt@mcmaster.ca

**CONTACT** Yaping Chang changy28@mcmaster.ca Department of Health Research, Methods Evidence, and Impact McMaster University, Hamilton, Canada, Ontario

© 2018 Yaping Chang, Jason W. Busse, Mohit Bhandari, Wei Qi, Li Wang, Diane Heels-Ansdell, Qi Zhou, Mei Wang, Kan Lun Zhu, Brad Petrisor, Lin Jin, Sean Alexander Kennedy, Sheila Sprague, Paula McKay, Nicole Simunovic, Shiyun Hu, Yuan Zhang, Lehana Thabane and Gordon H Guyatt. Published with license by Taylor & Francis Group, LLC.

This is an Open Access article distributed under the terms of the Creative Commons Attribution License (http://creativecommons.org/licenses/by/4.0/), which permits unrestricted use, distribution, and reproduction in any medium, provided the original work is properly cited.

**Aim**: We used data collected as part of the TRial to evaluate Ultra Sound in the Treatment of tibial Fractures (TRUST) to assess the association between baseline factors and resolution of pain among patients with surgically managed tibial fractures.

**Methods**: We defined our study outcome, the resolution of post-surgical pain, as two consecutive follow-ups in which patients reported no more than mild pain (pain score ≤3 on a 0–10 Numeric Rating Scale).

**Results**: We included 483 patients with open or closed tibial fractures managed with surgical fixation. At 12-month follow-up, 313 of 483 (64.8%) participants met criteria for pain resolution. We found significant and independent associations between male sex (HR = 1.34 [95% CI, 1.04 to 1.72]), non-smoking (HR = 1.74 [95% CI, 1.33 to 2.29]) and alcohol consumption (HR = 1.35 [95% CI, 1.06 to 1.73]) with resolution of post-surgical pain. Age, obesity, diabetes, closed or open fracture, presence of multi-trauma and post-operative weight-bearing status were not associated with resolution of pain.

**Conclusions**: Our findings suggest that non-medical factors may play an important role in the development of persistent pain following surgical fixation of tibial fractures. Further prospective studies are needed to confirm or refute our findings.

References1.Busse
JW, Bhandari
M, Einhorn
TA, Heckman JD, Leung KS, Schemitsch E, Tornetta P, Walter SD, Guyatt GH. Trial to re-evaluate ultrasound in the treatment of tibial fractures (TRUST): a multicenter randomized pilot study. Trials.
2014;15:206.10.1186/1745-6215-15-206PMC4060850248989872.Busse
J W, Bhandari
M, Einhorn
T A, Schemitsch E, Heckman JD, Tornetta P, Leung KS, Heels-Ansdell D, Makosso-Kallyth S, Della Rocca GJ, Jones CB. Re-evaluation of low intensity pulsed ultrasound in treatment of tibial fractures (TRUST): randomized clinical trial. The BMJ. 2016;355:i5351.2779778710.1136/bmj.i5351PMC50804473.Castillo
RC, MacKenzie
EJ, Wegener
ST, Bosse MJ, LEAP Study Group. Prevalence of chronic pain seven years following limb threatening lower extremity trauma. Pain. 2006;124(3):321–29.1678106610.1016/j.pain.2006.04.0204.Larsen
P, Elsoe
R, Hansen
SH, Graven-Nielsen
T, Laessoe
U, Rasmussen
S.
Incidence and epidemiology of tibial shaft fractures. Injury. 2015;46(4):746–50.2563653510.1016/j.injury.2014.12.0275.Radresa
O, Chauny
J M, Lavigne
G, Piette E, Paquet J, Daoust R. Current views on acute to chronic pain transition in post-traumatic patients: risk factors and potential for pre-emptive treatments. J Trauma Acute Care Surg. 2014;76(4):1142–50.2466288310.1097/TA.00000000000001886.Rivara
FP, MacKenzie
EJ, Jurkovich
GJ, Nathens AB, Wang J, Scharfstein DO. Prevalence of pain in patients 1 year after major trauma. Arch Surg. 2008;143(3):282–87.1834727610.1001/archsurg.2007.617.Treede
R D, Rief
W, Barke
A, Aziz Q, Bennett MI, Benoliel R, Cohen M, Evers S, Finnerup NB, First MB, Giamberardino MA. A classification of chronic pain for ICD-11. Pain. 2015;156(6):1003.2584455510.1097/j.pain.0000000000000160PMC44508698.Walker-Bone
K, Harvey
NC, Ntani
G, Tinati T, Jones GT, Smith BH, Macfarlane GJ, Cooper C. Chronic widespread bodily pain is increased among individuals with history of fracture: findings from UK Biobank. Arch Osteoporos. 2016;11(1):1.2667849110.1007/s11657-015-0252-1PMC4683164

## Availability of E-health tools for pediatric pain assessment and management: barriers, facilitators, costs, and design

Kristen S. Higgins^a,b^, Perri R. Tutelman^a,b^, Christine T. Chambers^b,c^, Holly O. Witteman^d,e,f^, Melanie Barwick^g,h^, Penny Corkum^i,j,k^, Doris Grant^l^, Jennifer N. Stinson^m,n^,  Chitra Lalloo^n,o^, Sue Robins^p^, Rita Orji^q^ and Isabel Jordan^r^

^a^Department of Psychology and Neuroscience, Dalhousie University, Halifax, Nova Scotia, Canada; ^b^Centre for Pediatric Pain Research, IWK Health Centre, Halifax, Nova Scotia, Canada; ^c^Departments of Pediatrics and Psychology and Neuroscience, Dalhousie University, Halifax, Nova Scotia, Canada; ^d^Department of Family & Emergency Medicine and Office of Education and Professional Development, Faculty of Medicine, Université Laval, Quebec City, Quebec, Canada; ^e^Centre de recherche sur les soins et les services de première ligne de l’Université Laval, CHU de Quebec, Quebec City, Quebec, Canada; ^f^Ottawa Hospital Research Institute, Ottawa, Ontario, Canada; ^g^The Hospital for Sick Children, Child and Youth Mental Health Research Unit, Child Health Evaluative Sciences, Research Institute, Toronto, Ontario, Canada; ^h^Department of Psychiatry and Dalla Lana School of Public Health, University of Toronto, Toronto, Ontario, Canada; ^i^Departments of Psychology and Neuroscience and Psychiatry, Dalhousie University, Halifax, Nova Scotia, Canada; ^j^Department of Pediatrics, Scientific Staff, IWK Health Centre, Halifax, Nova Scotia, Canada; ^k^Colchester East Hants Health Authority, Colchester East Hants ADHD Clinic, Truro, Nova Scotia, Canada; ^l^Industry Liaison and Innovation, Dalhousie University, Halifax, Nova Scotia, Canada; ^m^Child Health Evaluative Sciences and Chronic Pain Program, The Hospital for Sick Children, Toronto, Ontario, Canada; ^n^Lawrence S. Bloomberg Faculty of Nursing, University of Toronto, Toronto, Ontario, Canada; ^o^Child Health Evaluative Sciences, The Hospital for Sick Children, Toronto, Ontario, Canada; ^p^Patient Advocate and Partner, Bird Communications, Vancouver, British Columbia, Canada; ^q^Faculty of Computer Science, Dalhousie University, Halifax, Nova Scotia, Canada; ^r^Patient Partner, Squamish, British Columbia, Canada

**CONTACT** Kristen S. Higgins Kristen.higgins@dal.ca

© 2018 Kristen S. Higgins, Perri Tutelman, Christine T. Chambers, Holly Witteman, Melanie Barwick, Penny Corkum, Doris Grant, Jennifer Stinson and Chitra Lalloo. Published with license by Taylor & Francis Group, LLC.

This is an Open Access article distributed under the terms of the Creative Commons Attribution License (http://creativecommons.org/licenses/by/4.0/), which permits unrestricted use, distribution, and reproduction in any medium, provided the original work is properly cited.

**Introduction/Aim**: Numerous e-health tools to assist with pediatric pain assessment/management have been developed and evaluated with promising results regarding effectiveness. Although considerable resources are spent developing/evaluating these tools with the rationale that they will increase access to care, current evidence suggests they are rarely made available to end users, reducing their impact and creating research waste. This study extends previous work by examining barriers/facilitators to availability, development/evaluation costs, and relationships between user-centered design and availability.

**Methods**: A systematic review was conducted to identify papers describing e-health tools for pediatric pain assessment/management published in English in the past 10 years. Corresponding authors of included papers (one per tool) were then invited to complete an online survey about barriers/facilitators to tool availability, grant funding used, and a measure of user-centeredness of design process (UCD-11).

**Results**: Ninety papers describing 53 tools met inclusion criteria. Twenty-six survey responses were received (49.06%); 13 described available tools (*n *= 5 to the general public; *n *= 8 to patients of particular clinics/systems). Commonly endorsed facilitators of availability included beliefs in benefits to target population and research field; barriers included lack of infrastructure and time. An average of $398,006.72 CAD was spent on developing/evaluating each unavailable tool (M_available tools_ = $601,175.79 CAD). Available tools scored significantly higher than unavailable tools on UCD-11, *t*(16.01) = −2.33, *p *< 0.05.

**Discussion/Conclusions**: Systemic changes to academic and funding structures are needed to support tool availability and reduce research waste. The role of user-centered design principles and improved use of a priori implementation research methods in supporting availability should be further explored in future studies.

## Functional effects of TrkA inhibition on system x_C_^–^mediated glutamate release and cancer-induced bone pain

Tanya Miladinovic^a^, Robert G Ungard^a^, Katja Linher-Melville^a^, Snezana Popovic^a^ and Gurmit Singh^a^

Pathology and Molecular Medicine, McMaster University, Hamilton, Ontario, Canada

**CONTACT** Tanya Miladinovic miladint@mcmaster.ca

© 2018 Tanya Miladinovic, Robert G Ungard, Katja Linher-Melville, Snezana Popovic and Gurmit Singh. Published with license by Taylor & Francis Group, LLC.

This is an Open Access article distributed under the terms of the Creative Commons Attribution License (http://creativecommons.org/licenses/by/4.0/), which permits unrestricted use, distribution, and reproduction in any medium, provided the original work is properly cited.

**Introduction/Aim**: Breast cancer has a propensity to metastasize to the bone microenvironment, causing severe cancer-induced bone pain (CIBP). Breast cancer cells release glutamate via the system x_C_^−^ antiporter, which is up-regulated to exchange extracellular cystine for intracellular glutamate to protect against oxidative stress. Here, we demonstrate that system x_C_^−^ is functionally influenced by the actions of the neurotrophin NGF on its cognate receptor tyrosine kinase, TrkA, and that inhibiting this complex may reduce CIBP via downstream actions on xCT, the functional subunit of system x_C_^−^.

**Methods**: Murine 4T1 and human MDA-MB-231 triple-negative carcinoma cells were treated with recombinant β-NGF with or without the selective TrkA inhibitor AG879. Effects on system x_C_^−^ were characterized using crystal violet, MTT, Western blotting, qPCR, and cystine uptake assays. *In vivo*, naïve female Balb/c mice were inoculated with 2 × 10^4^ 4T1 cells percutaneously into the right distal femur to establish tumours. Animals were treated with AG879 via intraperitoneally-implanted osmotic pumps, and nociception was quantified throughout tumour progression. Animals were transcardially perfused and femurs were immunohistochemically stained to quantify patterns of functional system x_C_^−^ activity at the tumour site.

**Results**: AG879 abolished β-NGF-induced increases in xCT mRNA and protein levels, and significantly decreased functional system x_C_^−^ activity. *In vivo*, systemic treatment with AG879 inhibited nociceptive and physiologically relevant responses in tumor-bearing animals.

**Discussion/Conclusions**: Cumulatively, these data suggest that the activation of TrkA by NGF has functional implications on system x_C_^–^mediated cancer pain. This pathway therefore presents a promising target for therapeutic intervention in cancer pain treatment.

## Predictors of persistent post surgical pain, functional disability and return to work following the surgical lumbar microdiscectomy: a systematic review

Yasir Rehman^a^, Malgorzata M. Bala^b^, Arnav Agarwal^c^, Nadia Rehman^d^, Magdalena Koperny^e^, Savannah Grodecki^f^, Anna Wrzosek^g^, Wiktoria Lesniak^h^, Nathan Evaniew^i^, Li Wang^j^, Rachel Couban^j^, Brian Drew^k^, Gordon Guyatt^l^ and Jason Busse^a^

^a^Health Research Methods, Evidence and Impact, McMaster University, Hamilton, Ontario, Canada; ^b^Department, Chair of Epidemiology and Preventive Medicine, Jaiellonian Univeristy Medical College, Krakow, Lesser Poland, Poland; ^c^School of Medicine, University of Toronto, Toronto, Ontario, Canada; ^d^Pain Center, McMaster University, Hamilton, Ontario, Canada; ^e^Public Health and Health Promotion Department, Province Sanitary Epidemiological Station in Krakow, Krakow, Małopolska Voivodeship, Poland; ^f^Department of Medicine, Royal College of Surgeons in Ireland, Dublin, Ireland; ^g^Department of Interdisciplinary Intensive Care, Jagiellonian University, Collegium Medicum, Krakow, Poland; ^h^2nd Department of Internal Medicine, Jagiellonian University Medical College, Krakow, Poland; ^i^Department and Division, Orthopaedics, Department of Surgery, McMaster University, Hamilton, ON, Canada; ^j^Department of Anesthesia, McMaster University, Hamilton, ON, Canada; ^k^Department of Surgery, McMaster University, Hamilton, Ontario, Canada; ^l^Department of Medicine, Department of Health Research Methods, Evidence, and Impact, McMaster University, Hamilton, Ontario, Canada

**CONTACT** Yasir Rehman dry_rehman@yahoo.ca/rehmany@mcmaster.ca Health Research Methods, Evidence and Impact, McMaster University, Hamilton, Ontario, Canada

© 2018 Yasir Rehman, Malgorzata M. Bala, Arnav Agarwal, Nadia Rehman, Magdalena Koperny, Savannah Grodecki, Anna Wrzosek, Wiktoria Lesniak, Nathan Evaniew, Li Wang, Rachel Couban, Brian Drew, Gordon Guyatt and Jason Busse. Published with license by Taylor & Francis Group, LLC.

This is an Open Access article distributed under the terms of the Creative Commons Attribution License (http://creativecommons.org/licenses/by/4.0/), which permits unrestricted use, distribution, and reproduction in any medium, provided the original work is properly cited.

**Introduction**: Persistent post surgical pain (PPSP) following lumbar decompression for sciatica is reported by 10–40% of patients, and is associated with reduced quality of life and loss of productivity. Our aim was to systematically review the published literature to determine which factors are associated with PPSP, functional disability and failure to return to work (RTW).

**Methods**: In collaboration with an experienced medical librarian, we searched Medline, Embase, Psychinfo and Pubmed from inception to January 2017. Eligible studies explored, in an adjusted model, predictors of PPSP, functional impairment and unemployment. Literature screening, data extraction and risk of bias were completed independently and in duplicate. The GRADE approach was used to evaluate the quality of evidence.

**Results**: Our search identified 23 eligible studies with 2,766 participants. Limitations of the literature, such as inadequate reporting, precluded statistical pooling of measures of association. Low to moderate quality evidence showed a significant association between negative outcome expectations, fear of movement, somatization, and poor coping skills and PPSP, functional disability and failure to RTW. Also low to moderate quality evidence showed that longer wait time until surgery and higher pain intensity at baseline predicted PPSP and functional disability, respectively. Age, degenerative changes, examination findings, side and level of disc herniation were not associated with outcomes.

**Discussion/Conclusion**: Our results suggest that psychological factors and higher preoperative pain intensity are associated with poor outcome following lumbar decompression surgery for sciatica.

## Impact of the Adjuvanted Recombinant Zoster Vaccine on pain and use of pain medication in Adults Aged ≥50 Years

Robert Johnson^a^ and on behalf of the ZOE-50/70 study group

University of Bristol, Faculty of Health Sciences, Bristol, United Kingdom

**CONTACT** Robert Johnson rwjbristol@alumni.virginia.edu

© 2018 GlaxoSmithKline Biologicals SA. Published with license by Taylor & Francis Group, LLC.

This is an Open Access article distributed under the terms of the Creative Commons Attribution License (http://creativecommons.org/licenses/by/4.0/), which permits unrestricted use, distribution, and reproduction in any medium, provided the original work is properly cited.

**Introduction/Aim**: To determine the efficacy of an adjuvanted recombinant Zoster vaccine (RZV) in reducing the burden of illness (BOI, i.e. pain severity*duration*incidence) and use of pain-related medication in HZ episodes.

**Methods**: The assessments were integrated in two Phase III trials, ZOE-50 (subjects ≥50 years of age [YOA], NCT01165177) and ZOE-70 (subjects ≥70 YOA, NCT01165229). Pain was assessed by the Zoster Brief Pain Inventory (ZBPI) instrument. We report results of the ZOE-50 and a pooled analysis of subjects ≥70 YOA from the two trials combined. In addition, use of HZ-related pain medication was recorded.

**Results**: The estimated vaccine efficacy (VE) in reducing the HZ BOI was 98.4% and 92.1% in the ZOE-50 study and the pooled analysis of subjects ≥70 YOA, respectively. In confirmed cases, RZV significantly reduced the maximal ZBPI worst pain score in the pooled analysis (p = 0.032) and the maximal ZBPI average pain scores in both the ZOE-50 (p = 0.049) and the pooled analysis (p = 0.043). The use of medication for HZ pain was reported for six (66.6%) and 190 (74.8%) patients in the RZV and placebo groups, respectively for the ZOE-50 study (p = 0.697). Corresponding numbers in the pooled analysis were 11 (44.0%) and 205 (72.2%) patients, respectively (p = 0.006).

**Discussion/Conclusions**: RZV significantly reduced the HZ BOI, particularly by a very high VE in preventing HZ. For HZ breakthrough cases, the results suggest that RZV mitigated the severity of HZ-related pain compared to placebo HZ cases. Additionally, use of pain medication was reduced significantly in adults ≥70 YOA.

## Mechanisms of spinal hyperexcitability in rat and human models of pathological pain

Annemarie Dedek^a,b^, Jian Xu^c^, Chaya M. Kandegedara^a,b^, Eve C. Tsai^b,d,e^, Paul J. Lombroso^c^ and Mike E. Hildebrand^a,b^

^a^Department of Neuroscience, Carleton University, Ottawa, Ontario, Canada; ^b^Department of Neuroscience, Ottawa Hospital Research Institute, Ottawa, Ontario, Canada; ^c^The Child Study Centre, Yale University, Yale University School of Medicine, New Haven, Connecticut, USA; ^d^Faculty of Medicine, University of Ottawa, Ottawa, Ontario, Canada; ^e^Division of Neurosurgery, The Ottawa Hospital, Ottawa, Ontario, Canada

**CONTACT** Annemarie Dedek annemariededek@cmail.carleton.ca

© 2018 Crown. Published with license by Taylor & Francis Group, LLC.

**Introduction/Aim**: Chronic pain arises when there is an imbalance between excitation and inhibition of neurons in the spinal superficial dorsal horn. The mechanisms underlying this imbalance remain unclear in rodent models, and unexplored in human tissue. We have recently shown that in nerve-injured rats, BDNF-mediated disinhibition gates potentiation of GluN2B-containing NMDARs through Fyn kinase activation at lamina I dorsal horn synapses. We will explore whether loss of an associated phosphatase, STEP_61_, mediates this pathological coupling in lamina I neurons of rodents and humans. To investigate mechanisms of human spinal pain signaling, we have, for the first time, functionally characterized synaptic NMDAR responses in human lamina I neurons.

**Methods**: We paired patch-clamp electrophysiological recordings with pharmacology, behaviour, and biochemical approaches. We used an *ex vivo* BDNF spinal pathology model in rodent and human tissue. To model chronic inflammatory pain, we administered an *in vivo* injection of CFA into the rodent hindpaw. Human tissue was collected from organ donors 1–4 hours *post-mortem.*

**Results**: In humans and rats, we observed a decrease in STEP_61_ and an increase in pGluN2B and pFyn at lamina I synapses. Downregulation of STEP_61_ was both necessary and sufficient to prime subsequent phosphorylation and potentiation of synaptic NMDARs by BDNF. Preliminary data suggests that GluN2B-containing NMDARs dominate synaptic NMDAR responses in human lamina I neurons.

**Discussion/Conclusions**: STEP_61_ is the molecular brake that is lost to drive the potentiation of excitatory NMDAR responses following BDNF-mediated disinhibition at lamina I synapses of rodents and humans. Like rats, GluN2B-containing NMDARs dominate human lamina I synaptic responses.

## Effects of therapeutic clown distraction on children’ pain and anxiety during vaccination: a pilot study

Patricia A. Laforce^a^, Ariane Ballard^a^, Pilar Ramirez-Garcia^a^ and Sylvie LeMay^a^

Sciences infirmières, Université de Montréal, Montréal, Québec, Canada

**CONTACT** Patricia A. Laforce patricia.a.laforce@umontreal.ca

© 2018 Patricia A. Laforce, Ariane Ballard, Pilar Ramirez-Garcia and Sylvie LeMay. Published with license by Taylor & Francis Group, LLC.

This is an Open Access article distributed under the terms of the Creative Commons Attribution License (http://creativecommons.org/licenses/by/4.0/), which permits unrestricted use, distribution, and reproduction in any medium, provided the original work is properly cited.

**Introduction**: Distraction by a therapeutic clown is a promising intervention in procedural pain and anxiety management, but no study has been done in the context of vaccination.

**Methods**: A experimental pilot study with two groups, therapeutic clown (TC) and usual care (UC) was conducted. Children’s pain was assessed using the Face, Legacy, Activity, Cry, Consolability (FLACC) and anxiety with the Visual Analog Scale (VAS).

**Results**: A total of 24 children aged 2 to 17, their parent and a nurse participated in the study. All the families covering the inclusion criteria agreed to participate and completed the study. In addition, preliminary results show that both pain (median TC = 1 and IQ = 1.5 vs median UC = 2.5 and IQ = 2.5) than anxiety (median TC = 1 and difference IQ = 0.5 vs median UC = 4 and IQ = 2) were lower in children in the TC, as well as in their parent (median TC = 0 and IQ = 0 vs median UC = 2 and IQ = 4, 5). The nurse felt no anxiety at all when vaccinating children in the TC group (median TC = 0 and IQ = 0) and only slight anxiety with children in the UC group (median UC = 0 and IQ = 2).

**Discussion/conclusion**: Distraction by a therapeutic clown is a feasible and acceptable intervention that appears to help reduce the pain and anxiety of children and the anxiety of their parent and nurse during child immunization.

**KEYWORDS** Procedural pain; therapeutic clown; children; vaccination; needle-related procedure

## Preliminary analyses of a randomized controlled trial of an online chronic pain treatment for military and police

Pamela L. Holens^a,b^, Jeremiah Buhler^b^, Michelle Paluszek^b^, Kristen Klassen^c^, Luigi Imbrogno^d^, Adair Libbrecht^b^ and Brent Joyal^b^

^a^Department of Clinical Health Psychology, University of Manitoba, Winnipeg, MB, Canada; ^b^Department of Psychology, University of Manitoba, Winnipeg, MB, Canada; ^c^Disability Studies, University of Manitoba, Winnipeg, MB, Canada; ^d^External Relations, University of Manitoba, Winnipeg, MB, Canada

**CONTACT** Pamela L. Holens pholens@deerlodge.mb.ca

© 2018 Pamela L. Holens, Jeremiah Buhler, Michelle Paluszek, Kristen Klassen, Luigi Imbrogno, Adair Libbrecht and Brent Joyal. Published with license by Taylor & Francis Group, LLC.

This is an Open Access article distributed under the terms of the Creative Commons Attribution License (http://creativecommons.org/licenses/by/4.0/), which permits unrestricted use, distribution, and reproduction in any medium, provided the original work is properly cited.

**Introduction/Aim**: Rates of chronic pain in military and police populations are estimated to be double that of the general population. Chronic pain treatments range from medications to psychotherapies. Acceptance-based behavioural therapies (ABBT) have been shown to be particularly effective for individuals suffering from chronic pain. A pilot study of an online ABBT developed specifically for military and police populations with chronic pain has shown promise in improving pain-related variables (i.e., pain-related catastrophizing, kinesiophobia, pain acceptance). A randomized controlled trial (RCT) of this treatment is currently underway. The purpose of this poster is to present early findings of the RCT at approximately the halfway point of data collection.

**Methods**: To examine the effectiveness of this program, independent samples t-tests were conducted comparing changes in pain-related measures between a wait-list control group (n = 12) and a treatment group (n = 10).

**Results**: The wait-list and control groups were comparable on demographic variables and pre-treatment measures. Compared to the wait-list group, the treatment group demonstrated significant improvement on a measure of chronic pain acceptance and approached significance on a measure of kinesiophobia. The groups did not differ on measures of pain-related disability and pain catastrophizing.

**Discussion/Conclusions**: Although only half of the data has been collected for this RCT, preliminary results suggest that the online ABBT program is effective in increasing individuals’ acceptance of their pain condition and decreasing their pain-related fear of movement. Strengths, limitations, and implications of these results will be discussed.

## Site-specific phosphorylation of microglial P2X7R contributes to altered nociception in opioid tolerance and in neuropathic pain

Heather Leduc-Pessah^a^, Nicole E. Burma^a^, Alexandra Pilapil^a^, Rebecca Dalgarno^a^ and Tuan Trang^a^

Departments of Comparative Biology & Experimental Medicine and Physiology & Pharmacology, Hotchkiss Brain Institute, University of Calgary, Calgary, AB, Canada

**CONTACT** Heather Leduc-Pessah hlleducp@ucalgary.ca

© 2018 Heather Leduc-Pessah, Nicole E. Burma, Alexandra Pilapil, Rebecca Dalgarno and Tuan Trang. Published with license by Taylor & Francis Group, LLC.

This is an Open Access article distributed under the terms of the Creative Commons Attribution License (http://creativecommons.org/licenses/by/4.0/), which permits unrestricted use, distribution, and reproduction in any medium, provided the original work is properly cited.

**Introduction**: Chronic pain is a pervasive clinical problem that profoundly impacts the quality of life of afflicted individuals and their families. Overlapping mechanisms involving the microglial P2X7R have been found to contribute to both the development of hypersensitivity in neuropathic pain and the adverse effects that interfere with opioid analgesia, such as the development of opioid tolerance. Understanding this common mechanism may explain the lack of efficacy of opioids in neuropathic pain and improve the management of neuropathic chronic pain. Therefore, we further investigated the role of microglial P2X7R in opioid tolerance and neuropathic pain.

**Methods**: Cultured BV2 microglial cells and Sprague Dawley rats were used to assess the role of microglial P2X7R expression and function in response to morphine treatment and in a model of peripheral nerve injury (PNI).

**Results**:Using P2X7R mimetic peptides and mutant P2X7R constructs, we identified a putative tyrosine phosphorylation site on the P2X7R that contributes to changes in functional response but does not inhibit intrinsic receptor function. Blockade of this site *in vivo* attenuated the development of morphine tolerance and reversed established allodynia in a PNI model. Collectively, our findings demonstrate a critical role for phosphorylation of the P2X7R which contributes to the loss of anti-nociception in morphine tolerance and to mechanical allodynia in neuropathic pain.

**Conclusions**: This new converging P2X7R cellular mechanism can be therapeutically targeted for the management of neuropathic pain and to improve the pain-relieving actions of opioid analgesics.

## Stabilizing mast cells to treat pain: evidences from pre-clinical and clinical studies

Carolina B. Meloto^a^, Pablo Ingelmo^b^, Eduardo Vega Perez^c,d^, Rebecca Pitt^c^, Victor Hugo Gonzalez Cardenas^c,e^, Nada Mohamed^c^, Susana G. Sotocinal^f^, Jeffrey S. Mogil^g^ and Luda Diatchenko^h^

^a^McGill University, Faculty of Dentistry, The Alan Edwards Centre for Research on Pain, Montreal, Quebec, Canada; ^b^Chronic Pain Service, Montreal Children’s Hospital. The Alan Edwards Centre for Research on Pain, Montreal, Quebec, Canada; ^c^Chronic Pain Service, Montreal Children’s Hospital, Montreal, Quebec, Canada; ^d^Department of Anesthesia, School of Medicine, Pontifical Catholic University of Chile, Santiago, Chile; ^e^Fundacion Universitaria de Ciencias de la Salud, Bogotá, Colombia; ^f^Department of Psychology, McGill University, Montreal, Quebec, Canada; ^g^Department of Psychology and Anesthesia, and The Alan Edwards Centre for Research on Pain, Montreal, Quebec, Canada; ^h^The Alan Edwards Centre for Research on Pain, McGill University, Montreal, Quebec, Canada

**CONTACT** Carolina B. Meloto carol.meloto@mcgill.ca 740 Av. Dr. Penfield #2300, H3A0G1, Montreal, Quebec, Canada, (514) 398-2833

© 2018 Carolina B. Meloto, Pablo Ingelmo, Eduardo Vega Perez, Rebecca Pitt, Victor Hugo Gonzalez Cardenas, Nada Mohamed, Susana G. Sotocinal, Jeffrey S. Mogil and Luda Diatchenko. Published with license by Taylor & Francis Group, LLC.

This is an Open Access article distributed under the terms of the Creative Commons Attribution License (http://creativecommons.org/licenses/by/4.0/), which permits unrestricted use, distribution, and reproduction in any medium, provided the original work is properly cited.

**Introduction/Aim**: Mast cell (MC) activation could establish a positive feedback loop that perpetuates inflammation and maintains pain. Stabilizing MCs with ketotifen fumarate (KF) may disrupt this loop and consequently relieve pain. Here, we tested the effect of treatment with KF in assays of inflammatory pain in mice, and in pressure pain thresholds (PPT) of patients with chronic widespread pain (CWP).

**Methods**: Wildtype and MC-deficient (B6-kit^W-sh/W-sh^) mice (n = 12–15/group) were injected with formalin (0.5%) or CFA (50%) and treated with KF (30mg/kg). Pain behavior was measured in the early and late phases of the formalin test. Mechanical sensitivity was tested using von Frey fibers pre-CFA, 3 days post-CFA, and 30min post-KF injection on day 3. Adolescents (10 CWP, 5 controls; ages:15-18y) were treated with KF (6mg/day) or placebo for up to 4 months. Their PPT for the trapezius muscle was measured monthly using an algometer.

**Results**: Treatment with KF significantly reduced pain behavior in the late phase of the formalin test in wildtype but not mutant mice (genotype-drug interaction: *F*_1,48_ = 5.3, p = 0.02). It also significantly reduced mechanical allodynia in a genotype-dependent manner (genotype- drug: *F*_1,36_ = 4.5, p = 0.04). PPT of CWP patients was also significantly reduced after 4 months of treatment with KF (p = 0.02) but not with placebo.

**Discussion/Conclusions**: Our findings indicate that treatment with KF is capable of reducing pain in mice and humans in a mast cell-dependent manner. This suggests that KF, commonly used for the treatment of asthma and allergic conditions, may be useful for the treatment of chronic pain conditions.

## Patients’ opinions and concerns about the opioid crisis in Quebec: preliminary findings

Jean-Luc Kaboré^a^, Gabrielle Pagé^b^, Lise Dassieu^c^, Élise Roy^c^, Jacques Laliberté^d^, Alexandre Parent^e^, Jean-Sébastien Roy^f^, Marc O. Martel^g^ and Manon Choinière^h^

^a^Faculty of Medicine, Department of Pharmacology and Physiology, Centre de Recherche du Centre hospitalier de l’Université de Montréal, Université de Montréal, Montreal, Quebec, Canada; ^b^Faculty of Medicine, Department of Anesthesiology and Pain Medicine, Centre de Recherche du Centre hospitalier de l’Université de Montréal, Université de Montréal, Montreal, Quebec, Canada; ^c^Faculté de Médecine et des Sciences de la Santé, Chaire de recherche en toxicomanie, Université de Sherbrooke, Longueuil, Quebec, Canada; ^d^Association Québécoise de la douleur chronique (AQDC), Patient Partner, Montreal, Quebec, Canada; ^e^Université de Sherbrooke, Quebec Pain Research Network, Sherbrooke, Quebec, Canada; ^f^Faculty of Medicine, Department of Rehabilitation, Centre interdisciplinaire de recherche en réadaptation et en intégration sociale (CIRRIS), Université Laval, Quebec City, Quebec, Canada; ^g^Faculty of Dentistry, Faculty of Medicine, Department of Anesthesia, McGill University, Montreal, Canada; ^h^Faculty of Medicine, Department of Anesthesiology and Pain Medicine, Centre de Recherche du Centre hospitalier de l’Université de Montréal, Université de Montréal, Montreal, Quebec, Canada

**CONTACT** Jean-Luc Kaboré benewende.jean.luc.kabore@umontreal.caCentre de recherche du Centre hospitalier de l'Université de Montréal, Tour Saint-Antoine, 850 rue Saint-Denis, Montréal (Québec), H2X 0A9

© 2018 Jean-Luc Kaboré, Gabrielle Pagé, Lise Dassieu, Élise Roy, Jacques Laliberté, Alexandre Parent, Jean-Sébastien Roy, Marc O. Martel and Manon Choinière. Published with license by Taylor & Francis Group, LLC.

This is an Open Access article distributed under the terms of the Creative Commons Attribution License (http://creativecommons.org/licenses/by/4.0/), which permits unrestricted use, distribution, and reproduction in any medium, provided the original work is properly cited.

**Introduction/Aim**: The opioid crisis in the US and Canada has led to restrictive measures that could negatively impact chronic pain management and reduce patients’ access to treatment. This study aimed to examine patients’ opinions and concerns about the opioid crisis.

**Methods**: An online questionnaire was administered to chronic pain participants throughout the province of Quebec from January to March 2018. Multivariate logistic regressions were used to identify factors associated with patients’ opinions, concerns, and opioid dose decrease.

**Results**: A total of 723 participants with chronic pain completed the questionnaire. The mean age was 50.0 ± 13.9 years, 79.1% were women, and 82.9% had at least a college degree.

Current opioid users were more likely to report scrutiny by others (OR = 1.62 (95% CI: 1.33 – 1.97), p < 0.001) compared to non-current users. Current opioid users who consented to a dose decrease (8.20%, n = 30) were more likely to report fear of addiction (OR = 1.29 (95% CI: 1.01 – 1.66), p = 0.041).

Participants rated chronic pain treatment with scores close to “not acceptable” (score = 3.7 ± 2.5 (0 = not acceptable; 10 = optimal)) and rated with scores close to “distorted” the image of opioid users conveyed by media coverage (score = 7.2 ± 2.2, (0 = good image; 10 = distorted image)).

**Discussion/Conclusions**: Many patients reported that the opioid crisis is associated with scrutiny and with a distorted image of opioid users. Our findings also revealed that some patients might consent to a dose decrease due to fear of addiction. Optimal strategies need to be found to deal with the opioid crisis while maintaining access to treatment.

## Logic analysis: a standardized method of reporting treatment components and testing program theory plausibility in specialized paediatric pain rehabilitation programs

Karen Hurtubise^a^, Astrid Brousselle^b^, Melanie Noel^c^ and Chantal Camden^d^

^a^Faculté de Médecine et Sciences de la Santé, Université de Sherbrooke, Sherbrooke, QC, Canada;; ^b^School of Public Administration, University of Victoria, Public Health, Victoria, BC, Canada; ^c^Department of Psychology, Faculty of Arts, University of Calgary, Calgary, AB, Canada; ^d^Faculté de Médecine et Sciences de la Santé, Univeristé de Sherbrooke, Sherbrooke, QC, Canada

**CONTACT** Karen Hurtubise karen.hurtubise@usherbrooke.ca

© 2018 Karen Hurtubise, Astrid Brousselle, Melanie Noel and Chantal Camden. Published with license by Taylor & Francis Group, LLC.

This is an Open Access article distributed under the terms of the Creative Commons Attribution License (http://creativecommons.org/licenses/by/4.0/), which permits unrestricted use, distribution, and reproduction in any medium, provided the original work is properly cited.

**Introduction/Aim**: Detailed linkages between program components and their intended outcomes (i. e. program theory) have rarely been described in studies involving specialized paediatric pain rehabilitation programs. Logic analysis, a theory-based approach, aims to assess a programs plausibility in reaching expected results, by comparing the program,s logic to existing scientific knowledge. Our aim was to conduct a logic analysis as a preliminary evaluation of our specialized pain rehabilitation program.

**Methods**: A three-step process was used. First, a logic model, a visual representation of the program theory, was constructed by a 13-member expert committee of clinicians, managers, youth with pain related disability, and their parents. An evidence-based framework was then developed by the research team, guided by the following question: “What principles should a self-management program for youth with pain-related disability adopt to promote self-efficacy, and participation in age-appropriate activities?” Lastly, the initial logic model was examined against the framework and recommendations were identified collaboratively by research team and expert panel.

**Results**: Logic model construction using this process helped in 3 ways. First, it raised awareness of clinicians' beliefs of the causal mechanisms between program components and outcomes. Second, the involvement of youth and their parents ensured the inclusion of components and outcomes deemed most useful to service users. Third, it assisted in identifying program characteristics supported by scientific evidence and detecting program gaps highlighted by the literature outside the field of pain (e. g. youth self-management). Although evidence supported many program components, some principles arising from youth self-management, self-efficacy, and peer-coaching literature were lacking.

**Discussion/Conclusions**: Logic analysis proved useful in describing our specialized program, allowed the preliminary testing of its program theory, and recognize component gaps requiring further intervention refinement.

## Ehlers-Danlos syndrome patients’ needs assessment: how to treat or manage their pain is one of their greatest needs

Alishia Poccia^a^, Gail Ouellette^b^, Sandy Smeenk^c^ and Mihai Pascariu^b^

^a^Biotechnology and Genomics, Concordia University, Montreal, Canada; ^b^Regroupement québécois des maladies orphelines, Montreal, Canada; ^c^The ILC Foundation, Oakville, Canada

**CONTACT** Alishia Poccia alishiapoccia@hotmail.ca

© 2018 Alishia Poccia, Gail Ouellette, Sandy Smeenk and Mihai Pascariu. Published with license by Taylor & Francis Group, LLC.

This is an Open Access article distributed under the terms of the Creative Commons Attribution License (http://creativecommons.org/licenses/by/4.0/), which permits unrestricted use, distribution, and reproduction in any medium, provided the original work is properly cited.

**Introduction/Aim**: Ehlers-Danlos syndrome (EDS) is a heritable connective tissue disorder. Recent clinical research has shown that EDS is a complex, variable and multi-systemic disorder affecting many organs and systems. Pain associated with EDS is not only due to musculoskeletal problems, but can also be visceral and neurological. Pain can be of all subtypes: nociceptive, neuropathic and dysfunctional (abdominal, gynecological, headaches of various causes, fibromyalgia, etc.). Due to the complexity of this syndrome and its consequences on the quality of life of patients, we undertook a survey to determine the needs and priorities of this patient population.

**Methods**: An online questionnaire to determine the needs and priorities of this patient population in Canada was made available through our two patient organizations (Regroupement québécois des maladies orphelines and The ILC Foundation).

**Results**: Of the 202 respondents, 49% listed pain as one of their five most limiting symptoms and most challenging aspects of their lives. Pain was the number one issue for 27% of the respondents. Pain also came first as the symptom “the most under-appreciated by my doctor”. Fatigue, lack of awareness/knowledge of the medical community, and lack of help/support were among the other most frequent complaints.

**Discussion/Conclusions**: There is still much lack of awareness and knowledge about Ehlers-Danlos syndrome in the medical community in Canada, especially concerning the presence of pain in various forms. Patients are calling for more knowledge about this aspect of their disease, as well as more resources and support for the treatment and management of their pain.

## The efficacy of oral versus injectable analgesics for the treatment of pain in rodents

Mary Loka^a^, Chulmin Cho^b^, Vassilia Michailidis^c^, Matthew Danesh^d^ and Loren J. Martin^b^

^a^Neuroscience, McGill University, Montreal, Canada; ^b^Psychology, University of Toronto, Toronto, Canada; ^c^Cell and Systems Biology, University of Toronto, Toronto, Canada; ^d^Human Biology, University of Toronto, Toronto, Canada

**CONTACT** Mary Loka mary.loka@mail.mcgill.ca

© 2018 Mary Loka, Chulmin Cho, Vassilia Michailidis, Matthew Danesh and Loren Martin. Published with license by Taylor & Francis Group, LLC.

This is an Open Access article distributed under the terms of the Creative Commons Attribution License (http://creativecommons.org/licenses/by/4.0/), which permits unrestricted use, distribution, and reproduction in any medium, provided the original work is properly cited.

**Introduction/Aim**: Rodents are used in research to answer essential scientific and medical questions. In neuroscience, surgeries such as craniotomies are preformed to answer these questions. As such, research institutions have set mandates regarding the administration of analgesia to laboratory rodents undergoing invasive procedures. These are typically administered via injection, which can induce stress, altering an animal’s behaviour, and limiting the generalizability of the research. Administering medication in an animal’s water may be more effective in alleviating pain since it is a less invasive, and remains readily accessible as opposed to receiving medication all at once through injection.

**Methods**: For this experiment, we compare the efficacy of Carprofen, Meloxicam, Buprenorphine, and saline given both orally and through injection for alleviating craniotomy pain in mice. We use minimally invasive measures of well-being including the Mouse Grimace Scale to assess spontaneous pain.

**Results**: Results have shown that control animals expressed the most pain behavior on surgery day compared to animals receiving analgesics, though injections seem more effective in mitigating the pain response than oral administration. We also show that buprenorphine is the most effective for treating pain in both males and females, though the pain trajectories for other drugs differed between sexes.

**Discussion/Conclusions**: Although it is necessary to provide laboratory animals with analgesics after an invasive procedure, there remain gaps in the literature regarding treatment options for best outcomes in the domains of both animal welfare and efficacious research. This study reveals interesting sex differences in the development of drug mediated pain trajectories which should be further explored.

## Determining whether pain sensitivity contributes to the fear-avoidance model

Zakir Uddin^a^, Arthur Woznowski-Vu^a^, Daniel Flegg^a^, Andrea Aternali^b^, Rebekah Wickens^c^ and Timothy H. Wideman^a^

^a^McGill University, School of Physical and Occupational Therapy, Montreal, Quebec, Canada; ^b^McGill University, Psychology, Montreal, Quebec, Canada; ^c^Constance Lethbridge Rehabilitation Centre/McGill University, School of Physical and Occupational Therapy, Montreal, Quebec, Canada

**CONTACT** Zakir Uddin zakir.uddin@mail.mcgill.ca

© 2018 Zakir Uddin, Arthur Woznowski-Vu, Daniel Flegg, Andrea Aternali, Rebekah Wickens and Timothy H. Wideman. Published with license by Taylor & Francis Group, LLC.

This is an Open Access article distributed under the terms of the Creative Commons Attribution License (http://creativecommons.org/licenses/by/4.0/), which permits unrestricted use, distribution, and reproduction in any medium, provided the original work is properly cited.

**Introduction/Aim**: The Fear-Avoidance Model (FAM) is an empirical cognitive-behavioral model that predicts pain and disability-related outcomes. The FAM suggests that with chronic pain, disability is autonomous from neurophysiological sensory processing factors. Increased pain sensitivity has been shown to be involved in chronicity, but is not specifically addressed within FAM. The purpose of this study is to determine whether pain sensitivity, measured by Quantitative Sensory Testing (QST), contributes unique predictive value within the FAM.

**Methods**: Eighty participants with chronic musculoskeletal pain completed the measures for (1) FAM constructs: pain catastrophizing, pain-related fear, avoidance (physical interference and lift tolerance), pain-related disability, depression and pain severity; (2) QST: pressure pain threshold (PPT) and temporal summation of mechanical pain (TSP). Five multiple regression analyses (dependent variables: physical interference, lift tolerance, pain-related disability, depression and pain severity) were used to determine the predictive capability of QST measures in the FAM while controlling for significant individual characteristics’ covariates and cognitive-behavioral factors (pain catastrophizing and pain-related fear).

**Results**: The results revealed that (I) TSP factor was a predictor of physical interference (β = .252, t = 2.613, p < .01) and pain catastrophizing (p < .01) and pain-related fear (p < .01) contributed to the variance; (II) PPT factor was a predictor of lift tolerance (β = .249, t = 2.281, p < .05) and gender (p < .01) contributed to the variance, whereas TSP factor failed to contribute in the variance (P > .05); (III) PPT factor was a predictor of disability (β = ‒.321, t = ‒3.335, p < .01) and pain catastrophizing (p < .01) and pain-related fear (p < .05) contributed to the variance; (IV) pain catastrophizing significantly predicted depression (β = .626, t = 6.786, p < .01), whereas pain-related fear failed to predict and none of the QST variables were correlated to depression. (V) TSP factor was a predictor of pain severity (β = .248, t =2.576, p < .01), and pain catastrophizing (p < .01) and pain-related fear (p < .05) contributed to the variance.

**Discussion/Conclusions**: Pain sensitivity measures contributed to the FAM by showing additional predictive value in all outcomes, except depression. Contrary to the FAM, this analysis suggests that pain sensitivity contributes novel predictive value within the FAM. Theoretical and clinical implications are discussed.

References1.Vlaeyen
JWS, Linton
SJ.
Fear-avoidance and its consequences in chronic musculoskeletal pain: A state of the art. *Pain*. 2000;85(3):317–332.1078190610.1016/S0304-3959(99)00242-02.Wideman
TH, Asmundson
GGJ, Smeets
RJEM, et al. Rethinking the fear avoidance model: Toward a multidimensional framework of pain-related disability. *Pain*. 2013;154(11):2262–2265.2374811510.1016/j.pain.2013.06.005PMC40789763.Uddin
Z, MacDermid
JC.
Quantitative Sensory Testing in Chronic Musculoskeletal Pain. *Pain Med*. 2016;17(9):1694–1703.2689311610.1093/pm/pnv105

### Perceived injustice and self-efficacy mediate the relation between post-traumatic stress symptoms and adverse outcomes in individuals with whiplash injuries

Esther Yakobov^a^, Pascal Thibault^a^ and Michael JL Sullivan^a^

Psychology, McGill University, Montreal, Canada

**CONTACT** Esther Yakobov esther.yakobov@mail.mcgill.ca

© 2018 Esther Yakobov, Pascal Thibault and Michael JL Sullivan. Published with license by Taylor & Francis Group, LLC.

This is an Open Access article distributed under the terms of the Creative Commons Attribution License (http://creativecommons.org/licenses/by/4.0/), which permits unrestricted use, distribution, and reproduction in any medium, provided the original work is properly cited.

Research indicates that post-traumatic stress symptoms following whiplash contribute to prolonged disability and symptom chronicity. The pathways by which post-traumatic stress symptoms impact on poor outcomes remain understudied. Perceived injustice has been discussed as risk factor for complicated trajectories of recovery in individuals with whiplash injuries. Perceived self-efficacy has been shown to act as a protective factor and improve outcomes in patient with whiplash. To date the influence of perceived injustice and perceived self-efficacy on the relation between post-traumatic symptoms and post injury outcomes has not been systematically investigated.

**Introduction/Aim**: The aim of the present study was to investigate whether reductions in perceptions of injustice and increase in self-efficacy following treatment, mediated the relation between post-traumatic symptoms, disability, and symptom severity in individuals with whiplash injuries.

**Methods**: The study sample consisted of 104 individuals enrolled in a multidisciplinary treatment program aimed at promoting recovery following whiplash injury. Participants completed questionnaires prior to treatment, and immediately following treatment.

**Results**: Reductions in perceived injustice and increase in self-efficacy independently mediated the relation between the decrease in post-traumatic stress symptoms and disability. Increased self-efficacy mediated the relation between reduction in post-traumatic stress symptoms and reductions in pain severity and depressive symptoms.

**Discussion/Conclusions**: The results of the present study suggest that perceived injustice and self-efficacy might act as two distinct pathways by which post-traumatic stress symptoms contribute to disability. Self-efficacy emerged as unique mediator in the relation between reductions in post-traumatic stress symptoms and reductions in pain and depressive symptoms. Clinical and theoretical implications are discussed.

### The effect of intranasal oxytocin on pain and function among women with chronic pelvic pain: a feasibility trial

Michelle Flynn^a^, Tavis Campbell^a^, Magali Robert^b^, Maryam Nasr-Esfahani^b^ and Joshua A. Rash^c^

^a^Department of Psychology, University of Calgary, Calgary, Alberta, Canada; ^b^Obstetrics & Gynaecology, University of Calgary, Calgary, Alberta, Canada; ^c^Department of Psychology, Memorial University of Newfoundland, St. John’s, Newfoundland, Canada

**CONTACT** Joshua A. Rash jarash@mun.ca

© 2018 Michelle Flynn, Tavis Campbell, Magali Robert, Maryam Nasr-Esfahani and Joshua A. Rash. Published with license by Taylor & Francis Group, LLC.

This is an Open Access article distributed under the terms of the Creative Commons Attribution License (http://creativecommons.org/licenses/by/4.0/), which permits unrestricted use, distribution, and reproduction in any medium, provided the original work is properly cited.

**KEYWORDS** chronic pain; oxytocin; feasibility trial

**Introduction/Aim**: Chronic pelvic pain (CPP) affects 5.7%-26.6% of women in the general population and is associated with poor health and social outcomes. The range of empirically-supported treatments is limited. Studies suggest exogenous oxytocin (OT) administration reduces pain sensitivity for chronic back, headache and colon pain patients. This abstract details pragmatic considerations from the first trial investigating OT’s effect on pain and function among women with CPP (TrialRegistration#NCT02888574).

**Methods**: Women with CPP completed baseline measures; were randomized to experimental or control condition involving self-administering twice-daily doses of 24 IU OT or placebo, respectively; underwent a 2-week washout period; and crossed over to receive the condition they had not yet received. Pain and function were recorded in daily diaries, using validated measures.

**Results**: The trial is ongoing, with twelve women recruited and four completed. Women reported moderate pain and pain interference; average pain catastrophizing; high perceived social support; and mild depression, anxiety, and stress symptoms. Interested women needed more education regarding OT’s safety than anticipated. Completing daily diaries was perceived as a large commitment, though adherence was excellent (<1% missing data). Bi-weekly reminder calls were necessary to ensure adherence. No women reported missing doses. Women endorsed that the treatment is highly logical and anticipated experiencing an average 54% pain relief. No serious/unexpected adverse reactions were reported.

**Discussion/Conclusions**: The current study is an innovative trial investigating the effect of OT on pain and function, providing information regarding recruitment and design for a larger trial. Results indicate the treatment is acceptable and feasible.

### Diagnostic uncertainty in youth with chronic pain and their parents: A dyadic examination

Alexandra Neville^a^, Abbie Jordan^b^, Jaimie Beveridge^a^ and Melanie Noel^a^

^a^Department of Psychology, University of Calgary, Calgary, Alberta, Canada; ^b^Department of Psychology, University of Bath, Bath, England

**CONTACT** Alexandra Neville alexandra.neville@ucalgary.ca

© 2018 Alexandra Neville, Abbie Jordan, Jaimie Beveridge and Melanie Noel. Published with license by Taylor & Francis Group, LLC.

This is an Open Access article distributed under the terms of the Creative Commons Attribution License (http://creativecommons.org/licenses/by/4.0/), which permits unrestricted use, distribution, and reproduction in any medium, provided the original work is properly cited.

**Introduction/Aim**: Diagnostic uncertainty (DU), the perception of a lack of or incorrect label to explain symptoms, has been reported by parents of youth with chronic pain. This study provided a qualitative examination of DU in *both* youth with chronic pain and their parents.

**Methods**: Pain narratives were elicited through individual interviews with fifteen youth with idiopathic chronic pain aged 10– 18 years and one of their parents, recruited from a tertiary level pediatric chronic pain program. Interviews focused on exploring participants’ memories and perceptions around diagnosis. Parents and youth also completed 3 dichotomous questions to assess DU that were adapted from the adult chronic pain literature. Narratives were analyzed using thematic analysis.

**Results**: Responses to the dichotomous questions revealed that 9/15 parents and 4/15 children reported DU. When disparities occurred within parent-child dyads (5 dyads), it was parents who expressed uncertainty around the diagnosis. Thematic analysis revealed that, while some individuals “agreed” with the diagnosis, many also believed that something was missing and continued to search for an alternate diagnosis. When individuals expressed DU, this was met with frustration, disempowerment, feeling unheard and alone, and mistrust in the medical system.

**Discussion/Conclusions**: Some youth with chronic pain, and many of their parents, experience DU, which is integrally tied to their experiences with clinicians and the medical system. Greater understanding of DU, between and within parent-child dyads, may help tailor how clinicians deliver diagnoses to families to achieve ‘buy in’, increase understanding of pain and the diagnosis, and ultimately improve treatment response.

### Characterization of the nociceptive properties and cellular mechanism of lionfish venom

Stephanie Mouchbahani-Constance^a^, Stephen Lesperance^b^, Amanda Macpherson^a^, Hugues Petitjean^a^, Albena Davidova^a^, Steven Prescott^b^ and Reza Sharif-Naeini^a^

^a^McGill University, Department of Physiology and Cell Information Systems, Montreal, QC, Canada; ^b^Department of Physiology and the Institute of Biomaterials and Biomedical Engineering, University of Toronto Neurosciences and Mental Health, The Hospital for Sick Children, Toronto, ON, Canada

**CONTACT** Reza Sharif-Naeini reza.sharif@mail.mcgill.ca

© 2018 Stephanie Mouchbahani-Constance, L. Stephen Lesperance, Amanda Macpherson, Hugues Petitjean, Albena Davidova, Steven A. Prescott and Reza Sharif-Naeini. Published with license by Taylor & Francis Group, LLC.

This is an Open Access article distributed under the terms of the Creative Commons Attribution License (http://creativecommons.org/licenses/by/4.0/), which permits unrestricted use, distribution, and reproduction in any medium, provided the original work is properly cited.

**Introduction/Aim**: The lionfish (*Pterois volitans*) is a venomous species of fish that has invaded the Caribbean and Atlantic Coast of the U.S. In addition to decimating local fish populations, the lionfish administers an extremely painful sting that can be debilitating for up to one month in severe cases. There exists no treatment for those affected due to the lack of knowledge of the venom’s algogenic properties and mechanism of action. In this study, we provide the first characterization of the pain and inflammation caused by lionfish venom, and examine its cellular target(s).

**Methods**: We studied the short- and long-term pain resulting from intraplantar injection of the venom in mice with behavioral assays. Inflammation was studied with plasma extravasation assays, and expression levels of neuronal activation markers in the dorsal horn were examined via immunohistochemistry. Calcium imaging and electrophysiology experiments were performed to identify the venom’s cellular mechanism of action.

**Results**: We find that intraplantar injection of the venom causes an increase in pain behavior, which can be eliminated by trypsinizing or boiling it. The venom resulted in a sharp increase in mechanical sensitivity, without increasing thermal sensitivity. We observed local inflammation and increased activation of dorsal horn nociceptive circuits. Calcium imaging and electrophysiology experiments showed that the venom acts specifically on non-peptidergic, TRPV1-negative C-fibers.

**Discussion/Conclusions**: Our results provide the first characterization of the pain elicited by the lionfish venom, as well as the first demonstration that the venom acts preferentially on a specific subset of nociceptors.

### Activating the motor system with therapeutic exercises and transcranial direct current stimulation to relieve pain in elderly

Marie-Philippe Harvey^a^, Eléonor Riesco^a,b^, Kevin Whittingstall^c,d^ and Guillaume Léonard^a,e^

^a^Research Centre on Aging, CIUSSS de l’Estrie-CHUS, Sherbrooke, Québec, Canada; ^b^Faculty of Physical education and sport, Department of Kinanthropology, Université de Sherbrooke, Sherbrooke, Québec, Canada; ^c^Centre de Recherche du CHUS, Université de Sherbrooke, Sherbrooke, Québec, Canada; ^d^Faculty of Medicine and Health Sciences, Department of Nuclear Medecine and Radiobiology, Université de Sherbrooke, Sherbrooke, Québec, Canada; ^e^Faculty of Medicine and Health Sciences, School of rehabilitation, Université de Sherbrooke, Sherbrooke, Québec, Canada

**CONTACT** Marie-Philippe Harvey Marie.philippe.harvey@usherbrooke.ca Université de Sherbrooke, Sherbrooke, Québec, Canada

© 2018 Marie-Philippe Harvey, Eléonor Riesco, Kevin Whittingstall and Guillaume Léonard. Published with license by Taylor & Francis Group, LLC.

This is an Open Access article distributed under the terms of the Creative Commons Attribution License (http://creativecommons.org/licenses/by/4.0/), which permits unrestricted use, distribution, and reproduction in any medium, provided the original work is properly cited.

**Introduction/Aim**: Prevalence and intensity of chronic pain substantially increases with age. Therapeutic exercises have been shown to be effective to reduce chronic pain in elderly. Recently, some researchers have proposed that the addition of transcranial direct current stimulation (tDCS; a non-invasive brain stimulation technique) could potentiate the analgesic effect of exercises. The aim of the present study was to determine whether the analgesic effect of exercises + tDCS differs from the effect of exercises alone and if this effect is different between elderly with low corticospinal projections (CP) and those with strong CP.

**Methods**: Eighty elderly individuals suffering from chronic pain will be recruited in this parallel-group randomised control trial. Participants will be separated according to the strength of their corticospinal projections (evaluated with transcranial magnetic stimulation; Group A = low CP, Group B = strong CP), and will then be randomized to receive exercises combined to real tDCS (2 mA, 20 minutes) or to sham tDCS. Intervention will last 12 weeks (three treatment sessions per week). Pain intensity will be assessed with a logbook containing numerical pain rating scales.

**Results**: We believe that exercises + real tDCS will be more effective than exercises + sham tDCS, but only in individuals with low CP.

**Discussion/Conclusions**: This research project will help to better understand the role played by the motor system in the persistence and relief of chronic pain in elderly. This study could also help determine which individuals will benefit from the addition of tDCS to exercises to decrease pain.

### Sex differences in the contribution of spinal atypical PKCs in the maintenance of centrally-mediated persistent pain

Nicole C. George^a^, Andre Laferriѐre^a^ and Terence J. Coderre^a^

Anesthesia, McGill University, Montreal, Quebec, Canada

**CONTACT** Nicole C. George Nicole.george@mail.mcgill.ca

© 2018 Nicole C. George, Andre Laferriѐre and Terence J. Coderre. Published with license by Taylor & Francis Group, LLC.

This is an Open Access article distributed under the terms of the Creative Commons Attribution License (http://creativecommons.org/licenses/by/4.0/), which permits unrestricted use, distribution, and reproduction in any medium, provided the original work is properly cited.

**Introduction/Aim**: Protein kinase Mζ (PKMζ) has been implicated in the maintenance of hippocampal LTP and memory, as well as spinal nociceptive sensitization. Recent hippocampal studies established a compensatory mechanism by PKCι, though there has been little investigation into PKCι for nociception. Moreover, PKMζ-dependent sex differences suggest that females may rely on mechanisms other than PKMζ, such as PKCι. Thus, the purpose of this study was to examine the contribution of atypical PKCs to the maintenance of pain hypersensitivity in both male and female animals.

**Methods**: The specific PKCι inhibitor ICAP was delivered intrathecally to long Evans hooded rats, in two pain models. Intraplantar injection of formalin (2%) to the hind paw produced an acute biphasic nociceptive response. Two intramuscular (i.m.) injections of acidic saline to the rat thigh, spaced 5 days apart, were used to produce persistent referred allodynia in the hind paw. Formalin nociception was assessed by measuring sustained nociceptive behaviours (SNBs), and paw withdrawal thresholds (PWT) were assessed using von Frey filaments.

**Results**: Nociceptive behaviours (SNBs, PWT) were significantly reduced in males following PKCι inhibition in both the formalin and i.m. acidic saline tests, while no effect was shown in the females for either test.

**Discussion/Conclusions**: These findings demonstrate a role of spinal PKCι in centrally-mediated persistent pain for male rats, and suggest that females may rely on a mechanism independent of atypical PKCs for the maintenance of persistent nociceptive sensitization.

### What happens to intimacy when it hurts to be touched? A mixed methods study in persons with CRPS

Tara Packham^a^, Kaitlyn Wainio^b^ and Ming-Kin Wong^c^

^a^Michael G. DeGroote Institute for Pain Research and Care, McMaster University, Hamilton, Ontario, Canada; ^b^School of Rehabilitation Sciences, McMaster University, Hamilton, Ontario, Canada; ^c^McMaster University, School of Rehabilitation Sciences, Hamilton, Ontario, Canada

**CONTACT** Tara Packham packhamt@mcmaster.ca

© 2018 Tara Packham, Katelyn Wainio and Ming-Kin Wong. Published with license by Taylor & Francis Group, LLC.

This is an Open Access article distributed under the terms of the Creative Commons Attribution License (http://creativecommons.org/licenses/by/4.0/), which permits unrestricted use, distribution, and reproduction in any medium, provided the original work is properly cited.

**Introduction/Aim**: Persons with complex regional pain syndrome (CRPS) often experience allodynia, where non-painful stimuli are perceived as painful. Allodynia is associated with central sensitization and poor prognosis, but the impact on physical function and social relationships has not been clearly reported. This mixed methods study will report both quantitative ratings and themes derived from qualitative analysis of interview data addressing allodynia and the impact of CRPS on intimacy.

**Methods**: This is a secondary analysis of a cognitive debriefing study of a condition-specific patient-reported evaluation for CRPS. 44 persons with CRPS recruited through a Canadian national patient support organization were interviewed about their experiences with CRPS while orally completing the evaluation. This study contrasts ratings for allodynia, relationship, and intimacy items with overall scale and instrument scores, and compiles all qualitative transcript segments addressing allodynia, relationships and intimacy. Thematic content analysis will use an interpretive description framework informed by an a priori review of the literature describing the impact of chronic pain on intimacy.

**Results**: This study analysis is in progress. Interpretive description will generate thematic findings to inform clinical practice: these will be compared and contrasted with the mean scores, and relationships between symptom and social variables.

**Discussion/Conclusions**: There is a dearth of qualitative literature describing the experience of complex regional pain syndrome: further, few studies have investigated functional and quality of life impacts of associated symptoms. This study will lay a foundation for future investigations and open dialogue on pain and intimacy in this population.

### The price of pain

Hocine Slimani^a^, Pierre Rainville^b^ and Mathieu Roy^a^

^a^McGill University, Psychology, Montréal, Québec, Canada; ^b^Université de Montréal, Centre de Recherche de l’Institut Universitaire de Gériatrie de Montréal, Montréal, Québec, Canada;

**CONTACT** Hocine Slimani ho.slimani@gmail.com

© 2018 Hocine Slimani, Pierre Rainville and Mathieu Roy. Published with license by Taylor & Francis Group, LLC.

This is an Open Access article distributed under the terms of the Creative Commons Attribution License (http://creativecommons.org/licenses/by/4.0/), which permits unrestricted use, distribution, and reproduction in any medium, provided the original work is properly cited.

**Introduction/Aim**: In order to make optimal decisions between goods of different nature, instrumental decision-making systems must base their choices on an abstract quantity: *value*. In the present study, we aimed at determining the monetary value of pain in order to gain insight on how it influences reward seeking.

**Methods**: 3 groups of 30 healthy volunteers filled out questionnaires assessing various personality traits, before undergoing a pain sensitivity assessment using electric shocks delivered to the ankle. Thereafter, participants completed a decision-making task during which they had to accept or decline offers that included pairs of varying levels of pain (threshold to tolerance) and monetary compensations. While the 16 monetary offers ranged linearly from 0 to 5$ or 10$ in Group1 and 2, respectively, they increased exponentially from 0 to 5$ in Group3.

**Results**: Our data show that the monetary value of pain increased quadratically as a function of stimulus intensity (t = 5.04, p < 0.001). Whereas doubling the monetary offers had no significant influence on the pain value (Group2–Group1), changing their distribution (Group3–Group1) decreased it (t = 1.52, p = 0.045). The psychometric data showed that harm avoidant personalities predict an increased pain valuation, whereas goal-directed mindsets are predictors of a devaluation of pain.

**Discussion/Conclusions**: Our findings indicate that similar increases in perceived pain intensity yield greater gains in value when approaching pain tolerance. We further show that the experimental manipulation of the distribution, rather than the range, of the monetary rewards influences pain valuation. Finally, we show that the pain valuation can be predicted by psychometric measures.

### Grappling with uncertainty: a quantitative examination of diagnostic uncertainty in youth with chronic pain and their parents

Tatiana Lund^a^, Jaimie Beveridge^a^ and Melanie Noel^a^

University of Calgary, Psychology, Calgary, Alberta, Canada

**CONTACT** Tatiana Lund tclund@ucalgary.ca

© 2018 Tatiana Lund, Jaimie Beveridge and Melanie Noel. Published with license by Taylor & Francis Group, LLC.

This is an Open Access article distributed under the terms of the Creative Commons Attribution License (http://creativecommons.org/licenses/by/4.0/), which permits unrestricted use, distribution, and reproduction in any medium, provided the original work is properly cited.

**Introduction/Aim**: Previous qualitative research revealed that diagnostic uncertainty (DU), the perception that a label/explanation for an illness is missing or incorrect, is common and distressing among parents of youth with chronic pain. Nevertheless, research on the impact of DU in youth with chronic pain and their parents is largely lacking. The current study was the first quantitative study to examine DU and its relationship with mental health outcomes in a pediatric chronic pain sample.

**Methods**: Youth aged 10– 18 years (69.2% female) with chronic pain (headache, abdominal, complex) and one of their parents participated in an ongoing study (current N = 39). Youth and parents completed measures of catastrophic thinking about pain, internalizing mental health symptoms, and DU (based on adult literature; Serbic & Pincus, 2013).

**Results**: Results revealed that the majority of youth and parents reported never receiving a clear label/diagnosis (55%, 57%, respectively) or explanation for why they/their child had chronic pain (81%, 60%, respectively). 33% of youth and 40% of parents reported believing that another more serious reason for their pain had gone undetected. Greater parental DU was significantly related to greater child and parental catastrophic thinking about pain and higher parental depressive and PTSD symptoms (*ps* < .05).

**Discussion/Conclusions**: The majority of youth with chronic pain and their parents endorse DU, and this is linked to greater distress. Longitudinal research is needed to determine the directionality of these relationships and how to target DU in clinical encounters (e. g., clearer communication, distress reduction) to enhance treatment outcomes.

### Altered hippocampal subfield volume in patients with idiopathic trigeminal Neuralgia

Michael Vaculik^a^, Peter Shih-Ping Hung^b^ and Mojgan Hodaie^c^

^a^Faculty of Medicine, Dalhousie Medical School, Halifax, Nova Scotia, Canada; ^b^Institute of Medical Science, University of Toronto, Toronto, Ontario, Canada; ^c^Division of Neurosurgery, Toronto Western Hospital and Department of Surgery, University of Toronto, Toronto, Ontario

**CONTACT** Mojgan Hodaie mojgan.hodaie@uhn.ca

© 2018 Michael Vaculik, Peter Shih-Ping Hung and Mojgan Hodaie. Published with license by Taylor & Francis Group, LLC.

This is an Open Access article distributed under the terms of the Creative Commons Attribution License (http://creativecommons.org/licenses/by/4.0/), which permits unrestricted use, distribution, and reproduction in any medium, provided the original work is properly cited.

**Introduction/Aim**: Idiopathic Trigeminal Neuralgia (TN) is a neuropathic pain syndrome characterized by paroxysmal unilateral electric shock-like pains that are limited to one or more divisions of the trigeminal nerve. There are multiple theories regarding TN etiology and pathophysiology, including neurovascular compression (NVC) of the trigeminal nerve at its root entry zone. Alternatively, cases of idiopathic TN that are not explained by NVC suggest an underlying central nervous system (CNS) etiology. In agreement, other chronic pain disorders demonstrate abnormalities in CNS volume and microstructure in regions associated with pain perception and modulation, including the hippocampus. Currently, whether hippocampal subfields are altered in idiopathic TN patients is unknown. We address this question using voxel based morphometry and FreeSurfer 6.0 to analyze hippocampal subfield volume in patients with right-sided idiopathic TN.

**Methods**: We obtained T1-weighted 1x1x1 mm^3^ 3D FSPGR MRI axial images of twenty-three right-sided TN patients and matched healthy controls. Automated segmentation of hippocampal subfields was performed with FreeSurfer v6.0 and the HippocampalSubfields parcellation protocol. Statistical analysis was performed by one-way ANOVA with Geisser-Greenhouse’s correction and Bonferroni’s post-hoc analysis.

**Results**: Right-sided TN patients had a reduction in volume of the right CA1, CA4, Granule Cell Layer, Molecular Layer, and hippocampus-amygdala transition area, resulting in a decreased whole right hippocampal volume, compared to healthy controls.

**Discussion/Conclusions**: Our results suggest that specific hippocampal subfields may play a role in pain perception and modulation in TN. Ongoing investigations, including correlating clinical pain measures with changes in subfield volume, will help to elucidate the role of the hippocampus in TN.

### Recruitment of dorsal horn ascending pathways by calretinin neurons

Hugues Petitjean^a^, Farin Bourojeni^b^, Deborah Tsao^a^, Susana Sotocinal^c^, Jeffrey Mogil^c^, Artur Kania^b^ and Reza Sharif-Naeini^a^

^a^Department Physiology, McGill University, Montreal, Quebec, Canada; ^b^Institut de Recherches Cliniques de Montréal, Montréal, Québec, Canada; ^c^Department Phychology, McGill University, Montreal, Quebec, Canada

**CONTACT** Hugues Petitjean hugues.petitjean@mcgill.ca

© 2018 Hugues Petitjean, Farin Bourojeni, Deborah Tsao, Susana Sotocinal, Jeffrey Mogil, Artur Kania and Reza Sharif-Naeini. Published with license by Taylor & Francis Group, LLC.

This is an Open Access article distributed under the terms of the Creative Commons Attribution License (http://creativecommons.org/licenses/by/4.0/), which permits unrestricted use, distribution, and reproduction in any medium, provided the original work is properly cited.

**Introduction/Aim**: The dorsal horn of the spinal cord is the first relay center of sensory information from the periphery. In lamina II, nociceptive information is processed by a complex network of excitatory and inhibitory interneurons whose function and wiring remain poorly understood. Calretinin-expressing interneurons represent a subset of dorsal horn neuron lamina II located in the termination zone of the central endings of nociceptive fibers, yet their role in the processing of noxious inputs is not fully understood. In this study, we used a multi-disciplinary approach to characterize the role of these neurons in awake, freely moving mice.

**Methods**: Here, we used a combination of transgenic animals, neuronal tracing, and chemo- and optogenetic tools to examine the role of calretinin neurons in the processing of sensory information and characterize their position within the complex circuitry of the dorsal horn.

**Results**: Our data reveal that calretinin neurons are located in lamina II, where they receive appositions from the central endings of different nociceptive fibers (IB4+ and CGRP).

Activation of calretinin neurons by chemogenetic and optogenetic stimulation produces intense nociceptive behaviors demonstrating that calretinin neurons are a central part of the dorsal horn nociceptive circuits.

Furthermore, calretinin neurons form appositions on parabrachial-targeting projection neurons in lamina I, suggesting they could contribute to the modulation of one of the major ascending pain pathways from the dorsal horn.

**Discussion/Conclusions**: Taken together our data demonstrated that calretinin neurons are part of neuronal dorsal horn circuit that can process nociceptive mechanical information and initiates intense nociceptive behaviors. Calretinin neurons act with a pivotal role in this circuit by transmitting nociceptive information directly, monosynaptically, to parabrachial-targeting projection neurons in lamina I.

### Moving from pediatric to adult chronic pain care in Ontario: a period of transition

Justina Marianayagam^a^, Sharleen Friedman^b^, Jennifer Tyrrell^b^, Sarah Sheffe^c^, Tania Di Renna^d^ and Fiona Campbell^e^

^a^The Hospital for Sick Children, Faculty of Health Sciences, University of Ottawa, Ottawa, Canada; ^b^Anesthesia and Pain Medicine, The Hospital for Sick Children, Toronto, Canada; ^c^Toronto Academic Pain Medicine Institute, Women’s College Hospital, Toronto, Canada; ^d^Anesthesiology, University Health Network, Toronto, Canada; ^e^The Hospital for Sick Children, Anesthesia and Pain Medicine, University of Toronto, Toronto, Canada

**CONTACT** Justina Marianayagam justina.marianayagam@gmail.com

© 2018 Justina Marianayagam, Sharleen Friedman, Jennifer Tyrrell, Sarah Sheffe, Tania Di Renna and Fiona Campbell. Published with license by Taylor & Francis Group, LLC.

This is an Open Access article distributed under the terms of the Creative Commons Attribution License (http://creativecommons.org/licenses/by/4.0/), which permits unrestricted use, distribution, and reproduction in any medium, provided the original work is properly cited.

**Introduction/Aim**: In 2013, in order to increase access to services, the Ontario Ministry of Health and Long Term Care partially funded 12 adult and four pediatric hospitals to either create an outpatient chronic pain clinic or enhance existing clinical services. The initial focus was on standardizing a model of care. As an important next step, the purpose of this study was to identify opportunities to improve the transition from pediatric to adult chronic pain care.

**Methods**: An informal email survey was sent out to all chronic pain programs regarding current processes, strengths, gaps, and needs for tools and resources to make the transition into adult care easier. A patient perspective was included in this assessment.

**Results**: Providers in both the pediatric and adult settings identified a large gap in transition care. While all health care institutions have developed informal processes, no formal processes are in place. Most pediatric institutions transition patients on a case-by-case basis. Adult clinics show difficulty in maintaining similar treatment plans for incoming pediatric patients due to high demand and limited resources. The most vulnerable patient population was identified as between 16– 25 years of age, in terms of limited specialized care.

**Discussion/Conclusions**: There is a clear need to formalize transition from pediatric to adult chronic pain care in Ontario. It is recommended that pediatric and adult hospitals collaborate to develop targeted evidence-based strategies to identify appropriate handovers, establish specific treatment programming, tools to identify patient readiness and compliance and to ensure sustainability for young adult  patients (16–25 years). Patients and caregivers should also be engaged consistently during the transition process development to ensure patient and family centered care.

### A meta-analysis of chronic pain studies investigating grey matter alterations in the medial temporal lobe

Lizbeth J. Ayoub^a^, Mary Pat McAndrews^b^ and Massieh Moayedi^c^

^a^Faculty of Dentistry, Krembil Research Institute, University of Toronto, Toronto, Ontario, Canada; ^b^Systems Neuroscience Division, Faculty of Psychology, Krembil Research Institute, University of Toronto, Toronto, Ontario, Canada; ^c^Faculty of Dentistry, University of Toronto, Toronto, Ontario, Canada

**CONTACT** Lizbeth J. Ayoub lizbeth.ayoub@utoronto.ca

© 2018 Lizbeth J. Ayoub, Mary Pat McAndrews and Massieh Moayedi. Published with license by Taylor & Francis Group, LLC.

This is an Open Access article distributed under the terms of the Creative Commons Attribution License (http://creativecommons.org/licenses/by/4.0/), which permits unrestricted use, distribution, and reproduction in any medium, provided the original work is properly cited.

Chronic pain affects 19% of the adult population in Canada. An increasing number of pain neuroimaging studies report structural alterations in the central nervous system of chronic pain patients. Structures of the medial temporal lobe (MTL), notably the hippocampus and parahippocampal gyrus, are reported in some, but not all chronic pain conditions and show grey matter volume (GMV) increases or decreases. The hippocampus in particular is involved in memory, and recent evidence has implicated its structure in the transition from subacute to chronic pain. However, the MTL’s role in chronic pain remains to be elucidated. Here, we conducted the first coordinate-based meta-analysis of chronic pain studies reporting GMV alterations to identify which regions within the MTL show consistent abnormalities in patients compared to healthy controls. Furthermore, we aimed to determine whether these regions show increases or decreases in GMV. Our meta-analysis followed PRISMA guidelines. We selected peer-reviewed articles according to our search criteria and used GingerALE v2.3.6 to create spatial maps of anatomical likelihood estimation across studies at a cluster-level corrected threshold *p* < 0.05. From our article search, we identified 17 structural neuroimaging articles reporting GMV abnormalities in the MTL, which met our criteria. Our meta-analytic results yielded consistent GMV increases in bilateral hippocampi and parahippocampal gyri in chronic pain patients compared to healthy controls (cluster-corrected at *p* < 0.05). Interestingly, although some previous studies have reported GMV decrease, our finding could generate new groundwork for future mechanistic studies investigating the role of the MTL in chronic pain.

**Introduction/Aim**: Chronic pain affects 19% of the adult population in Canada. An increasing number of pain neuroimaging studies report structural alterations in the central nervous system of chronic pain patients. Structures of the medial temporal lobe (MTL), notably the hippocampus and parahippocampal gyrus, are reported in some, but not all chronic pain conditions and show grey matter volume (GMV) increases or decreases. The hippocampus in particular is involved in memory, and recent evidence has implicated its structure in the transition from subacute to chronic pain. However, the MTL’s role in chronic pain remains to be elucidated. Here, we conducted the first coordinate-based meta-analysis of chronic pain studies reporting GMV alterations to identify which regions within the MTL show consistent abnormalities in patients compared to healthy controls. Furthermore, we aimed to determine whether these regions show increases or decreases in GMV.

**Methods**: Our meta-analysis followed PRISMA guidelines. We selected peer-reviewed articles according to our search criteria and used GingerALE v2.3.6 to create spatial maps of anatomical likelihood estimation across studies at a cluster-level corrected threshold *p* < 0.05.

**Results**: From our article search, we identified 17 structural neuroimaging articles reporting GMV abnormalities in the MTL, which met our criteria. Our meta-analytic results yielded consistent GMV increases in bilateral hippocampi and parahippocampal gyri in chronic pain patients compared to healthy controls (cluster-corrected at *p* < 0.05). **Discussion/Conclusions**: Interestingly, although some previous studies have reported GMV decrease, our finding could generate new groundwork for future mechanistic studies investigating the role of the MTL in chronic pain.

### High-fidelity usability testing of novel systems to support pain management and recovery following cardiac surgery in Canada and the United Kingdom

Carley Ouellette^a^, Shaunattonie Henry^a^, Marissa Bird^a^, Sandra L. Carroll^a^, Jennifer Yost^b^ and Michael McGillion^a^

^a^School of Nursing, McMaster University, Hamilton, Ontario, Canada; ^b^Villanova University, Philadelphia, Pennsylvania, United States of America

**CONTACT** Carley Ouellette ouellc1@mcmaster.ca School of Nursing, McMaster University, Hamilton, Ontario, Canada

© 2018 Carley Ouellette, Shaunattonie Henry, Marissa Bird, Sandra L. Carroll, Jennifer Yost, and Michael McGillion. Published with license by Taylor & Francis Group, LLC.

This is an Open Access article distributed under the terms of the Creative Commons Attribution License (http://creativecommons.org/licenses/by/4.0/), which permits unrestricted use, distribution, and reproduction in any medium, provided the original work is properly cited.

**Aim**: The purpose of this study was to user test an eHealth intervention, SMArTVIEW, which combines in-hospital and post-discharge remote automated monitoring, education, and self-management training to optimize pain and related recovery outcomes following cardiac surgery in Canada (CA) and the United Kingdom (UK).

**Methods**: Patients and clinicians engaged in high fidelity usability testing. Participants were trained on how to use SMArTVIEW assessment and monitoring systems and engage in use case workflows with the equipment in the clinical environment. Hypothetical data (e. g. pain intensity scores) were ‘pushed’ into the devices to ensure a risk-free yet realistic experience. Participants were video recorded and asked to think aloud while completing required tasks and while being rated on user performance. Feedback was solicited about the user experience and participants rated their perceived importance of the software interfaces for supporting recovery (range: 0- unimportant, 10- most important)

**Results**: 37 participants (11 patients, 26 nurses) completed user testing. Data indicate that the patient interfaces were well received, with a mean (M) score of 8.8/10, indicating a high degree of perceived importance. Adequate time to learn the interfaces was stressed to ensure user comfort. Nurse interfaces were also well received (M = 8.4/10), being rated as easy to use overall. The need to acclimatize patients to the use of enhanced, continual surveillance of pain and related outcomes was emphasized.

**Conclusion**: SMArTVIEW technologies were found acceptable to patients and clinicians; results have been used to optimize workflows in an international trial (n = 600) which commenced in December 2017.

### Can acetaminophen increase conditioned pain modulation effectiveness?

Yesmine Krid^a^, Guillaume Léonard^a,b^, Philippe Chalaye^a^ and Serge Marchand^a^

^a^Department of surgery, Faculty of Medicine and Health Sciences, Université de Sherbrooke, Quebec, Canada; ^b^Research Center on Aging, Institute of Geriatrics of Sherbrooke, Sherbrooke, Quebec, Canada

**CONTACT** Yesmine Krid yesmine.krid@usherbrooke.ca Université de Sherbrooke, Département de chirurgie, Sherbrooke, Quebec, Canada

© 2018 Yesmine Krid, Philippe Chalaye, Guillaume Léonard and Serge Marchand. Published with license by Taylor & Francis Group, LLC.

This is an Open Access article distributed under the terms of the Creative Commons Attribution License (http://creativecommons.org/licenses/by/4.0/), which permits unrestricted use, distribution, and reproduction in any medium, provided the original work is properly cited.

**Introduction/Aim**: Acetaminophen is one of the most used analgesic. It’s mechanism of action is still unclear, but the descending serotonergic pathways seems to be involved. These pathways are part of the descending pain inhibitory mechanism. Conditioned pain modulation (CPM) is a commonly used experimental protocol (thermode-cold pressor test-CPT-thermode) to evaluate the effectiveness of the endogenous descending pain inhibitory mechanism in humans. The aim of this study is to explore if acetaminophen reinforces the CPM.

**Methods**: In this double-blind randomized controlled-trial with cross-over design, 30 healthy volunteers were included and took 1g oral acetaminophen or placebo. Heat pain stimulations (thermode, left forarm,2-min) were performed before and after the application of a conditioning stimulus (CPT, 2min, 10°C), also before the intake of medication (2^nd^ and 3^rd^ session). The difference in pain intensity induced by the heat pain stimulations before and after the CPT was used to evaluate CPM effectiveness.

**Results**: Pain intensity during CPT was comparable in all 3 experimental sessions (*P*=0.5). Pain induced by the heat pain stimuli decreased after CPT during the three sessions (all *Ps*<0.05). CPM effectiveness was similar during all three sessions and failed to reach significant difference (*P*=0.08). Pain intensity before and 45 minutes after the medication (acetaminophen/placebo) was comparable (*Ps*>0.05).

**Discussion/Conclusions**: - Our results suggest that descending pain inhibition effectiveness is not affected by acetaminophen in healthy volunteers. The analgesic effect of acetaminophen does not seem to be related to descending serotoninergic pathways. As acetaminophen is widely used, more investigation about its mechanism of action is still required.

### Brief physical tasks evoke changes in pain intensity and pain threshold for people with low back pain

Arthur Woznowski-Vu^a^, Zakir Uddin^a^, Daniel Flegg^a^, Rebekah Wickens^b^, Andrea Aternali^c^ and Timothy H. Wideman^a^

^a^School of Physical and Occupational Therapy, McGill University, Montreal, Quebec, Canada; ^b^School of Physical and Occupational Therapy, Constance Lethbridge Rehabilitation Centre/McGill University, Montreal, Quebec, Canada; ^c^Psychology, McGill University, Montreal, Quebec, Canada

**CONTACT** Arthur Woznowski-Vu arthur.woznowskivu@gmail.com McGill University, School of Physical and Occupational Therapy, Montreal, Quebec, Canada

© 2018 Arthur Woznowski-Vu, Zakir Uddin, Daniel Flegg, Rebekah Wickens, Andrea Aternali and Timothy H. Wideman. Published with license by Taylor & Francis Group, LLC.

This is an Open Access article distributed under the terms of the Creative Commons Attribution License (http://creativecommons.org/licenses/by/4.0/), which permits unrestricted use, distribution, and reproduction in any medium, provided the original work is properly cited.

**Introduction/Aim**: Standardized measures of sensitivity to physical activity (SPA) have emerged in recent literature. These SPA measures, however, have mainly focused on pain intensity changes, but not on changes in pressure pain threshold (PPT) or mechanical temporal summation of pain (TSP). The aim of this study is to estimate the extent to which pain intensity, PPT, and TSP change in response to brief physical tasks, among people with low back pain.

**Methods**: Preliminary analysis of 28 participants, characterized by low back pain lasting no more than six months. Testing procedure included two pain-provoking brief physical tasks: 6-minute walking task and 10-repetition lifting task. During the tasks, participants rated their initial and peak pain intensity (0–100). Before and after each task, PPT and TSP measures were recorded (lower back and hands).

**Results**: Wilcoxon signed-rank test found significantly greater pain was evoked from ten repeated lifts than from a single corresponding lift, z = 3.635, p < .0005. The walking task, however, did not provoke a significant increase in pain intensity on paired-samples t-test, t(27) = 1.561, p = .130. Wilcoxon signed-rank test found right hand PPT and left lower back PPT to significantly decrease from pre-to-post walking task (respectively z = −3.211, p = .001 and z = −1.970, p = .049), but no other PPT measure showed significant change. TSP remained stable throughout the brief physical tasks.

**Discussion/Conclusions**: Pain intensity and PPT changed in response to brief physical tasks, but TSP did not. These findings may inform future research aimed at determining the best approach to SPA measurement.

### Characteristics and global impression of change of patients admitted to a chronic pain clinic in southeastern Ontario: an observational study

Etienne J. Bisson^a,c^, Elizabeth Brown^a^, Mary Anne Good^a^, Kyle Vader^a^, Nader Ghasemlou^b,c,h^, Ian Gilron^b,c,h^, Ron Levy^d^, Jordan Miller^e^, Dean A. Tripp^f^, Rosemary Wilson^c,g^, Elizabeth VanDenKerkhof^g^ and Scott Duggan^a^

^a^Chronic Pain Clinic, Kingston Health Sciences Centre-Hotel Dieu Hospital site, Kingston, Ontario, Canada; ^b^Department of Biomedical and Molecular Sciences, Queen’s University, Kingston, Ontario, Canada; ^c^Department of Anesthesiology and Perioperative, Queen’s University, Kingston, Ontario, Canada; ^d^Department of Surgery, Queen’s University, Kingston, Ontario, Canada; ^e^School of Rehabilitation Therapy, Queen’s University, Kingston, Ontario, Canada; ^f^Department of Psychology, Queen’s University, Kingston, Ontario, Canada; ^g^School of Nursing, Queen’s University, Kingston, Ontario, Canada; ^h^Centre for Neuroscience Studies, Queen's University, Kingston, Ontario, Canada

**CONTACT** Etienne J. Bisson etienne.bisson@kingstonhsc.ca

© 2018 Etienne J. Bisson, Elizabeth Brown, Mary Anne Good, Kyle Vader, Nader Ghasemlou, Ian Gilron, Ron Levy, Jordan Miller, Dean A. Tripp, Rosemary Wilson, Elizabeth VanDenKerkhof and Scott Duggan. Published with license by Taylor & Francis Group, LLC.

This is an Open Access article distributed under the terms of the Creative Commons Attribution License (http://creativecommons.org/licenses/by/4.0/), which permits unrestricted use, distribution, and reproduction in any medium, provided the original work is properly cited.

**Introduction/Aim**: This analysis presents characteristics and global impression of change (PGIC) of patients receiving care at the Chronic Pain Clinic, Kingston Health Sciences Centre; and identifies associations between clinical changes and PGIC.

**Methods**: Recruited patients completed a pain surveillance questionnaire at initial and follow-up visits to the clinic which included sociodemographics, medical history, and measures reflecting the biopsychosocial nature of pain. Descriptive statistics were performed on initial and main follow-up measures. Using logistic regression, we examined associations between PGIC and improvement in pain severity, pain interference, and physical and emotional functioning (SF12v2 physical and mental composite score, PCS, MSC) at first follow-up.

**Results**: Between September 2013 and March 2017, 779 patients had complete data on 2279 visits. Patient characteristics were: 51 ± 16 (mean±SD) years of age, 61% female, 51% on opioids, and 41% on at least 3 pain medications. At initial visit, pain severity was moderate (55.7 ± 24.7/100), pain interference was moderate (6.2 ± 2.2/10), PCS was 2 SD below norms (31.7 ± 9.9), and MCS was 1 SD below norms (39.8 ± 11.9). Most common pain locations were lower limb (58%), lower back (55%), and neck and shoulder (54%); 60% had evidence of neuropathic pain. At first follow-up, 46% of patients reported some change, 26% reported moderate change or better, and PGIC was associated with improvement in pain severity, PCS, and MCS.

**Discussion/Conclusions**: Patient characteristics are comparable to other larger pain registries. Improvements in pain severity, physical and emotional functioning are independently associated with PGIC, and expectation is further improved PGIC with interdisciplinary care implementation.

### Automatic pain level classification with physiological signals using machine learning

Weina Jin^a^, Diane Gromala^a^, Junbo Bao^b^, Yabin Guo^b^, Tianpei Shen^b^ and Oliver Schulte^b^

^a^School of Interactive Arts and Technology, School of Computing Science, Simon Fraser University, Surrey, British Columbia, Canada; ^b^Simon Fraser University, School of Computing Science, Burnaby, British Columbia, Canada

**CONTACT** Weina Jin weinaj@sfu.ca

Color versions of one or more of the figures in the article can be found online at www.tandfonline.com/ucjp.

© 2018 Weina Jin, Diane Gromala, Junbo Bao, Yabin Guo, Tianpei Shen and Oliver Schulte. Published with license by Taylor & Francis Group, LLC.

This is an Open Access article distributed under the terms of the Creative Commons Attribution License (http://creativecommons.org/licenses/by/4.0/), which permits unrestricted use, distribution, and reproduction in any medium, provided the original work is properly cited.

Automatic Pain Level Classification with Physiological Signals using Machine Learning

Although patients’ self-reports are recommended to be the primary measure of pain, objective pain assessment with physiological signals has the potential to augment self-reports in recognizing pain for patients who have language barriers or cognitive impairment. In this study, we built machine learning models for pain level recognition with physiological signals from 5 channels: Electrocardiograph (ECG), Galvanic Skin Response (GSR), and Electromyography (EMG) at corrugator, zygomaticus and trapezius muscles. With raw data readings from the biosensors, we experimented with end-to-end Recurrent Neural Network (RNN) models and reached 70.05% accuracy on pain level 0 (no pain) against level 4 (highest level of pain, i. e.: pain tolerance) discrimination with two-layer neural network of Gated Recurrent Units (GRU) cells.

**Introduction/Aim**: Accurate pain assessment is crucial to timely and proper pain management. Although the current pain assessment methods mainly rely on subjective self-report scale, for patients with language barriers or cognitive impairment, traditional pain assessment tools such as behavioral or functional assessment methods are laborious and often requires special training. Recent research that attempt to develop objective pain assessment includes inferring pain level from facial expressions and/or physiological signals. When suffering from pain, the state of the autonomic nervous system changes and the peripheral signals can be read from biosensor measures such as Electromyography (EMG), Electrocardiography (ECG) and Galvanic Skin Response (GSR). Once solid inferences from these data are validated, the peripheral physiological signals then can be fed into a machine learning model to estimate the pain level. With the popularity of wearable technology, inferring pain from physiological signals has the potential to become an adjuvant objective pain assessment tool for those who are suffering from pain in silence. The aim of this study is to explore machine learning in pain level recognition with physiological signals. We built 6 different machine learning models for pain level recognition and compared them.

**Methods**: We used the BioVid Heat Pain Database generated by Walter et al.. 2012 ^1^ The data was collected from 86 healthy participants and consists of 8600 samples. In the experiment, various pain levels (from 0 to 4, 0 is no pain, 1 is pain threshold, 4 is pain tolerance, 2 and 3 are levels between 0 and 4) were elicited by a thermode on the right arm. During the pain window period of 5.5 seconds, 5 channels of physiological signals were recorded: Electrocardiography (ECG), Galvanic Skin Response (GSR), and Electromyography (EMG) at 3 muscles (corrugator, zygomaticus and trapezius muscles). A detailed description of how this dataset was collected is in. ^1^

**Figure 1.**
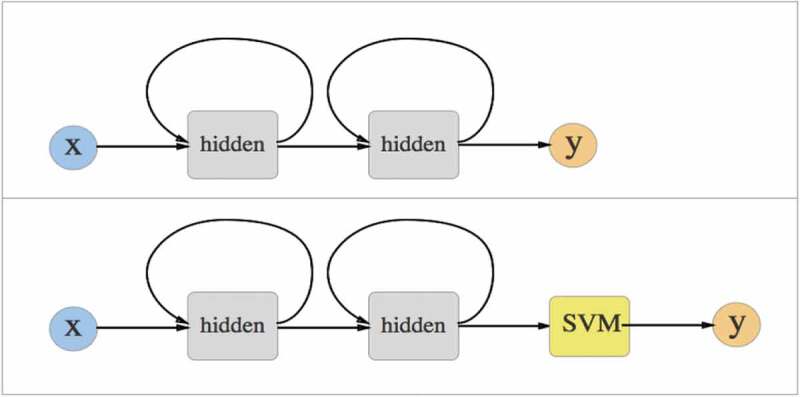
Model architecture of RNN (top) and RNN + SVM (bottom).

We built many-to-one RNN models for sequence classification. To increase the discriminating capacity, the RNN models have two layers, with 128 nodes in each layer (Figure 1 top). We experimented with different RNN cell settings in each layer: (1) long short-term memory (LSTM) cells in the two layers; (2) gated recurrent units (GRU) cells in the two layers; (3) GRU cells in the first layer, and LSTM cells in the second layer.

In addition to the generic RNN models, we also build hybrid RNN+SVM (Support Vector Machine) models for each of the above settings. To increase the discriminating capacity, we replaced the last layer of fully-connected cells with the SVM classifier. In concrete terms, we trained the RNN model first. Next, we fed each training sample into the trained RNN model and calculated the second RNN layer as feature vectors. We used the RNN models to extract features automatically. Instead of feeding the last fully-connected layers with logistic regression, the extracted feature vectors were fed into SVM as training data (Figure 1 bottom).

The RNN model is implemented with Keras 2.1.0, and the SVM classification is built with sklearn library.

**Results**: As shown in Table 1, the highest classification accuracy (70.05%) for recognizing 0 (no pain) against 4 (highest level of pain, i. e.: pain tolerance) is the generic RNN model with two layers of GRU cells. Since the GRU cell has fewer number of parameters than LSTM, for this relatively small dataset, it reached better results than LSTM models. Compared to the generic RNN models, the hybrid RNN+SVM models showed some trends in slightly increasing the accuracy, but the general improvement is not prominent.

**Table 1.** Pain classification accuracy percentage (%) with RNN models of different cell settings. The numbers 0– 4 denote pain level 0– 4 (0: no pain, 1: pain threshold, 4: pain tolerance; 2 and 3 are levels between 0 and 4). GRU: gated recurrent units cell; LSTM: long short-term memory cell; SVM: support vector machine.

	GRU	GRU + SVM	LSTM	LSTM + SVM	GRU/LSTM	GRU/LSTM + SVM
0 vs. 1 vs. 2 vs. 3 vs. 4	24.3	26.75	25.46	25.82	24.19	24.81
0 vs. 1 vs. 4	44.18	44.24	47.48	43.5	48.45	43.21
0 vs. 1	49.41	53.03	46.8	50.32	48.25	50.17
0 vs. 2	49.12	51.77	45.34	50.44	49.42	50.12
0 vs. 3	54.06	55.73	56.39	55.18	51.16	54.22
0 vs. 4	70.05	66.91	61.63	64.09	66.27	65.49

**Discussion/Conclusions**: We experimented with 6 different models to automatically recognize pain levels inferred from physiological signals. To save the manual feature engineering, we also built RNN models with the raw sequential data, and reached 70.05% accuracy on discriminating pain level 0 (no pain) against level 4 (pain tolerance) with a two-layer neural network of GRU cells. Limited by the small numbers that comprise the dataset, however, the end-to-end RNN model did not reach satisfying accuracy. Compared with generic RNN, the hybrid RNN+SVM architecture did not significantly improve the performance for sequential classification. Nevertheless, the 70.05% accuracy suggests that repeating this approach using a much larger dataset may reveal the potential for a machine learning approach.

References1.Walter
S, Gruss
S, Ehleiter
H, Tan
J, Traue
HC, Crawcour
S, Werner
P, Al-Hamadi
A, Andrade
AO (2013). The biovid heat pain database data for the advancement and systematic validation of an automated pain recognition system. In 2013 IEEE International Conference on Cybernetics (CYBCO), pp. 128–31.

### Development of the chronic pain integration questionnaire

Research poster abstract

Kathryn Deshaies^a^, Sharon Kaasalainen^b^ and Noori Akhtar-Danesh^b^

^a^School of Nursing, St. Clair College, Windsor, ON, Canada; ^b^School of Nursing, McMaster University, Hamilton, ON, Canada

**CONTACT** Kathryn Deshaies kdeshaies@stclaircollege.ca

© 2018 Kathryn Deshaies, Sharon Kaasalainen and Noori Akhtar-Danesh. Published with license by Taylor & Francis Group, LLC.

This is an Open Access article distributed under the terms of the Creative Commons Attribution License (http://creativecommons.org/licenses/by/4.0/), which permits unrestricted use, distribution, and reproduction in any medium, provided the original work is properly cited.

**Introduction/Aim**: The primary objective of the study was to develop and test the psychometric properties of a chronic pain integration questionnaire (CPIQ) and examine several proposed relationships between chronic pain and integration constructs; enhancing knowledge of adjustment in chronic pain.

**Methods**: The current study employed a quantitative methodological design with psychometric methods. Additional measures used in the analysis were the Chronic Pain Acceptance Questionnaire (CPAQ), the Acceptance and Action Questionnaire (AAQ-II), a measure of health (SF-12v2), and a visual analog scale of social support.

**Results**: Analysis revealed test-retest reliability (ICC = .88); internal consistency reliability (α = .72); and beginning evidence of validity as demonstrated through confirmation of four research hypotheses: positive correlation with the CPAQ (.36; *p* ≤ .01); negative correlation with the AAQ-II (-.39; *p* ≤ .01); positive correlation with mental and general health of the SF-12v2 (.45 and .23 respectively; *p* ≤ .01) and social support (.35; *p* ≤ .01). Additionally, three domains of chronic pain integration were identified through exploratory factor analysis (self-management; self-awareness; intrinsic adjustment).

**Discussion/Conclusions**: Findings provide evidence for the psychometric properties of the CPIQ and its potential relationship to acceptance of chronic pain, psychological flexibility, health, and social support. The small number of items and ease of use enhances its continued use in research and practice to understand adjustment in chronic pain.

### Pain expectancies are associated with altered pain sensitivity in chronic low back pain patients

Junie S. Carriere^a^, Marc-Olivier Martel^b^, Samantha Meints^a^, Marise C. Cornelius^c^ and Robert R. Edwards^a^

^a^Department of Anesthesiology, Brigham and Women’s Hospital, Harvard Medical School, Chestnut Hill, MA, USA; ^b^Faculties of Dentistry and Medicine, McGill University, Montreal, Canada; ^c^Pain Management Center, Brigham and Women’s Hospital, Chestnut Hill, MA, USA

**CONTACT** Junie S. Carriere jcarriere@bwh.harvard.edu

© 2018 Junie S. Carriere, Marc-Olivier Martel, Samantha Meints, Marise C. Cornelius and Robert R. Edwards. Published with license by Taylor & Francis Group, LLC.

This is an Open Access article distributed under the terms of the Creative Commons Attribution License (http://creativecommons.org/licenses/by/4.0/), which permits unrestricted use, distribution, and reproduction in any medium, provided the original work is properly cited.

**Introduction/Aim**: Changes in central pain processing have been shown in patients with chronic low back pain (CLBP). Research has also shown that individuals with high pain expectancies report higher pain intensity and higher pain responses to noxious stimuli. However, research has yet to examine whether CLBP patients with high pain expectancies experience greater temporal summation of pain, a frequently used index of central nervous system sensitization. This study used quantitative sensory testing to identify differences in pain sensitivity between CLBP patients with high and low pain expectancies.

**Methods**: Patients (N = 437) with CLBP completed measures of pain intensity and pain expectancies prior to undergoing standardized a psychophysical pain-testing procedure designed to assess temporal summation of mechanical pain. Patients were classified as having high or low pain expectancies based on a median split.

**Results**: Results of independent samples t-tests indicated that during the temporal summation task, patients with high expectancies reported higher pain ratings in response to the mechanical probes than patients with low pain expectancies. Patients with high pain expectancies also reported higher ratings of painful after-sensations, and greater temporal summation of pain (p’s < .05).

**Discussion/Conclusions**: Taken together, these results indicate that CLBP patients with high pain expectancies show increased pain sensitivity and enhanced pain-facilatory processes, compared to patients with low pain expectancies. The findings of this study provide further evidence for changes in the central processing of pain in CLBP patients with high pain expectancies.

### Changes in self-efficacy, fear of moving, specific goals and physical performance of patients attending an interdisciplinary chronic pain program

Adria Fransson^a^ and Laura Katz^b^

^a^Physiotherapy, Michael G. DeGroote Pain Clinic, McMaster University Medical Centre, Hamilton, ON, Canada; ^b^C.Psych., Michael G. DeGroote Pain Clinic, McMaster University Medical Centre, Hamilton, ON, Canada

**CONTACT** Adria Fransson fransson@hhsc.ca

© 2018 Adria Fransson, and Laura Katz. Published with license by Taylor & Francis Group, LLC.

This is an Open Access article distributed under the terms of the Creative Commons Attribution License (http://creativecommons.org/licenses/by/4.0/), which permits unrestricted use, distribution, and reproduction in any medium, provided the original work is properly cited.

**Introduction/Aim**: The Michael G. DeGroote Pain Clinic offers an interdisciplinary pain program for patients with chronic pain. Research has demonstrated that increasing self-efficacy and decreasing fear of moving is associated with increasing patients confidence to move. As such, the aim of this study was to evaluate the effect of this program on self-efficacy, fear of moving, attainment of specific patient goals and physical performance.

**Methods**: Questionnaires were administered and completed by patients attending their first and last day of an 8-day interdisciplinary chronic pain program. The weekly program included group psych-educational, fitness, goal setting and relaxation classes. Questionnaires included the Pain Self Efficacy Questionnaire (PSEQ), Tampa Scale of Kinesiophobia (TSK), and Patient Specific Functional Scale (PSFS). Physical performance measures were the 6 minute walk test (6MWT), Timed Up and Go (TUG), and forward flexion of the spine. Data were analyzed using paired sample t-tests.

**Results**: The change in the 6MWT (*t *= 0–36, *p *= 0.72), TUG (*t *= 0.43, *p *= 0.67), and forward flexion (*t *= −0.25, *p *= 0.81) were not significant. However, the changes in the PSEQ (*t *= −3.21, *p *< 0.01), TSK (*t *= 2.10, *p *= 0.04), and the PSFS (*t *= −5.43, *p *< 0.01) were significant post-program.

**Discussion/Conclusions**: Physical measures did not significantly change pre- and post- 8-week Program, however, the PSEQ, TSK, and PSFS did change. These preliminary results imply that physiotherapists might want to evaluate the responsiveness of the physical performance measures being used, and continue to implement more functional and patient specific measures.

### The role of mTORC2 in the development of chronic pain

Calvin Wong^a^, Shannon Tansley^a^, Noosha Yosefpour^b^, Alfredo Ribeiro-Da-Silva^b^ and Arkady Khoutorsky^c^

^a^Anaesthesia, McGill University, Montreal, Quebec, Canada; ^b^Pharmacology & Therapeutics, McGill University, Montreal, Quebec, Canada; ^c^Anaesthesia, McGill Unicersity, Montreal, Quebec, Canada

**CONTACT** Calvin Wong calvin.wong3@mail.mcgill.ca

© 2018 Calvin Wong, Shannon Tansley, Noosha Yosefpour, Alfredo Ribeiro-Da-Silva and Arkady Khoutorsky. Published with license by Taylor & Francis Group, LLC.

This is an Open Access article distributed under the terms of the Creative Commons Attribution License (http://creativecommons.org/licenses/by/4.0/), which permits unrestricted use, distribution, and reproduction in any medium, provided the original work is properly cited.

**Introduction/Aim**: Neuropathic chronic pain can persist for years as a result of nerve injury. Inflammatory pain is commonly acute, and involves tissue damage due to inflammation. Changes associated with the development of pain involve the reorganization of pain circuitry, and alterations in gene expression. mTOR is a highly evolutionarily conserved serine/threonine kinase that regulates cell homeostasis through key cellular processes, including cell growth and proliferation, translation, autophagy, and cytoskeleton organization. mTOR is present in two structurally and functionally distinct multiprotein complexes: mTORC1 (mTOR Complex 1) and mTORC2. The activity of mTORC1 is required for the development of chronic pain, via regulation of mRNA translation. Much less is known about mTORC2, which has recently emerged as a key signaling molecule in a variety of cellular processes.

**Methods**: To study the role of mTORC2 in pain, we selectively ablated rictor, a key protein within the mTORC2, in Nav1.8-positive nociceptors. To ensure that behavioural effects are not a result of abberant developmental changes from the conditional knockout of Rictor, immunohistochemistry and western blot analysis were carried out. We also performed experiments to study the effect of rictor cKO on intracellular signaling following inflammation and tissue injury.

**Results**: Our behavioural experiments demonstrate that rictor conditional KO (cKO) mice exhibit reduced hypersensitivity in a mouse model of inflammatory pain, complete-freund’s adjuvant, but not in the model of neuropathic pain, spared-nerve injury.

**Discussion/Conclusions**: In summary, our study demonstrates for the first time the central role of mTORC2 in nociceptors in the development of pain hypersensitivity in response to inflammation.

### Structural plasticity of spinal dorsal horn touch circuitry in neuropathic pain

Noosha Yousefpour^a^, Maria Aparicio^a^, Samantha Locke^a^ and Alfredo Ribeiro-Da-Silva^a^

Pharmacology and Therapeutics, McGill University, Montreal, Canada

**CONTACT** Noosha Yousefpour noosha.yousefpour@mail.mcgill.ca

© 2018 Noosha Yousefpour, Maria Aparicio, Samantha Locke and Alfredo Ribeiro-Da-Silva. Published with license by Taylor & Francis Group, LLC.

This is an Open Access article distributed under the terms of the Creative Commons Attribution License (http://creativecommons.org/licenses/by/4.0/), which permits unrestricted use, distribution, and reproduction in any medium, provided the original work is properly cited.

**Introduction/Aim**: Pain induced by light touch (mechanical allodynia), is a symptom of neuropathic pain. It is thought that loss of inhibitory control of touch coding low-threshold mechanoreceptors (LTMRs) in the spinal dorsal horn is implicated in mechanical allodynia. This study focuses on nerve injury induced morphological changes in pre-synaptic inhibition on the central terminals of LTMRs and aims to find what mechanism underlies these changes. We hypothesize that loss of synapses between LTMRs and spinal GABAergic inhibitory neurons contributes to mechanical allodynia, and this loss is mediated by microglia.

**Methods**: Using Minocycline, we chronically inhibited microglia in neuropathic and control rats and measured mechanical allodynia weekly. After three weeks, we examined the effect of peripheral nerve injury and microglial inhibition on the integrity of LTMR terminals in the rat dorsal horn. Confocal and ultrastructural immunocytochemistry for LTMRs, inhibitory neurons and microglia were used to compare number of pre-synaptic inhibitory inputs per LTMR terminal and microglial engulfment of inhibitory synaptic elements, respectively.

**Results**: Following nerve injury, there was a loss of pre-synaptic inhibitory input on LTMRs. Compared to vehicle-treated rats, neuropathic animals treated with minocycline showed reduced mechanical allodynia and microgliosis. Furthermore, the loss of inhibitory synapses on LTMR terminals was less in minocycline-treated neuropathic animals.

**Discussion/Conclusions**: This study demonstrates that in a model of neuropathic pain, microglia contribute to mechanical allodynia by controlling a selective removal of inhibitory pre-synaptic input on LTMR terminals in the spinal dorsal horn.

### Effects of an exercise intervention on low back pain and cortico-limbic connectivity: preliminary results

Scott J. Thompson^a^, Florian Bobeuf^b^, Anna Bendas^a^, Francis Comte^b^, Pierre Rainville^b^, Louis Bherer^c^, Julien Cohen-Adad^b^ and Mathieu Roy^a^

^a^Department of Psychology, McGill University, Montreal, QC, Canada; ^b^Centre de recherche de l’Institut universitaire de gériatrie de Montréal, Montreal, QC, Canada; ^c^Centre de recherche de l’Institut universitaire de gériatrie de Montréal & Institut de Cardiologie de Montréal, Montreal, QC, Canada

**CONTACT** Scott J. Thompson scott.thompson4@mail.mcgill.ca Department of Psychology, McGill University, Montreal, QC, Canada

© 2018 Scott J. Thompson, Florian Bobeuf, Anna Bendas, Francis Comte, Pierre Rainville, Louis Bherer, Julien Cohen-Adad, and Mathieu Roy. Published with license by Taylor & Francis Group, LLC.

This is an Open Access article distributed under the terms of the Creative Commons Attribution License (http://creativecommons.org/licenses/by/4.0/), which permits unrestricted use, distribution, and reproduction in any medium, provided the original work is properly cited.

**Introduction/Aim**: Several studies have shown that exercise can decrease the pain, disability and comorbidities associated with chronic low back pain. Unfortunately, the mechanisms underlying these exercise-induced changes for chronic low back pain are unclear. A previous study (Baliki, 2012, DOI:10.1038/nn.3153) found that greater nucleus accumbens (NAc) - medial prefrontal cortex (mPFC) functional connectivity predicted which subacute low back pain subjects developed chronic low back pain (as opposed to those who recovered). Here we investigated whether or not NAc-mPFC functional connectivity is affected by exercise.

**Methods**: Twelve people (11 female, 1 male, age range 22–65) with chronic low back pain completed a three month strength and cardiovascular training program. At the beginning and end of the program, participants underwent magnetic resonance imaging (MRI) scans to assess brain functional connectivity. The MATLAB toolbox CONN(17f) was used to test the *a priori* hypothesis that the nucleus accumbens functional connectivity may play an important role in the effects of the intervention on chronic pain.

**Results**: After the exercise intervention, pain decreased significantly for the participants (Pre 4.33 ± 1.826, Post 2.42 ± 1.240, p < 0.01). Seed-to-voxel functional connectivity with the seed in bilateral nucleus accumbens revealed that functional connectivity to the medial prefrontal cortex decreased after the three-month exercise intervention (p-FWE = 0.005034, p-FDR = 0.002902, p-unc = 0.000055).

**Discussion/Conclusions**: Our findings suggest that NAc-mPFC connectivity, a potential indicator for subacute to chronic low back pain development, is reduced with exercise.

### Distress responses during vaccination as an indicator of early childhood mental health

Nicole M. Racine^a^, Hannah G. Gennis^b^, Rebecca Pillai Riddell^b,c,d^, Saul Greenberg^e^ and Hartley Garfield^f^

^a^Psychology, University of Calgary & Alberta Children’s Hospital Research Institute, Calgary, Alberta, Canada; ^b^Psychology, Faculty of Health, York University, The Opportunities to Understand Childhood Hurt Laboratory, Toronto, Ontario, Canada; ^c^Psychiatry Research, The Hospital for Sick Children, Toronto, Ontario, Canada, Toronto, Ontario, Canada; ^d^Psychiatry, University of Toronto, Toronto, Ontario, Canada; ^e^Pediatrics, Pediatric Medicine, University of Toronto, Toronto, Ontario, Canada; ^f^Pediatric Medicine, The Hospital for Sick Children, Toronto, Ontario, Canada

**CONTACT** Nicole Racine nicole.racine2@ucalgary.ca

© 2018 Nicole M. Racine, Hannah G. Gennis, Rebecca Pillai Riddell, Saul Greenberg, and Hartley Garfield. Published with license by Taylor & Francis Group, LLC.

This is an Open Access article distributed under the terms of the Creative Commons Attribution License (http://creativecommons.org/licenses/by/4.0/), which permits unrestricted use, distribution, and reproduction in any medium, provided the original work is properly cited.

**Introduction/Aim**: Distress regulation is established in early childhood and is critical for the development of children’s mental health and wellbeing. Routine vaccinations in primary care provide a unique opportunity to relate responses to a universal, relatively standardized, distress regulation paradigm (i. e., pain-related distress) to key developmental outcomes. The current study sought to examine distress regulation during routine vaccination in infancy and preschool as predictors of outcomes related to children’s mental health in preschool.

**Methods**: As part of an ongoing longitudinal cohort, 172 parent–child dyads (53.2% male children and 86% mothers) were videotaped during vaccinations in infancy and preschool, and subsequently participated in a full-day psychological assessment in a university lab. Videotapes were coded for child pre-needle distress (baseline distress), immediate post-needle pain-related distress reactivity (immediate distress reactivity), and pain-related distress regulation (distress regulation). Parent sensitivity during the preschool vaccination was also coded. Parents rated their child’s internalizing problems, externalizing problems, and behavioural symptoms at 4–5 years.

**Results**: Two significant main effects were found: (1) externalizing behavior was positively predicted by baseline preschool pain-related distress at *p* ≤ 0.01 (β = 0.23); and (2) behavior symptoms were positively associated with baseline preschool pain-related distress (β = 0.17, *p* < 0.05). Parent sensitivity did not moderate the association between any child distress behaviors and socioemotional development indicators.

**Conclusion**: Child distress behaviors prior to injection, regardless of parent behavior, during the vaccination context may provide valuable information to health care professionals about child socioemotional functioning in the behavioral and emotional domains.

### Predictors of response of persons participating in an intensive interdisciplinary program for treament of chronic pain

Adria Fransson^a^ and Jean Wessel^b^

^a^Michael G. DeGroote Pain Clinic, McMaster University Medical Centre, Hamilton, Ontario, Canada; ^b^Rehabilitation Science, McMaster University, Hamilton, Ontario, Canada

**CONTACT** Adria Fransson fransson@hhsc.ca

© 2018 Adria Fransson and Jean Wessel. Published with license by Taylor & Francis Group, LLC.

This is an Open Access article distributed under the terms of the Creative Commons Attribution License (http://creativecommons.org/licenses/by/4.0/), which permits unrestricted use, distribution, and reproduction in any medium, provided the original work is properly cited.

**Introduction/Aim**: The Michael G. DeGroote Pain Clinic offers an intensive 4-week interdisciplinary program for persons with chronic pain. The aim of this study was to determine if measurements taken at entry to the program could predict changes in self-efficacy, fear of movement and physical performance.

**Methods**: The data for analyses came from 222 (112 F, 110 M) consecutive patients admitted to the pain program over a 47 month period. The program included daily psycho-educational, fitness and relaxation classes, weekly group activation events and individual treatment with any team member. Cognitive behavioural therapy techniques were used within each component. The outcomes of interest were the changes in the Pain Self-Efficacy Questionnaire (PSEQ), the Tampa Scale of Kinesiophobia (TSK) and the Six Minute Walk Test (6MWT). Each of these change scores was the dependent measure in a regression equation with age, gender, number of painful body parts, number of co-morbidities and baseline scores of PSEQ, TSK and 6MWT entered as independent measures.

**Results**: For all three change scores, the best predictor was the baseline value of the measure (PSEQ r = −0.426, TSK r = 0.409, 6MWT r = −0.176). Adding the other independent variables resulted in values of r = 0.466 (r^2^ = 0.218), r = 0.460 (r^2^ = 0.211) and r = 0.271 (r^2^ = 0.073) respectively.

**Discussion/Conclusions**: More than 20% of the variance in changes in self-efficacy and fear of movement was explained by the baseline variables. The changes were greater in participants who started with poorer scores in all outcomes.

### Sex differences in analgesia induced by conditioned pain modulation, hypnosis and placebo treatments

Janie Damien^a^, Guillaume Léonard^b^, Philippe Chalaye^a^ and Serge Marchand^a^

^a^Neurosurgery, Université de Sherbrooke, Sherbrooke, Canada; ^b^School of Rehabilitation, Université de Sherbrooke, Shebrooke, Canada

**CONTACT** Janie Damien Janie.Damien@USherbrooke.ca

© 2018 Janie Damien, Guillaume Léonard, Philippe Chalaye and Serge Marchand. Published with license by Taylor & Francis Group, LLC.

This is an Open Access article distributed under the terms of the Creative Commons Attribution License (http://creativecommons.org/licenses/by/4.0/), which permits unrestricted use, distribution, and reproduction in any medium, provided the original work is properly cited.

**Introduction/Aim:** Analgesia induced by hypnosis, placebo and conditioned pain modulation (CPM) rely on the activation of endogenous pain inhibitory mechanisms. These pain-inhibitory effects could be related and involve sex differences. This study investigates the relationships between pain reduction produced by CPM, hypnosis and placebo treatments and explores sex differences.

**Methods:** Twenty-four healthy adults (12 men and 12 women; 18–45 years) were enrolled in this study. CPM was evaluated by comparing mean pain intensity induced by a two-minute heat test stimulus (HTS) performed before and after the cold pressor test (CPT; two-minute immersion, 10°C). The same HTS was completed before and after suggestions of analgesia in: 1) Standardized hypnosis intervention and 2) Placebo tablets (presented as analgesics).

**Results:** Pain intensity decreased significantly after CPT (p < .001), hypnosis (p < .001) and placebo (p = .006) interventions. CPM procedure produced significant pain relief in women (p = .008) and men (p = .02). Hypnosis more effectively reduced pain in women than in men (p = .04). In contrast, placebo treatment was effective in men (p = .01) but not in women (p = .1). No significant association was found between the hypoalgesic effects induced by CPM, hypnosis and placebo (ps > .05).

**Discussion/Conclusions:** CPM, hypnosis and placebo treatments were effective in reducing experimental heat pain in healthy adults. Findings suggest that the hypoalgesic effects induced by counter-irritation (CPM), hypnosis and placebo involve different mechanisms. Hypnosis was more analgesic in women and placebo was more effective for men. Our results suggest that interventions relying on endogenous pain-inhibitory mechanisms may present individual differences and should be considered to optimize their analgesic effects.

### Genetic association studies of temporomandibular disorder: a comprehensive review

Nicol Tugarinov^a^, Marc Parisien^a^, Luda Diatchenko^a^ and Carolina B. Meloto^b^

^a^The Alan Edwards Centre for Research on Pain,McGill University, Montreal,Quebec,Canada; ^b^Faculty of Dentistry, The Alan Edwards Centre for Research on Pain, McGill University,Montreal, Quebec, Canada

**CONTACT** Nicol Tugarinov nicol.tugarinov@mail.mcgill.ca

Luda Diatchenko and Carolina B. Meloto are equally contributed to the presented work.

© 2018 Nicol Tugarinov, Marc Parisien, Luda Diatchenko and Carolina B. Meloto. Published with license by Taylor & Francis Group, LLC.

This is an Open Access article distributed under the terms of the Creative Commons Attribution License (http://creativecommons.org/licenses/by/4.0/), which permits unrestricted use, distribution, and reproduction in any medium, provided the original work is properly cited.

**Introduction/Aim**: Painful temporomandibular disorders (TMDs) are the second most commonly occurring musculoskeletal conditions leading to pain and disability. Familial studies have provided evidence for heritability of TMD and genetic association studies have linked TMD to many genetic variants. Here, we summarized the current knowledge on the genetics of TMD and aimed to validate the association of all genetic variants with reported associations with TMD in independent cohorts.

**Methods**: Queries on MEDLINE/PubMed were performed using “TMD” AND “genetic association” AND “human” NOT “review”. “Genetic association” was replaced with: “variant”, “polymorphism”, “genetic polymorphism”, and “single nucleotide polymorphism”. We tested the association of all genetic variants with previously reported associations with TMD in 3 independent cohorts: the UK Biobank (UKBB, n = 167,320), the OPPERA (n = 3,104) and Brazilian-TMD (BRZ, n = 636) cohorts.

**Results**: Sixty-six genetic variants (38 unique genetic loci) have been associated with painful TMD in the literature. None were associated with facial pain in the UKBB. In OPPERA, four variants exhibited nominally significant associations with painful TMD: rs33389 (p = 0.041,*NR3C1*), rs143383 (p = 0.030,*GDF5*), rs3918242 (p = 0.023,*MMP9*), and rs60249166 (p = 0.021,*RXP2*). In the BRZ cohort, another two: rs4794106 (p = 0.032,*SGCA*) and rs12901499 (p = 0.006,*SMAD3*). Meta-analysis of OPPERA and BRZ cohorts revealed the significant association of rs60249166 with painful TMD (p = 0.009).

**Discussion/Conclusions**: Our findings indicate that rs60249166 (upstream of *RXP2*) is implicated in the pathophysiology of painful TMD. This variant was identified by a genome-wide association study (PMID:28081371) and our finding reinforces the dependability of such data-driven studies. The mechanisms underlying the implication of rs60249166 in painful TMD remain to be investigated.

### Reporting of pain assessment and management throughout the entire neonatal intensive care unit (NICU) stay

Adele Orovec^a^, Timothy Disher^b^ and Marsha Campbell-Yeo^c^

^a^Bachelor of Science in Medical Science, Faculty of Science, Dalhousie University, Halifax, Canada; ^b^School of Nursing, Dalhousie University, Halifax, Canada; ^c^School of Nursing, Departments of Pediatrics, Psychology and Neuroscience, Dalhousie University and IWK Health Centre, Halifax, Canada

**CONTACT** Adele Orovec aorovec@dal.ca

© 2018 Adele Orovec, Timothy Disher and Marsha Campbell-Yeo. Published with license by Taylor & Francis Group, LLC.

This is an Open Access article distributed under the terms of the Creative Commons Attribution License (http://creativecommons.org/licenses/by/4.0/), which permits unrestricted use, distribution, and reproduction in any medium, provided the original work is properly cited.

**Introduction/Aim**: Despite strong evidence that repeated pain exposure in neonates is associated with adverse outcomes, inadequate pain assessment and management has been reported with less than half receiving pain relief. This highlights the need to evaluate the current status of pain assessment and use of pain relieving interventions in this population. The aim of this study was to evaluate the level of pain assessment and management in a cohort of hospitalized Canadian preterm neonates throughout their entire stay.

**Methods**: A secondary analysis of study data collected from preterm neonates enrolled in a clinical trial and supplemental chart review.

**Results**: The 242 neonates included in the study underwent a total of 10468 painful procedures (4801 tissue breaking and 5667 non-tissue breaking with only 56.6% and 12.2% having a documented pain score using the Premature Infant Pain Profile (PIPP) respectively). Of those with a documented pain score the most likely procedures to receive a pain score were heel sticks (60.8%), venipunctures (58.4%) and peripherally inserted central catheters (56.8%). Procedures least likely to receive a pain score were suctioning (0.2%), tape removals (6.8%), and endotracheal tube insertions (7.4%). Having a PIPP score charted, having a tissue breaking procedure, and having a procedure during the day were associated with an increased likelihood of preprocedural pain relieving interventions. A pain-relieving intervention was only documented in 41.9% of procedures.

**Discussion/Conclusions**: There was considerable variation in reporting and treatment of pain. Increased efforts are needed to promote consistent pain assessment and management.

### Psilocybin and cluster headache: case report and literature review

Mark A. Ware^a^, Ziyu Xiao^b^ and Andrea Tone^c^

^a^Departments of Family Medicine and Anesthesia, McGill University, Montreal, Quebec, Canada; ^b^Medical student, McGill University, Montreal, Quebec, Canada; ^c^Department of Social Studies of Medicine, McGill University, Montreal, Quebec, Canada

**CONTACT** Mark A. Ware mark.ware@mcgill.ca Departments of Family Medicine and Anesthesia, McGill University, Montreal, Quebec, CanadaDepartments of Family Medicine and Anesthesia, McGill University, Montreal, Quebec, Canada

© 2018 Mark A. Ware, Ziyu Xiao and Andrea Tone. Published with license by Taylor & Francis Group, LLC.

This is an Open Access article distributed under the terms of the Creative Commons Attribution License (http://creativecommons.org/licenses/by/4.0/), which permits unrestricted use, distribution, and reproduction in any medium, provided the original work is properly cited.

A female (53y) experiences episodic cluster headaches (CHs) since age 19. There is a family history of headaches. She experiences episodic headache cycles behind the right eye with unpredictable onset, duration, and intensity. Prior treatments include calcium channel blockers, beta blockers, tricyclic antidepressants, SSRIs & SNRIs, magnesium IV, lithium, chiropractic, oxygen, anticonvulsants, topamax, Botox, lidocaine, capsaicin, occipital nerve blocks, nabilone, caffeine, solumedrol, ergotamine, melatonin, TENS, prednisone, IM opiates and Imitrex injections.

In summer 2017, the patient self-administered “magic mushrooms”. Patient had no history of illicit drug use. Patient ingested about 1/6 of a gram in June 2017. After 1½ hours this dosage had no effect, so she increased it to a total of about 1/4 of a gram. She denied hallucinations; the only side effect was excessive laughter. She ingested about ¼ of a gram again in August 2017, again well tolerated. The patient experienced a rapid resolution of the headache cycle.

“Magic mushrooms” contain psilocybin, a pro-drug of psilocin, a 5-HT2A receptor partial agonist^1^ responsible for the subjective effects of psilocybin.^2^ Psilocybin as a possible treatment for cluster headaches was discovered^3^ by a patient in 1993. Psilocybin has been reported as effective in 85% of patients surveyed^4^; for prophylactic treatment (90%) and remission (91%). Compared to first-line treatments such as verapamil, psilocybin showed promise. In a 2017 qualitative study,^5^ psilocybin was reported to be highly effective by patients suffering from cluster headaches. Psilocybin deserves further research for cluster headaches.

References1.Glennon
RA, Titeler
M, McKenney
JD. Evidence for 5-HT2 involvement in the mechanism of action of hallucinogenic agents. Life Sci. 1984;35(25):2505–11.651372510.1016/0024-3205(84)90436-32.Vollenweider
FX, Vollenweider-Scherpenhuyzen
MF, Bäbler
A, Vogel
H, Hell
D. Psilocybin induces schizophrenia-like psychosis in humans via a serotonin‐2 agonist action. Neuroreport. 1998;9(17):3897–902.987572510.1097/00001756-199812010-000243.Sewell
RA. Unauthorized research on cluster headache. Entheogen Rev. 2008;16:117–25.4.Sewell
RA, Halpern
JH, Pope
HG. Response of cluster headache to psilocybin and LSD. Neurology. 2006;66(12):1920–22.1680166010.1212/01.wnl.0000219761.05466.435.Andersson
M, Persson
M, Kjellgren
A. Psychoactive substances as a last resort—a qualitative study of self-treatment of migraine and cluster headaches. Harm Reduct J. 2017;14(1):60.2887022410.1186/s12954-017-0186-6PMC5584001

### Barriers and facilitators to using pain treatment during newborn screening blood tests at a mother-baby unit

Carolina Lavin Venegas^a,b^, Monica Taljaard^b,c^, Jessica Reszel^a^ and Denise Harrison^d,e^

^a^Children’s Hospital of Eastern Ontario Research Institute, Ottawa, Canada; ^b^School of Epidemiology and Public Health, University of Ottawa, Ottawa, Canada; ^c^Ottawa Hospital Research Institute, Ottawa, Canada; ^d^Children’s Hospital of Eastern Ontario, Canada; ^e^School of Nursing, University of Ottawa, Ottawa, Canada

**CONTACT** Carolina Lavin Venegas clavi052@uottawa.ca

© 2018 Carolina Lavin Venegas, Monica Taljaard, Jessica Reszel and Denise Harrison. Published with license by Taylor & Francis Group, LLC.

This is an Open Access article distributed under the terms of the Creative Commons Attribution License (http://creativecommons.org/licenses/by/4.0/), which permits unrestricted use, distribution, and reproduction in any medium, provided the original work is properly cited.

**Introduction/Aim**: Newborns undergo newborn screening blood tests. Breastfeeding, skin-to-skin care (SSC) and sweet solutions effectively reduce pain, however these strategies are inconsistently used.

**Methods**: A cross-sectional web-based survey administered to nurses working at a mother-baby unit. The 15-item questionnaire included demographics, nurses’ pain management practices during newborn screening (closed-ended questions) and open-ended questions regarding barriers and facilitators to using pain treatment. Participants were invited to view a brief video demonstrating breastfeeding, SSC, sucrose during bloodwork and report their intention to use each strategy on a 0 to 100 (0 = not at all likely, 100 = extremely likely) scale. Descriptive statistics and content analysis of open-ended questions was used.

**Results**: Thirty-six nurses participated in the survey (response rate = 80%). Thirty-five (97%) reported completing bloodwork outside the patient’s room, and 20 (56%) stated parents were sometimes present in the room during bloodwork. Breastfeeding was sometimes (44%) or rarely (41%) used; SSC was rarely (40%) or never (28%) used, while 53% reported often using sweet solutions. After viewing the video, participants were more likely to report an intention to use sweet solutions (mean = 82, SD = 35) compared to breastfeeding (mean = 44, SD = 36) or SSC (mean = 44, SD = 26). Perceived barriers to using breastfeeding and SSC were ergonomics, contextual factors (i. e. parents asleep), time, side-effects and environmental barriers. Barriers to using sweet solutions were no parental consent, lack of confidence in effectiveness and perceived side-effects. Facilitators included knowledge about effectiveness, ease of use and availability.

**Discussion/Conclusions**: Further work is warranted to address barriers to using evidence-based pain treatment for newborns during blood tests.

### The effects of cognitive fatigue on pain regulation

Bianca Chabot^a^, Todd A. Vogel^a^, Vanessa Tabry^b^ and Mathieu Roy^a^

^a^Department of Psychology, McGill University, Montreal, Canada; ^b^Faculty of Medicine, McGill University, Montreal, Canada

**CONTACT** Bianca Chabot bianca.chabot@mail.mcgill.ca

© 2018 Bianca Chabot, Todd A. Vogel, Vanessa Tabry and Mathieu Roy. Published with license by Taylor & Francis Group, LLC.

This is an Open Access article distributed under the terms of the Creative Commons Attribution License (http://creativecommons.org/licenses/by/4.0/), which permits unrestricted use, distribution, and reproduction in any medium, provided the original work is properly cited.

Individuals who suffer from chronic pain have been shown to demonstrate reduced cognitive functioning alongside their regularly experienced pain, significantly impacting daily life.

That is, pain can interrupt and hinder one’s ability to focus, thus making it more difficult to meet one’s goals. Previous evidence from clinical research demonstrated that individuals who showed impaired cognitive functioning were more susceptible to the development of chronic pain. The present study aimed to assess how laboratory-induced cognitive fatigue impairs cognitive functioning and pain regulation. We investigated the consequences of completing the cognitively demanding OSPAN Task on subsequent pain perception and task performance in the main experimental task. Healthy participants completed a cognitively demanding task (2-back task) and a non-demanding task (Left–Right arrow pointing task) while receiving painful thermal stimuli. Our findings showed that participants who were cognitively fatigued showed a reduction in ability to regulate pain compared to those who were not fatigued. These results suggest that cognitive fatigue may interfere with one’s capacity to cope with and distract oneself from pain.

**Introduction/Aim**: Individuals who suffer from chronic pain have been shown to demonstrate reduced cognitive functioning alongside their regularly experienced pain, significantly impacting daily life.

That is, interruption by pain can hinder one’s ability to focus on meeting a cognitive goal. Previous evidence from clinical research demonstrates that individuals who have impaired cognitive functioning are more susceptible to the development of chronic pain Attal et al.^1^ However, clinical studies do not allow for the manipulation of presumed causes, therefore a causal relationship of cognitive impairments on pain perception and of the interrupting effects of pain on cognition cannot be assessed. In the present study, laboratory-induced cognitive depletion was administered using the cognitively demanding OSPAN task prior to the main experimental task to observe its effects on subjective pain ratings and task performance. The main aim was to assess how cognitive resource depletion affected pain regulation while performing a high-load cognitive task (i. e. distraction).

**Methods**: Two groups of healthy young adults (control group: n = 41 of 41, experimental group: n = 11 of 40) were recruited to participate in the study. Participants completed a computerized cognitive task in order to measure task-induced analgesia—i. e., how a cognitively demanding task, compared to a less demanding task, distracts one from pain and therefore reduces pain ratings. More specifically, participants completed a cognitively demanding task (2-back task) and a less-demanding task (Left–Right arrow pointing task) while receiving painful thermal stimuli. The Operation-Span Task [OSPAN, Unsworth et al.^2^) was completed twice by the experimental group in order to deplete cognitive resources. Meanwhile, the control group did not complete any further tasks.

**Results**: The variances between the two groups were found to be unequal, Levene’s *F*(1) = 6.99, *p* = .011, therefore Welch’s *t*-test was used to examine mean differences between groups. Participants in the experimental group whose cognitive resources were depleted prior to performing the main task showed a near-significant reduction in task-induced analgesia compared to those who were not depleted, *t*(48.8) = 1.77, *p* = .082. In other words, a high-load task was less effective at reducing pain ratings when participants were cognitively depleted prior to performing the task. No differences were found in performance on the 2-back task between the two groups, Student’s *t*(49) = 0.67, *p* = .507.

**Discussion/Conclusions**: These results suggest that cognitive fatigue may interfere with one’s capacity to distract oneself from pain. Furthermore, chronic pain patients—who constantly experience cognitive difficulties—may have more difficulty in distracting themselves from pain and thus achieving cognitive goals.

References1.Attal, N., Masselin-Dubois, A., Martinez, V., Jayr, C., Albi, A., Fermanian, J., Bouhassira, D. and Baudic, S.
Does cognitive functioning predict chronic pain? Results from a prospective surgical cohort. Brain. 2014;137(3):904–17. doi:10.1093/brain/awt354.244411732.Unsworth
N, Heitz
RP, Schrock
JC, Engle
RW. An automated version of the operation span task. Behavior Research Methods. 2005;37(3):498–505. doi:10.3758/BF03192720.16405146

### Increased pain sensitivity and decreased opioid analgesia in t cell-deficient mice and implications for sex differences

Sarah F. Rosen^a^, Boram Ham^a^, Michael Haichin^a^, Ilana Walters^a^, Sarasa Tohyama^a^, Susana Sotocinal^a^ and Jeffrey S. Mogil^a^

Psychology, McGill University, Montreal, Canada

**CONTACT** Sarah Rosen srosen625@gmail.com

© 2018 Sarah F. Rosen, Boram Ham, Michael Haichin, Ilana Walters, Sarasa Tohyama, Susana Sotocinal and Jeffrey S. Mogil. Published with license by Taylor & Francis Group, LLC.

This is an Open Access article distributed under the terms of the Creative Commons Attribution License (http://creativecommons.org/licenses/by/4.0/), which permits unrestricted use, distribution, and reproduction in any medium, provided the original work is properly cited.

**Introduction/Aim**: The processing of pain in the central nervous system is now known to have an important immune component, including T cells of the adaptive immune system. T cells have been shown to release endogenous opioids, and although it is well known that opioids have effects on T cell populations, very little attention has been given to the converse: how T cells may affect opioid regulation.

**Methods**: CD-1, CD-1 nude, Rag1 null mutant and Cd4 null mutant of both sexes were used. Behavioral tests performed include Tail Withdrawal and Formalin Test. Morphine was given intraperitoneal at various doses.

**Results**: We find here that in addition to displaying significantly increased baseline pain sensitivity across various pain modalities, T cell deficient mice exhibit pronounced deficiencies in morphine inhibition of thermal or inflammatory pain. Nude mice are also deficient in endogenous opioid-mediated analgesia, exhibiting no stress-induced analgesia from restraint. The relevant T cell subpopulation appears to be CD4 + T cells, since adoptive transfer of them but not CD8+ cells into nude mice rescues both the pain and morphine analgesia phenotypes. As previously reported, we also observe a sex difference in CD-1 mice, with females requiring 2–3-fold more morphine than males to produce equal analgesia. Nude mice display no sex differences in morphine analgesia, and the sex difference is restored in nude mice of either sex receiving CD4 + T cells from CD-1 donor male or female mice.

**Discussion/Conclusions**: These results suggest that CD4 + T cells play an as yet unappreciated role in opioid analgesia, and may be a driver of sex differences therein.

### Spinal disinhibition during forced walking in a rat model of inflammatory arthritis

Samantha Locke^a^, Noosha Yousefpour^b^ and Alfredo Ribeiro-Da-Silva^b^

^a^Integrative Program of Neuroscience, McGill University, Montreal, Canada; ^b^Pharmacology and Therapeutics, McGill University, Montreal, Canada

**CONTACT** Samantha Locke samantha.locke@mail.mcgill.ca Integrative Program of Neuroscience, McGill University, Montreal, Canada

© 2018 Samantha Locke, Noosha Yousefpour, and Alfredo Ribeiro-Da-Silva. Published with license by Taylor & Francis Group, LLC.

This is an Open Access article distributed under the terms of the Creative Commons Attribution License (http://creativecommons.org/licenses/by/4.0/), which permits unrestricted use, distribution, and reproduction in any medium, provided the original work is properly cited.

**Introduction/Aim**: Movement-related pain is a major complaint of arthritis patients and remains poorly managed. In inflammatory arthritis, inflammation and pain do not always correlate, suggesting that mechanisms other than overt inflammation are involved. Microglia activation and spinal disinhibition are documented in neuropathic pain models but remain under-investigated in arthritis models.

**Methods**: Following ankle joint CFA injection, weight bearing assessment was performed before and after treadmill walking. Sham and CFA-treated animals were divided into either walking or non-walking groups. At 4 weeks post-CFA, 2 hours after walking, animals were sacrificed and processed for immunohistochemistry. Spinal neuronal activation was assessed using anti-fos antibodies, inhibitory neurons and microglia were identified with antibodies against paired box transcription factor (Pax2) and ionized calcium binding adaptor molecule 1 (Iba1), respectively. Antibodies against complement factors complement initiating factor C1q and C3 were used.

**Results**: Weight bearing showed a greater distribution of weight to the uninjured paw following walking as compared to the non-walking distribution. In the dorsal horn, fos expression was maximal in walked CFA-treated rats compared to non-walked CFA-treated animals, with the least fos expression in sham animals. Compared to sham groups, CFA-treated groups showed less activation of inhibitory neurons. Microgliosis was observed in CFA-treated spinal cords and walking resulted in differentially altered morphology of these cells in sham and CFA animals.

**Discussion/Conclusions**: These data suggest that forced walking induces changes in the spinal cord and weight bearing reflective of a pain state and that neuropathic-changes may underlie pain in relation to movement in this model.

### Influence of reward on pain perception and task performance

Todd A. Vogel^a^, Vanessa Tabry^b^ and Mathieu Roy^a^

^a^Department of Psychology, McGill University, Montreal, Quebec, Canada; ^b^Faculty of Medicine, McGill University, Montreal, Quebec, Canada

**CONTACT** Todd A. Vogel todd.vogel@mail.mcgill.ca

© 2018 Todd A. Vogel, Vanessa Tabry and Mathieu Roy. Published with license by Taylor & Francis Group, LLC.

This is an Open Access article distributed under the terms of the Creative Commons Attribution License (http://creativecommons.org/licenses/by/4.0/), which permits unrestricted use, distribution, and reproduction in any medium, provided the original work is properly cited.

Pain is a salient signal that interrupts current behavior and directs attention to itself. While this disruption may be advantageous evolutionarily, often we wish to suppress the pain signal to continue pursuing our goals. Engagement in a demanding cognitive task has been shown to reduce pain perception by distracting oneself from concurrent pain (e. g., Verhoeven et al., 2010). In this way, a trade-off between pain perception and performance of a task is observed. However, the mechanisms driving this trade-off are less clear. Here we investigated how the value of a task in the context of pain is influenced by additional monetary incentives.

**Introduction/Aim**: To examine how the value of a cognitively demanding task influences ongoing pain perception and task performance.

**Methods**: Ten people were recruited for the study. Participants completed a computerized task examining task analgesia, i. e., how a cognitively demanding task reduces ongoing pain perception compared to a less demanding task. During the task, participants received thermal stimulations while performing either a high-load 2-back task or a low-load Left–Right arrow task. For half of the trials, participants could win bonus money for good performance. No reward was offered for the other half of trials.

**Results**: Preliminary results revealed a main effect of reward whereby a rewarded cognitive task was more effective at reducing pain perception (i. e., more distracting) compared to a non-rewarded task, *F*(1,9) = 12.73, *p* = .006. An near-significant interaction between reward and task difficulty suggested that this effect of reward occurred only when the task was difficult (i. e., 2-back task), *F*(1,9) = 4.49, *p* = .063

**Discussion/Conclusions**: These findings suggest that reward and the value of a task may influence one’s motivation and ability to distract from pain and continue in the pursuit of a goal.

#### Modulation of pain in human Thoracolumbar spinal cord: an fMRI investigation

Ali Khatibi^a^, Hamed Dehghani^b^, Amir Hosein Batouli^b^ and Mohammad Ali Oghabian^b^

^a^McConnell Brain Imaging Centre, Montreal Neurological Institute, McGill University, Montreal, Canada; ^b^Neuroimaging and Analysis group (NIAG), Tehran university of Medical sciences, Tehran, Tehran, Iran

**CONTACT** Ali Khatibi ali.khatibi@gmail.com

Ali Khatibi and Hamed Dehghani are equally contributed to this abstract.

© 2018 Ali Khatibi, Hamed Dehghani, Amir Hosein Batouli and Mohammad Ali Oghabian. Published with license by Taylor & Francis Group, LLC.

This is an Open Access article distributed under the terms of the Creative Commons Attribution License (http://creativecommons.org/licenses/by/4.0/), which permits unrestricted use, distribution, and reproduction in any medium, provided the original work is properly cited.

**Introduction/Aim**: All theories of pain processing emphasized the role of the spinal cord but due to methodological limitations few studies used fMRI (and specifically BOLD signal) to characterize pain processing in the human spinal cord. Here, we aimed to investigate feasibility of imaging pain processing and pain modulation in the Thoracolumbar spinal cord.

**Methods**: Eighteen healthy male volunteers were scanned using a Siemens-Prisma-3T scanner (ZOOMit sequence, TR = 3000ms; Res = 2.5 × 2.5 × 4mm). Pressure-pain (m = 4.691 ± 0.577kg; 120% of the participant’s threshold) was applied in three blocks of 10 on the L5 dermatome. Before the second block, an ice-pack was placed on the forearm of the subject inside the scanner and was removed before the third block. Image processing was done using the SCT, FSL and in-house Matlab programs. To increase signal quality, the PNM was used for physiological noise removal, whereas slice-wise spinal motion parameters was used for movement-artifact detection. Group-level statistics were corrected for multiple comparisons using the GRF approach at the cluster-level (Z > 2.3, p(cluster)<0.05).

**Results**: Pain ratings showed that subjects perceived significantly less pain during the second block than the other two. Imaging results revealed that the activation following painful stimulation was observed at between T11-12 (Vertebral), ipsilateral to pain stimulation site and toward the dorsal horn. Modulation of activity during the second block was observed at T11.

**Discussion/Conclusions**: The DNIC protocol used in this study could significantly reduce pain in subjects and it was associated with the modulation of observed activity in the corresponding spinal cord segments.

#### The importance of integrating pain awareness and knowledge into the training of future health and safety at work professionals

Anaïs Lacasse^a^, Chúk Odenigbo^b^, Nancy Julien^a^ and Nabiha Benyamina Douma^a^

^a^Département des sciences de la santé, Université du Québec en Abitibi-Témiscamingue, Rouyn-Noranda, Canada; ^b^École de Santé publique, Université de Montréal, Montréal, Canada

**CONTACT** Anaïs Lacasse anais.lacasse@uqat.ca

© 2018 Anaïs Lacasse, Chúk Odenigbo, Nancy Julien and Nabiha Benyamina Douma. Published with license by Taylor & Francis Group, LLC.

This is an Open Access article distributed under the terms of the Creative Commons Attribution License (http://creativecommons.org/licenses/by/4.0/), which permits unrestricted use, distribution, and reproduction in any medium, provided the original work is properly cited.

**Introduction/Aim**: While many pain-awareness campaigns target healthcare professionals, the dissemination of said information within public spheres is critical to aid in mitigation and rehabilitation efforts. A good starting point is investigating the current knowledge and attitudes of health and safety at work (HSW) students since they are the believed (future) experts in the workplace.

**Methods**: During the winter semester in 2015, a web-based cross-sectional study was conducted amongst 88 students enrolled in the distance learning HSW undergraduate certificate program at the *Université du Québec en Abitibi-Témiscamingue* (Quebec, Canada). The study population was mostly made up of field workers, human resource staff, technicians and managers wanting to increase their understanding of HSW.

**Results**: Although 75% of respondents were already employed within HSW or human resources, only 13.6% chose to take the optional 45-hour course dedicated to pain within their program. A substantial proportion of respondents reported to intervene in cases involving acute (46.6%) or chronic pain (40.2%) in their line of work. Half of the respondents (51.9%) were not aware that chronic pain affects approximately 1 in 5 adults. When compared to published data about the provincial population, HSW students exhibited poorer knowledge and more negative attitudes towards people suffering from chronic pain (score of 35.4 ± 6.3 - Chronic Pain Myth Scale) than other groups in Quebec (healthcare professionals: 39.9 ± 4.1; chronic pain patients: 40.6 ± 4.0; people not suffering from chronic pain: 38.4 ± 4.7).

**Discussion/Conclusions**: Our results emphasize the need for mandatory pain education within programs for HSW professionals.

#### Determining the predictive value of sensitivity to physical activity in objectively measured activity levels in adults with chronic musculoskeletal pain

Daniel Flegg^a^, Arthur Woznowski-Vu^a^, Andrea Aternali^b^, Rebekah Wickens^c^ and Timothy H. Wideman^a^

^a^School of Physical and Occupational Therapy, McGill University, Montreal, Quebec, Canada; ^b^Psychology, McGill University, Montreal, Quebec, Canada; ^c^School of Physical and Occupational Therapy, Constance Lethbridge Rehabilitation Centre/McGill University, Montreal, Quebec, Canada

**CONTACT** Daniel Flegg daniel.flegg@mail.mcgill.ca

© 2018 Daniel Flegg, Arthur Woznowski-Vu, Andrea Aternali, Rebekah Wickens, and Timothy H. Wideman. Published with license by Taylor & Francis Group, LLC.

This is an Open Access article distributed under the terms of the Creative Commons Attribution License (http://creativecommons.org/licenses/by/4.0/), which permits unrestricted use, distribution, and reproduction in any medium, provided the original work is properly cited.

**Introduction/Aim**: Previous research has highlighted the challenge of predicting objective measures of daily activity by showing the limited predictive value of psychological factors and different indices of pain sensitivity. The primary aim of this investigation is to determine whether indices of Sensitivity to Physical Activity (SPA) predict objectively measured activity levels in adults with musculoskeletal pain.

**Methods**: 70 participants with chronic (>3 months) musculoskeletal pain performed the 6-Minute Walk Test (6-MWT). SPA was measured by evaluating the change in self-report pain intensity using a numeric scale from 0 (no pain) to 100 (most pain imaginable). Moreover, their peak pain during the task was recorded. Participants were briefed at the end of a training session on how to wear the triaxial accelerometer on their hip for 9 days. The participants were also shown how to log their wear times in a journal provided to them. A Pearson’s correlation was run to assess the relationship between SPA from the 6-MWT and daily activity levels measured by an accelerometer.

**Results**: Analyses showed significant negative correlations between peak pain and the distance traveled during the 6-MWT with objectively measured physical activity levels. However, SPA was not significantly correlated with objectively measured physical activity levels.

**Discussion/Conclusions**: This preliminary analysis demonstrates that a SPA index from the 6-MWT does not predict objectively measured activity levels in adults with chronic musculoskeletal pain. Future research should evaluate the predictive value of SPA measure using lifting tasks.

#### Does the radiographic severity of knee osteoarthritis correlate with the duration of symptoms, pain intensity and medication use?

Laetitia De Polo^a^, Manon Choinière^b^, Nathalie J. Bureau^c^, Madeleine Durand^d^, Alix Cagnin^a^ and Nicola Hagemeister^a^

^a^École de Technologie Supérieure (ÉTS), Laboratoire d’Imagerie et d’Orthopédie (LIO), Montréal, Québec; ^b^Anesthésiologie, Université de Montréal (UdeM), Montréal, Canada; ^c^Radiologie, radio-oncologie et médecine nucléaire, Université de Montréal (UdeM), Montréal, Canada; ^d^Médecine interne, Université de Montréal (UdeM), Montréal, Canada

**CONTACT** Laetitia De Polo laetitiadepolo@crchum.qc.ca

© 2018 Ecole de Technologie Supérieure de Montréal. Published with license by Taylor & Francis Group, LLC.

This is an Open Access article. Non-commercial re-use, distribution, and reproduction in any medium, provided the original work is properly attributed, cited, and is not altered, transformed, or built upon in any way, is permitted. The moral rights of the named author(s) have been asserted.

**Introduction/Aim**: The objective was to examine the relationships between Kellgren-Lawrence osteoarthritis (KL OA) grades, duration of symptoms, pain intensity and medication use in knee OA patients.

**Methods**: Patients were included if they rated their worst pain in the past 7 days≥4 on a 0–10 pain intensity scale and if they had a radiographic KL severity grade≥2 out of 4. Duration of symptoms in months and type(s) of medication taken in the past 3 months for pain relief of knee OA were recorded.

**Results**: 539 patients (66% of women) with a mean age of 63.5 years participated (SD:9.3). Duration of symptoms varied from 1 to 540 months (median = 48) and a longer duration was weakly associated with higher KL grades (Spearman’s r = 0.18,p < 0.001). KL grades explained a small proportion of the variability in the patients’ pain intensity scores (r = 0.15,p < 0.001). 30% of patients reported taking no pain medication; acetaminophen, nonsteroidal anti-inflammatory drugs, and other drugs/natural products were used by 34%, 42% and 24% respectively. 14% had had a cortisone injection and 10% viscosupplementation. The correlation between the number of distinct medication type taken and KL grades was weak (r = 0.13,p < 0.001). The type of medication was not related to KL grade, except weakly for acetaminophen and cortisone, more used by patients with higher KL grade (r = 0.15/r = 0.10, both p < 0.001).

**Discussion/Conclusions**: These results suggest that patients with higher KL grades use the same medication than patients with lower grades and that pain intensity is largely unrelated to knee OA radiographic severity. This stress the importance of tailoring interventions by taking into account patient’s specific needs to improve their condition.

#### Mental health-related quality of life and work performance in adults with knee osteoarthritis

Alix Cagnin^a^, Manon Choinière^b^, Nathalie J. Bureau^c^, Madeleine Durand^d^, Laetitia De Polo^a^ and Nicola Hagemeister^a^

^a^Laboratoire d’Imagerie et d’Orthopédie (LIO), École de Technologie Supérieure (ÉTS), Montréal, Québec, Canada; ^b^Anesthésiologie, Université de Montréal (UdeM), Montréal, Québec, Canada; ^c^Radiologie, radio-oncologie et médecine nucléaire, Université de Montréal (UdeM), Montréal, Québec, Canada; ^d^Médecine interne, Université de Montréal (UdeM), Montréal, Québec, Canada

**CONTACT** Alix Cagnin alix.cagnin.1@ens.etsmtl.ca

© 2018 Ecole de Technologie Supérieure de Montréal. Published with license by Taylor & Francis Group, LLC.

This is an Open Access article distributed under the terms of the Creative Commons Attribution License (http://creativecommons.org/licenses/by/4.0/), which permits unrestricted use, distribution, and reproduction in any medium, provided the original work is properly cited.

**Introduction/Aim**: The aim of this study was to determine the associations between mental health-related quality of life and loss of productivity while at work (presenteeism) among workers with knee osteoarthritis (OA).

**Methods**: Patients were included if they rated their worst pain in the past 7 days ≥4 on a 0–10 pain intensity scale and if they had a Kellgren-Lawrence (KL) OA severity grade ≥2 out of 4 on radiographs. Mental well-being was assessed with the health-related quality of life subscale of the SF12-v2 (M-QOLS) and the Hospital Anxiety and Depression Scale (HADS). Presenteeism was assessed with the Health and Work Performance Questionnaire: participants rated their performance during the past 4 weeks of work on a 0–100 scale (0 = the worst possible performance and 100 = no loss of performance).

**Results**: 212 patients from 53 clinics with a full or part-time job participated. 57.5% were women, the mean age was 57.1 years (standard deviation SD: 7.8). Mean scores on presenteeism were lower than 90/100 in 84% of the participants (mean = 78.1; SD:18.5), suggesting that a majority experienced loss of performance at work. Patients who had a M-QOLS<50 (mean standardized score in the healthy population) had significantly lower work performance (M-QOLS<50: 70.9, M-QOLS>50: 82.9; p < 0.001). This was confirmed by the significant correlation between presenteeism and HADS_depression scores (r = −0.40; p < 0.001), but presenteeism was only weakly correlated with HADS_anxiety (r = −0.19; p = 0.01).

**Discussion/Conclusions**: Poor mental health-related quality of life and especially high depression levels were associated with poor performance at work. These results point out the need for a biopsychosocial approach in the treatment of knee OA.

#### Demographic and clinical factors related to pain and analgesia in newborns: prospective study in neonatal intensive care units in Brazil

Mily Constanza Moreno Ramos^a^, Ligyana Korki de Candido^b^, Taine Costa^c^, Anna Caroline Leite^d^, Bruna Figueiredo Manzo^e^, Elysangela Dittz Duarte^f^ and Mariana Bueno^g^

^a^Mily Constanza Moreno Ramos, RN MScN, PhD candidate, School of Nursing of the University of Sao Paulo, Sao Paulo/SP, Brazil, milyconstanza@usp.br; ^b^Ligyana Korki de Candido, RN MScN, School of Nursing of the University of Sao Paulo, Sao Paulo/SP, Brazil, ligyanakorki@usp.br; ^c^Taine Costa, RN MScN, PhD candidate, School of Nursing of the University of Sao Paulo, Sao Paulo/SP, Brazil, tainecosta@usp.br; ^d^Anna Caroline Leite, Undergraduate Student, School of Nursing of the Federal University of Minas Gerais, Belo Horizonte/MG, Brazil, Brazil, anna.leitebh@gmail.com; ^e^Bruna Figueiredo Manzo, RN, PhD, School of Nursing of the Federal University of Minas Gerais, Maternal-child Nursing and Public Health Department, Belo Horizonte/MG, Brazil, brunaamancio@yahoo.com.br; ^f^Elysangela Dittz Duarte, RN, PhD, School of Nursing of the Federal University of Minas Gerais, Maternal-child Nursing and Public Health Department, Belo Horizonte/MG, Brazil, elysangeladittz@gmail.com; ^g^Mariana Bueno, RN, PhD, The Hospital for Sick Children, Child Health Evaluative Sciences, Toronto/ON, Canada, mariana.bueno@sickkids.ca

**CONTACT** Mily Constanza Moreno Ramos atselep@uoi.gr

© 2018 Mily Constanza Moreno Ramos, Ligyana Korki de Candido, Taine Costa, Anna Caroline Leite, Bruna Figueiredo Manzo, Elysangela Dittz Duarte and Mariana Bueno. Published with license by Taylor & Francis Group, LLC.

This is an Open Access article distributed under the terms of the Creative Commons Attribution License (http://creativecommons.org/licenses/by/4.0/), which permits unrestricted use, distribution, and reproduction in any medium, provided the original work is properly cited.

**Introduction/Aim**: To identify the type and frequency of stressful and painful procedures, the use of comforting and analgesic strategies, and to identify the relation between demographic and clinical variables with procedures and pain management during newborns’ hospitalization in Neonatal Intensive Care Units (NICU).

**Methods**: This prospective study was conducted in two Brazilian NICU between June 2014 and January 2015. Data were collected on a daily basis during newborns’ hospitalization. Quantitative analysis was performed. The study was approved by local research ethics committee.

**Results**: The 140 newborns included underwent 21.291 painful and stressful procedures during hospitalization. Of these, 18.131 procedures were considered stressful and 3.160, painful. Opioids were poorly administered (0.6%), and the most implemented non-pharmacological strategies included comforting measures (80.1%), breastfeeding (6.4%), skin-to-skin contact (2.4%), and sweet solutions (2.0%). Significant correlations between number of painful procedures and length of hospitalization (0.964), weight gain (0.791), and head circumference gain (0.427) were found. Negative correlations between pharmacological analgesia and gestational age at birth (-0.601), and birth weight (-0.140) were observed. For each day of hospitalization, the use of non-pharmacological strategies increased by 3.4% and pharmacological strategies, increased by 9.3%. For each week of gestational age at birth, number of painful procedures increased by 2.9, use of non-pharmacological strategies decreased by 0.06%, and use of pharmacological strategies, increased by 23.7%.

**Discussion/Conclusions**: Neonates underwent a high number of stressful and painful procedures and pain management was suboptimal during their entire hospitalization period. Changing practice strategies to improve neonatal pain management are required.

#### The role of intravenous lidocaine in the management of chronic neuropathic pain of peripheral nerve origin

Zameer Pirani^a^, Patricia Morley-Forster^a^, Larry Stitt^a^ and Dwight Moulin^b^

^a^Anesthesia and Peri-Operative Medicine, Western University, London, Canada; ^b^Clinical Neurological Sciences and Oncology, Western University, London, Canada

**CONTACT** Zameer Pirani zpirani@uwo.ca Anesthesia and Peri-Operative Medicine, Western University, London, Canada

© 2018 Zameer Pirani, Patricia Morley-Forster, Larry Stitt and Dwight Moulin. Published with license by Taylor & Francis Group, LLC.

This is an Open Access article distributed under the terms of the Creative Commons Attribution License (http://creativecommons.org/licenses/by/4.0/), which permits unrestricted use, distribution, and reproduction in any medium, provided the original work is properly cited.

**Introduction/Aim**: Neuropathic pain results from injury to the peripheral or central nervous system. Consensus guidelines provide an evidence-based step-wise pharmacological approach to treatment. However, studies consistently show that 50 percent of patients do not achieve adequate relief.

Several placebo-controlled studies show that IV lidocaine infusion at 5 mg/kg provides significant pain relief for neuropathic pain for up to six hours; but no RCT has examined its long-term efficacy.

Our objective was to determine if IV lidocaine provides significant analgesia and improvement in quality of life for up to four weeks in patients with chronic neuropathic pain.

**Methods**: This was a single site randomized double-blind, crossover trial. Subjects were randomized to receive either intravenous lidocaine (5 mg/kg) or active placebo infusion (diphenhydramine). After 4 weeks, they returned for the alternate infusion. Data on average pain intensity, physical functioning, and quality of life measures were collected for each week 1,2,3 and 4 as per IMMPACT recommendations.

**Results**: 34 subjects were enrolled. Mean age was 58 years with an average pain duration of 89.4 months (±SD 57.4). The mean daily morphine equivalent dose (MED) was 195.5 mg (±SD 163.4 mg). No difference was observed at week 1,2,3, or 4 in pain intensity or in secondary outcomes.

**Discussion/Conclusions**: This RCT did not show significant long term analgesic or quality of life benefit for chronic neuropathic pain. However, 80% of our sample had diabetic neuropathy. There may still be a role for IV lidocaine in other neuropathic pain disorders of shorter duration.

#### The role of vitamin D level and vitamin D receptor ‎polymorphisms on migraine in an Iranian population

Alireza Zandifar^a^, Faraidoon Haghdoost^a^, Navid Manouchehri^a^, Omid Shafaat^a^ and Mohammad Saadatnia^b^

^a^Medical Student Research Center, Isfahan University of Medical Sciences, Isfahan, Iran; ^b^Neurology Department, Isfahan Neurosciences Research Center, Isfahan University of Medical Sciences, Isfahan, Iran

**CONTACT** Alireza Zandifar, M.D ar_zandifar@yahoo.com Medical Student Research Center, Isfahan University of Medical Sciences, Hezar jarib Avenue, Isfahan, 81745-319, Iran.

© 2018 Alireza Zandifar, Faraidoon Haghdoost, Navid Manouchehri, Omid Shafaat and Mohammad Saadatnia. Published with license by Taylor & Francis Group, LLC.

This is an Open Access article distributed under the terms of the Creative Commons Attribution License (http://creativecommons.org/licenses/by/4.0/), which permits unrestricted use, distribution, and reproduction in any medium, provided the original work is properly cited.

**Introduction/Aim**: The role of vitamin D as an effective anti-inflammatory agent is still unclear in ‎pathogenesis of migraine. There is no study to examine the role of vitamin D level and VDR ‎polymorphisms on migraine simultaneously. To investigate the associations between 25(OH) D level and two vitamin D receptor (VDR) ‎polymorphisms (TaqI and FokI) and migraine. ‎

**Methods**: 142 newly diagnosed migraineurs and 158 controls, matched for age and sex were ‎enrolled. 25(OH) D plasma levels and genotypic and allelic frequencies were assessed. T-test, ‎chi-square, and logistic regression analysis were used for data analysis.

**Results**: Vitamin D level was not significantly different between cases and controls. ‎Heterozygote genotypes were statistically more frequent in the patients than the controls both ‎for TaqI and FokI. Also, heterozygote genotypes (Ff/Tt) of VDR played an independent ‎risk factor role in migraine. The separate analysis of vitamin D levels between different genotypes ‎within cases and controls showed a counteractive result. There was a significant difference of ‎level of vitamin D between TT and Tt genotypes within controls (13.86 ± 8.81 vs. 9.67 ± 5.44, ‎P < 0.05) and also a significant difference between FF and Ff (14.50 ± 10.93 vs. 10.50 ± 7.28, P< ‎‎0.05) within cases.‎

**Discussion/Conclusions**: We found no relation between migraine and serum vitamin D. However TaqI ‎and FokI gene polymorphisms of VDR are associated with migraine but vitamin D levels ‎between different genotypes of VDR within cases and controls showed a counteractive ‎result. These results could be elucidated no differences between the level of vitamin D between ‎migraineurs and controls.

#### Effects of massage in reducing the pain and anxiety of the cardiac surgery critically Ill- a randomized controlled trial

Madalina Boitor^a^, Géraldine Martorella^b^, Andréa Maria Laizner^a^, Christine Maheu^a^ and Céline Gélinas^a^

^a^Ingram School of Nursing, McGill University, Montréal, Qc, Canada; ^b^College of Nursing, Florida State University, Florida, USA

**CONTACT** Madalina Boitor madalina.boitor@mail.mcgill.ca

© 2018 Madalina Boitor, Géraldine Martorella, Andréa Maria Laizner, Christine Maheu, and Céline Gélinas. Published with license by Taylor & Francis Group, LLC.

This is an Open Access article distributed under the terms of the Creative Commons Attribution License (http://creativecommons.org/licenses/by/4.0/), which permits unrestricted use, distribution, and reproduction in any medium, provided the original work is properly cited.

**Introduction/Aim**: To evaluate the effectiveness of hand massage on the pain and anxiety of the cardiac surgery critically ill.

**Methods**:

*Design*: A three-arm randomized controlled trial.

*Setting*: This study was conducted in a medical-surgical intensive care unit in Canada.

*Subjects*: Adult patients who underwent elective cardiac surgery, able to speak French/English and to self-report symptoms, without a high risk of postoperative complications were eligible.

*Procedures*: Patients were randomly allocated to standard care plus either two 20-minute hand massages (experimental), two 20-minute hand holdings (active control) or two 20-minute rest periods (passive control/standard care). Pain intensity, pain unpleasantness, anxiety, muscle tension and vital signs were evaluated before, after, and 30 minutes later for each intervention.

**Results**: From the 83 patients recruited, 60 were randomized (20 massage, 19 hand holding, 21 standard care). After controlling for baseline scores, the massage group reported significantly lower pain intensity, pain unpleasantness and anxiety for the 1st data collection set compared to both hand holding and standard care (ANCOVA, p < 0.02) with an average decrease of 2 points on a 0–10 scale. No statistically significant differences were noted between hand holding and standard care for any of the symptoms. Similar results were observed for the 2nd data collection set (n = 43). Patients had decreased muscle tension post-massage. Vital signs did not differ significantly between groups.

**Discussion/Conclusions**: Findings suggest that a 20-minute hand massage in addition to routine postoperative pain management can concomitantly reduce pain intensity, pain unpleasantness and anxiety by 2 points on average on a 0–10 scale.

#### Associations between infant pain behaviours and heart rate measures during vaccination across the second year of life

Jordana Waxman^a^, Miranda DiLorenzo^a^, Rebecca Pillai Riddell^a^ and Hartley Garfield^b^

^a^Psychology, York University, Toronto, ON, Canada; ^b^Pediatrics, University of Toronto, Toronto, ON, Canada

**CONTACT** Jordana Waxman waxmanja@yorku.ca

© 2018 Jordana Waxman, Miranda DiLorenzo, Rebecca Pillai Riddell, and Hartley Garfield. Published with license by Taylor & Francis Group, LLC.

This is an Open Access article distributed under the terms of the Creative Commons Attribution License (http://creativecommons.org/licenses/by/4.0/), which permits unrestricted use, distribution, and reproduction in any medium, provided the original work is properly cited.

**Introduction/Aim**: Although multidimensional infant pain scales are pervasive in the hospital setting, little research has investigated the associations between behavioural and physiological indexes in typically developing infants within a pain context. The aim of this study is to provide preliminary data on the relationship between infant pain behaviours and cardiovascular indices during vaccinations across the second year of life.

**Methods**: The data is part of an ongoing longitudinal study that follows caregivers and infants through the second year of life (12 [*N = 82*], 18 [*N = 45*], and 24 months [*N = *33]) during their well baby visits. Behavioural and cardiac data were simultaneously collected from 1-minute pre-needle (baseline) until 3-minutes post-vaccination. Behavioural distress was measured using the Face Legs Activity Cry Consolability (FLACC) coding system (15-s epochs). Heart rate (HR) and respiratory sinus arrhythmia (RSA) values were calculated using MindWare technologies (30-s epochs). Bivariate Pearson’s correlations were used to test the relationship between infant pain behaviours and HR/RSA during the vaccination.

**Results**: Across the second year of life, FLACC and HR were positively correlated across the baseline and post-vaccination periods. Depending on the age of measurement, FLACC and RSA were negatively correlated at baseline (12-months) and post-vaccination (24-months) periods. For both cardiovascular indices, the strength of the correlations with FLACC increased across the year (medium to large effect sizes, HR *r = *.37-.80, RSA *r = *.33-.56).

**Discussion/Conclusions**: This study provides preliminary evidence that as infants develop over the second year of life, physiological and behavioural indexes of distress become increasingly associated.

#### Gender differences in relation between headache-related ‎disability and anxiety/depression based on MIDAS and HADS ‎questionnaires among migraine patients

Omid Shafaat, M.D.^a^, Faraidoon Haghdoost^a^, Alireza Zandifar^a^ and Mohammad Saadatnia^b^

^a^Medical Student Research Center, Isfahan University of Medical Sciences, Isfahan, Iran; ^b^Isfahan Neurosciences Research Center, Isfahan University of Medical Sciences, Isfahan, Iran

**CONTACT** Omid Shafaat, M.D. omid.shafaat@yahoo.com M.D. Medical Students Research Center, Isfahan University of Medical Sciences, Hezar-Jerib Ave, Isfahan, Iran, 8174673461, +98 31 3670 0479

© 2018 Omid Shafaat, Faraidoon Haghdoost, Alireza Zandifar and Mohammad Saadatnia. Published with license by Taylor & Francis Group, LLC.

This is an Open Access article distributed under the terms of the Creative Commons Attribution License (http://creativecommons.org/licenses/by/3.0/), which permits unrestricted use, distribution, and reproduction in any medium, provided the original work is properly cited. The moral rights of the named author(s) have been asserted.

**Introduction/Aim**: Migraine is one of the most disabling disorders in the world with a high score ‎of DALY. On the other hand, some evidence showed anxiety and depression were more ‎prevalent in patients suffering from migraine. The objectives of the present study were to determine whether headache-related ‎disability correlates with depression and anxiety based on MIDAS and HADS questionnaires ‎and if that correlation differs by gender.‎

**Methods**: In this cross-sectional study, we included migraine patients based on ICHD-2 ‎criteria from four clinics in Isfahan, Iran. HADS and MIDAS were fulfilled by the patients. ‎Regards to odd questions summation of HADS scores’ we determined depression score, and ‎we calculated anxiety score by summing the even questions of HADS scores. Pearson ‎correlation was used for calculating the relation between quantitative variables.

**Results**: In this study, 205 migraine patients with mean (±SD) age of 31.05 ± 9.2 and 72.2% ‎female gender were included. There was a significant positive correlation between depression ‎and anxiety scores with MIDAS total score in all of the patients (r = 0.38 and 0.36 ‎respectively, P < 0.01). A separate analysis in male and female subjects showed a high level of ‎correlation between depression/anxiety scores and disability only in male subjects (r = 0.77 and ‎‎0.79 respectively, P < 0.001). There was no significant relationship in female subjects (r = 0.27 and ‎‎0.16 respectively).‎

**Discussion/Conclusions**: Results of this study showed the correlations between headache-related disability and depression/anxiety were significantly higher among male subjects than females. Probably gender is a significant predictor of correlation between disability and anxiety/depression in migraineurs.

#### Chronic pain management among people who use illicit drugs in the context of the “opioid crisis”: a qualitative study

Lise Dassieu^a^, Jean-Luc Kaboré^b^, Manon Choinière^b^ and Élise Roy^c^

^a^Faculté de Médecine et des Sciences de la Santé, Chaire de Recherche en toxicomanie, Université de Sherbrooke, Longueuil, Québec, Canada; ^b^Carrefour de l’innovation et de l’évaluation en santé, Centre de Recherche du Centre hospitalier de l’Université de Montréal, Montréal, Québec, Canada; ^c^Faculté de Médecine et des Sciences de la Santé, Chaire de Recherche en toxicomanie, Université de Sherbrooke, Longueuil, Québec, Canada

**CONTACT** Lise Dassieu Lise.Dassieu@USherbrooke.ca

© 2018 Lise Dassieu, Jean-Luc Kaboré, Manon Choinière, and Élise Roy. Published with license by Taylor & Francis Group, LLC.

This is an Open Access article distributed under the terms of the Creative Commons Attribution License (http://creativecommons.org/licenses/by/4.0/), which permits unrestricted use, distribution, and reproduction in any medium, provided the original work is properly cited.

**Introduction/Aim**: Literature suggests that chronic pain (CP) among people who use drugs (PWUD) is both highly prevalent and undertreated. This study aims to understand how PWUD with CP describe their access to CP management in the context of opioid prescription restrictions recommended by several health authorities to address the “opioid crisis”.

**Methods**: This ongoing qualitative study is based on in-depth interviews with community-based PWUD who used either cocaine/crack, stimulants, heroin or other opioids, and experienced CP for more than 3 months in Montreal (Canada). Interviews were analyzed through grounded theory comparative method.

**Results**: Seventeen participants have been interviewed so far (07/2017 to 11/2017). A good number of them reported having been denied prescription opioids for pain relief, even those with severe CP. While this phenomenon already existed, participants thought that physicians’ reluctance increased since the “opioid crisis” became mediatic. The situation is further complicated by the fact that participants cannot afford non-pharmaceutical alternatives such as physiotherapy. Furthermore, several participants felt unfairly treated by physicians, like if they were lying about their condition.

**Discussion/Conclusions**: Our findings are consistent with studies documenting the presence of the “drug-seeking-addict” representation among professionals treating PWUD for CP, and the powerlessness of physicians due to insufficient alternatives to opioids. This study suggests that the “opioid crisis” may contribute to increased defiance among physicians towards PWUD who suffer from CP. Actions are needed to help physicians recognize CP as a real issue for PWUD as for any patient, and to improve pain management for this population.

#### Effect of pain severity on quality of life based on demographic features among patients with migraine

Navid Manouchehri^a^, Alireza Zandifar^b^, Omid Shafaat^b^, Faraidoon Haghdoost^b^ and Mohammad Saadatnia^c^

^a^Isfahan Neurosciences Research Center, Isfahan University of Medical Sciences, Isfahan, Iran; ^b^Medical Student Research Center, Isfahan University of Medical Sciences, Isfahan, Iran; ^c^Neurology Department, Isfahan Neurosciences Research Center, Isfahan University of Medical Sciences, Isfahan, Iran

**CONTACT** Navid Manouchehri, M.D dr.navid.manouchehri@gmail.com Isfahan Neurosciences Research Center, Isfahan University of Medical Sciences, Hezar jarib Avenue, Isfahan, 81745-319, Iran.

© 2018 Navid Manouchehri, Alireza Zandifar, Omid Shafaat, Faraidoon Haghdoost, and Mohammad Saadatnia. Published with license by Taylor & Francis Group, LLC.

This is an Open Access article distributed under the terms of the Creative Commons Attribution License (http://creativecommons.org/licenses/by/4.0/), which permits unrestricted use, distribution, and reproduction in any medium, provided the original work is properly cited.

**Introduction/Aim**: Headaches compromise quality of life (QOL) among migraine patients; however its effect on QOL may vary based on demographic features. We aimed to assess these effects on QOL assessments between different headache severities among migraine patients.

**Methods**: In this cross-sectional study, migraine headache severity and QOL was assessed using HIT-6 (Headache Impact Test) and SF-36 questionnaires respectively. Headache was categorized as either mild-moderate or severe based on HIT-6 scores. Physical and mental components of SF-36 were used for assessment of QOL. Differences of QOL scores within each demographic feature across headache severity groups were assessed; including gender, age, urban/rural residence, educational attainment, occupation and weekly working hours.

**Results**: We assessed headache severity and QOL among 209 migraine patients. The mean±SD of age was 31.04 ± 9.1 years and 72.7% of the patients were female. There was a significant negative correlation between HIT-6 and both physical and mental components(r = −0.15 and −0.4 respectively, P < 0.05). Separate analysis of physical and mental SF-36 scores based on demographic features revealed that despite the primary assumption, when patients were female, younger than 30, living in cities or had University education, across the two headache severity groups there were no significant differences between SF-36 physical scores; Also, SF-36 mental score was not significantly different between headache severity groups when patients had University degree.

**Discussion/Conclusions**: We found that in migraine headache, certain demographic features including female sexuality, younger age, urban life style and advanced educations may impair the correlation between headache severity and QOL (especially physical component), requiring attention during data interpretation.

#### The reach of the #KidsCancerPain campaign: a partnership to improve parent awareness and use of evidence-based pain management in children with cancer

Jennifer A. Parker^a^, Christine T. Chambers^b^, Jennifer N. Stinson^c^, Perri R. Tutelman^d^, Emily K. Drake^e^, Melanie Barwick^f^, Fiona Campbell^g^, Conrad V. Fernandez^h^, Karen Irwin^i^, Lindsay Jibb^j^, Paul Nathan^k^ and Holly Witteman^l^; ^f^Child Health Evaluative Sciences & Department of Psychiatry, The Hospital for Sick Children & University of Toronto, Toronto, Ontario, Canada; ^g^Department of Anesthesia, The Hospital for Sick Children & University of Toronto, Toronto, Ontario, Canada; ^b^Departments of Pediatrics & Psychology and Neuroscience, Dalhousie University & IWK Health Centre, Halifax, Nova Scotia, Canada; ^c^Child Health Evaluative Sciences & Lawrence S. Bloomberg Faculty of Nursing, The Hospital for Sick Children & University of Toronto, Toronto, Ontario, Canada; ^d^Department of Psychology and Neuroscience, Dalhousie University, Halifax, Nova Scotia, Canada; ^e^Independent Healthcare Consultant, Halifax, Nova Scotia, Canada

^a^IWK Health Centre, Centre for Pediatric Pain Research, Halifax, Nova Scotia, Canada; ^h^Department of Pediatrics, IWK Health Centre and Dalhousie University, Halifax, Nova Scotia, Canada; ^i^Cancer Knowledge Network, Milton, Ontario, Canada; ^j^Nursing Sciences, University of Ottawa, Ottawa, Canada; ^k^Child Health Evaluative Sciences & Paediatrics and Health Policy, Management & Evaluation, The Hospital for Sick Children &University of Toronto, Toronto, Ontario, Canada; ^l^Department of Family & Emergency Medicine, Université Laval, Quebec City, Quebec, Canada

**CONTACT** Dr. Christine T Chambers Christine.Chambers@dal.ca

© 2018 Jennifer A. Parker, Christine T. Chambers, Jennifer N. Stinson, Perri R. Tutelman, Emily K. Drake, Melanie Barwick, Fiona Campbell, Conrad V. Fernandez, Karen Irwin, Lindsay Jibb, Paul Nathan and Holly Witteman. Published with license by Taylor & Francis Group, LLC.

This is an Open Access article distributed under the terms of the Creative Commons Attribution License (http://creativecommons.org/licenses/by/4.0/), which permits unrestricted use, distribution, and reproduction in any medium, provided the original work is properly cited.

**Introduction/Aim**: Children with cancer experience pain, often severe and prolonged, throughout their disease and treatment. Knowledge-to-action gaps are well documented and unfortunately, children with cancer do not always receive the best pain management possible. Parents are increasingly using social media for health information and the translation of evidence-based health knowledge over social media has the potential to reach parents when they need it most. We aim to describe the reach (i. e., potential audience size) of a social media campaign, ‘Making Cancer Less Painful for Kids’ (#KidsCancerPain), intended to improve parent awareness and use of evidence-based pain management in children with cancer.

**Methods**: The campaign shared research evidence in a parent-friendly manner via a partnership with Cancer Knowledge Network (CKN). It was led by a team of interdisciplinary scientists/clinicians, engaged a parent/patient panel, and had the support of various well-known cancer and pain organizations.

**Results**: The campaign (July 2016-September 2017) consisted of a targeted dissemination of content about children’s cancer pain via 1 Thunderclap, 7 blog posts, 3 videos, 9 social media images, 5 Facebook questions, and 1 Twitter chat. Twitter social listening data indicated a reach (i.e., estimated unique users who saw #KidsCancerPain tweets) of 3.4 million with 6,812 tweets by 2,337 users. Additional reach analytics, such as content views, will be presented.

**Discussion/Conclusions**: The #KidsCancerPain campaign demonstrates the effectiveness of social media and partnerships in reaching a large audience to increase awareness. The impact of spreading research evidence via social media on pain outcomes in the pediatric oncology population should be evaluated.

#### The relationship between pain and somatization in pediatric populations: A systematic review of the current state of the research literature

Katelynn E. Boerner^a^, Amrit Dhariwal^b^, Katie Green^c^, Andrea Chapman^d^, Theresa Newlove^e^ and Elizabeth Stanford^f^

^a^Katelynn Boerner, PhD, BC Children’s Hospital, Mental Health, Vancouver, British Columbia, Canada, katelynn.boernerwall@cw.bc.ca, @KatelynnBoerner; ^b^Amrit Dhariwal, PhD R.Psych, BC Children’s Hospital and University of British Columbia, Mental Health, Vancouver, British Columbia, Canada, Amrit.Dhariwal@cw.bc.ca; ^c^Katie Green, BSc, University of British Columbia, Experimental Medicine, Vancouver, British Columbia, Canada, katherine.green@cw.bc.ca; ^d^Andrea Chapman, MD, FRCPC, BC Children’s Hospital and University of British Columbia, Psychiatry, Vancouver, British Columbia, Canada, achapman@cw.bc.ca; ^e^Theresa Newlove, PhD R.Psych, BC Children’s Hospital and University of British Columbia, Psychology, Vancouver, British Columbia, Canada, tnewlove@cw.bc.ca; ^f^Elizabeth Stanford, PhD R.Psych, BC Children’s Hospital and University of British Columbia, Psychology, Vancouver, British Columbia, Canada, Elizabeth.Stanford@cw.bc.ca

**CONTACT** Katelynn E. Boerner katelynn.boernerwall@cw.bc.ca

© 2018 Katelynn Boerner, Amrit Dhariwal, Katie Green, Andrea Chapman, Theresa Newlove and Elizabeth Stanford. Published with license by Taylor & Francis Group, LLC.

This is an Open Access article distributed under the terms of the Creative Commons Attribution License (http://creativecommons.org/licenses/by/4.0/), which permits unrestricted use, distribution, and reproduction in any medium, provided the original work is properly cited.

**Introduction/Aim**: Chronic pain and somatization are highly prevalent experiences often emerging in childhood with significant functional consequences. The role of somatization in pain is complex and there are concerns about the potential stigma of classifying a symptom as “somatic”. The aim of this review is to describe the current research on the relationship between pain and somatization in children/adolescents.

**Methods**: A search of electronic databases was conducted; articles were included if the following criteria was met: (1) Empirical research published since 1991; (2) Sample of minimum *n*=20 children/adolescents aged 0-18 years; (3) Pain, somatization, and their relationship were measured.

**Results**: Searching retrieved 3,624 articles. Pilot testing on a random sample of 200 articles resulted in 94.5% excluded due to duplication/not meeting eligibility criteria. The majority of eligible articles (73%) described the prevalence of somatic symptoms in patients with pain. Clinically significant somatization is a common complaint in pediatric pain (particularly abdominal pain), is more common in children with recurrent pain than healthy children, and is associated with worse clinical outcomes. Significant clinical pain predicts later somatic symptoms (9% of articles), and somatization is related to worse experimental pain outcomes (18% of articles). Pain is also reported in more than half of children with somatic symptom disorders (18% of articles).

**Discussion/Conclusions**: Despite concerns about the potentially stigmatizing use of the concept of somatization, it continues to be reported in pediatric pain research. The co-occurrence of pain and somatization suggests shared mechanisms, and worse outcomes are observed for youth who experience both.

#### Causes and characteristics of inappropriate referrals to a community pain clinic

Stacey Salagubang, BA(Hons), BEd, ^a^, S. Fatima Lakha, MSc, PhD, ^b^, Angela Mailis, MD and MSc, FRCPC(PhysMed), ^c^

^a^Pain and Wellness Centre, Dept. of Medicine, Div. of Physical Medicine; Toronto, Ontario, Canada; Stacey@thepwc.ca; ^b^Pain and Wellness Centre, Dept. of Medicine, Div. of Physical Medicine; Toronto, Ontario, Canada; shahfat@gmail.com; ^c^Pain and Wellness Centre, Vaughan Ontario; Dept. of Medicine, Division of Physical Medicine, UofToronto; and Toronto Rehab Institute/University Health Network, Toronto Ontario Canada, angela.mailis@uhn.ca

**CONTACT** Stacey Salagubang, BA(Hons), BEdStacey@thepwc.ca

© 2018 Stacey Salagubang, S. Fatima Lakha, and Angela Mailis. Published with license by Taylor & Francis Group, LLC.

This is an Open Access article distributed under the terms of the Creative Commons Attribution License (http://creativecommons.org/licenses/by/4.0/), which permits unrestricted use, distribution, and reproduction in any medium, provided the original work is properly cited.

**Introduction/Aim**: The study describes inappropriate referrals to an interdisciplinary non-interventional community pain clinic causing delays or rejection of referred patients and consternation to both patients and receiving facility

**Methods**: Data was collected on consecutive rejected referrals and the physicians who made them. Rejected referrals were classified as: Group A: Inadequate (lack of supportive information and/or illegible); Group B: Nothing to offer Group C: Out of scope of practice; and Group D: outside the clinic’s catchment area. Information regarding gender, university from which the MD degree was obtained, specialty, languages spoken, and years elapsed between graduation and start of practice, was collected through the publicly available administrative database of the College of Physicians and Surgeons of Ontario.

**Results**: There were 68 referrals by 62 physicians. Group A (inadequate) was by far the largest (33 rejected referrals by 31 MDs; 5 of whom had the same referral rejected twice and 2 of whom had 2 separate referrals rejected); Group B consisted of 14 referrals; Group C of 7 referrals; and Group D of 14 referrals. Some physicians were “repeat offenders” and belonged to more than one group. Group A included 22 Canadian University and 9 foreign university graduates. Rejected referrals in general accounted for 25% of all referrals to our clinic with only 1/5 of Group A referrals resubmitted with appropriate information and accepted.

**Discussion/Conclusions**: Further analysis will detail the characteristics of the physicians and whether physicians “learnt” to submit appropriate and comprehensive referrals by analysis of our database of accepted referrals.

#### Tetrahydrocannabinol (THC) Alleviates Pain in Neuropathic Morphine-Tolerant Rats

Alexandra Teggin^a^, Luca Posa^b^ and Gabriella Gobbi^c^

^a^Alexandra Teggin, McGill University, Department of Psychiatry, Montreal, Quebec, Canada, alexandra.teggin@mail.mcgill.ca; ^b^Luca Posa, Department of Psychiatry, Montreal, Quebec, Canada; Alan Edwards Centre for Research on Pain, McGill University, Montreal, QC, Canada, luca.posa@mail.mcgill.ca; ^c^Gabriella Gobbi, McGill University, Department of Psychiatry, Montreal, Quebec, Canada; Alan Edwards Centre for Research on Pain, McGill University, Montreal, QC, Canada, gabriella.gobbi@mcgill.ca

**CONTACT** Gabriella Gobbi gabriella.gobbi@mcgill.ca

© 2018 Alexandra Teggin, Luca Posa and Gabriella Gobbi. Published with license by Taylor & Francis Group, LLC.

This is an Open Access article distributed under the terms of the Creative Commons Attribution License (http://creativecommons.org/licenses/by/4.0/), which permits unrestricted use, distribution, and reproduction in any medium, provided the original work is properly cited.

**Introduction/Aim**: In 2016, 64,000 people died from opioid overdose in USA, mostly caused by opioid tolerance during pain treatments. THC has been proposed as an alternative analgesic drug to opioid tolerance, but few preclinical studies have assessed this hypothesis.

**Methods**: Wistar rats underwent sciatic nerve surgery (n=4 non-tolerant, n=8 morphine tolerant). One group was rendered morphine-tolerant (5 mg/kg, s.c. twice daily for 7 days) and another treated with vehicle. After day 7, both groups were treated with THC (2.5 mg/kg, i.p.) and then tested for mechanical allodynia. *In-vivo* electrophysiology studies were conducted on pronociceptive ON cells.

**Results**: After chronic treatment, morphine’s potency decreased by 62% (15g to 8g). The antinociceptive effect of THC on both groups reached the same maximal effect (~5.5g), but total analgesic effect was lower in tolerant compared to non-tolerant group (P<0.001). Indeed, THC decreased mechanical allodynia in tolerant rats (AUC=16.91±0.65) compared to non-tolerant rats (AUC=21.52±0.34). This suggests a cross-interaction between cannabinoid and opioid receptors.

Similarly through electrophysiology recordings, ON cells in morphine-tolerant rats showed a lower baseline firing (44%) compared to non-tolerant rats. THC reduced the firing rate in both groups, with a greater effect observed in non-tolerant rats (45.7% vs. 33.7%).

**Discussion/Conclusions**: THC has analgesic effects even in morphine-tolerant rats but to a lesser extent. Moreover, chronic treatment with morphine decreases ON cell baseline firing rate, suggesting an alteration in neuronal plasticity which affects analgesic potential. Altogether, these results suggests that THC has analgesic properties in morphine tolerance individuals, but to a smaller degree.

#### Safety and efficacy of medical cannabis in cancer pain: a retrospective analysis

Maria Fernanda Arboleda^a^, Erin Prosk^b^, Vi Dam^c^, Michael Dworkind^d^ and Antonio Vigano^e^

^a^Maria Fernanda Arboleda, MD, Supportive Care and Medical Cannabis Post-Doctoral Research Fellow, McGill University, Department of Oncology, Montreal, Quebec, Canada; ^b^Erin Prosk, MSc, Director Santé Cannabis, Montreal, Quebec, Canada; ^c^Vi Dam, RN, MSc, Clinical Research Coordinator and Research Nurse, Santé Cannabis; ^d^Michael Dworkind MD, Associate Professor Family Medicine and Palliative Care, McGill University, Medical Director and co-founder Santé Cannabis, Montreal, Quebec, Canada; ^e^Antonio Vigano, MD, MSc, Attending Physician Supportive and Palliative Care Service McGill University Health Centre, Associate Professor Departments of Oncology and Medicine, McGill University, Research Director, Santé Cannabis, Montreal, Quebec, Canada

**CONTACT** Maria Fernanda Arboleda antonio.vigano@mcgill.ca McGill University Health Centre,Associate Professor Departments of Oncology and Medicine, McGill UniversityResearch Director, Santé Cannabis, Montreal, Quebec, Canada.

© 2018 Maria Fernanda Arboleda, Erin Prosk, Vi Dam, Michael Dworkind and Antonio Vigano Published with license by Taylor & Francis Group, LLC.

This is an Open Access article distributed under the terms of the Creative Commons Attribution License (http://creativecommons.org/licenses/by/4.0/), which permits unrestricted use, distribution, and reproduction in any medium, provided the original work is properly cited.

**Introduction/Aim**: Medical cannabis (MC), though still lacking high-quality evidence, is increasingly considered as a potential therapy to complement conventional interventions for cancer pain management. This retrospective study aimed to explore the safety and effectiveness of MC in the treatment of uncontrolled cancer pain.

**Methods**: A retrospective chart review of adult cancer patients assessed at Santé Cannabis Clinic between April 2015 and July 2017 was conducted; patients who experienced cancer pain rated ≥ 4/10 on the “average pain” scale of the Brief Pain Inventory-Short Form (BPI-SF) were selected.

**Results**: Sixty patients had complete data at baseline and three months follow-up (mean age 57.3 years, 35% male). The most common types of cancer were: breast (20%), prostate (13%), lung (8%) and pancreas (6%). A combination of oral and inhaled MC (43.3%) along with THC/CBD 1:1 ratio products (32%) were most frequently prescribed. BPI scores for “average pain”, “worst pain” and “pain interference” significantly improved (Wilcoxon signed-rank test, p<0.0001). The mean relative difference for “average pain” was 29%, (95% CI: 20%-38%), for “worst pain” was 24%, (15-33) and for “pain interference” was 29%, (17-41). Overall, 16 patients (26.7%) experienced side effects, all classified as mild or moderate, including drowsiness, dizziness and dry mouth.

**Discussion/Conclusions**:MC therapy, supervised through a specialized clinic, was found effective and safe for relieving cancer pain not adequately controlled by conventional treatments. Further studies with bigger samples and randomized, placebo controlled designs are necessary to confirm these preliminary data.

#### Identifying effective coping strategies and associated personality characteristics for chronic pain through a retrospective data analysis

Eleni G. Hapidou^a^, Shelley Zhu^b^ and Cindy Li^c^

^a^Hamilton Health Sciences, Psychiatry and Behavioral Neurosciences & Michael G. DeGroote Pain Clinic, McMaster University, Hamilton, ON, Canada; ^b^Psychology Neuroscience and Behavior Mental Health Specialization, McMaster University, Hamilton, ON, Canada; ^c^Psychology Neuroscience and Behavior, McMaster University, Hamilton, ON, Canada

**CONTACT** Eleni G. Hapidou hapidou@hhsc.ca

© 2018 Eleni G. Hapidou, Shelley Zhu and Cindy Li. Published with license by Taylor & Francis Group, LLC.

This is an Open Access article distributed under the terms of the Creative Commons Attribution License (http://creativecommons.org/licenses/by/4.0/), which permits unrestricted use, distribution, and reproduction in any medium, provided the original work is properly cited.

**Introduction/Aim**: The purpose of this study was to predict changes in coping strategy use as measured by the Chronic Pain Coping Inventory (CPCI) at discharge from a four-week inter-disciplinary chronic pain management program from the clinical scales of the Minnesota Multiphasic Personality Inventory-2 (MMPI-2). Participants (n = 461; 216 males) were patients with heterogeneous chronic pain referred to the program by insurance companies, the workers’ compensation board, and Veterans Affairs. Patients completed a set of questionnaires that assess various pain–related variables at admission and discharge including the CPCI and the MMPI-2.

**Methods**: Changes in coping strategy use was determined by CPCI difference scores between admission and discharge. Paired t-tests were conducted to investigate the differences between each subscale of the CPCI. Multiple regression was then used to predict changes in significant CPCI subscale difference scores with the clinical scales of the MMPI-2.

**Results**: Significant differences between admission and discharge scores were found for all of the CPCI subscales (p < 0.001) except Resting (p > 0.05). The largest differences were obtained for Relaxation, Exercise/Stretch and Pacing. Clinical scales 1 and 6 of the MMPI-2 were predictive of the changes in the CPCI subscale Relaxation scores. Changes in CPCI Relaxation use were significantly predicted by Hs (Scale 1) scores (Beta = –0.182, p < 0.05) as well as the Pa (Scale 6) scores (Beta = –0.110, p < 0.05).

**Discussion/Conclusions**: Results showed that patient scores on the MMPI-2 clinical Scales 1 and 6 are significant predictors of improvements in the coping strategy of Relaxation at discharge. This means patients who score lower on these scales are more likely to increase their use of Relaxation as a coping strategy following the four-week interdisciplinary chronic pain management program. These results are discussed within the context of personality characteristics and coping strategies in chronic pain. Further analysis remains to explore gender differences and MMPI-2 clusters.

#### Pain-related activity management patterns as predictors of treatment outcomes in patients with fibromyalgia syndrome

Mélanie Racine, PhD., ^a^, Elisabet Sánchez-Rodríguez, PhD., ^b,c,d^, Rocío De La Vega, PhD., ^b,e^, Santiago Galán, MSc., ^b,c,d^, Ester Solé, PhD., ^b,c,d^, Mark P. Jensen, PhD., ^e^, Jordi Mirò, PhD., ^b,c,d^, Dwight E. Moulin, MD., ^a^ and Warren R. Nielson, PhD., ^f^

^a^Department of Clinical Neurological Sciences, Schulich School of Medicine & Dentistry, Western University, London, ON, Canada; ^b^Unit for the Study and Treatment of Pain – ALGOS, Universitat Rovira i Virgili, Catalonia, Spain; ^c^Research Center for Behavior Assessment (CRAMC), Department of Psychology, Universitat Rovira i Virgili, Catalonia, Spain; ^d^Institut d’Investigació Sanitària Pere Virgili, Universitat Rovira i Virgili, Catalonia, Spain; ^e^Department of Rehabilitation Medicine, University of Washington, Seattle, WA, USA; ^f^Department of Psychology, Western University and Lawson Health Research Institute, London, ON, Canada

**CONTACT** Mélanie Racine research@melanieracine.com

© 2018 Mélanie Racine, Elisabet Sánchez-Rodríguez, Rocío De La Vega, Santiago Galán, Ester Solé, Mark P. Jensen, Jordi Mirò, Dwight E. Moulin and Warren R. Nielson. Published with license by Taylor & Francis Group, LLC.

This is an Open Access article distributed under the terms of the Creative Commons Attribution License (http://creativecommons.org/licenses/by/4.0/), which permits unrestricted use, distribution, and reproduction in any medium, provided the original work is properly cited.

**Introduction/Aim**: This study sought to determine if pre- to post-treatment changes in pain-related activity patterns (i. e., overdoing, avoidant, pacing) are associated with pre- to post-treatment changes in function (i. e., pain interference, psychological function and physical function) in patients with fibromyalgia syndrome who participated in either an operant learning or an energy conservation based training in activity management.

**Methods**: Sixty-nine patients with fibromyalgia syndrome participated in an activity management treatment (32 in an operant learning group and 37 in an energy conservation group). Outcomes were assessed at pre- and post-treatment, and patients provided demographic information and completed measures assessing pain intensity, pain interference, psychological function, physical function, and pain management activity patterns. Three linear hierarchical regression analyses predicting changes in pain outcomes from changes in pacing, overdoing and avoidant activity patterns were performed.

**Results**: Changes in pain-related activity patterns made significant contributions to the prediction of changes in patients’ function. Specifically: (a) increases in overdoing predicted reductions in pain interference; (b) decreases in avoidance predicted improvements in psychological function; and (c) increases in pacing predicted improvements in physical function.

**Discussion/Conclusions**: This study provides support for a role of activity management treatment in improved adjustment to chronic pain. Research is needed to replicate and extend these findings, in order to build an empirical basis for developing more effective chronic pain treatments for facilitating improved physical and psychological function in individuals with chronic pain.

#### Prevalence of pain and analgesic adequacy in hospitalized adults in a University affiliated hospital in Sao Paulo, Brazil

Marina G. Salvetti^a^, Paulo C. Garcia^b^, Lisabelle M. Rossato^c^ and Mariana Bueno^d^

^a^Nursing Department, School of Nursing, University of São Paulo, São Paulo, SP, Brazil; ^b^Nursing Department, University Hospital, University of São Paulo, São Paulo, Brazil; ^c^Child and Maternal Department, School of Nursing, University of Sao Paulo, Sao Paulo, SP, Brazil; ^d^Child Health Evaluative Sciences, The Hospital for Sick Children, Toronto, ON, Canada

**CONTACT** Marina G. Salvetti mgsalvetti@usp.br Av. DR. Eneas de Carvalho Aguiar, 419, Cerqueira Cesar, São Paulo, SP

© 2018 Marina G. Salvetti, Paulo C. Garcia, Lisabelle M. Rossato and Mariana Bueno. Published with license by Taylor & Francis Group, LLC.

This is an Open Access article distributed under the terms of the Creative Commons Attribution License (http://creativecommons.org/licenses/by/4.0/), which permits unrestricted use, distribution, and reproduction in any medium, provided the original work is properly cited.

**Introduction/Aim**: Our aims were to identify the prevalence and the impact of pain in hospitalized adults, and to analyze analgesic adequacy.

**Methods**: This cross-sectional study was conducted at the University Hospital of the University of Sao Paulo, Brazil. Data collection was performed by trained research assistants in two time-points of 24-hour period each, with a 15-day interval. All adults and elderly patients hospitalized in six units during data collection period were included. Data were obtained from medical charts and from a structured interview at the bedside. The research project was approved by local ethics committee.

**Results**: 134 patients were included, mean age 52 (±19.4) years, male (56.7%), 53.4% received clinical medicine care, 25.9% general surgery. During the interview, pain was referred by 27.6% patients. In the 24 hours prior to the interview, 43.3% reported pain. Mean pain score was 6.6 (±2.4), assessed with the numerical verbal rating scale. Moderate pain (5 to 7) was observed in 34.8% of the patients and severe pain (8 to 10) in 42.0%. Pain affected patients’ mobility in bed (61.2%), sleep (56.7%) and walk (52.2%). Half of the prescriptions (50%) were not adequate for pain intensity.

**Discussion/Conclusions**: High prevalence of pain was identified in hospitalized adults (27.3% to 43.3%). Patients reported moderate to severe pain that affected the mobility in bed, sleep and walk. Pain control was inadequate. Results confirm negative impact of pain on recovery and self-care of patients and reinforce the need of implementation of guidelines and policies for pain management.

#### Comparing health anxious beliefs in individuals with and without chronic or recurring pain

G. S. Rachor^a^ and A. M. Penney^a^

MacEwan University, Department of Psychology, Edmonton, Alberta, Canada

**CONTACT** G. S. Rachor rachorg2@macewan.ca

© 2018 G. S. Rachor and A. M. Penney. Published with license by Taylor & Francis Group, LLC.

This is an Open Access article distributed under the terms of the Creative Commons Attribution License (http://creativecommons.org/licenses/by/4.0/), which permits unrestricted use, distribution, and reproduction in any medium, provided the original work is properly cited.

**Introduction/Aim**: The occurrence of health anxiety (HA) in chronic pain is associated with increased disability, and often results in poor prognosis. HA is characterized by increased awareness to somatic sensations, as well as increased illness perceptions, such as perceived likelihood of becoming ill, and perceived severity of illness. Although researchers have identified somatic awareness and illness perceptions to also predict outcomes in chronic pain, few studies have examined these factors from a broader HA perspective.

**Methods**: University students who self-reported as either experiencing chronic or recurring pain (*n *= 191), or no-pain (*n *= 173), completed the Chronic Pain Grade Scale and Short Health Anxiety Inventory (SHAI). Independent samples *t*-tests were conducted to determine whether the pain and non-pain samples significantly differed on scores of body vigilance, perceived likelihood of illness, and perceived illness severity, as measured by the SHAI.

**Results**: Results indicated that the pain sample scored significantly higher than the non-pain sample on measures of body vigilance, *t* (361) = 9.50, *p *< .001, and perceived likelihood of illness, *t* (360) = 5.34, *p *< .001, but not on perceived illness severity, *t* (361) = −0.70, *p *= .48.

**Discussion/Conclusions**: The current findings suggest that in comparison to individuals without chronic pain, individuals with chronic pain are more likely to report health anxious beliefs about somatic sensations and perceive a greater likelihood of contracting illness. These results further support the notion that HA in chronic pain reflects broader health concerns that extend beyond the underlying physical pathology of the pain condition.

#### Quebec serve and protect low back pain study: what about mental quality of life?

Nabiha Benyamina Douma^a^, Charles Côté^a^ and Anaïs Lacasse^a^

Département des sciences de la santé, Université du Québec en Abitibi-Témiscamingue, Rouyn-Noranda, Canada

**CONTACT** Nabiha Benyamina Douma benyamin@uqat.ca

© 2018 Nabiha Benyamina Douma, Charles Côté, and Anaïs Lacasse. Published with license by Taylor & Francis Group, LLC.

This is an Open Access article distributed under the terms of the Creative Commons Attribution License (http://creativecommons.org/licenses/by/4.0/), which permits unrestricted use, distribution, and reproduction in any medium, provided the original work is properly cited.

**Introduction/Aim**: As of now, the impact of low back pain (LBP) and its chronic state (CLBP) on mental health-related quality of life (HRQOL) have never been investigated among police officers. The present investigation aims at studying this relationship using a biopsychosocial model.

**Methods**: Between May and October 2014, a web-based cross-sectional study was conducted among Quebec police officers (Canada). Mental HRQOL was measured using the Role Emotional (RE) and the Mental Health (MH) domains of the SF-12v2 Health Survey. The impact of CLBP on mental HRQOL (as opposed to acute/subacute LBP or no LBP) was studied with a multivariate linear regression model.

**Results**: Of the 3589 police officers who participated in the study, 1013 (28.4%) reported CLBP. The mean age of respondents was 38.5 ± 8.7 years and 32.0% were females. The RE (44.1/100) and MH (49.0/100) mean scores of the CLBP group were comparable to the scores found in populations suffering from cancer or heart diseases. Compared to officers without LBP, the presence of CLBP was significantly associated with lower RE (β: −0.068; p = 0.003) and MH (β: −0.062; p = 0.002) scores. These relationships were not found in the acute/subacute LBP group.

**Discussion/Conclusions**: Our results underscore how frequent CLBP is among police officers and how burdensome it is. Considering the importance of good physical and mental health for this occupational population, police organizations should be aware of this issue and contribute to the efforts towards CLBP prevention and management in the workplace.

#### Using wechat to disseminate effective pain treatments for newborn blood sampling in China

Jiale Hu^a^, Feng Xue^b^, Nanping Shen^c^, Leilei Yu^d^ and Denise Harrison^a^

^a^School of Nursing & Research Institute, University of Ottawa and Children’s Hospital of Eastern Ontario, Ottawa, Ontario, Canada; ^b^Shanghai Children’s Medical Center, NICU, Shanghai, China; ^c^Nursing Administration Department, Shanghai Children’s Medical Center, Shanghai, China; ^d^Nursing Administration Department, Shanghai Ninth People’s Hospital, Shanghai, China

**CONTACT** Jiale Hu jhu081@uottawa.ca

© 2018 Jiale (Gary) Hu, Feng Xue, Nanping Shen, Leilei Yu and Denise Harrison. Published with license by Taylor & Francis Group, LLC.

This is an Open Access article distributed under the terms of the Creative Commons Attribution License (http://creativecommons.org/licenses/by/4.0/), which permits unrestricted use, distribution, and reproduction in any medium, provided the original work is properly cited.

**Introduction/Aim**: To disseminate knowledge about the evidence based pain management strategies of breast-feeding, skin-to-skin care and sucrose for newborns during blood work, Harrison et al developed and posted a BSweet2Babies video on YouTube in multiple languages including Chinese. However, in China, YouTube is not accessible. This study aimed to disseminate the Chinese BSweet2Babies video through WeChat, the most popular social media in China, and evaluate the reach and acceptability.

**Methods**: A WeChat blog was developed, which included the BSweet2Babies video and a survey link. A comprehensive dissemination plan was implemented to maximize views during one-year period (SEP15, 2017 to SEP14, 2018).

**Results**: After the first 2.5-month posting, the video had 10,045 views, 166 “likes” and 215 completed surveys. Eighty respondents were parents (37.2%). Over half of respondents (60.9%) did not know any of the pain management strategies. However, after viewing the video, most viewers, who were not previously aware of the pain management strategies, intended to use or advocate for breastfeeding (71.6%), skin-to-skin care (82.2%), and sucrose (80.3%). Nearly all respondents would recommend the video and found the video was helpful and easy to understand. Around 40% respondents felt the video was too long and 10% felt the strategies were not easy to apply in real life.

**Discussion/Conclusions**: WeChat is a potentially effective approach to disseminate evidence to parents and health care professionals in China. However, response rates to the survey are low, warranting furtherwork on maximizing viewer response rates to the study.

#### Attitudes and practices regarding disability certification: a survey of Canadian family physicians

Jason W. Busse^a^, Zain Izhar^b^, Jagpreet Kaler^c^, Samantha Craigie^a^, Abishek Hariharan^d^, Bilal Maqbool^e^, Hassan Ali Al Saleh^e^, Priya Gupta^e^, John Riva^f^, Jaynendr Jadoo^g^, Saeha Shin^e^, Arnav Agarwal^h^, Brian Hong^i^, Leyla Eryuzlu^h^, Sheniz Eryuzlu^j^, Chris J. Hong^k^ and Sohail Mulla^l^

^a^Anesthesia, McMaster University, Hamilton, Canada; ^b^Kansas City University of Medicine and Biosciences, Kansas City, Missouri, USA; ^c^Medicine, Queens University, Kingston, Canada; ^d^Medicine, Ross University, Iselin, New Jersey, USA; ^e^Health Sciences, McMaster University, Hamilton, Canada; ^f^Family Medicine, McMaster University, Hamilton, Canada; ^g^Biology, McMaster University, Hamilton, Canada; ^h^Medicine, University of Toronto, Toronto, Canada; ^i^Medicine, University of Ottawa, Ottawa, Canada; ^j^Medicine, McMaster University, Hamilton, Canada; ^k^Otolaryngology-Head & Neck Surgery, University of Toronto, Toronto, Canada; ^l^Health Research Methods, Evidence, and Impact, McMaster University, Hamilton, Canada

**CONTACT** Jason Busse bussejw@mcmaster.ca

© 2018 Jason Bussea, Zain Izharb, Jagpreet Kalerc, Samantha Craigiea, Abishek Hariharand, Bilal Maqboole, Hassan Ali Al Salehe, Priya Guptae, John Rivaf, Jaynendr Jadoog, Saeha Shine, Arnav Agarwalh, Brian Hongi, Leyla Eryuzluh, Sheniz Eryuzluj, Chris J. Hongk and Sohail Mulla. Published with license by Taylor & Francis Group, LLC.

This is an Open Access article distributed under the terms of the Creative Commons Attribution License (http://creativecommons.org/licenses/by/4.0/), which permits unrestricted use, distribution, and reproduction in any medium, provided the original work is properly cited.

**Intro/aim**: Employers and disability insurers require documentation that an employee is medically unable to work, which is often provided by family physicians. Medical training, however, does not provide instruction in disability assessment. We administered two surveys to Canadian family physicians to determine knowledge and attitudes, and approaches toward disability certification.

**Methods**: We administered 3,000 surveys via fax between May-December 2014. Responses were analyzed descriptively. We used a multivariable model to assess factors associated with physicians’ self-rated proficiency at completing disability certification.

**Results**: 276 family physicians responded. Most (94%) felt that medical training should include disability certification, but only 40% believed their own training was adequate. Most (79%) considered disability certification an unwelcome obligation, and 75% expressed difficulty acting as a neutral examiner. Many respondents (65%) felt relationships with patients would suffer if they did not support disability certification, with nearly half endorsing that patient’s views determined their recommendation in cases of uncertainty. In an adjusted model, only comfort with assessing non-credible symptoms was significantly associated with self-perceived proficiency at disability certification (OR 0.45, 95% CI 0.26–0.76).

**Discussion/Conclusions**: Most family physicians report discomfort and insufficient training to perform disability assessments, and conclusions may often reflect patient’s beliefs rather than objective findings.

#### The effects of fibromyalgia on the working experience

Karim Mukhida^a^ and Wendy Carroll^b^

^a^Anesthesiology, Pain Management and Perioperative Medicine, Dalhousie University, Halifax, Canada; ^b^Saint Mary’s University, Sobey School of Business, Halifax, Canada

**CONTACT** Karim Mukhida kmukhida@dal.ca

© 2018 Karim Mukhida and Wendy Carroll. Published with license by Taylor & Francis Group, LLC.

This is an Open Access article distributed under the terms of the Creative Commons Attribution License (http://creativecommons.org/licenses/by/4.0/), which permits unrestricted use, distribution, and reproduction in any medium, provided the original work is properly cited.

**Introduction/Aim**: Chronic pain conditions, such as fibromyalgia, adversely affect individuals’ abilities to work. The aim of this study was to examine, from the perspective of patients, the effects that fibromyalgia symptoms had on their ability to work, the challenges that they encountered in the workplace that did not foster their continued employment, the types of modifications to their work or workplace that they thought would facilitate their productivity and ability to work, and the role that health care providers can play in aiding patients with their employment goals.

**Methods**: A systematic review of the qualitative literature regarding fibromyalgia and work was undertaken using the PubMed and EBSCOhost databases from 1982 to 2017. A descriptive review of the articles’ content was conducted to identify themes from the findings of the studies.

**Results**: A variety of themes emerged from the review including: i) the work experience being a challenging one with which to cope; ii) relationships being strained at work; iii) repercussions of fibromyalgia on subjects’ attitudes towards work and its relation to life outside of work; and iv) subjects’ perspectives on the possible solutions to helping them better cope with fibromyalgia and work.

**Discussion/Conclusions**: Based upon these findings we highlight strategies that potentially could foster continued employment of patients with fibromyalgia and the ways in which health care providers can support patients’ employment goals by collaborating with patients and their employers, dispelling stigma regarding the illness, and providing practical and specific advice regarding workplace accommodations.

#### Chronic opioid toxicity as a clinically important concept. A narrative literature review

Irina Kudrina^a^, Yoram Shir^b^, Gillian Bartlett^a^ and Leon Tourian^c^

^a^Department of Family Medicine, McGill University, Montreal, Canada; ^b^The Alan Edwards Pain Management Unit, McGill University, Montreal, Canada; ^c^The Alan Edwards Pain Management Unit, Department of Psychiatry, McGill University, Montreal, Canada

**CONTACT** Irina Kudrina irina.kudrina@gmail.com

© 2018 Irina Kudrina, Yoram Shir, Gillian Bartlett and Leon Tourian. Published with license by Taylor & Francis Group, LLC.

This is an Open Access article distributed under the terms of the Creative Commons Attribution License (http://creativecommons.org/licenses/by/4.0/), which permits unrestricted use, distribution, and reproduction in any medium, provided the original work is properly cited.

**Introduction/Aim**: Clinicians encounter long-term opioid use (LTOU), ≥ 1 month, in pain and addiction management. Similarly to the chronic acetaminophen toxicity, we propose a concept of *chronic opioid toxicity (COT)*. Due to the abundance of opioid receptors in humans, *COT* may result in multitude of reversible and irreversible effects. A vast majority of clinicians have poor understanding of the scope of *COT*. A consensus among clinicians on *COT* screening and management is lacking.

**Objectives**: 1) To describe the scope of the clinically relevant *COT* symptoms and signs. 2) Briefly discuss suggested clinical approach to *COT* screening and management.

**Methods**: Narrative literature review. Databases: Ovid MEDLINE(R), EMBASE, PsychInfo, Cochrane and Google Scholar. Empirical studies and systematic reviews looking at LTOU-induced toxic effects in humans, screening and/or management and/or reversibility of *COT* effects. Due to a high degree of heterogeneity of data, we grouped results by organ system and summarized narratively.

**Results**: *COT* is common and manifests by one or more partially/fully irreversible organ systems dysfunctions, including those of neuroendocrine, gastrointestinal, respiratory, cardiovascular, musculoskeletal, skin and immune organ systems. Hormone replacement therapy for opioid-induced androgen deficiency is an example of *COT* effects management. Examples of irreversible *COT* effects include advanced neuroendocrine changes, addiction and death.

**Discussion/Conclusions**: The concept of *COT* is important to assure high quality patient care. Clinicians should discuss LTOU risks and benefits, and obtain an explicit consent from each patient for whom LTOU is considered. Screening for hormonal baseline abnormalities and *COT*-induced endocrinopathy should become a standard of care.

#### The role of parental minimizing and monitoring responses in pediatric chronic pain outcomes in youth

Caroline Summers^a^, Soeun Lee^b^, Nez Elik^c^ and C. Meghan McMurtry^d^

^a^Psychology, Neuroscience and Behaviour, McMaster University, Hamilton, ON, Canada; ^b^Psychology, McMaster Children's Hospital, University of Guelph, Hamilton, ON, Canada; ^c^Psychiatry and Behavioural Neuroscience, McMaster Children’s Hospital, McMaster University, Hamilton, ON, Canada; ^d^Psychology, McMaster Children’s Hospital, University of Guelph, Guelph, ON, Canada

**CONTACT** Caroline Summers summerca@mcmaster.ca

© 2018 Caroline Summers, Soeun Lee, Nez Elik and C. Meghan McMurtry. Published with license by Taylor & Francis Group, LLC.

This is an Open Access article distributed under the terms of the Creative Commons Attribution License (http://creativecommons.org/licenses/by/4.0/), which permits unrestricted use, distribution, and reproduction in any medium, provided the original work is properly cited.

**Introduction/Aim**: Pediatric chronic pain impacts 11–38% of youth, resulting in a lower quality of life. Parental responses relate to how children experience pain, however, the role of parental minimizing and monitoring behaviours in youth outcomes is unclear. Parental psychological flexibility, and youth pain acceptance and self-efficacy are associated with positive outcomes in youth. This study examined the relationship between parental responses (i. e., minimizing, monitoring) and youth pain acceptance and self-efficacy, and whether parental minimizing and monitoring behaviours mediate the relationship between parental psychological flexibility and youth quality of life.

**Methods**: Youth (8–17 years) and their parents completed questionnaires during their intake assessment at the Pediatric Chronic Pain Program at McMaster Children’s Hospital. Youth completed questionnaires on their pain intensity, pain self-efficacy, pain acceptance, and quality of life. Parents completed questionnaires on their psychological flexibility and responses to their youth’s pain.

**Results**: Currently, data from 80 parent-youth dyads have been obtained; data collection is ongoing to achieve sufficient power to obtain medium effects (n = 85). It is expected that there will be significant positive correlations among parental minimizing, monitoring, and youth pain intensity, and significant negative correlations among parental psychological flexibility, and youth pain acceptance, pain self-efficacy, and quality of life. Finally, parental minimizing and monitoring will partially mediate the relationship between parental psychological flexibility and youth quality of life.

**Discussion/Conclusions**: Current results will increase our understanding of how parental minimizing and monitoring responses relate to their youth’s pain and mediate the impact of parental psychological flexibility on youth quality of life.

#### Selective melatonin MT2 receptor ligands relieve neuropathic pain through modulation of brainstem descending antinociceptive pathways and opioid interactions.

L. Posa^a,b^, M. Lopez-Canul^a^, S. Comai^c^, S. Boccella^d^, S. Maione^d^, V. Granados Soto^e^ and G. Gobbi^a,b^

^a^Psychiatry, McGill University, Montreal, Canada; ^b^Alan Edwards Centre for Research on Pain, McGill University, Montreal, Canada; ^c^Neuroscience, Vita-Salute University, Milano, Italy; ^d^Experimental Medicine, Seconda Università di Napoli, Napoli, Italy; ^e^Pharmacobiology, Centro de Investigación y de Estudios Avanzados del I.P.N, Mexico City, Mexico

**CONTACT** L. Posa luca.posa@mail.mcgill.ca

© 2018 L. Posa, M. Lopez-Canul, S. Comai, S. Boccella, S. Maione, V. Granados Soto and G. Gobbi. Published with license by Taylor & Francis Group, LLC.

This is an Open Access article distributed under the terms of the Creative Commons Attribution License (http://creativecommons.org/licenses/by/4.0/), which permits unrestricted use, distribution, and reproduction in any medium, provided the original work is properly cited.

**Introduction/Aim**: Neuropathic pain is an important health problem for which only a few treatments are available. Preclinical studies have shown that melatonin (MLT) and its related MT2 selective agonists have analgesic properties, likely through opioid (OPr) receptors. Here, we determined the effects of the selective MT2 receptor partial agonist (UCM924) in two rat neuropathic pain models and examined its supraspinal mechanism of action.

**Methods**: Rat L5–L6 spinal nerve ligation (SNL) and spared nerve injury (SNI) models were used to evaluate neuropathic allodynia and in-vivo electrophysiological recordings of ON and OFF cells in the periaqueductal grey-rostral ventral medulla projection were collected to determine the mechanism of action.

**Results**: In both neuropathic models, UCM924 (20–40 mg/kg) produced a prolonged antinociceptive effect that is: dose-dependent, superior to a high dose of MLT (150 mg/kg) and comparable with gabapentin (100 mg/kg), but without motor coordination impairments. Using in-vivo electrophysiology combined with tail-flick, we observed that microinjection of UCM924 into the ventrolateral periaqueductal gray decreased the tail-flick response, depressed the firing activity of ON cells, and activated the firing of OFF cells. Importantly, non-selective (naloxone) and selective mu-OPr antagonist (CTOP), but not selective delta-OPr antagonist (naltrindole) (1 mg/kg) blocked the antinociceptive effects of UCM924 in the SNI model and its effect on ON cell and OFF cells.

**Discussion/Conclusions**: Altogether, these data demonstrate that selective MT2 receptor partial agonists have analgesic properties through modulation of brainstem descending antinociceptive pathways and this effect is mediated by mu-OPr. MT2 receptors may represent a novel target in the treatment of neuropathic pain.

#### Non-opioid options for managing pain: what does the evidence say?

Janice Mann^a^ and Sirjana Pant^b^

^a^CADTH Opioid Working Group and Knowledge Mobilization, Ottawa, ON, Canada; ^b^Consultant to the CADTH Opioid Working Group, CADTH, Ottawa, ON, Canada

**CONTACT** Janice Mann janicem@cadth.ca

© 2018 Janice Mann and Sirjana Pant. Published with license by Taylor & Francis Group, LLC.

This is an Open Access article distributed under the terms of the Creative Commons Attribution License (http://creativecommons.org/licenses/by/4.0/), which permits unrestricted use, distribution, and reproduction in any medium, provided the original work is properly cited.

**Introduction/Aim**: Guidelines recommend optimizing non-opioid alternatives for pain management, before a trial of opioids. However, these guidelines do not provide evidence on the effectiveness of non-opioid alternatives, and this leaves a gap for those attempting to put the recommendation into practice. This poster will present the evidence synthesized on different non-opioid and non-pharmacological treatment options for the management of pain.

**Methods**: In collaboration with partners including clinicians and policymakers, we identified non-opioid treatment options that could potentially be effective in managing pain. For each, we conducted a rapid evidence review in which a limited literature search was conducted of key resources, and titles and abstracts of the retrieved publications were reviewed. Full-text publications were evaluated for final article selection according to predetermined selection criteria (population, intervention, comparator, outcomes, and study designs). Final reports were posted on the CADTH website.

**Results**: CADTH has produced over 20 Rapid Response reports that synthesize and appraise evidence on non-opioid alternatives in the management of pain, both acute and chronic. We are also developing environmental scan reports on the availability and access to non-pharmacological treatments for pain across Canada, and on emerging non-opioid drugs for pain. Clinical and policy tools based on the reviews are being developed.

**Discussion/Conclusions**: To follow guideline recommendations to optimize non-opioid alternatives for pain before a trial of opioids, it is crucial to know what the evidence says. Putting the evidence into policy and practice can potentially reduce the inappropriate use of opioids and help to address Canada’s opioid crisis.

#### Does culture influence pain-related parent-behaviors?

Ólöf Kristjánsdóttir^a^, Patrick J. McGrath^b^, Sean P. MacKinnon^c^, Guðrún Kristjánsdóttir^a^, Pusluk Siripul^d^, G. Allen Finely^e^ and Yoko Yoshida^c^

^a^Faculty of Nursing, University of Iceland, Reykjavík, Iceland; ^b^Centre for Research in Family Health, IWK Health Centre, Halifax, Nova Scotia, Canada; ^c^Psychology, Dalhousie University, Halifax, Nova Scotia, Canada; ^d^Faculty of Nursing, Khon-Kaen University, Khon-Kaen, Thailand; ^e^Anesthesia & Psychology, IWK Health Centre, Halifax, Nova-Scotia, Canada

**CONTACT** Ólöf Kristjánsdóttir Olof.Kristjansdottir@Dal.Ca

© 2018 Ólöf Kristjánsdóttir, Patrick McGrath, Sean MacKinnon, Guðrún Kristjánsdóttir, Pusluk Siripul, Allen Finely and Yoko Yoshida. Published with license by Taylor & Francis Group, LLC.

This is an Open Access article distributed under the terms of the Creative Commons Attribution License (http://creativecommons.org/licenses/by/4.0/), which permits unrestricted use, distribution, and reproduction in any medium, provided the original work is properly cited.

**Introduction/Aim**: Studies suggest that cultural models of parenting (CMP) influence parental behaviors. Predominant cultural values are believed to inform the parenting styles caregivers adopt. Cultural values were expected to affect parental behaviors indirectly through parenting styles. We believed this would be moderated by ecosocial context. The present study aimed to examine cultural influences on pain-related parent-behaviors (PRPB). We hypothesized that ecosocial context would moderate the relationship between cultural values, parenting styles, and PRPB; and parenting styles would mediate the effect of cultural values on PRPB.

**Methods**: A cross-cultural survey design was employed using a convenience sample of 547 caregivers of 6–12-year-olds living in Canada (*n* = 183), Iceland (*n *= 184), and Thailand (*n *= 180).

The individualism-collectivism scale measured vertical and horizontal individualism, and collectivism. The parenting styles and dimensions questionnaire measured authoritative, and authoritarian parenting styles. The inventory of parent/caregiver responses to the children’s pain experience scale measured solicitousness and discouraging.

**Results**: Multigroup structural equation modeling, showed that country did not affect which CMP caregivers adopted. Parenting styles mediated the relationship between cultural values and PRPB. Vertical/horizontal individualism, collectivism, and authoritative and authoritarian-parenting styles positively predicted solicitousness. Vertical individualism and authoritarian-parenting style positively predicted discouraging, whereas other predictors did not.

**Discussion/Conclusions**: Unexpectedly, ecosocial context did not influence which CMP caregivers adopt, including their PRPB. As expected, parenting styles were mediators. Results supports others’ claims of solicitousness universality in a pediatric pain context. However, solicitousness may have different cultural meanings among individuals, and may be used in conjunction with discouraging.

#### Self-management perceived ability and quality of life in adults with early to late stage knee osteoarthritis

Alix Cagnin^a^, Manon Choinière^b^, Nathalie J. Bureau^c^, Madeleine Durand^d^, Laetitia De Polo^a^ and Nicola Hagemeister^a^

^a^Laboratoire d’Imagerie et d’Orthopédie (LIO), École de Technologie Supérieure (ÉTS), Montréal, Québec, Canada; ^b^Anesthésiologie, Université de Montréal (UdeM), Montréal, Québec, Canada; ^c^Radiologie, radio-oncologie et médecine nucléaire, Université de Montréal (UdeM), Montréal, Québec, Canada; ^d^Médecine interne, Université de Montréal (UdeM), Montréal, Québec, Canada

**CONTACT** Alix Cagnin alix.cagnin.1@ens.etsmtl.ca

© 2018 École de Technologie Supérieure de Montréal. Published with license by Taylor & Francis Group, LLC.

This is an Open Access article distributed under the terms of the Creative Commons Attribution License (http://creativecommons.org/licenses/by/4.0/), which permits unrestricted use, distribution, and reproduction in any medium, provided the original work is properly cited.

**Introduction/Aim**: The aim of this study was to investigate the associations between self-management perceived ability, functional status and socioeconomic characteristics in patients with knee osteoarthritis (OA).

**Methods**: Patients were included if they rated their worst pain in the past 7 days ≥ 4 on a 0–10 pain intensity scale and if they had a Kellgren-Lawrence (KL) OA severity grade ≥ 2 out of 4 on radiographs. The patients’ self-management perceived competency was measured with the Partners in Health Scale (PIHS). The Knee Injury and Osteoarthritis Outcome Score (KOOS) was also used to assess knee function.

**Results**: 543 patients from 76 clinics participated. 65.2% were women, the mean age was 63.5 years (standard deviation SD: 9.3). Mean scores on the PIHS subscales were the following: knowledge of their treatment (PK: 80.5/100; SD: 19.0), adherence (PA: 87.5; 13.2) and symptoms management (PSM: 76.2; 20.5). Women had higher PK scores than men (82.5 vs 76.7, p < 0.001) and better PA scores (88.6 vs 85.4, p = 0.008). Patients who completed a college or university degree had greater PK score compared with those who finished their studies after secondary school (83.5 vs 77.7; p = 0.027). PSM scores were moderately correlated with all KOOS subscales, but mostly with the quality of life subscale (r = 0.45; all p < 0.001). PK scores correlation coefficients remained small and there was no significant correlation with the PA scores.

**Discussion/Conclusions**: Lower quality of life is associated with lower perceived symptom management ability. Patients may overestimate their adherence so there is still room for empowering them to take an active role in the management of their condition.

#### Postoperative chronic pain and opioid use after major pediatric orthopedic surgery

Brittany N. Rosenbloom^a^, Lisa Isaac^b^, Fiona Campbell^b^, Gabrielle Pagé^c^, Shima Razavi^b^, Meghan Rossi^b^, Jennifer Stinson^b,d^ and Joel Katz^a^

^a^Psychology, York University, Toronto, Canada; ^b^Anesthesia and Pain Medicine, The Hospital for Sick Children, University of Toronto, Toronto, Canada; ^c^Centre de recherche du Centre hospitalier de l’Université de Montréal, Montreal, Canada; ^d^Lawrence S. Bloomberg, Faculty of Nursing, University of Toronto, Toronto, Canada

**CONTACT** Brittany Rosenbloom bnrosen@yorku.ca

© 2018 Brittany N. Rosenbloom, Lisa Isaac, Fiona Campbell, Gabrielle Pagé, Shima Razavi, Meghan Rossi, Jennifer Stinson and Joel Katz. Published with license by Taylor & Francis Group, LLC.

This is an Open Access article distributed under the terms of the Creative Commons Attribution License (http://creativecommons.org/licenses/by/4.0/), which permits unrestricted use, distribution, and reproduction in any medium, provided the original work is properly cited.

**Aims**: In-hospital opioid use after major pediatric surgeries, such as posterior spinal fusion for scoliosis, tends to be higher than other procedures. The incidence of long-term opioid use has rarely been investigated among the pediatric population and it is not clear if the high in-hospital rates continue. The study aims are to examine the incidence of (1) chronic post-surgical pain (CPSP) and (2) prescription opioid use for post-operative pain management 6- and 12-months after major pediatric surgery.

**Methods**: A prospective, longitudinal study was used to follow 265 patients (58.49% male, mean age = 14.05 years, SD = 2.51) and their parents/guardians prior to surgery (T0, n = 238), during hospitalization (T1, n = 265), and at 6- (T2, n = 215) and 12-months (T3, n = 226) post-surgery. Data were collected by questionnaire and chart review.

**Results**: At T0, 59.24% (n = 141) children reported experiencing chronic pain and 1.26% (n = 3) were using opioids. After surgery at 6 months, 34.5% (n = 78) developed CPSP and 3.7% (n = 8) were using opioids. Interestingly, 2 of the T0 patients using opioids discontinued their use post-surgically. The proportion of children with CPSP remained stable from 6-months to one year post-surgery 38.1% (n = 86), McNemar χ^2^ *p* = .609) as did the incidence of opioid use (3.98%, n = 9, McNemar, *p *= 1.00).

**Discussion/Conclusion**: Approximately one third of children undergoing major orthopedic surgery have developed CPSP by one year and 3% are using an opioid medication for the pain. Further research is required to examine the predictors of CPSP and opioid use.

#### Underlying risk factors for chronic post-traumatic headache: a preliminary study

Sabrina Bouferguene^a^, Bérengère Houzé^b^, Pierre Rainville^c^ and Caroline Arbour^d^

^a^Department of Biomedical Sciences, Faculty of Medicine, Université de Montréal, Montréal, Québec, Canada; ^b^Research Center and Trauma Division, Hôpital du Sacré-Coeur de Montréal, Montréal, Québec, Canada; ^c^Department of Stomatology, Faculty of Dental Medicine, Université de Montréal, Montréal, Québec, Canada; ^d^Faculty of Nursing, Université de Montréal, Montréal, Québec, Canada

**CONTACT** Sabrina Bouferguene sabrina.bouferguene@umontreal.ca Bsc of Biomedical Sciences, Faculty of Medicine, Université de Montréal, Montréal, Québec, Canada

© 2018 Sabrina Bouferguene, Bérengère Houzé, Pierre Rainville and Caroline Arbour. Published with license by Taylor & Francis Group, LLC.

This is an Open Access article distributed under the terms of the Creative Commons Attribution License (http://creativecommons.org/licenses/by/4.0/), which permits unrestricted use, distribution, and reproduction in any medium, provided the original work is properly cited.

**Introduction/Aim**: Chronic post-traumatic headache (CPTHA) is frequent after traumatic brain injury (TBI). Damage to neck structures, afferent nociceptive pathways, and pain catastrophizing could all contribute to the development of CPTHA. This ongoing study aims to identify risk factors of CPTHA after moderate-to-severe TBI.

**Methods**: TBI survivors were categorized in three groups: CPTHA, with other pain or without pain. All underwent thermal quantitative somatosensory testing. Thresholds for warmth and cold detection, as well as heat-pain were measured. Chronic pain was characterized with the Brief Pain Inventory. Pain Catastrophizing Scale was also used.

**Results**: So far, N = 16 adults (11 male; 41 ± 9 years) have been tested (n = 5 CPTHA, n = 6 other pain, n = 5 without pain) 8–24 months post-TBI. Chronic pain was mostly unilateral with an average intensity of 3 ± 2 at testing. No difference in cold and warmth detection thresholds was observed between participants with CPTHA and other pain. Surprisingly, no neck damage (as per imagery testing) was found in CPTHA participants, whereas it was found in 50% (n = 3) of those with other pain. Participants with CPTHA on the other hand showed a significant tendency to exhibit higher pain catastrophizing s (12 ± 6, *F *= 2.273, *p *= 0.149) compared to those with other pain (7 ± 6) or no pain (4 ± 5).

**Discussion/Conclusions**: Our preliminary findings suggest that the characteristics of CPTHA resembled those of other central pain syndrome post-TBI. The sensory profile indicated that damage to the temperature systems could be a necessary but not sufficient condition for the development of CPTHA. Pain catastrophizing however could contribute to CPTHA.

#### Parental chronic pain status related to higher posttraumatic stress symptoms and lower quality of life in youth with chronic pain

Jaimie Beveridge^a^, Amanda Stone^b^, Anna Wilson^b^ and Melanie Noel^a^

^a^Department of Psychology, University of Calgary, Calgary, OR, Canada; ^b^Department of Pediatrics, Oregon Health & Science University, Portland, AB, USA

**CONTACT** Jaimie Beveridge jaimie.beveridge@ucalgary.ca

© 2018 Jaimie Beveridge, Amanda Stone, Anna Wilson and Melanie Noel. Published with license by Taylor & Francis Group, LLC.

This is an Open Access article distributed under the terms of the Creative Commons Attribution License (http://creativecommons.org/licenses/by/4.0/), which permits unrestricted use, distribution, and reproduction in any medium, provided the original work is properly cited.

**Introduction/Aim**: Posttraumatic stress disorder symptoms (PTSS) are highly prevalent in children with chronic pain and their parents and are associated with worse pain outcomes and lower quality of life (QoL). Current models suggest that parental factors (e. g., parental physical and mental health) may further increase children’s risk of developing co-occurring chronic pain and PTSS. Indeed, children whose parents have chronic pain are significantly more likely to report pain and poor psychological outcomes than children of parents without chronic pain. Research has yet to examine how parental chronic pain relates to co-occurring PTSS in children with chronic pain. The aim of this study was to examine how parental chronic pain relates to parent and child PTSS and child QoL in a sample of youth with chronic pain.

**Methods**: To date, 213 youth (67% girls) aged 8–18 receiving tertiary-level treatment for chronic pain (headache, abdominal, or complex pain) and one of their parents (90% mothers) completed questionnaires that assessed parent and child pain and PTSS and child QoL.

**Results**: As expected, findings revealed that parental chronic pain was significantly related to parent and child PTSS and child QoL. Parents with chronic pain reported higher PTSS than parents without chronic pain. Children of parents with chronic pain reported higher PTSS and lower QoL than children of parents without chronic pain.

**Discussion/Conclusions**: Parental chronic pain may be a risk factor for the development of co-occurring PTSS in children with chronic pain. Future longitudinal research is needed to examine the directionality of this relationship.

#### “Achy Penguin”: usability testing of a smartphone-based tool to improve pain assessment and management in children aged 4–7 years

Jennifer Stinson^a^, Kathryn Birnie^a^, Chitra Lalloo^b^, Ted Gerstle^c^, Lesley Baker^d^, Carley Ouellette^e^, Tamara Do Amaral^b^, Cynthia Nguyen^b^ and Fiona Campbell^f^

^a^Lawrence S. Bloomberg Faculty of Nursing, University of Toronto, Child Health Evaluative Sciences, The Hospital for Sick Children, Toronto, Canada; ^b^Child Health Evaluative Sciences, Hospital for Sick Children, Toronto, Canada; ^c^Division of General and Thoracic Surgery, Hospital for Sick Children, Toronto, Canada; ^d^For Jack & Jill, Seattle, WA, USA; ^e^School of Nursing, McMaster University, Hamilton, Canada; ^f^Anesthesia, University of Toronto, Child Health Evaluative Sciences, The Hospital for Sick Children, Toronto, Canada

**CONTACT** Jennifer Stinson Jennifer.stinson@sickkids.ca

© 2018 Jennifer Stinson, Kathryn Birnie, Chitra Lalloo, Ted Gerstle, Lesley Baker, Carley Ouellette, Tamara Do Amaral, Cynthia Nguyen and Fiona Campbell. Published with license by Taylor & Francis Group, LLC.

This is an Open Access article distributed under the terms of the Creative Commons Attribution License (http://creativecommons.org/licenses/by/4.0/), which permits unrestricted use, distribution, and reproduction in any medium, provided the original work is properly cited.

**Introduction/Aim**: Achy Penguin is a smartphone-based tool designed to improve pain assessment and management among children. The objective is to evaluate and refine the usability of the Achy Penguin application in children with acute postoperative pain.

**Methods**: Qualitative usability testing were conducted in 3 iterative cycles, with 5–7 participants per cycle. A purposive sample of English-speaking children (4–7 years) who underwent daycase surgeries were recruited from a Canadian pediatric tertiary care centre. Consent was obtained, questionnaires were completed and participants proceeded through the application in a step-wise manner. A trained observer recorded difficulties and navigation errors. Participants provided feedback on the application and recommendations for improvement through an audio-recorded semi-structured interview. Data was analyzed using content analyses and used to refine the application for further testing cycles.

**Results**: Three iterative cycles of usability testing have been completed with 20 participants (n = 20, mean age = 5.80 years, SD = 0.95). All participants reported having a smartphone in the household and the majority of participants felt ‘comfortable’ or ‘very comfortable’ using a smartphone (n = 17). Usability issues were identified after each cycle of testing. Specifically, (1) aesthetics (i. e., larger font/icons, adding interactive visuals), (2) ease of use (i. e. appropriate reading level, adding audio instructions and navigation tools, clarifying game instructions), (3) pain reporting (i. e. defined body map), and (4) gameplay (i. e. customization).

**Discussion/Conclusions**: Participants found Achy Penguin enjoyable, easy to use, and quick to complete. Refinements to the app will be made prior to conducting a pilot randomized controlled trial.

#### Researching what matters to improve chronic pain care in Canada: a priority setting process to support patient-oriented research

Patricia A. Poulin^a^, Yaadwinder Shergill^b^, Heather Romanow^c^, Jason W. Busse^d^, Christine T. Chambers^e^, Lynn Cooper^f^, Paula Forgeron^g^, Anita Olsen Harper^h^, Maria Hudspith^i^, Alfonso Iorio^j^, Chitra Lalloo^k^, Carley Ouellette^l^, Rosalind Roberston^m^, Sandy Smeenk^n^, Bonnie Stevens^o^ and Jennifer Stinson^p^

^a^The Ottawa Hospital Pain Clinic, The Ottawa Hospital Research Institute, School of Psychology, Faculty of Social Sciences, Department of Anesthesiology and Pain Medicine, Faculty of Medicine, University of Ottawa, Ottawa, Ontario, Canada; ^b^The Ottawa Hospital Research Institute, Centre for Collaborative Health, Oakville, Ontario, Canada; ^c^The Ottawa Hospital Research Institute; ^d^Department of Anesthesia, Department of Health Research Methods, Evidence and Impact, The Michael G. DeGroote Institute for Pain Research and Care, McMaster University, Hamilton Health Sciences Centre, Hamilton, Ontario, Canada; ^e^Canada Research Chair in Children’s Pain, Pediatrics and Psychology & Neuroscience Dalhousie University and IWK Health Centre, Halifax, Nova Scotia, Canada; ^f^Canadian Pain Coalition, Oshawa, Ontario, Canada; ^g^School of Nursing, University of Ottawa; Faculty of Medicine, Dalhousie University; Adjunct Researcher, Children’s Hospital of Eastern Ontario Research Institute, Ottawa, Ontario, Canada; ^h^Independent Researcher, Ottawa, Ontario, Canada; ^i^Pain BC, Vancouver, British Columbia, Canada; ^j^Department of Health Research Methods, Evidence, and Impact, Department of Medicine, McMaster University, Hamilton, Ontario, Canada; ^k^Improving Outcomes in Child Health Through Technology (iOUCH) Lab, Child Health Evaluative Sciences, The Hospital for Sick Children, Peter Gilgan Centre for Research and Learning, Institute of Health Policy, Management and Evaluation, University of Toronto, Toronto, Ontario, Canada; ^l^School of Nursing, McMaster University, Hamilton, Ontario, Canada; ^m^Patient representative, Toronto, Ontario, Canada; ^n^The ILC Chronic Pain and Ehlers Danlos Syndromes Charitable Foundation, Oakville, Ontario, Canada; ^o^University of Toronto Centre for the Study of Pain, University of Toronto, Research Institute, The Hospital for Sick Children, Toronto, Ontario, Canada; ^p^Marie Jo Haddad Nursing Chair in Child Health, Child Health Evaluative Sciences, The Hospital for Sick Children Chronic Pain Program, Lawrence S. Bloomberg Faculty of Nursing, University of Toronto, Toronto, Ontario, Canada^e^

**CONTACT** Patricia Poulin ppoulin@toh.on.ca

© 2018 Patricia Poulin, Yaadwinder Shergill, Jason Busse, Christine Chambers, Lynn Cooper, Paula Forgeron, Olsen Harper Anita, Maria Hudspith, Alfonso Iorio, Chitra Lalloo, Carley Ouellette, Rosalind Roberston, Sandy Smeenk, Bonnie Stevens, Jennifer Stinson, and Heather Romanow. Published with license by Taylor & Francis Group, LLC.

This is an Open Access article distributed under the terms of the Creative Commons Attribution License (http://creativecommons.org/licenses/by/4.0/), which permits unrestricted use, distribution, and reproduction in any medium, provided the original work is properly cited.

**Introduction/Aim**: Chronic pain affects more than 6 million Canadians. To ensure that research focuses in areas important to those who will be most impacted by the results, patient and clinicians need to be involved in setting research priorities. The aim of this study is to leverage patient experiences to identify chronic pain research priorities in Canada

**Methods**: The process was informed by the James Lind Alliance. After gathering an exhaustive list of questions using surveys, town-hall meetings, interviews and social media consultations, we used a computerized Delphi with 4 successive iterations to select the final list of priorities. The final Delphi round was conducted by a panel of 10 patients living with chronic pain and 10 clinicians from different disciplines

**Results**: We received more than 5,000 suggestions from 1,500 people. The Delphi process led to the identification of 14 questions fitting under the following 4 themes: Improving knowledge and competencies in chronic pain; improving patient-centered chronic pain care; preventing chronic pain and reducing associated symptoms; as well as improving access to and coordination of patient centered chronic pain care. Challenges included the issue of chronic pain being ubiquitous to many diseases, leading to many initial suggestions focusing on these diseases. We also identified the need for further engagement efforts with marginalized groups in order to validate the priorities identified or identify different sets of priorities specific to these groups

**Discussion/Conclusions**: The priorities identified can guide patient-oriented chronic pain research to ultimately improve the care offered to people living with pain.

#### Characterization of the 24 hour sleep-awake cycle in neuropathic pain rats

Martha López-Canul^a^, Maria-Luisa Vigano^b^, Rafael Ochoa-Sanchez^c^, Shelly Yin^d^ and Gabriella Gobbi^e^

^a^Martha López-Canul, PhD, McGill University, Department of Psychiatry Montreal, QC, Canada, martha.lopezcanul@mcgill.ca; ^b^Maria-Luisa Vigano, Degrees McGill University, Department of Psychiatry Montreal, QC, Canada, mvigano@wearelcc.ca; ^c^Rafael Ochoa-Sanchez, PhD, University of Montreal, Centre de recherche du Centre hospitalier de l’Université de Montréal (CRCHUM), Montreal, QC, Canada, rochoa.sanchez@gmail.com; ^d^Department of Psychiatry Montreal, McGill University, QC, Canada; ^e^Gabriella Gobbi, MD PhD, McGill University, Department of Psychiatry Montreal, QC, Canada. gabriella.gobbi@mcgill.ca

**CONTACT** Martha López-Canul atselep@uoi.gr

© 2018 Martha López-Canul, Maria-Luisa Vigano, Rafael Ochoa-Sanchez and Gabriella Gobbi. Published with license by Taylor & Francis Group, LLC.

This is an Open Access article distributed under the terms of the Creative Commons Attribution License (http://creativecommons.org/licenses/by/4.0/), which permits unrestricted use, distribution, and reproduction in any medium, provided the original work is properly cited.

**Introduction/Aim**: Neuropathic pain (NP) is a chronic and disabling condition characterized by hyperalgesia and allodynia, caused by neuronal injury. Between 50% and 88% of patients with NP suffer from sleep disturbances (Finan et al., 2013), reducing productivity and increasing the health care costs. Little is known about the neuropathology underlying the link between NP and sleep disorders. The aim of this study is thus to determine whether and how the NP produced by the ligature of the nerves L5/L6 alters the sleep-awake cycle in rats.

**Methods**: NP was induced according to Kim and Chung (1992). On day 10 after NP induction, rats were implanted with six stainless-steel wire electrodes in the skull, for EEG/EMG monitoring. On day 14 after NP, tactile allodynia was measured with Von Frey filaments and the EEG/EMG recorded over a period of 24 h (from 6 PM to 6 PM).

**Results**: All animals with L5/L6 nerve ligature developed allodynia. They showed a 24h reduction of non- rapid eyes movement (NREM) sleep (-35%, p<0.05) compared to naive animals. During the light/inactive phase NP rats showed a decrease in NREM sleep (-35%, p<0.05) and increase wakefulness time (+39.6%, p<0.05), without alteration of REM (rapid eyes movement) sleep (p=0.66,NS). During the dark/active phase no differences were found in REM (p=0.33,NS), NREM (p=0.67,NS) and awake (p=0.71,NS) time.

**Discussion/Conclusions**: Our finding suggest that neuropathic pain induces an alteration of the physiological sleep-awake cycle by decreasing NREM sleep in the inactive phase, similar to the phenomenon reported in humans.

#### Patient values and preferences regarding opioids for chronic non-cancer pain: a systematic review

Anna Goshua^a^, Samantha Craigie^b^, Gordon H. Guyatt^c^, Arnav Agarwal^d^, Regina Li^e^, Justin S. Bhullar^f^, Naomi Scott^g^, Jasmine Chahal^a^, Sureka Pavalagantharajah^a^, Yaping Chang^c^, Rachel Couban^b^ and Jason W. Busse^h^

^a^Health Sciences, McMaster University, Hamilton, Canada; ^b^The Michael G. DeGroote National Pain Centre, McMaster University, Hamilton, Canada; ^c^Health Research Methods, Evidence, and Impact, McMaster University, Hamilton, Canada; ^d^Medicine, University of Toronto, Toronto, Canada; ^e^Medicine, McMaster University, Hamilton, Canada; ^f^Medicine, University of British Columbia, Vancouver, Canada; ^g^Midwifery, McMaster University, Hamilton, Canada; ^h^Anesthesia, McMaster University, Hamilton, Canada

**CONTACT** Anna Goshua goshuaam@mcmaster.ca

© 2018 Anna Goshua, Samantha Craigie, Gordon H. Guyatt, Arnav Agarwal, Regina Li, Justin S. Bhullar, Naomi Scott, Jasmine Chahal, Sureka Pavalagantharajah, Yaping Chang, Rachel Couban, and Jason W. Busse. Published with license by Taylor & Francis Group, LLC.

This is an Open Access article distributed under the terms of the Creative Commons Attribution License (http://creativecommons.org/licenses/by/4.0/), which permits unrestricted use, distribution, and reproduction in any medium, provided the original work is properly cited.

**Introduction/Aim**: Shared-care decision-making between patients and clinicians involves making trade-offs between desirable and undesirable consequences of management strategies. Although patient values and preferences should provide the basis for these trade-offs, few guidelines consider the relevant evidence when formulating recommendations. To inform a guideline for use of opioids in patients with chronic non-cancer pain, we conducted a systematic review of studies exploring values and preferences of affected patients towards opioid therapy.

**Methods**: We searched MEDLINE, CINAHL, EMBASE, and PsycINFO, from the inception of each database through October 2016. We included studies examining patient preferences for alternative approaches to managing chronic non-cancer pain, and studies that assessed how opioid-using chronic non-cancer pain patients value alternative health states and their experiences with treatment. We compiled structured summaries of the results.

**Results**: Pain relief and nausea and vomiting were ranked as highly significant outcomes across studies. When considered, the adverse effect of personality changes was rated as equally important. Constipation was assessed in most studies and was an important outcome, secondary to pain relief and nausea and vomiting. Of only two studies that evaluated addiction, both found it less important to patients than pain relief. No studies examined opioid overdose, death, or diversion.

**Discussion/Conclusions**: Our findings suggest that the adverse effects of opioids, especially nausea and vomiting, may reduce or eliminate any net benefit of opioid therapy unless pain relief is significant (>2-points on a 10-point scale). Further research should investigate patient values and preferences regarding opioid overdose, diversion, and death.

#### Clinical implications of opioid-induced hyperalgesia in chronic pain patients

Elena Kum^a^, Jason Busse^b,c^, Oscar Deleon-Casasola^d^, Mark Lema^d^ and Norm Buckley^b,c^

^a^Western University, London, ON, Canada; ^b^Department of Anesthesia, McMaster University, Hamilton, ON, Canada; ^c^Michael G. DeGroote Institute for Pain Research and Care, Hamilton, ON, Canada; ^d^Department of Anesthesiology, Roswell Park Cancer Institute, Buffalo, NY, US

**CONTACT** Norman Buckley buckleyn@mcmaster.ca

© 2018 Elena Kum, Jason Busse, Oscar Deleon-Casasola, Mark Lema and Norm Buckley. Published with license by Taylor & Francis Group, LLC.

This is an Open Access article distributed under the terms of the Creative Commons Attribution License (http://creativecommons.org/licenses/by/4.0/), which permits unrestricted use, distribution, and reproduction in any medium, provided the original work is properly cited.

**Introduction/Aim**: While opioids have been widely prescribed for their high analgesic potency, their clinical utility may be limited by an opposing effect that induces greater pain sensitivity and lower pain thresholds in patients. This counteractive phenomenon is known as opioid-induced hyperalgesia (OIH), a syndrome that increases a patient’s opioid requirements following exposure to opioids. Preclinical studies on the mechanisms of this phenomenon have supported the concept of OIH, but the clinical implications of these findings remain uncertain. Attempts to gather conclusive evidence for OIH in humans have been limited by the lack of consensus regarding the operational definition of OIH, the difficulty of applying this definition into clinical testing, and the difficulty of conducting high quality, randomized-controlled trials with this patient population. Understandably, this has led to a range of attitudes endorsed by pain practitioners about whether OIH merits significant attention in the clinical setting. A lack of standard guidelines for the diagnosis and treatment of OIH have also contributed to uncertainties surrounding the management of this condition. We wanted to understand whether chronic pain practitioners approach the OIH concept with uncertainty.

**Methods**: We conducted a cross-sectional, international survey of 850 chronic pain practitioners to examine their attitudes towards, and practices regarding OIH. Participants were recruited through membership in either the chronic pain section of the Canadian Anesthesiologists’ Society or the American Society of Regional Anesthesia and Pain Medicine, from which we received a 40% and 37% response rate, respectively. Responses to survey questions were analyzed for associations between background characteristics and responses, and written responses were summarized using a thematic analysis.

**Results**: 75.5% of respondents believed OIH to be a true clinical entity. However, the results revealed considerable practice variability in the diagnosis and treatment of OIH.

**Discussion/Conclusions**: This suggests that further research is warranted to develop evidence-based guidelines for managing OIH in the chronic pain patient population.

#### Level of catastrophizing and depression co-morbidity following whiplash injury

Catherine Paré^a^, Pascal Thibault^a^ and Michael J. L. Sullivan^a^

Psychology, McGill University, Montréal, Canada

**CONTACT** Catherine Paré catherine.pare2@mail.mcgill.ca

© 2018 Catherine Paré, Pascal Thibault and Michael J. L. Sullivan. Published with license by Taylor & Francis Group, LLC.

This is an Open Access article distributed under the terms of the Creative Commons Attribution License (http://creativecommons.org/licenses/by/4.0/), which permits unrestricted use, distribution, and reproduction in any medium, provided the original work is properly cited.

**Introduction/Aim**: Previous research has revealed significant relations between measures of catastrophizing and depression in individuals with whiplash injuries. Such findings raise the possibility that individuals with high scores on catastrophizing might have co-morbid clinically significant depression. The purpose of the present study was to examine the relation between level of catastrophizing and depression co-morbidity in individuals with whiplash injuries.

**Methods**: The study sample consisted to 310 (163 women) individuals aged 18 to 61 years (= 36.2 years) who had sustained whiplash injuries in rear collision motor vehicle accidents. The mean time since injury was 11.2 weeks, with a range of 4 to 48 weeks. All participants were enrolled in a multidisciplinary pain rehabilitation program and completed pre- and post-treatment measures of pain severity, depressive symptoms, and pain catastrophizing.

**Results**: Consistent with previous research, there was a significant correlation between pain catastrophizing and depressive symptoms (r = .51, p < .001). Frequency analyses revealed that the probability of scoring above clinical threshold on the BDI-II increased with level of catastrophizing. For individuals with PCS scores below 20, the prevalence of clinically significant symptoms of depression was 22%. For PCS scores between 20 and 25, the prevalence rose to 46%; for individuals with PCS scores above 25, the prevalence of clinically significant symptoms of depression was 62%.

**Discussion/Conclusions**: The results suggest that high levels of pain catastrophizing in individuals with whiplash injuries should alert clinicians to the possibility of depression co-morbidity, which would require intervention approaches different from those used to decrease catastrophic thinking.

#### Perioperative pregabalin and intraoperative lidocaine infusion to reduce persistent neuropathic pain after breast cancer surgery: a multicenter, factorial, randomized controlled pilot trial

James S. Khan^a^, Nicole Hodgson^b^, Stephen Choi^c^, Susan Reid^b^, James E. Paul^d^, Nicole J. Look Hong^e^, Claire Holloway^e^, Jason W. Busse^d,f^, Ian Gilron^g^, Norman Buckley^i^, Michael McGillion^i^, Hance Clarke^j^, Joel Katz^j^, Sean Mackey^a^, Ronen Avram^b^, Kayla Pohl^h^, Purnima Rao-Melacini^h^ and P. J. Devereaux^j^

^a^Division of Pain Medicine, Department of Anesthesiology, Perioperative and Pain Medicine, Stanford University, Palo Alto, CA, USA; ^b^Department of Surgery, McMaster University, Hamilton, Canada; ^c^Department of Anesthesia, Sunnybrook Health Sciences Center, Toronto, Canada; ^d^Department of Anesthesia, McMaster University, Hamilton, Canada; ^e^Department of Surgery, University of Toronto, Toronto, Canada; ^f^Michael G. DeGroote Institute for Pain Research and Care, Hamilton, Canada; ^g^Department of Anesthesiology & Perioperative Medicine, Queen’s University, Kingston, Canada; ^h^Population Health Research Institute, McMaster University, Hamilton, Canada; ^i^Department of Anesthesia, University of Toronto, Toronto, Canada; ^j^Department of Health Research Methods, Evidence and Impact, McMaster University, Hamilton, Canada

**CONTACT** James S. Khan james.khan@medportal.ca

© 2018 James S. Khan, Nicole Hodgson, Stephen Choi, Susan Reid, James E. Paul, Nicole J. Look Hong, Claire Holloway, Jason W. Busse, Ian Gilron, Norman Buckley, Michael McGillion, Hance Clarke, Joel Katz, Sean Mackey, Ronen Avram, Kayla Pohl, Purnima Rao-Melacini and P. J. Devereaux. Published with license by Taylor & Francis Group, LLC.

This is an Open Access article distributed under the terms of the Creative Commons Attribution License (http://creativecommons.org/licenses/by/4.0/), which permits unrestricted use, distribution, and reproduction in any medium, provided the original work is properly cited.

**Introduction/Aim**: Persistent neuropathic pain is a common complication after breast cancer surgery and there is insufficient evidence to support any preventative strategy. To establish the feasibility of a large trial of the effects of pregabalin and lidocaine in breast surgery, we conducted a pilot study.

**Methods**: We conducted a multicenter 2-by-2 factorial randomized placebo-controlled pilot trial of 100 female patients undergoing breast cancer surgery. Patients were randomized to receive an intraoperative lidocaine infusion (1.5 mg/kg bolus followed by 2 mg/kg/hr) or placebo and perioperative pregabalin (300 mg preoperatively, 75 mg twice daily for nine days) or placebo. Primary outcome was feasibility of conducting a larger definitive trial. Secondary outcomes included persistent neuropathic pain, acute postoperative pain, pain interference, quality of life, and adverse events.

**Results**: All our feasibility criteria were surpassed; recruitment of 100 patients within 42-weeks, follow-up rate of 100%, and study-drug compliance of ≥80%. At 3-months, 53% of patients reported persistent neuropathic pain; 77.4% had mild pain (numeric rating scale [NRS] 0–3), 15.1% had moderate pain (NRS 4–6), and 7.5% severe pain at rest (NRS 7–10). Lidocaine significantly reduced the development of persistent neuropathic pain at 3-months (43.1% vs 63.3%; RR 0.68, 95% CI 0.47–1.0; p = 0.049). Pregabalin did not affect persistent pain (60% vs 46%; RR 1.3, 95% CI 0.90 to 1.90; p = 0.166) and neither pregabalin nor lidocaine impacted acute postoperative pain, opioid consumption, pain interference, or quality of life.

**Discussion/Conclusions**: Our pilot trial demonstrated feasibility of conducting a larger definitive trial to establish the effects of perioperative pregabalin and intraoperative lidocaine infusion to prevent persistent neuropathic pain after breast cancer surgery.

#### Pain phenotypes in rheumatoid arthritis: differences in patient outcome and functioning

Abi Muere^a^, Nadil Zeiadin^b^, Matthew Woo^b^, Alison Crawford^a^, Phylicia Verreault^c^, Dean A. Tripp^d^ and Mala Joneja^b^

^a^Psychology, Queen’s University, Kingston, Canada; ^b^Medicine, Queen’s University, Kingston, Canada; ^c^Psychology, Université Laval, Quebec City, Canada; ^d^Psychology, Anaesthesiology, Urology, Queen’s University, Kingston, Canada

**CONTACT** Abi Muere abigail.muere@queensu.ca

© 2018 Abi Muere, Nadil Zeiadin, Matthew Woo, Alison Crawford, Phylicia Verreault, Dean A. Tripp and Mala Joneja. Published with license by Taylor & Francis Group, LLC.

This is an Open Access article distributed under the terms of the Creative Commons Attribution License (http://creativecommons.org/licenses/by/4.0/), which permits unrestricted use, distribution, and reproduction in any medium, provided the original work is properly cited.

**Introduction/Aim**: Rheumatoid arthritis (RA) is a chronic degenerative joint disease characterized by painful joint inflammation, often in the hands and feet. The current study investigated the relationship between pain phenotype, biological factors (disease activity, disability), psychological factors (catastrophizing, depressive symptoms, resilience), and social factors (social support) over a six-month period.

**Methods**: RA patients (N = 116; 89 women, 27 men) recruited from tertiary care completed questionnaires and a measure of objective disease activity after a rheumatology appointment (Time 1). Patients completed the same questionnaires six months post-appointment (Time 2). Patients were classified into pain phenotypes at Time 1 based on number of pain sites. “Typical” patients reported pain in the hands and/or feet, “Typical-Plus I” participants reported pain in one to three additional areas, and “Typical-Plus II” participants reported pain in four or more additional areas. ANOVA and RM ANOVA were conducted to examine differences in patient outcome and biopsychosocial factors among pain phenotypes.

**Results**: “Typical-Plus II” reported higher levels of disease activity, disability, and depressive symptoms than “Typical-Plus I” and “Typical” patients. “Typical-Plus I” patients reported higher levels of disease activity and disability than “Typical” patients. Catastrophizing and social support decreased linearly over six months, regardless of phenotype. No interaction effects between pain phenotype and time were significant.

**Discussion/Conclusions**: Pain phenotypes differ with respect to several indices of patient functioning. Given the adverse effect of depressive symptoms and catastrophizing on patient outcome, the present findings advocate the use of screening tests for catastrophizing and depression for specific pain phenotypes.

#### CRPS prognosis: a tertiary care pain clinic cohort 2 years after end of active treatment

Guillaume Martel^a^, Julie Steele^a^ and Anne-Marie Pinard^a,b^

^a^Département d’anesthésiologie et de soins intensifs, Université Laval, Québec, Québec, Canada; ^b^Anesthesiolgist, CHUL du Chu de Québec, Québec, Canada

**CONTACT** Guillaume Martel guillaume.martel.4@ulaval.ca

© 2018 Guillaume Martel, Julie Steele and Anne-Marie Pinard. Published with license by Taylor & Francis Group, LLC.

This is an Open Access article distributed under the terms of the Creative Commons Attribution License (http://creativecommons.org/licenses/by/4.0/), which permits unrestricted use, distribution, and reproduction in any medium, provided the original work is properly cited.

**Introduction/Aim**: Little data is available on long term prognosis of patients with Complex Regional Pain Syndrome (CRPS). Our previous study showed a strong correlation between opioid consumption or long reference delay and negative outcome at discharge from clinic. The aim of this study is to describe the evolution of a cohort CRPS patients two years after being treated in a tertiary pain clinic.

**Methods**: After approval of Research Ethics Board, 65 patients with clinical upper limb CRPS (from a previous retrospective study) were contacted. Demographic data, signs and symptoms of CRPS, psychometric scales, active range of motion (AROM) and grip strength were obtained and added to the database from the first study. Our main outcomes included pain reduction, return to work and self-reported recovery.

**Results**: Of the 65 contacted patients, 49 participated to the study, either by an interview (questionnaires only n = 7) or a visit at the clinic (questionnaire and AROM measurement n = 42). Eight patients (16%) were still invalid from CRPS (69% at the beginning of treatments). Since the end of treatments, the *Brief Pain Inventory* score dropped for a mean 20 points (p < 0,05) and 61% considered that their CRPS condition either healed or improved. Grip strength and the majority of AROM measured upon liberation from the clinic remained stable.

**Discussion/Conclusions**: This primary analysis tends to demonstrate that although disability and pain intensity continue to decrease, most patients remain with clinical sequelaes. AROM generally remains stable after the end of active treatments.. Further multivariate et bivariate analysis will come.

#### The chronic pain network and knowledge translation

Alfonso Iorio^a^, Mary Brachaniec^b^, Norm Buckley^c^, Stephanie Perreault^d^, Jennifer Stinson^e^, John Lavis^f^, Isabel Jordan^g^, Lesley Singer^h^ and Megan Groves^i^

^a^Department of Health Research Methods, Evidence, and Impact, McMaster University, Hamilton, Ontario, Canada; ^b^Patient Partner, Chronic Pain Network, Canada; ^c^, McMaster University, Hamilton, Ontario, Canada; ^d^Patient Partner, Chronic Pain Network, Canada; ^e^Department of Anesthesia and Pain Management, The Hospital for Sick Children, Toronto, Ontario, Canada; ^f^Health Research Methods, Evidence, and Impact, McMaster University, Hamilton, Ontario, Canada; ^g^Patient Partner, Chronic Pain Network, Canada; ^h^Patient Partner, Chronic Pain Network, Canada; ^i^Communications Coordinator, Chronic Pain Network, Canada

**CONTACT** Norm Buckley buckleyn@mcmaster.ca

© 2018 Alfonso Iorio, Mary Brachaniec, Norm Buckley, Stephanie Perreault, Jennifer Stinson, John Lavis, Isabel Jordan, Lesley Singer and Megan Groves. Published with license by Taylor & Francis Group, LLC.

This is an Open Access article distributed under the terms of the Creative Commons Attribution License (http://creativecommons.org/licenses/by/4.0/), which permits unrestricted use, distribution, and reproduction in any medium, provided the original work is properly cited.

**Introduction/Aim**: The overarching goal of the Chronic Pain Network’s Knowledge Translation committee is to create a robust and comprehensive knowledge translation infrastructure, building on existing expertise and leveraging available resources.

**Methods**: In collaboration with McMaster University’s Health Information Research Unit, the Chronic Pain Network saw the creation of Pain+CPN, a knowledge translation platform. The platform sees involvement from doctors, researchers, clinicians and patient partners in reviewing pain-related journal articles and assigning them a rating.

**Results**: To date, Pain+CPN pulls articles from more than 120 journals. Additional patients and professionals alike are being recruited to serve as raters, helping to identify relevant health goals and health research priorities for each group of stakeholders.

**Discussion/Conclusions**: Committee next steps will be determining best practices for the dissemination of information each stakeholder group has self-identified as relevant to them.

#### The patient oriented research of the chronic pain network

Cyril Schneider^a^, Billie Jo Bogden^b^, Jason Busse^c^, Louis Gendron^d^, Ian Gilron^e^, Joy MacDermid^f^, Tiffany Rice^g^, Barry Sessle^h^, Janice Sumpton^i^, Marc White^j^, Norm Buckley^k^, Kim Begley^l^ and Donna Marfisi^l^

^a^Department of Rehabilitation, Université Laval, Quebec, Quebec, Canada; ^f^Departments of Surgery and Epidemiology, University of Western Ontario, London, Ontario, Canada; ^b^Patient Partner, Chronic Pain Network, Ottawa, Ontario, Canada; ^c^Anesthesia, McMaster University, Hamilton, Ontario, Canada; ^d^Department of Pharmacology-physiology, Université Sherbrooke, Sherbrooke, Quebec, Canada; ^e^Biomedical and Molecular Science, Queen’s University, Kingston, Ontario, Canada; ^g^Alberta Children’s Health Research Institute, Calgary, Alberta, Canada; ^h^Department of Physiology, University of Toronto, Toronto, Ontario, Canada; ^i^Patient Partner, Chronic Pain Network, London, Ontario, Canada; ^j^Patient Partner, Chronic Pain Network, Vancouver, British Columbia, Canada; ^k^Anesthesiology, McMaster University, Hamilton, Ontario, Canada; ^l^Coordination Center, Chronic Pain Network, Hamilton, Ontario, Canada

**CONTACT** Cyril Schneider cyril.schneider@rea.ulaval.ca

© 2018 Cyril Schneider, Billie Jo Bogden, Jason Busse, Louis Gendron, Ian Gilron, Joy MacDermid, Tiffany Rice, Barry Sessle, Janice Sumpton, Marc White, Norm Buckley, Kim Begley and Donna Marfisi. Published with license by Taylor & Francis Group, LLC.

This is an Open Access article distributed under the terms of the Creative Commons Attribution License (http://creativecommons.org/licenses/by/4.0/), which permits unrestricted use, distribution, and reproduction in any medium, provided the original work is properly cited.

**Introduction/Aim**: The Patient Oriented Research (POR) committee of the Chronic Pain Network (CPN) seeks to ensure that research projects funded by the Chronic Pain Network align with the Network’s strategy.

**Methods**: The committee, made up of seven researchers, four patients, and three ex-officios, reviews already vetted research projects and examines their milestones and their commitment to or progress in areas such as Patient Engagement, Training & Mentoring initiatives, and Knowledge Translation activities.

**Results**: The POR committee collaborated with the other CPN committees to build a review template that is useful to assess each project’s progress and to make recommendations to the executive committee for pursuing project’s funding or not. The inner processing of the POR committee proves to be a successful partnership between patients and researchers. Challenges of POR committee included procedural delays and different funding timelines between projects, all research types to cover (basic vs. behavioral science vs. population surveys vs. clinical trials), number of reviews and core member expectations.

**Discussion/Conclusions**: The review template continues to evolve and improve with the feedback received from project leads and other committees. The committee continues to seek new and meaningful ways to create successful partnerships between patients and researchers and to identify appropriately the resources related to recommendations (e.g., provincial support units).

#### The chronic pain network and patient engagement

Maria Hudspith^a^, Mario Di Carlo^b^, Carolynn Bulmer^c^, Kathleen Eubanks^d^, Janet Gunderson^e^, Therese Lane^d^, Rebecca Lee^f^, Patricia Poulin^g^, Linda Wilhelm^h^, Cyndi Sand-Eveland^i^, Jennifer Stinson^j^, Dawn Richards^k^, Kim Begley^l^ and Norm Buckley^m^

^a^Vancouver, British Columbia, Canada; ^b^Patient Partner, Chronic Pain Network, Canada; ^c^Patient Partner, Chronic Pain Network, Canada; ^d^Patient Partner, Chronic Pain Network, Canada; ^e^Patient Partner, Chronic Pain Network, Canada; ^f^Patient Partner, Chronic Pain Network, Canada; ^g^Department of Anesthesiology and Pain Medicine, University of Ottawa, Ottawa, Ontario, Canada; ^h^Patient Partner, Chronic Pain Network, Canada; ^i^Patient Partner, Cyndi Sand-Eveland, Chronic Pain Network, Canada; ^j^Department of Anesthesia and Pain Management, The Hospital for Sick Children, Toronto, Ontario, Canada; ^k^Patient Engagement Coordinator, Chronic Pain Network, Canada; ^l^Managing Director, Chronic Pain Network, Canada; ^m^McMaster University, Hamilton, Ontario, Canada

**CONTACT** Maria Hudspith maria@painbc.ca

© 2018 Maria Hudspith, Mario Di Carlo, Carolynn Bulmer, Kathleen Eubanks, Janet Gunderson, Therese Lane, Rebecca Lee, Patricia Poulin, Linda Wilhelm, Cyndi Sand-Eveland, Jennifer Stinson, Dawn P. Richards, Kim Begley and Norm Buckley. Published with license by Taylor & Francis Group, LLC.

This is an Open Access article distributed under the terms of the Creative Commons Attribution License (http://creativecommons.org/licenses/by/4.0/), which permits unrestricted use, distribution, and reproduction in any medium, provided the original work is properly cited.

**Introduction/Aim**: The Chronic Pain Network’s Patient Engagement committee seeks to increase the capacity of: (a) network members to meaningfully engage patients in their research projects, and (b) patients to be engaged in the Network, as well as to evaluate and report on the outcomes of patient engagement in Network activities.

**Methods**: Each committee within the Chronic Pain Network’s governance structure includes a patient co-chair and additional patient partners. The Patient Engagement committee helps set Network priorities, guide Knowledge Translation initiatives, and create meaningful opportunities for patients and researchers to collaborate throughout the research process utilizing unique lived experience.

**Results**: Working in collaboration with the Patient Oriented Research committee, the Patient Engagement committee helped finalize the template used during each Network-funded project review. Increased opportunities for engagement were also created through matching patient partners with Network-funded research projects to create dialogue and an exchange of ideas between patients and researchers. The Patient Engagement committee also began hosting monthly Patient Engagement in Research Rounds webinars to provide examples of meaningful patient engagement in research.

**Discussion/Conclusions**: There is a continuing need for resources to increase commitment to, and skills in, patient engagement in pain research. The Network aims to foster this, building the capacity of both patient partners and pain researchers in Canada.

#### Rivermead post–concussion symptom questionnaire (RPQ) scores, concussion diagnosis, and psychological symptoms in a chronic pain population

Sean Sutton^a^, Alex Christopher Mailis^a^, Shah Fatima Lakha^a^ and Angela Mailis^b,a,c^

^a^Pain and Wellness Centre, Vaughan, Canada; ^b^Dept. of Medicine, Division of Physical Medicine, UofToronto; ^c^Toronto Rehab Institute/University Health Network, Toronto, Canada

**CONTACT** Sean Sutton ssutton@thepwc.ca

© 2018 Sean Sutton, Alex Christopher Mailis, Shah Fatima Lakha and Angela Mailis. Published with license by Taylor & Francis Group, LLC.

This is an Open Access article distributed under the terms of the Creative Commons Attribution License (http://creativecommons.org/licenses/by/4.0/), which permits unrestricted use, distribution, and reproduction in any medium, provided the original work is properly cited.

**Aim:** To explore the Rivermead Post­Concussion Symptom Questionnaire (RPQ) applicability in a population of chronic pain patients involved in a car accident.

**Methods:** We compared pain patients referred to an interdisciplinary pain program with concussion/post-concussion diagnosis (CD) by specialists or family physicians to patients without (NCD). Data was collected on demographics, pain diagnosis, psychopathology, and Rivermead scores.

**Results:** The study group consisted of 75 patients (females/males 1.78/1); median age 41 yrs; 61.3% Canadian born; 18.5 ± 15 months post MVA (median 20 mos); 75.9% drivers, 17.2% passengers, 8% pedestrians. The CD and NCD groups included 13 and 62 patients respectively. Loss of consciousness had been sustained in 1/13 CD and 3/62 NCD patients.

RPQ-3 (for acute post-concussion symptoms) was 9.46 (range 5–12) in the CD and 4.3 (range 2–12) in the NCD group. RPQ-13 (for late post-concussion symptoms) was 40 (range 26–50) in the CD and 30.7 (range 0–48) in the NCD group.

Of the CD group, 12/13 (92.3%) had symptoms consistent with 1–3 DSM 5 diagnoses (Trauma, Anxiety and/or Depressive Disorders) as compared to 47/62 (75.8%) NCD patients. The commonest diagnoses for both the CD and NCD group included myofascial pain and mechanical neck or back pain.

**Conclusion:** The higher RPQ-3 and RPQ-13 scores in the CD group cannot be attributed to concussion/post-concussion syndrome given the prevalence of Trauma, Anxiety and/or Depressive symptoms in the overwhelming majority of CD patients. The similarity of symptoms between the two subgroups and their relevance to concussion diagnosis will be discussed.

#### Components of emotion regulation related to pain, depression, and disability in women with interstitial cystitis/bladder pain syndrome

Alison Crawford^a^, Dean A. Tripp^b^, J. Curtis Nickel^c^, Lesley Carr^d^, Robert Moldwin^e^, Robert Mayer^f^, Laura Katz^g^ and Abi Muere^a^

^a^Department of Psychology, Queen’s University, Kingston, ON, Canada; ^b^Departments of Psychology, Urology, & Anesthesiology, Queen’s University, Kingston, ON, Canada; ^c^Department of Urology, Queen’s University, Kingston, ON, Canada; ^d^Department of Surgery, University of Toronto, Toronto, ON, Canada; ^e^Department of Urology, Hosftra University School of Medicine, New Hyde Park, NY, USA; ^f^Asante Physician Partners, Grants Pass, OR, USA; ^g^Michael G DeGroote Pain Clinic, McMaster University Hospital, Hamilton, ON, Canada

**CONTACT** Alison Crawford alison.crawford@queensu.ca

© 2018 Alison Crawford, Dean A. Tripp, J. Curtis Nickel, Lesley Carr, Robert Moldwin, Robert Mayer, Laura Katz and Abi Muere. Published with license by Taylor & Francis Group, LLC.

This is an Open Access article distributed under the terms of the Creative Commons Attribution License (http://creativecommons.org/licenses/by/4.0/), which permits unrestricted use, distribution, and reproduction in any medium, provided the original work is properly cited.

**Introduction/Aim**: Interstitial cystitis/bladder pain syndrome (IC/BPS) is a chronic pelvic pain condition with an unknown etiology. Treatment of this condition revolves around pain/symptom management. It has been found that catastrophizing and difficulties with emotion regulation play a role in the relationship between pain, depression, and disability. The aim of this study was to investigate the particular components of catastrophizing and emotion regulation at play in the maintenance of this relationship.

**Methods**: 225 women diagnosed with IC/BPS recruited from tertiary care clinics completed questionnaires regarding demographics, pain, catastrophizing, emotion regulation, depression, and pain-related disability. 135 of these women completed the same questionnaire six months and one year later. Regressions were run predicting depression and disability from subscales of catastrophizing and emotion regulation at baseline. Another regression was run predicting pain at one year from the subscales of catastrophizing at six months, while controlling for baseline depression and catastrophizing.

**Results**: Baseline pain, helplessness, nonacceptance, and rumination of negative emotions predicted baseline depression (*F*(8, 216) = 37.73, *p* < .001). Baseline pain, helplessness, magnification, and self-efficacy predicted baseline disability (*F*(8, 216) = 15.72, *p* < .001). Helplessness at six months predicted pain at one year, while controlling for baseline pain, depression, and catastrophizing (*β* = .42, *p* = .006).

**Discussion/Conclusions**: These findings highlight the role of helplessness in the maintenance of the relationship between pain, depression, and disability. Helplessness also plays a role in this relationship over time. Targeting helplessness using cognitive behavioural techniques could help women with IC/BPS manage their pain.

#### Parent-Child Reminiscing about Painful and Sad Events: A Comparative Analysis

Maria Pavlova^a^, Jillian Vinall^b^, Susan A. Graham^a^, Abbie Jordan^c^, Jill Chorney^d^ and Melanie Noel^a^

^a^Department of Psychology, University of Calgary, Calgary, Alberta, Canada; ^b^Department of Anesthesia, University of Calgary, Calgary, Alberta, Canada; ^c^Department of Psychology, University of Bath, Bath, UK; ^d^Department of Anesthesia, Pain Management and Perioperative Medicine, Dalhousie University, Halifax, Nova Scotia, Canada

**CONTACT** Maria Pavlova mpavlova@ucalgary.ca

© 2018 Maria Pavlova, Jillian Vinall, Susan A. Graham, Abbie Jordan, Jill Chorney and Melanie Noel. Published with license by Taylor & Francis Group, LLC.

This is an Open Access article distributed under the terms of the Creative Commons Attribution License (http://creativecommons.org/licenses/by/4.0/), which permits unrestricted use, distribution, and reproduction in any medium, provided the original work is properly cited.

**Introduction/Aim**: Children’s memories for pain are a powerful predictor of future pain experiences. Parent-child reminiscing about past events plays a key role in shaping young children’s memories. Parents differ dramatically in their reminiscing styles, whereas some parents are highly elaborative (i.e., they use open-ended detailed questions) and supportive, other parents are repetitive. Elaborative reminiscing has been linked with more accurate memory in children. No studies have examined how parents and children reminisce about past painful experiences and how this might differ from other emotional events. This study was the first to investigate differences in reminiscing style and content in parent-child narratives about painful versus sad events.

**Methods**: Two weeks following a tonsillectomy surgery, 78 4- to 7-year old children (42% girls, *M_age_ *= 5.21) and their parents completed a structured narrative elicitation task wherein they reminisced about two painful (surgery and non-surgery) and one sad event. Narratives were coded using an established coding scheme based on the developmental psychology literature.

**Results**: Parents reminisced with children in more adaptive ways about past events involving sadness versus pain. Specifically, parents were more elaborative, supportive, and used more negative emotion-laden words and explanations when talking about sad events as compared to painful events, *ps* < .05.

**Discussion/Conclusions**: Parents reminisce with children about past events involving sadness in a more adaptive way than they do about painful events. This may lead to less accurate recall of painful experiences and differential socialization of future pain behaviors. Parent-child reminiscing is amenable to intervention and offers a promising avenue for pediatric pain management interventions.

#### Associations between self-reported chronic pain, substance use, and workplace safety among Canadian oil and gas employees

Sabine Soltani^a^, Yannick Griep^a^, Daniel McGrath^a^ and Melanie Noel^a^

Department of Psychology, University of Calgary, Calgary, Alberta, Canada

**CONTACT** Sabine Soltani ssoltani@ucalgary.ca

© 2018 Sabine Soltani, Yannick Griep, Daniel McGrath and Melanie Noel. Published with license by Taylor & Francis Group, LLC.

This is an Open Access article distributed under the terms of the Creative Commons Attribution License (http://creativecommons.org/licenses/by/4.0/), which permits unrestricted use, distribution, and reproduction in any medium, provided the original work is properly cited.

**Introduction/Aim**: Pain-related concerns are the primary cause of healthcare utilization and disability among working-age adults in Canada.^1^ Less understood is how chronic pain may influence the wellbeing of those who continue to attend work despite their pain. There is an additional paucity of research examining chronic pain in men, who are disproportionately less likely to seek treatment for pain as compared to women.^2^,^3^ The oil and gas industry is a traditionally young, male-dominated economic sector, characterized by remote and challenging work environments, which enhance the potential for injury, impairment, and persistent pain.^4^,^5^ This study is the first to examine links between mental health, chronic pain, and maladaptive coping and their potential implications for workplace safety among a male sample of oil workers.

**Methods**: Men (*n *= 301, *M*_age_ = 41.80) employed in the Canadian oil industry completed psychometrically sound measures assessing pain characteristics, depressive symptoms, suicidality, substance use, and workplace burnout and safety as part of a comprehensive online questionnaire.

**Results**: Hierarchical regression analyses revealed that in men with chronic pain (*n *= 71), higher pain intensity predicted higher suicidality (*β *= .31,*R^2^ *= .10,*p *< .05), depressive symptoms (*β *= .95, *R^2^ *= .75,*p *< .001), alcohol use (*β *= .48,*R^2^ *= .49, *p *< .001), substance use (*β *= .57, *R^2^ *= .61, *p *< .001) and burnout (*β *= .53, *R^2^ *= .75, *p *< .001), and lower safety behaviours (*β *= -.66, *R^2^ *= .72, *p *< .001). In men without chronic pain (*n *= 284), higher pain intensity predicted higher suicidal ideation (*β *= .35, *R^2^ *= .11, *p *< .001) and safety behaviours (*β *= .54, *R^2^ *= .33,*p *< .001).

**Discussion/Conclusions**: The results suggest that, among men with chronic pain, pain intensity is strongly associated with mental health symptoms, substance use, and adverse work-related outcomes. Pain may also confer risk for suicidality before it becomes chronic.

References1.Arim R. A profile of persons with disabilities among Canadians aged 15 years or older, 2012. *Stat. Can.* 2015;89-654-X2015001.2.Kaur S, Stechuchak KM, Coffman CJ, Allen KD, Bastian LA. Gender differences in health care utilization among veterans with chronic pain. *J. Gen. Internal Med.* 2007;22:228–233.10.1007/s11606-006-0048-5PMC1824743173569913.Weir R, Browne G, Tunks E, Gafni A, Roberts J. Gender differences in psychosocial adjustment to chronic pain and expenditures for health care services used. *Clin. J. Pain.* 1996;12:277–290.10.1097/00002508-199612000-0000789698734.Guo HR, Tanaka S, Halperin WE, Cameron LL. Back pain prevalence in US industry and estimates of lost workdays. *Am. J. Public Health.* 1999;89:1029–1035.10.2105/ajph.89.7.1029PMC1508850103943115.Southwick R. Oil and gas industry presses provinces, Ottawa on pot ban in dangerous jobsites. *Calgary Herald.* 2017.

#### Patient-controlled analgesia versus continuous opioid infusion for the treatment of mucositis pain following hematopoietic stem cell transplantation: a retrospective review

Matthew Foss^a^ and Mary Lynch^a^

Department of Anesthesia, Pain Management & Perioperative Medicine, Dalhousie University, Halifax, Nova Scotia, Canada

**CONTACT** Matthew Foss matthew.foss@dal.ca

© 2018 Matthew Foss and Mary Lynch. Published with license by Taylor & Francis Group, LLC.

This is an Open Access article distributed under the terms of the Creative Commons Attribution License (http://creativecommons.org/licenses/by/4.0/), which permits unrestricted use, distribution, and reproduction in any medium, provided the original work is properly cited.

**Introduction/Aim**: Myeloablative chemotherapy prior to hematopoietic stem cell transplantation (HSCT) often results in the development of painful oral mucositis. Treatment involves high levels of opioid consumption resulting in significant side effects. Prior studies have shown that, compared to continuous opioid infusion (COI), patient-controlled analgesia (PCA) results in less opioid consumption and shorter duration of therapy but no absolute reductions in pain scores. The goal of this study was to determine the success of PCA implementation in this patient population along these same measures.

**Methods**: This was a retrospective chart review of HSCT patients one year before and after the transition from COI to PCA at the QEII Health Sciences Centre in Halifax, Nova Scotia. Opioid doses were standardized between patients by converting to oral morphine equivalents (OME). Pain scores were calculated using a weighted average over therapy duration. Groups were compared using independent-samples t-tests.

**Results**: Twenty-three patients received COI with three requiring naloxone. Twenty-two patients received PCA. The PCA group had significantly less opioid use (M = 506.0, SD = 239.9) than the COI group (M = 1181.5, SD = 659.9), t(36) = 3.64, p < 0.001, d = 1.13. Additionally, the PCA group required fewer days of intravenous opioid therapy (M = 5.28, SD = 3.29) than the COI group (M = 7.67, SD = 2.97), t(40), p = 0.018, d = 0.76. There was no difference in average pain scores between groups.

**Discussion/Conclusions**: This study demonstrates that patients using PCA versus COI experienced reduced opioid consumption, decreased therapy duration, and fewer cases requiring naloxone. Consistent with the literature, there was no reduction seen in pain scores. Further work is needed to effectively reduce the pain experienced by patients undergoing HSCT.

#### A systematic survey of the reporting quality of pilot studies in high impact anesthesia journals

Harsha Shanthanna^a^, Alka Kaushal^a^, Lawrence Mbuagbaw^b^, Rachel Couban^a^, Jason Busse^c^ and Lehana Thabane^b^

^a^Anesthesia, McMaster University, Hamilton, Canada; ^b^Department of Health Research Methods, Evidence, and Impact, McMaster University, Hamilton, Canada; ^c^Department of Health Research Methods, Evidence, and Impact, Department of Anesthesia, Michael G. DeGroote Institute for Pain Research and Care, McMaster University, Hamilton, Canada

**CONTACT** Harsha Shanthanna harshamd@gmail.com

© 2018 Harsha Shanthanna, Alka Kaushal, Lawrence Mbuagbaw, Rachel Couban, Jason Busse and Lehana Thabane. Published with license by Taylor & Francis Group, LLC.

This is an Open Access article distributed under the terms of the Creative Commons Attribution License (http://creativecommons.org/licenses/by/4.0/), which permits unrestricted use, distribution, and reproduction in any medium, provided the original work is properly cited.

**Introduction/Aim**: Pilot trials inform the feasibility of a definitive trial. Using the CONSORT pilot extension guidelines, we assessed their reporting quality in five high-impact anesthesia journals and explored factors associated with reporting quality.

**Methods**: Five highest-impact anesthesia journals were screened for randomized controlled trials published as pilot or feasibility trials between 2006 and 2016. A pair of reviewers independently screened citations, extracted data, and assessed reporting quality using the CONSORT checklist for both abstracts and full texts. We reported the percentage adherence for each item, along with the median and percentiles (Q1:Q3) or mean and standard deviation (SD) for all items. The factors considered to influence reporting were; 1) trial registration, 2) industry funding, 3) trial identification as a pilot or feasibility in title or abstract, 4) primary objective as ‘feasibility’, and 5) the specific journal. The association was estimated using generalized estimating equations and reported as incidence rate ratios (IRR) with 95% confidence intervals (CI).

**Results**: Out of 364 citations, 58 articles were eligible. The median (Q1:Q3) CONSORT abstract items reported was 5 (4:7), and the mean (SD) full text items reported was 13 (5). Significantly poor reporting was associated with ‘not registering the trial as a pilot’ for both abstracts and full texts; ‘industry funding’ for abstracts; and ‘hypothesis testing as the primary objective’ for full texts.

**Discussion/Conclusions**: The reporting quality of pilot trials published in leading anesthesia journals is poor. Journal editorial boards can encourage improved reporting by supporting adherence to the CONSORT extension for pilot trials.

#### Parental pain and children’s health and functional problems: The role of difficulty in performing parenting activities and partner parental support

Somayyeh Mohammadi^a,b^, Christine T. Chambers^a,b,c^, Natalie O. Rosen^d^, Annmarie Cano^e^, Perri R. Tutelman^f,g^ and Kristen S. Higgins^f,g^

^a^Centre for Pediatric Pain Research, IWK Health Centre, Halifax, Canada; ^b^Department of Pediatrics, Dalhousie University, Halifax, Canada; ^c^Department of Psychology and Neuroscience, Dalhousie University, Halifax, Canada; ^d^Department of Obstetrics and Gynecology, Dalhousie University, Halifax, Canada; ^e^Department of Psychology, Wayne State University, Detroit, Michigan, USA; ^f^Department of Psychology & Neuroscience, Dalhousie University, Halifax, Nova Scotia, Canada; ^g^Centre for Pediatric Pain Research, IWK Health Centre, Halifax, Nova Scotia, Canada

**CONTACT** Somayyeh Mohammadi somayyeh.mohammadi@iwk.nshealth.ca

© 2018 Somayyeh Mohammadi, Christine T. Chambers, Natalie O. Rosen, Annmarie Cano, Perri R. Tutelman and Kristen S. Higgins. Published with license by Taylor & Francis Group, LLC.

This is an Open Access article distributed under the terms of the Creative Commons Attribution License (http://creativecommons.org/licenses/by/4.0/), which permits unrestricted use, distribution, and reproduction in any medium, provided the original work is properly cited.

**Introduction/Aim**: Children of parents with chronic pain often report more health and functional problems compared to other children. This study investigated the mediating role of parents’ difficulty in performing parenting activities (e.g., helping children with their homework) and the moderating role of partner support in parenting (e.g., sharing responsibility for raising the child) on the relationships between parents’ pain intensity and their children’s health (i.e., pain intensity and general health) and functional problems at home, school, and in socializing.

**Methods**: Participants were 174 parents with chronic pain who had a child between the ages of 8–17 years old. They completed measures of their pain intensity, difficulty in performing parenting activities, partner support in parenting, and their child’s pain intensity, general health, and functional problems at home, school, and in socializing.

**Results**: Difficulty in performing parenting activities did not mediate the relationship between parent pain intensity and children’s pain intensity or general health. It did, however, mediate the relationship between parent pain intensity and children’s functional problems; that is more pain intensity in parents was related to having more difficulty in performing parenting activities, which in turn was related to more functional problems in children. Partner support in parenting moderated the association between performing parenting activities and children’s functioning.

**Discussion/Conclusions**: these findings suggest that parental pain and difficulty in performing parenting activities are negatively related to children’s functioning; however, receiving support from partners in parenting can mitigate some of these negative effects.

#### MyHealthMyRecord: a case study in registering the private perspective of learning to live with chronic pain of undefined origins

S. Fatima Lakha^a^, Carly B. Holtzman^b^, Z. Abidin Akkok^c^, Margot Whitfield^a^, West Suhanic^d^, Peter Pennefather^e^ and Deborah I. Fels^a^

^a^Inclusive Media and Design Centre, School of Information Technology Management, Ryerson University, Toronto, Canada; ^b^Department of Communication and culture, York University, Toronto, Canada; ^c^Department of Mechanical and Industrial Engineering, Ryerson University, Toronto, Canada; ^d^gDial Inc Toronto, Canada; ^e^Leslie Dan Department of Pharmacy, University of Toronto, Toronto, Canada

**CONTACT** S. Fatima Lakha slakha@ryerson.ca

© 2018 S. Fatima Lakha, Carly B. Holtzman, Z. Abidin Akkok, Margot Whitfield, West Suhanic, Peter Pennefather and Deborah I. Fels. Published with license by Taylor & Francis Group, LLC.

This is an Open Access article distributed under the terms of the Creative Commons Attribution License (http://creativecommons.org/licenses/by/4.0/), which permits unrestricted use, distribution, and reproduction in any medium, provided the original work is properly cited.

**Introduction/Aim**: We provide a case study report on a digital health application designed to support people adjusting to living with sudden onset pain without a clinical course for addressing its biomedical causes. Patients living with such conditions need non-medical accommodations to cope with their new reality. The case study describes a mobile app, MyHealthMyRecord, that allows patients to record 30–60 sec of personal and private health audio-video commentary. These self-authenticating data can then be curated and shared with others, and/or linked to and indexed with formal medical records.

**Methods**: One patient with serious biomedical painful chronic condition of sudden onset in Toronto, Ontario recorded commentaries over a 3-month period. Thematic analysis was used to identify the major themes in the data to address the research question: How can the individual’s perspective be represented through patent-generated narrative?

**Results**: The technology showed promise as a self-reflection/coping system, and media for including the person’s voice and experiences in official records of accommodations made to facilitate their return to their life before the onset of the pain condition. We will discuss an in-depth case study where Participant X recorded 64 short duration video clips over the 3-month period. We found that, despite her/his condition, s/he successfully made a variety of videos using different communication styles that documented experiences, concerns, issues, positive interactions and pain episodes.

**Discussion/Conclusions**: The insights from the recordings offered an in-the-moment perspective of the individual’s longitudinal journey of coping with the onset of chronic pain and resulting new life circumstances.

#### Predictors of completion of an online chronic pain management program for military and police

Pamela L. Holens^a^, Kristen Klassen^b^, Jeremiah Buhler^c^ and Michelle Paluzek^c^

^a^Clinical Health Psychology, University of Manitoba, Winnipeg, Manitoba, Canada; ^b^Disability Studies, University of Manitoba, Winnipeg, Manitoba, Canada; ^c^Psychology, University of Manitoba, Winnipeg, Manitoba, Canada

**CONTACT** Pamela L. Holens pholens@deerlodge.mb.ca

© 2018 Pamela L. Holens, Kristen Klassen, Jeremiah Buhler and Michelle Paluzek. Published with license by Taylor & Francis Group, LLC.

This is an Open Access article distributed under the terms of the Creative Commons Attribution License (http://creativecommons.org/licenses/by/4.0/), which permits unrestricted use, distribution, and reproduction in any medium, provided the original work is properly cited.

**Introduction/Aim**: Chronic pain is a serious health issue in Canada, with a greater prevalence in military and police personnel. Online psychological treatments for disorders such as chronic pain have recently been growing in popularity. Those with chronic pain may be more inclined to use online rather than in-person treatments due to the difficulty of attending in-person sessions when experiencing pain flare-ups, general discomfort when travelling, and pain-related avoidance behaviours. The purpose of this study was to determine characteristics of those more likely to complete an online chronic pain treatment designed for military and police personnel.

**Methods**: Participants were 57 individuals with chronic pain and a background in the military or RCMP who agreed to participate in an 8-week online treatment for chronic pain. Participants were also offered an optional biweekly group “support” session. Prior to treatment, participants provided demographic information and completed a number of pain-related measures and measures of depression, anxiety, and PTSD. Logistic regressions were performed to evaluate factors predictive of program completion.

**Results**: Just over 60% of participants completed the online program. Consistent with our expectations, completion of the online program was more likely among individuals who chose to attend the optional group-based support sessions. Contrary to our expectations, having a diagnosis of PTSD was also a predictor of completion of the program.

**Discussion/Conclusions**: Predictors of completion of an online chronic pain program for military and police included participation in optional group-based support sessions and having a diagnosis of PTSD. Reasons for these outcomes are discussed.

#### Mannitol (30%) Cream in the Treatment of Post-Herpetic Neuralgia

Tess Elaine Helen Debelle^a^, Hélène Bertrand^b^, Marylene Kyriazis^c^ and K. Dean Reeves^d^

^a^Department of Medicine, University of Groningen, Groningen, Netherlands; ^b^Department of Family Practice, Vancouver, BC, Canada; ^c^Faculty of Pharmaceutical Sciences, University of British Columbia, Vancouver, Canada; ^d^Department of Physical Medicine and Rehabilitation, University of Kansas, Roeland Park, Kansas, USA

**CONTACT** Tess Elaine Helen Debelle tessdeb94@gmail.com

© 2018 Tess Elaine Helen Debelle, Hélène Bertrand, Marylene Kyriazis and K. Dean Reeves. Published with license by Taylor & Francis Group, LLC.

This is an Open Access article distributed under the terms of the Creative Commons Attribution License (http://creativecommons.org/licenses/by/4.0/), which permits unrestricted use, distribution, and reproduction in any medium, provided the original work is properly cited.

**Introduction/Aim**: Post-herpetic neuralgia is painful, debilitating, and significantly affects quality of life. Treatments are often unsatisfactory with multiple side effects.

To determine if:

(1) mannitol cream is effective in treating nociceptive pain in post-herpetic neuralgia.
(2) adding menthol to the Mannitol cream results in further pain relief.

**Methods**: Twenty participants suffering with post-herpetic neuralgia were included. The first 24-day trial was a randomized double-blind, placebo controlled crossover study, comparing pain relief between the two creams with a baseline week. Participants were subsequently given a three-month supply of Mannitol cream, followed by a one-month supply of Mannitol and menthol cream.

**Results**: Paired samples t-tests were used to compare data. Mannitol cream generated slightly lower average pain levels during the 24-day trial, however neither the Mannitol (*p *= 0.506) nor placebo cream (*p *= 0.522) proved significant. Continued use of Mannitol cream resulted in significant improvements in average (*p *= 0.006) and maximum (*p *= 0.0058) pain levels. Brief Pain Inventory (BPI) (*p *= 0.0679 using intention to treat, *p *= 0.001 without) and Patient Health Questionnaire-9 (PHQ-9) (*p = 0.0421* for both) scores were also significant. After Mannitol and menthol cream use, maximum pain levels remained relatively unchanged (*p *= 0.0077), whereas PHQ-9 (*p *= 0.04) and BPI (*p *= 0.0114) scores decreased. The only side effect was dermatitis in three participants.

**Discussion/Conclusions**: Mannitol cream presumably works cumulatively, causing a greater therapeutic effect with continued use. Patient quality of life improves as Mannitol takes effect. It can therefore be considered a clinically effective treatment against post-herpetic neuralgia. Menthol appears to improve the effect of Mannitol on sufferers’ ability to function.

##### Sparing of the intercostobrachial nerve during breast cancer surgery involving axillary lymph node dissection: a systematic review

Lucas Gallo^a^, Annie Lok^b^, Andrei Smarandache^a^, Li Wang^c,d^, Long Ge^e^ and Jason W. Busse^c,e,f^

^a^Michael G. DeGroote School of Medicine, McMaster University, Hamilton, Canada; ^b^McMaster University; ^c^Department of Anesthesia, McMaster University, Hamilton, Canada; ^d^Michael G. DeGroote Institute for Pain Research and Care, Department of Anesthesia, McMaster University, Hamilton, Canada; ^e^Department of Health Research Methods, Evidence, and Impact, McMaster University, Hamilton, Canada; ^f^Michael G. DeGroote Institute for Pain Research and Care

**CONTACT** Lucas Gallo Lucas.gallo@medportal.ca

© 2018 Lucas Gallo, Annie Lok, Andrei Smarandache, Li Wang, Long Ge and Jason W. Busse. Published with license by Taylor & Francis Group, LLC.

This is an Open Access article distributed under the terms of the Creative Commons Attribution License (http://creativecommons.org/licenses/by/4.0/), which permits unrestricted use, distribution, and reproduction in any medium, provided the original work is properly cited.

**Introduction/Aim**: The intercostobrachial nerve (ICBN) is routinely divided during breast cancer surgery involving axillary lymph node dissection (ALND), and sacrifice of this nerve is associated with the development of persistent post-surgical pain. We undertook a systematic review of ICBN sparing procedures to establish the effect on patient-important outcomes after ALND breast cancer surgery.

**Methods**: We searched MEDLINE, EMBASE, and CENTRAL from inception to 2017 for randomized controlled trials that explored ICBN sparing versus sacrifice. We pooled all patient-important outcomes that were reported by >1 trial, and used the GRADE approach to rate the quality of evidence.

**Results**: Of 1,416 articles eligible for initial screening, 7 articles (n = 570 patients) were included. Low quality evidence suggested that ICBN preservation prevents postoperative numbness (RR 0.59; 95% CI 0.44 to 0.78), arm sensory disturbance (RR 0.20; 95% CI 0.07 to 0.58) and persistent post-surgical pain (RR 0.51; 95% CI 0.36 to 0.71); however, nerve sparing also increased the length of surgery (weighted mean difference 8.34 minutes; 95% CI 2.04 to 14.65).

**Discussion/Conclusions**: Limited evidence from 5 small trials, at high risk of bias, suggests that ICBN sparing reduces persistent pain, numbness and sensory disturbance following breast cancer surgery, and increases duration of surgery. Large trials, at low risk of bias, are required to confirm these findings.

##### The impact of inflammatory cytokines and biopsychosocial factors on acute pain after breast cancer surgery

Shannon Goodall^a,*^, Lynn R. Gauthier^b,*^, Madeline Li^c^, Michael Connor^a^, Vincent Chan^d^, Alexandra Easson^e^ and Lucia Gagliese^a^

^a^School of Kinesiology and Health Science, York University, Toronto, ON, Canada; ^b^Department of Family and Emergency Medicine, Laval University, Québec, QC, Canada; ^c^Department of Supportive Care, University Health Network, Toronto, ON, Canada; ^d^Department of Anesthesia and Pain Management, University Health Network, Toronto, ON, Canada; ^e^Department of Surgical Oncology, University Health Network, Toronto, ON, Canada

**CONTACT** Shannon Goodall shannongoodall@gmail.com

^*^ These authors contributed equally to this work.

© 2018 Shannon Goodall, Lynn R. Gauthier, Madeline Li, Michael Connor, Vincent Chan, Alexandra Easson and Lucia Gagliese. Published with license by Taylor & Francis Group, LLC.

This is an Open Access article distributed under the terms of the Creative Commons Attribution License (http://creativecommons.org/licenses/by/4.0/), which permits unrestricted use, distribution, and reproduction in any medium, provided the original work is properly cited.

**Introduction/Aim**: Acute pain after breast cancer surgery (BCS) remains a significant problem and is one of the most consistently reported risk factors for persistent post-operative pain. This study aimed to identify risk factors for pain intensity at rest and with movement, pain qualities and neuropathic pain after BCS.

**Methods**: 86 women scheduled for BCS were recruited. Prior to surgery, demographic, health status, cancer treatment factors, and pain data were collected, psychological questionnaires were completed, and baseline inflammatory cytokines were measured. 24 hours post-BCS, participants completed the Numeric Rating Scale-Rest (NRS-R) and NRS-Movement (NRS-M), Short-Form McGill Pain Questionnaire (SF-MPQ) and Short-Form Neuropathic Pain Questionnaire (SF-NPQ).

**Results**: Backward regression analyses identified significant (p ≤ .05) correlates of each outcome. Correlates of NRS-R were younger age (β = –.25), increased pain catastrophizing (β = .32) and bilateral surgery (β = .29). Correlates of NRS-M were younger age (β = –.21), increased trait anxiety (β = .29), bilateral surgery (β = .24) and mastectomy (β = .25). Correlates of SF-MPQ were increased pain catastrophizing (β = .33), bilateral surgery (β = .28) and previous breast surgery (β = .26). Correlates of SF-NPQ were decreased interleukin-10 (β = –.28) and increased pain catastrophizing (β = .24).

**Discussion/Conclusions**: Each model contained a range of biological, psychological and surgical factors, supporting the biopsychosocial model of pain. Distinct models supported the importance of assessing more than one pain outcome in post-operative pain research. Variables accounting for the most variance in each outcome (pain catastrophizing [NRS-R; SF-MPQ], trait anxiety [NRS-M] and baseline IL-10 [SF-NPQ]) are potentially modifiable. These findings may inform future research on interventions to improve post-operative pain management.

##### Exploring the impact of ECHO in Ontario on primary health care provider’s knowledge sharing about chronic pain: a qualitative study

Naima Salemohamed^a^, Jennifer Stinson^b^, Jane Zhao^a^, Leslie Carlin^c^, Ruth Dubin^d^, Paul Taenzer^e^, Fiona Webster^f^ and Andrea Furlan^g^

^a^University Health Network, Torornto Rehab, Toronto, Canada; ^b^Institute of Health Policy, Management and Evaluation, University of Toronto, Toronto; ^c^Department of Physical Therapy, University of Toronto, Toronto, Canada; ^d^Department of Family medicine, Queens University, Kingston, Canada; ^e^Department of Psychology, University of Calgary, Calgary, Canada; ^f^Dalla Lana School of Public Health, University of Toronto, Toronto, Canada; ^g^Department of Medicine, University of Toronto, Toronto, Canada

**CONTACT** Naima Salemohamed naima.salemohamed@mail.utoronto.ca

© 2018 Naima Salemohamed, Jennifer Stinson, Jane Zhao, Leslie Carlin, Ruth Dubin, Paul Taenzer, Fiona Webster and Andrea Furlan. Published with license by Taylor & Francis Group, LLC.

This is an Open Access article distributed under the terms of the Creative Commons Attribution License (http://creativecommons.org/licenses/by/4.0/), which permits unrestricted use, distribution, and reproduction in any medium, provided the original work is properly cited.

The ECHO chronic pain program is a telementoring (telehealth) platform, which supports Health care providers (HCPs; spokes) in managing their own patients with chronic pain in their home communities, using the expertise of subspecialists (hub). The aims for this project include understanding: a) if HCPs have increased knowledge and skills in pain management and opioid stewardship, b) whether ECHO’s community of practice model impacted HCPs’ knowledge sharing and c) how HCPs in this program gained insights into their motivations and confidence levels in managing this challenging population.

**Methods**: Thirteen qualitative semi-structured interviews were completed with participants who have completed or are still attending ECHO. A representative sample of HCPs were included (a) rural vs. urban settings, (b) those who presented ECHO cases vs. others who chose not to, and (c) different professions of HCPs.

**Results**: Preliminary results have demonstrated that the ECHO chronic pain program supports collaboration by providing a variety of views from the different disciplines, having a respectful space to listen while learning, and supporting the building of links with other HCP through this virtual community. Additionally, HCPs value the different resources available and tools being taught by the expert team.

**Discussion/Conclusions**: These preliminary results suggest that the ECHO chronic pain program has built a supportive network of HCPs. Further analysis will provide the opportunity to understand the reach of ECHO and to learn how ECHO is having an impact at the community level in rural, remote and underserved areas.

##### Microglial pannexin-1 critically underlies opioid withdrawal, but not opioid-induced hyperalgesia or analgesic tolerance

Nicole E. Burma^a^, Heather Leduc-Pessah^a^, Michael Mousseau^a^ and Tuan Trang^a^

Departments of Comparative Biology & Experimental Medicine and Physiology & Pharmacology, Hotchkiss Brain Institute, University of Calgary, Calgary, Canada

**CONTACT** Tuan Trang trangt@ucalgary.ca

© 2018 Nicole E. Burma, Heather Leduc-Pessah, Michael Mousseau and Tuan Trang. Published with license by Taylor & Francis Group, LLC.

This is an Open Access article distributed under the terms of the Creative Commons Attribution License (http://creativecommons.org/licenses/by/4.0/), which permits unrestricted use, distribution, and reproduction in any medium, provided the original work is properly cited.

**Introduction/Aim**: Opioids are essential analgesics for managing pain; however, their use has steadily increased over the past decade. Prolonged opioid use is associated with adverse effects such as physical dependence (characterized by a withdrawal syndrome), analgesic tolerance (loss of the pain relieving effects) and opioid-induced hyperalgesia (a paradoxical increase in pain). We have previously identified the microglial pannexin-1 (Panx1) channel as a novel therapeutic target in opioid withdrawal. In this study, we tested whether blocking microglial Panx1 alleviates morphine analgesic tolerance and hyperalgesia.

**Methods**: To assess the role of microglial Panx1 in opioid tolerance and hyperalgesia, we used a transgenic mouse strain with a targeted deletion of Panx1 from microglia (Cx3cr1-Cre^ERT2^::Panx1^flx/flx^). Opioid analgesic tolerance was established by daily injection of morphine (10 mg/kg) for 7 days, and opioid-induced hyperalgesia was established by injecting escalating doses of morphine twice a day for 5 days. Changes in morphine induced antinociception and hyperalgesia were assessed by measuring tail withdrawal latencies from a constant thermal stimulus.

**Results**: Opioid tolerance was characterized by a decline in morphine antinociception and a loss in morphine analgesic potency. Genetic deletion of microglial Panx1 had no effect on the development of analgesic tolerance. Using the opioid-induced hyperalgesia paradigm, we found that tail withdrawal latencies of wild-type mice progressively decreased over the 5 day treatment period, and genetic deletion of microglial Panx1 had no effect on this decline in response.

**Discussion/Conclusions**: Together, our findings suggest that microglial Panx1 represents a divergent mechanism that preferentially underlies opioid withdrawal, and not opioid analgesic tolerance or hyperalgesia.

##### Pain free laceration repairs using intra-nasal ketamine: DosINK1 - a dose escalation clinical trial

Soha Rached-D’Astous^a^, Benoit Bailey^a^, Christopher Marquis^b^, Marie-Pier Desjardins^a^, Denis Lebel^b^ and Evelyne D-Trottier^a^

^a^Department of Pediatric Emergency, CHU Sainte-Justine, Montreal, Quebec, Canada; ^b^Department of Pharmacy, CHU Sainte-Justine, Montreal, Quebec, Canada

**CONTACT** Soha Rached-d’Astous soharached@gmail.com

© 2018 Soha Rached-D’Astous, Benoit Bailey, Christopher Marquis, Marie-Pier Desjardins, Denis Lebel and Evelyne D-Trottier. Published with license by Taylor & Francis Group, LLC.

This is an Open Access article distributed under the terms of the Creative Commons Attribution License (http://creativecommons.org/licenses/by/4.0/), which permits unrestricted use, distribution, and reproduction in any medium, provided the original work is properly cited.

**Introduction/Aim**: Laceration is common in children presenting to the emergency department. They are often uncooperative related to pain and distressed during repair. Few studies have evaluated intranasal (IN) ketamine for procedural sedation in children with doses ranging from 3 to 9 mg/kg. We sought to evaluate the optimal IN ketamine dose for effective and safe procedural sedation.

**Methods**: A dose escalation trial with an initial dose of 3 mg/kg up to 9 mg/kg of IN Ketamine for laceration repair with suture in children 1 to 12 years in the ED, using a 3 + 3 trial design. For each dose, 3 patients are enrolled. Escalation to the next dose is permitted if sedation is unsuccessful without serious adverse event (SAE). This process is repeated until effective sedation for 6 consecutive patients with a maximum of 1 SAE. The primary outcome is the optimal dose for successful procedural sedation as per the PERC/PECARN consensus criteria.

**Results**: Nine patients have been recruited from March to December 2017 with median age of 2.9 years-old with laceration length of 2 to 5 cm and facial involvement in 55% of cases, respectively. Sedation was successful in 1/3, 1/3 and 3/3 patients at doses of 3, 4, 5 mg/kg respectively, without SAE. Median time to return to baseline status and to discharge were 35 and 98 min, respectively. Study completion is expected in March 2018.

**Discussion/Conclusions**: The results from the dosINK1 trial is a groundwork for future dose-finding study. A multicentric trial (DosINK-2), is already set up to further validate the dose from the current trial.

##### The effects of *MC1R* genetic variants on red hair and pain

Katerina Zorina-Lichtenwalter^a^, Marc Parisien^a^, Ryan N. Lichtenwalter^a^, Andrey Bortsov^b^, Gary Slade^c^, Ronard Dubner^d^, Roger Fillingim^e^, Joel Greenspan^d^, Richard Ohrbach^f^, Charlie Knott^g^, William Maixner^b^ and Luda Diatchenko^a^

^a^Alan Edwards Centre for Pain Research, McGill University, Montreal, Quebec, Canada; ^b^Anesthesiology, Duke University, Durham, USA; ^c^Regional Centre for Neurosensory Disorders, University of North Carolina, Chapel Hill, USA; ^d^Departments of Oral and Maxillofacial Surgery and Neural and Pain Sciences, University of Maryland, Baltimore, USA; ^e^Department of Community Dentistry and Behavioral Science, University of Florida, Gainesville, USA; ^f^Department of Oral Diagnostic Sciences, University of Buffalo, Buffalo, USA; ^g^RTI International, Social- Statistical- and Environment Sciences Survey Research Division, Durham, USA

**CONTACT** Katerina Zorina-Lichtenwalter katerina.lichtenwalter@mail.mcgill.ca

© 2018 Katerina Zorina-Lichtenwalter, Marc Parisien, Ryan N. Lichtenwalter, Andrey Bortsov, Gary Slade, Ronard Dubner, Roger Fillingim, Joel Greenspan, Richard Ohrbach, Charlie Knott, William Maixner and Luda Diatchenko. Published with license by Taylor & Francis Group, LLC.

This is an Open Access article distributed under the terms of the Creative Commons Attribution License (http://creativecommons.org/licenses/by/4.0/), which permits unrestricted use, distribution, and reproduction in any medium, provided the original work is properly cited.

**Introduction/Aim**: Melanocortin-1 receptor, encoded by the gene *MC1R*, has an established role in red hair pigmentation and tanning ability. It has also been linked to pain sensitivity, although the same nonsynonymous (amino acid-changing) single nucleotide polymorphisms (SNPs) have been associated with both greater and lower sensitivity to noxious stimuli. Here we attempt to reconcile these apparently contradicting results by investigating all common SNPs (nonsynonymous as well synonymous SNPs in the regulatory region) in *MC1R*.

**Methods**: We genotyped all common *MC1R* SNPs in a TMD case-control cohort as part of the multi-centre OPPERA (Orofacial Pain: Prospective Evaluation and Risk Assessment) project. Participants underwent clinical assessment and quantitative sensory testing to evaluate sensitivity to thermal stimuli. Then we:

1. analysed for association between *MC1R* SNPs and thermal sensitivity

2. examined *MC1R* haplotypic structure to determine possible correlation effects

**Results**: Our findings show that regulatory region SNP rs3212361 is significantly associated with heat pain sensitivity. Out of six common nonsynonymous “red hair” SNPs, two – rs1805008 and rs885479 – have minor alleles correlated with the protective alleles of the regulatory region SNPs, and the other four – rs1805005, rs2228479, rs1805007, and rs1805009 – have minor alleles correlated with the risk alleles of the regulatory region SNPs.

**Discussion/Conclusions**: While association analyses have identified different SNPs as mediators of red hair and pain sensitivity phenotypes, linkage disequilibrium between them may have led to contradicting findings in prior publications regarding the relationship between red hair and altered pain sensitivity.

##### System xC- knockdown in cancer cells reduces glutamate release and cancer pain

Robert Ungard^a^, Adam Merlo^a^, Tanya Miladinovic^a^, Katja Linher-Melville^a^ and Gurmit Singh^a^

Pathology & Molecular Medicine, McMaster University, Hamilton, ON, Canada

**CONTACT** Robert Ungard ungardr@mcmaster.ca

© 2018 Robert Ungard, Adam Merlo, Tanya Miladinovic, Katja Linher-Melville and Gurmit Singh. Published with license by Taylor & Francis Group, LLC.

This is an Open Access article distributed under the terms of the Creative Commons Attribution License (http://creativecommons.org/licenses/by/4.0/), which permits unrestricted use, distribution, and reproduction in any medium, provided the original work is properly cited.

**Introduction/Aim**: Cancer pain is often under-treated or poorly responsive to therapy, and effective therapies are limited by severe dose-dependent side effects. We have found that malignant cells secrete the neurotransmitter and cell-signaling molecule glutamate via the oxidative stress-related cystine/glutamate antiporter, system xC-. This investigation examines the impact of implanting system xC- knockdown cancer cell lines in animal models of cancer-induced bone pain.

**Methods**: Stable MDA-MB-231 human breast adenocarcinoma xCT knockdown breast cancer cells were generated using siRNA transfection. IPTG-inducible xCT knockdown cells were generated using lentiviral shRNA transduction. Viable clones were screened for xCT knockdown at the transcript, protein, and functional levels. Immunodeficient mice were inoculated intrafemorally with 1 × 10^6^ cells. Behavioural recordings were performed throughout the duration of the animal model. Animal weight bearing, automated von Frey, and limb use were measured. Bone, tumour, and plasma were collected for analysis.

**Results**: Animals implanted with xCT knockdown cancer cell displayed reduced nociceptive behaviours and an extended time until the onset of behavioural evidence of pain.

**Discussion/Conclusions**: These preliminary results suggest that a reduction in glutamate secretion from cancers in bone by inhibition of the system xC- transporter may provide some benefit for treating the often severe and intractable pain associated with bone metastases.

##### Surgical nurses’ and nurse practitioners’ knowledge & attitudes of post-operative pain management: current landscape and future educational opportunities

Salima S. J. Ladak, NP, PhD, ^a^, Jiao Jiang, NP, MN, ^a^, Arlene Buzon-Tan, NP, MN, ^b^, Susan Walker, NP, MN, ^b^, Diana Tamir, MD FRCPC, ^c^, Hance Clarke, MD and PhD, FRCPC, ^c^

^a^Toronto General Hospital, Anesthesia and Pain Management, Toronto, Ontario, Canada; ^b^Toronto Western Hospital, Anesthesia and Pain Management, Toronto, Ontario, Canada; ^c^University Health Network, University of Toronto, Toronto, Ontario, Canada

**CONTACT** Salima S. J. Ladak **CONTACT**salima.ladak@uhn.ca Toronto General Hospital, Anesthesia and Pain Management, Toronto, Ontario, Canada

© 2018 Salima S. J. Ladak, Jiao Jiang, Arlene Buzon-Tan and Susan Walker. Published with license by Taylor & Francis Group, LLC.

This is an Open Access article distributed under the terms of the Creative Commons Attribution License (http://creativecommons.org/licenses/by/4.0/), which permits unrestricted use, distribution, and reproduction in any medium, provided the original work is properly cited.

**Introduction/Aim**: This study was designed to describe nurses’ (RNs) and nurse practitioners’ (NPs) knowledge and attitudes related to post-operative pain management. This poster will illustrate key findings and knowledge gaps that were identified, and provide recommendations for future education.

**Methods**: A prospective observational study of RNs (n = 147; aged 20–70 years) and NPs (n = 19; aged 30–70 years) in a multi-site quaternary academic health science centre was conducted. The main outcome measure was the composite score of the Knowledge and Attitude Survey Regarding Pain, and demographic predictors which influenced this outcome.

**Results**: NPs achieved higher overall scores than RNs (80% vs. 75%, η^2^ = 0.02, *p* = 0.04). Average correct scores demonstrating attitude towards pain management were greater than 80% for both groups. Average correct scores related to knowledge of pharmacology, respiratory depression, use of adjunct medications, and equi-analgesia ranged from 42% to 65% for the entire group. Level of education was not a predictor of the composite score (η^2^ = 0.01, *p* = 0.36). However those who participated in at least one educational seminar over the previous 6 months achieved higher scores compared to those who did not (η^2^ = 0.059, *p* = 0.08).

**Discussion/Conclusions**: Knowledge gaps were identified despite NPs and RNs, on average, scoring greater than 80% on attitudes-related questions regarding pain management. Those who participated in educational seminars had higher average scores compared to those who did not. Future educational interventions for both groups can focus on pharmacology of analgesics, anticipated utilization of adjuvant analgesics and opioid-related respiratory depression.

##### The Chronic Pain Network

Norm Buckley^a^, Karen Davis^b^, Luda Diatchenko^c^, Allen Finley^d^, Garry Salisbury^e^, Ian Gilron^f^, Maria Hudspith^g^, Alfonso Iorio^h^, Margot Latimer^i^, Joy MacDermid^j^, Patricia Poulin^k^, Cyril Schneider^l^, Bonnie Stevens^m^, Jennifer Stinson^n^, John R. Sylliboy, Kim Begley^o^ and Megan Groves^p^

^a^Department of Anesthesia, McMaster University, Hamilton, Ontario, Canada; ^b^Department of Surgery & Institute of Medicine Science, University of Toronto, Toronto, Ontario, Canada; ^c^Department of Anesthesia, McGill University, Montreal, Quebec, Canada; ^d^Department of Anesthesia, Pain Management & Perioperative Medicine, Dalhousie University, Halifax, Nova Scotia, Canada; ^e^Ministry of Health and Long-Term Care, Kingston, Ontario, Canada; ^f^Biomedical and Molecular Science, Queen’s University, Kingston, Ontario, Canada; ^g^Pain BC, Vancouver, British Columbia, Canada; ^h^Department Health Research Methods, Evidence, and Impact, McMaster University, Hamilton, Ontario, Canada; ^i^Centre for Pediatric Pain Research, IWK Health Centre, Halifax, Nova Scotia, Canada; ^j^Departments of Surgery and Epidemiology, University of Western Ontario, London, Ontario, Canada; ^k^Department of Anesthesiology and Pain Medicine, University of Ottawa, Ottawa, Ontario, Canada; ^l^Department of Rehabilitation, Université Laval, Quebec, Quebec, Canada; ^m^Department of Nursing Science, University of Toronto, Toronto, Ontario, Canada; ^n^Department of Anesthesia and Pain Management, The Hospital for Sick Children, Toronto, Ontario, Canada; ^o^Chronic Pain Network, Hamilton, Ontario, Canada; ^p^Centre for Pediatric Pain Research, IWK Health Centre, Halifax, Nova Scotia, Canada

**CONTACT** Norm Buckley buckleyn@mcmaster.ca

© 2018 Kim Begley, Norm Buckley, Karen Davis, Luda Diatchenko, Allen Finley, Garry Salisbury, Ian Gilron, Maria Hudspith, Alfonso Iorio, Margot Latimer, Joy MacDermid, Patricia Poulin, Cyril Schneider, Bonnie Stevens, Jennifer Stinson and Megan Groves. Published with license by Taylor & Francis Group, LLC.

This is an Open Access article distributed under the terms of the Creative Commons Attribution License (http://creativecommons.org/licenses/by/4.0/), which permits unrestricted use, distribution, and reproduction in any medium, provided the original work is properly cited.

**Introduction/Aim**: Despite pain being a common problem, there is a small proportion of total research funding in Canada dedicated to understanding and treating it. Patients and patient organizations are integral to leading the demand for change. The Chronic Pain Network was created under the auspices of CIHR’s Strategies for Patient Oriented Research, to enhance patient engagement and efficient knowledge translation in research in pain and to establish a formal national research network. Its goal is to change the way pain is understood and treated in Canada.

**Methods**: The Chronic Pain Network supports its overarching goal through funding innovative research, supporting training and mentoring efforts and creating new opportunities for knowledge translation through KT initiatives, a Registry Working Group, a Clinical Research Network and by using resources available through the provincial SPOR SUPPORT Units. Patients are engaged in Network activities as members of the governance structure, contributing to Network projects and helping set Network priorities, with an Indigenous Health Research Advisory committee supporting the Network to help guide relationships between health researchers and Indigenous communities.

**Results**: Network initiatives include Pain+ CPN, a curated database of pain related articles, reviewed by patients and professionals, that identify relevant content for knowledge translation; genetic analysis of patients included in the Quebec Pain Registry to create a large scale database and opportunities for future research; a National Pain Registry, based upon the model of the Quebec Pain Registry; 26 funded Patient Oriented Research projects, reviewed twice yearly by means of a template developed with patient partners, and Training and Mentoring activities.

**Discussion/Conclusions**: Patients can and should play a meaningful role in the advancement of pain research.

By working in collaboration with patients and partnering with pain-centric organizations, we can guide the future of pain research in Canada, i.e. adapted to the needs of those living with pain.

##### Mechanosensitive ion channels in articular nociceptors contribute to osteoarthritis pain

Marine Christin^a^, Billy Haitian He^a^, Stephanie Mouchbahani-Constance^a^, Albena Davidova^a^, Anastasia Cheng^b^, Inés Colmegna^c^ and Reza Sharif Naeini^a^

^a^Physiology Department, McGill University, Montreal, Quebec, Canada; ^b^Division of Experimental Medicine, McGill University, Montreal, Quebec, Canada; ^c^Division of Rheumatology, Department of Medicine, McGill University Health Center, Montreal, Quebec, Canada

**CONTACT** Marine Christin marine.christin@mcgill.ca

© 2018 Marine Christin, Billy Haitian He, Stephanie Mouchbahani-Constance, Albena Davidova, Anastasia Cheng, Inés Colmegna and Reza Sharif Naeini. Published with license by Taylor & Francis Group, LLC.

This is an Open Access article distributed under the terms of the Creative Commons Attribution License (http://creativecommons.org/licenses/by/4.0/), which permits unrestricted use, distribution, and reproduction in any medium, provided the original work is properly cited.

Osteoarthritis (OA) is a chronic debilitating disease affecting 5 million Canadians and causing over $30 billion in direct and indirect costs. Pain is the major complaint of OA patients and is presently inadequately managed. It manifests as mechanical allodynia, a painful response to innocuous stimuli such as joint movement. Allodynia is mostly due to the sensitization of articular nociceptors to mechanical stimuli. These nociceptors respond to noxious mechanical stimuli applied to their terminals via the expression of depolarizing high-threshold mechanosensitive ion channels (MSCs) that convert painful mechanical forces into electrical signals. In this study, we examined the contribution of MSCs to mechanical allodynia in a mouse model of OA.

We recorded MSC activity in knee-innervating nociceptors from naïve and OA mice and discovered that OA nociceptors have greater mechanically-activated currents. Furthermore, their activation threshold is greatly reduced, causing their opening at significantly lower stimulus intensities. Consequently, nociceptors are activated by mild mechanical stimuli. These channels are reversibly inhibited by the selective MSC inhibitor GsMTx4, and the intra-articular injection of this peptide significantly reduced mechanical allodynia in OA mice. This indicates MSCs contribute to OA pain. To examine whether factors in synovial fluids sensitize these channels, we incubated nociceptors with synovial fluids from OA patients. Our data demonstrate enhanced MSC currents in nociceptors exposed to OA synovial fluids.

Overall, our results suggest that MSCs are sensitized during OA and directly contribute to mechanical allodynia. They therefore represent potential therapeutic targets in the treatment of OA pain.

**Introduction/Aim**: Osteoarthritis (OA) is a disabling and highly prevalent condition affecting millions worldwide. Pain is the most important, yet least understood, symptom of OA. It manifests as a hypersensitivity to mechanical stimuli such as joint palpation or movement. Although it is understood that, during OA, nociceptors (pain-sensing neurons) become sensitized to mechanical stimuli and then can be activated by innocuous stimuli, the underlying mechanisms remain poorly understood. Our hypothesis is that mechanosensitive ion channels (MSCs), a type of membrane protein expressed in nociceptors and responsible for sensing mechanical stimuli, are sensitized during OA. The aim of this project is to identify, using a combination of electrophysiology, histology, and behavioral approaches, the molecular bases of mechanical hypersensitivity observed in a mouse model of osteoarthritis pain.

**Methods**: We used intra-articular sodium mono-iodoacetate (MIA) injection as a model of OA. Histological damage following MIA injection was quantified and primary mechanical allodynia was monitored using the knee-bend test. Mechanically-evoked currents of knee-innervating nociceptors were recorded by single-channel patch clamp electrophysiology and dorsal horn neuronal activation was assessed by Fos immunoreactivity.

**Results**: In this study we show that in nociceptors from OA mice, MSCs’ activation threshold is greatly reduced, causing their opening at significantly lower stimuli intensities. Therefore, nociceptors are activated by non-noxious mechanical stimuli. These channels can be reversibly inhibited by a selective MSC inhibitor, GsMTx4, and the intra-articular injection of this peptide significantly reduced the activation of dorsal horn nociceptive circuits as well as primary allodynia in OA mice. Furthermore, we show that incubating nociceptors with synovial fluids from OA patients also increased MSC currents.

**Discussion/Conclusions**: These results suggest that MSCs during OA are sensitized and directly contribute to mechanical allodynia in this pathology. Thus, MSCs are promising therapeutic targets in the treatment of OA pain.

##### Evaluation of vaso-occlusive crisis management with patient controlled analgesia in children with sickle cell disease requiring admission

Claire Arbitre^a^, Yves Pastore^b^, Nancy Robitaille^b^, Benoit Bailey^a^, Annie Viau^c^, Marie-Joelle Bergeron^d,e^, Niina Kleiber^e^, Edith Villeneuve^d,f^ and Evelyne D. Trottier^a^

^a^Division of Emergency Medicine, CHU Sainte-Justine, Université de Montréal, Montréal, Canada; ^b^Division of Hematology, CHU Sainte-Justine, Université de Montréal, Montréal, Canada; ^c^Department of Pharmacy, CHU Sainte-Justine, Université de Montréal, Montréal, Canada; ^d^Pain team, CHU Sainte-Justine, Université de Montréal, Montréal, Canada; ^e^Department of Pediatric, CHU Sainte-Justine, Université de Montréal, Montréal, Canada; ^f^Department of Anesthesiology, CHU Sainte-Justine, Université de Montréal, Montréal, Canada

**CONTACT** Claire Arbitre clairearbitre@yahoo.fr Division of Emergency Medicine CHU Sainte-Justine, Université de Montréal, Montréal

© 2018 Claire Arbitre, Yves Pastore, Nancy Robitaille, Benoit Bailey, Annie Viau, Marie-Joelle Bergeron, Niina Kleiber, Edith Villeneuve and Evelyne D. Trottier.Published with license by Taylor & Francis Group, LLC.

This is an Open Access article distributed under the terms of the Creative Commons Attribution License (http://creativecommons.org/licenses/by/4.0/), which permits unrestricted use, distribution, and reproduction in any medium, provided the original work is properly cited.

**Background**: Vaso-occlusive crisis (VOC) is one of the most distressing occurrences in patients with sickle cell disease (SCD). Patient controlled analgesia (PCA) is recommended by NIH and expert opinions favor its early use.

**Objective**: We aim to review the use of PCA in patients with VOC and to evaluate if its early use is associated with faster pain control and reduced length of stay (LOS).

**Methods**: This retrospective single center study included all pediatric patients admitted and treated with PCA for a severe VOC from 2010 to 2016. “Early” use was defined as start of PCA within 48 hours of arrival in the emergency department (ED) and “late” use after 48 hours. Time to reach adequate analgesia was defined as OUCHER, verbal scale or Faces Pain Scale < 5/10 obtained twice consecutively in a 4-hours interval. Time to reach adequate analgesia and LOS were compared between early-PCA and late-PCA groups.

**Results**: A total of 46 patients presented 87 episodes of VOC treated with PCA during the study. Sixty-three episodes (72%) were treated with early-PCA and 24 (28%) with late-PCA. Both groups were comparable in terms of age (12.0 vs 13.2 years old), gender (54% female vs 62%), hemoglobin phenotype (79.4% HbSS vs 79.2%), but median pain score at admission was higher in early-PCA than in late-PCA (9/10 vs 7/10, median difference 1 (95% CI 0, 2). Early-PCA was associated with a median reduction in LOS of 3.4 days (95% CI -4.9, -1.9) (median early-PCA LOS 6.8 vs late-PCA 10.3 days). Time to reach analgesia could be evaluated only in a subset of patients (21 in Early-PCA and 11 in late-PCA group). Although time to reach adequate analgesia tended to be shorter in the early-PCA group, it was not statistically different: median 101.5 hours vs 143.1 hours, difference of 41.7 (95% CI −4.0, 72.5).

Side effects were observed during 29 (33.3%) PCA treatments (22/63 (34.9%) episodes in early-PCA, 9/24 (37.5%) in late-PCA group) among which 16 (18.6%) were significant adverse events. These were observed in 15 patients who required interventions: 2 desaturations requiring oxygen without intubation, 8 neurologic abnormalities (hallucinations, visual abnormalities, no stroke), 6 urinary retentions.

**Conclusion**: Early use of PCA for severe VOC was associated with a reduced length of hospital stay despite that these patients had higher pain score on admission. Prospective studies are needed to support these positive outcomes.

##### Quality evaluation of a web-based neonatal pain assessment learning tool in Portuguese (Brazil): the Neonatal Pain Assessment Program (PAD-Neo)

Fernanda Felipe Ferreira Da Silva^a^, Taine Costa^a^, Heloísa Helena Ciqueto Peres^a^, Amélia Fumiko Kimura^a^, Elysangela Dittz Duarte^b^, Thaila Correa Castral^c^ and Mariana Bueno^d^

^a^Department of Maternal-Child and Mental Health Nursing, School of Nursing of the University of Sao Paulo, Sao Paulo, SP, Brazil; ^b^School of Nursing of Federal University of Minas Gerais, Maternal-child Nursing and Public Health Department, Belo Horizonte, MG, Brazil; ^c^Department of Nursing, Faculty of Nursing of Federal University of Goias, Goiânia, GO, Brazil; ^d^The Hospital for Sick Children, Child Health Evaluative Sciences, Toronto, ON, Canada

**CONTACT** Fernanda Felipe Ferreira da Silva fernanda.felipe.silva@usp.br

© 2018 Fernanda Felipe Ferreira Da Silva, Taine Costa, Heloísa Helena Ciqueto Peres, Amélia Fumiko Kimura, Elysangela Dittz Duarte, Thaila Correa Castral and Mariana Bueno. Published with license by Taylor & Francis Group, LLC.

This is an Open Access article distributed under the terms of the Creative Commons Attribution License (http://creativecommons.org/licenses/by/4.0/), which permits unrestricted use, distribution, and reproduction in any medium, provided the original work is properly cited.

**Introduction/Aim**: The Neonatal Pain Assessment Program (Programa de Avaliação da Dor Neonatal - PAD-Neo) is a web-based learning tool that aims to provide health sciences students and health care professionals with specific knowledge and skills on pain assessment. Educational material includes podcasts, presentations, summaries, image-based (videos and photos) exercises, and discussion forums. Our aim was to evaluate the quality of PAD-Neo.

**Methods**: Twenty-four experts on neonatal care, neonatal and/or pediatric pain, and education were invited to participate. A validated 20-items tool was used. For each item scores range from 0 to 1, and values ≥0.70 indicate high quality. This study was approved by local research ethics committee (#41048915.8.0000.5392).

**Results**: Items evaluated included navigation (mean score 0.92), clarity of information (0.97), ease location of information (0.89), relevance of content (0.96), contextualization (0.94), correction of content (0.98), multiple windows (0.97), intuitive interactions (0.96), ease of use (0.94), ease of return (0.92), ergonomics (0.94), design (0.96), labels (0.93), audiovisual resources (0.85), references (0.93), interactivity (0.82), management of errors (0.85), help resources (0.81), quality of information (0.96) and portability (0.97). Hence all average scores were ≥0.70, no significant changes were made to the tool. Minor suggestions were incorporated, such as increasing the amount of reading resources written in Brazilian Portuguese.

**Discussion/Conclusions**: The PAD-Neo is considered a high quality educational tool. Future studies are required to evaluate the impact of the PAD-Neo in educational outcomes such as users’ knowledge acquisition, and in neonatal pain outcomes such as documentation of pain assessment in clinical settings.

##### Developing a screening measure for pediatric neuropathic pain

Jennifer Stinson^a^, Kathryn A. Birnie^a^, Chitra Lalloo^b^, Naiyi Sun^c^, Amos Hundert^b^, Cynthia Nguyen^b^, Lauren Harris^b^ and Fiona Campbell^c^

^a^Lawrence S. Bloomberg Faculty of Nursing, University of Toronto, Child Health Evaluative Sciences, The Hospital for Sick Children, Toronto, Canada; ^b^Child Health Evaluative Sciences, The Hospital for Sick Children, Toronto, Canada; ^c^Department of Anesthesia, The Hospital for Sick Children, Department of Anesthesia and Pain Medicine, University of Toronto, Toronto, Canada

**CONTACT** Jennifer Stinson Jennifer.stinson@sickkids.ca

© 2018 Jennifer Stinson, Kathryn A. Birnie, Chitra Lalloo, Naiyi Sun, Amos Hundert, Cynthia Nguyen, Lauren Harris and Fiona Campbell. Published with license by Taylor & Francis Group, LLC.

This is an Open Access article distributed under the terms of the Creative Commons Attribution License (http://creativecommons.org/licenses/by/4.0/), which permits unrestricted use, distribution, and reproduction in any medium, provided the original work is properly cited.

**Introduction/Aim**: Early and accurate detection of neuropathic pain (NeP) is critical for its diagnosis and treatment. Although valid NeP screening measures exist in adults, there is no measure with broad application to pediatric populations. Our aim is to develop, evaluate, and disseminate the first NeP screening measure specifically for children and adolescents.

**Methods**: Two phases of measure development are complete: (1) a systematic review of studies describing the development or validation of existing NeP measures; and (2) an online modified Delphi procedure to generate and refine potential items describing clinical signs and symptoms for the pediatric NeP measure. A steering committee generated 8 items to be refined by an international group of multidisciplinary pediatric chronic pain experts across two rounds of the Delphi process (n = 23 round one; n = 27 round two).

**Results**: The systematic review identified 7 measures for adults. Five of the 7 measures had strong psychometric properties and were commonly used. The Delphi process confirmed a definition of NeP and relevant clinical signs and symptoms. Respondents reported that patients with NeP symptoms represent <25–75% of the pediatric patients they see annually. Depending on the item, 48–91% of respondents identified the 8 potential items describing clinical signs and symptoms of NeP as ‘relevant’ or ‘very relevant’ for inclusion. Respondents suggested 14 additional items for consideration.

**Discussion/Conclusions**: A consensus conference is planned to: (1) finalize the items and wording; (2) devise a research plan to evaluate psychometric properties and clinical utility of the measure; and (3) develop a knowledge translation strategy.

##### Gender differences in subjective pain experience and coping strategies in patients with inflammatory bowel disease

Valentina Mihajlovic^a^, Katherine Fretz^a^, Madelaine Gierc^a^, Michael Beyak^b^, Laura Katz^c^ and Dean Tripp^a^

^a^Department of Psychology, Queen’s University, Kingston, Canada; ^b^Division of Gastroenterology, School of Medicine, Queen’s University, Kingston, Canada; ^c^Michael G DeGroote Pain Clinic, McMaster University Hospital, Hamilton, Canada

**CONTACT** Valentina Mihajlovic valentina.mihajlovic@queensu.ca

© 2018 Valentina Mihajlovic, Katherine Fretz, Madelaine Gierc, Michael Beyak, Laura Katz and Dean Tripp. Published with license by Taylor & Francis Group, LLC.

This is an Open Access article distributed under the terms of the Creative Commons Attribution License (http://creativecommons.org/licenses/by/4.0/), which permits unrestricted use, distribution, and reproduction in any medium, provided the original work is properly cited.

**Introduction/Aim**: Inflammatory bowel disease (IBD) describes a group of conditions that inflame the lining of the gastrointestinal tract, and often result in chronic pain. Coping strategies – illness-focused (i.e., avoidance) or wellne ss-focused (i.e., confrontation) – are a cognitive-behavioural response associated with pain. While previous research has found gender differences regarding coping strategies, this has been overlooked in IBD. The present study compares subjective pain experience and coping strategies in men and women with IBD.

**Methods**: Patients with physician-diagnosed IBD were recruited from a tertiary care clinic, and completed two questionnaires: 294 patients (122 men, 172 women) completed the Short Form-McGill Pain Questionnaire (SF-MPQ), and 289 patients (121 men, 168 women) completed the Brief Chronic Pain Coping Inventory (B-CPCI).

**Results**: Mann-Whitney tests were employed to compare gender differences for pain experience and coping strategies. Subjective pain experience was greater for women (Mdn = 5) than men (Mdn = 3), *U* = −3.378, *p* < 0.01. Women (Mdn = 11) employed significantly more illness-focused coping strategies than men (Mdn = 4); *U* = −3.348, *p* < 0.01. No significant difference between genders was found in use of wellness-focused coping strategies (men: Mdn = 15; women: Mdn = 16); *U* = −1.202, *p* = 0.229.

**Discussion/Conclusions**: Women with IBD reported significantly higher subjective pain and used significantly more illness-focused coping strategies than men. This suggests that an important area for IBD patient care involves teaching patients, especially women, about the differences in outcomes related to use of different coping strategies.

##### The chronic pain network and Indigenous health research

Margot Latimer^a^, John R. Sylliboy^a^, Sharon Rudderham^a^, Brent Young^b^ and Norm Buckley^c^

^a^Centre for Pediatric Pain Research, IWK Health Centre, Halifax, Nova Scotia, Canada; ^b^Indigenous Student Representative, Chronic Pain Network, Halifax, Nova Scotia, Canada; ^c^Anesthesiology, McMaster University, Hamilton, Ontario, Canada

**CONTACT** Margot Latimer mlatimer@dal.ca

© 2018 Margot Latimer, John R. Sylliboy, Sharon Rudderham, Brent Young and Norm Buckley. Published with license by Taylor & Francis Group, LLC.

This is an Open Access article distributed under the terms of the Creative Commons Attribution License (http://creativecommons.org/licenses/by/4.0/), which permits unrestricted use, distribution, and reproduction in any medium, provided the original work is properly cited.

**KEYWORDS** Indigenous Health; Pain; Two-Eyed Seeing

**Introduction/Aim**: The primary goal of the Indigenous Health Research Advisory committee is to gather community-based knowledge and protocols regarding how research is conducted from a community perspective. This information will help to facilitate the development of authentic partnerships and enhanced understanding between Indigenous communities and health researchers.

**Methods**: The Indigenous Health Research Advisory committee embraces the strengths of both Indigenous and Western knowledge using an approach known as Etuaptmumk, or Two-Eyed Seeing. This framework was originated by Mi’kmaq Elders, Albert & Murdena Marshall of Eskasoni First Nation.

**Results**: Preliminary efforts have been focused on compiling community supplied information, available grey and published academic literature.

**Discussion/Conclusions**: The committee will continue to develop community profiles, identifying research protocols and priorities for individual communities, as well as create an online public database of all identified Indigenous health research resources.

##### A structural equation modeling analysis of the diathesis-stress model of chronic pain and disability 6 months or more after cardiac bypass surgery

Iana Ianakieva^a^, Hance Clarke^b^, Ze’ev Seltzer^c^, George Djaiani^b^, Scott Beattie^b^, Vivek Rao^d^ and Joel Katz^a^

^a^Psychology, York University, Toronto, Canada; ^b^Anaesthesia and Pain Management, Toronto General Hospital, Toronto, Canada; ^c^Dentistry, University of Toronto, Toronto, Canada; ^d^Cardiovascular Surgery, Toronto General Hospital, Toronto, Canada

**CONTACT** Iana Ianakieva iana14@yorku.ca

© 2018 Iana Ianakieva, Hance Clarke, Ze’ev Seltzer, George Djaiani, Scott Beattie, Vivek Rao and Joel Katz. Published with license by Taylor & Francis Group, LLC.

This is an Open Access article distributed under the terms of the Creative Commons Attribution License (http://creativecommons.org/licenses/by/4.0/), which permits unrestricted use, distribution, and reproduction in any medium, provided the original work is properly cited.

**Introduction/Aim**: Chronic pain is associated with a myriad of negative impacts on cardiac patients and on the healthcare system alike, such as increased anxiety and cost of care. Factors that contribute to the transition from acute to chronic pain must be better understood in order to develop and provide better pain management care post-cardiac surgery. The diathesis-stress model, which theorizes that higher anxiety sensitivity, higher fear of pain, higher pain catastrophizing, increased escape/avoidance behaviours, and lower pain self-efficacy are associated with greater chronic pain-related psychosocial disability, was assessed in the current study.

**Methods**: Structural equation modeling was conducted using lavaan in R to evaluate the fit of the diathesis-stress model of chronic pain in a sample of 366 post-cardiac bypass surgery patients.

**Results**: The model fit was adequate, as suggested by the comparative fit index value of .940 (the suggested lower cut-off is .90), and the standardized root mean square residual value of .059 (the suggested upper cut-off is .08). Regression coefficients indicated that higher anxiety sensitivity predicted greater fear of pain and pain catastrophizing. Greater fear of pain predicted higher escape/avoidance, while greater pain catastrophizing predicted lower escape/avoidance. Greater escape/avoidance was subsequently related to higher pain disability. Lastly, increased pain disability was related to greater pain catastrophizing and lower pain self-efficacy, but was not linked to changes in fear of pain.

**Discussion/Conclusions**: The relevance of the diathesis-stress model in explaining the development of pain-related disability suggests that it would be beneficial to generate strategies to mitigate the negative effects of the involved factors for patients undergoing cardiac surgery.

##### Chronic pain network - registry working group

Manon Choinière^a^, Richard Hovey^b^, Norm Buckley^c^, Ian Gilron^d^, Curtis May^e^, Jennifer Stinson^f^ and Mark Ware^g^

^a^Anesthesiology and Pain Medicine/Research Center of the Centre hospitalier de l’Université de Montréal, Université de Montréal, Montreal, Quebec, Canada; ^b^Chronic Pain Network, Patient Perspective Partner, & Faculty of Dentistry, McGill University, Montreal, Quebec, Canada; ^c^Anesthesiology, McMaster University, Hamilton, Ontario, Canada; ^d^Anesthesiology, Queen’s University, London, Ontario, Canada; ^e^Chronic Pain Network, Patient Perspective Partner; ^f^Lawrence S Bloomberg Faculty of Nursing, University of Toronto, Toronto, Ontario, Canada; ^g^Family Medicine, McGill University, Montreal, Quebec, Canada

**CONTACT** Manon Choinière manon.choiniere@umontreal.ca Anesthesiology and Pain Medicine/Research Center of the Centre hospitalier de l’Université de Montréal, Université de Montréal, Montreal, Quebec, Canada

© 2018 Manon Choinière, Richard Hovey, Norm Buckley, Ian Gilron, Curtis May, Jennifer Stinson and Mark Ware. Published with license by Taylor & Francis Group, LLC.

This is an Open Access article distributed under the terms of the Creative Commons Attribution License (http://creativecommons.org/licenses/by/4.0/), which permits unrestricted use, distribution, and reproduction in any medium, provided the original work is properly cited.

**Introduction/Aim**: In Canada, there are presently no national standardized or uniform measures to monitor quality of care and outcomes of patients who suffer from chronic pain (CP). The Chronic Pain Network’s Registry Working Group is mandated to implement across Canada a needs-based minimal set of valid and reliable measures that will be integrated into daily clinical practices and serve clinical, administrative and research purposes.

**Methods**: Methods include the creation of three sub-committees (pediatric, adult, and information technology), the utilization of online Delphi-type surveys and face-to-face meetings, and the identification of existing/planned CP patient registries and multidisciplinary pain treatment clinics across Canada in collaboration with the Canadian Agency for Drugs and Technologies in Health.

**Results**: The Pediatric Pain Registry has been created and funding from the Health Ministry in Ontario has been obtained to pilot it at the SickKids Hospital with the Centre for Healthcare Organizational and Innovation Research (CHOIR) electronic platform. The minimal domains/subdomains and measurement tools that will constitute the core of the Adult Pain Registry are in the process of being selected.

**Discussion/Conclusions**: The Pediatric and Adult Pain Registries will help clinicians and decision-makers in their efforts to improve the care and condition of CP patients. At the same time, these registries will set the basis of a Canadian integrated clinical research infrastructure that will be most useful for conducting for rapid multi-site studies, large RCTs, and other types of projects aiming at predicting patient prognosis and treatment.

##### Buzzy device for pain management in children during needle-related procedures: a systematic review

Ariane Ballard^a^, Christelle Khadra^a^, Samara Adler^b^, Evelyne Doyon-Trottier^c^ and Sylvie Le May^a^

^a^Faculty of Nursing, University of Montreal, Montreal, Quebec, Canada; ^b^Faculty of Medicine, University of Montreal, Montreal, Quebec, Canada; ^c^Division of Emergency Medicine, Department of Pediatrics, CHU Sainte-Justine, Montreal, Quebec, Canada

**CONTACT** Ariane Ballard ariane.ballard@umontreal.ca

© 2018 Ariane Ballard, Christelle Khadra, Samara Adler, Evelyne Doyon-Trottier and Sylvie Le May. Published with license by Taylor & Francis Group, LLC.

This is an Open Access article distributed under the terms of the Creative Commons Attribution License (http://creativecommons.org/licenses/by/4.0/), which permits unrestricted use, distribution, and reproduction in any medium, provided the original work is properly cited.

**Introduction/Aim**: Needle-related procedures are an important source of pain in children in medical settings. The Buzzy® device consisting of a vibrating motor and a set of ice wings has been developed as a solution for pain management when placed above the needle-insertion site. Studies examining its effect on needle-related pain in children showed beneficial outcomes. However, a systematic review is needed to critically appraise its quality and pooled effect. The aim of this systematic review is to examine evidence for efficacy of the Buzzy® device for needle-related procedural pain in children.

**Methods**: Electronic databases search was conducted from inception to December 2017 for randomized controlled trials using the Buzzy® device for pain management in children undergoing needle-related procedures. Selection of studies, data extraction, and assessment of risk of bias and quality of evidence was independently performed by two reviewers and consensus was reached by discussion. Quantitative (random-effects meta-analysis) and qualitative analysis were conducted.

**Results**: Nine studies with 1138 participants aged between 3 to 18 years old were included in the analysis. Six studies compared the Buzzy® device to “no intervention” for procedural pain (SMD: −1.11; 95%CI:-1.52, −0.70). Additional studies compared the device to vapocoolant spray (n = 1), topical anesthetic (n = 1), and distraction cards (n = 1).

**Discussion/Conclusions**: The Buzzy® device seems to be a promising intervention for procedural pain management in children. However, the presence of significant heterogeneity and low-quality evidence limits these findings. Additional high quality studies are needed to conclude to its efficacy.

##### The chronic pain network and training & mentoring

Bonnie Stevens^a^, Carley Ouellette^b^, Renée El-Gabalawy^c^, Lesley Singer^d^, Alfonso Iorio^e^, Christine Chambers^f^, Manon Chioniere^g^, Margot Latimer^h^, Michael McGillion^i^, Jeffrey Mogil^j^, Judy Watt-Watson^a^, Renata Musa^k^, Kim Begley^l^ and Norm Buckley^m^

^a^Faculty of Nursing, University of Toronto, Toronto, Ontario, Canada; ^b^Patient Partner, Chronic Pain Network, Hamilton, Ontario, Canada; ^c^Departments of Clinical Health Psychology and Anesthesia & Perioperative Medicine, University of Manitoba, Winnipeg, Manitoba, Canada; ^d^Patient Partner, Chronic Pain Network, Montreal, Quebec, Canada; ^e^Health Research Methods, Evidence, and Impact, McMaster University, Hamilton, Ontario, Canada; ^f^Departments of Pediatrics and Psychology & Neurscience, Dalhousie University, Halifax, Nova Scotia, Canada; ^g^Anesthesiology and Pain Medicine/Research Center of the Centre hospitalier de l’Université de Montréal, Université de Montréal, Montreal, Quebec, Canada; ^h^Centre for Pediatric Pain Research, IWK Health Centre, Halifax, Nova Scotia, Canada; ^i^Cardiovascular Nursing Research, McMaster University, Hamilton, Ontario, Canada; ^j^Department of Psychology, McGill University, Montreal, Quebec, Canada; ^k^Centre for the Study of Pain, University of Toronto, Toronto, Ontario, Canada; ^l^Managing Director, Chronic Pain Network, Hamilton, Ontario, Canada; ^m^Anesthesiology, McMaster University, Hamilton, Ontario, Canada

**CONTACT** Bonnie Stevens bonnie.stevens@sickkids.ca

© 2018 Bonnie Stevens, Carley Ouellette, Renée El-Gabalawy, Lesley Singer, Alfonso Iorio, Christine Chambers, Manon Chioniere, Margot Latimer, Michael McGillion, Jeffrey Mogil, Judy Watt-Watson, Renata Musa, Kim Begley and Norm Buckley. Published with license by Taylor & Francis Group, LLC.

This is an Open Access article distributed under the terms of the Creative Commons Attribution License (http://creativecommons.org/licenses/by/4.0/), which permits unrestricted use, distribution, and reproduction in any medium, provided the original work is properly cited.

**Introduction/Aim**: A key priority of the Chronic Pain Network is to enhance training of highly qualified professionals to increase capacity for chronic pain treatment and research across Canada.

**Methods**: The Training & Mentoring committee supports five established training initiatives that address pre-licensure, graduate and post graduate levels of education.

**Results**: Increased engagement of patients in collaborative chronic pain research initiatives; strengthened research capacity in chronic pain, building on existing expertise; and strengthening linkages between trainees, mentors, clinicians, consumers, and researchers.

**Discussion/Conclusions**: Outcomes continue to be analyzed to ensure objectives are met, and the Network will continue to work with patients to enhance all training and mentoring initiatives and events.

##### Molecular pathophysiology of low back pain chronicity

Nehme El-Hachem^a^, Concetta Dagostino^b^, Marc Parisien^a^, Massimo Allegri^b^ and Luda Diatchenko^a^

^a^Alan Edwards Centre for Research on Pain, McGill University, Montreal, Canada; ^b^Department of Medicine and Surgery, University of Parma, Parma, Italy

**CONTACT** Nehme El-Hachem nehme.el-hachem@mcgill.ca

© 2018 Nehme El-Hachem, Concetta Dagostino, Marc Parisien, Massimo Allegri and Luda Diatchenko. Published with license by Taylor & Francis Group, LLC.

This is an Open Access article distributed under the terms of the Creative Commons Attribution License (http://creativecommons.org/licenses/by/4.0/), which permits unrestricted use, distribution, and reproduction in any medium, provided the original work is properly cited.

**Introduction**: Low back pain (LBP) is a serious public health issue creating a significant socio-economic burden. Given that molecular pathophysiology of the transition from acute to chronic LBP is not understood, our study sought to describe differences between individuals with persistent or resolved LBP pain after an acute episode at the genome-wide transcriptome level.

**Methods**: A cohort of 100 Caucasian patients, male and female older than 18 years old, with acute LBP was evaluated in two times: t0, within 6 weeks of the first acute pain episode, and t1, at 3 months after the enrollment time. Fifty patients still reported pain during the follow-up at 3 months and another 50 patients did not. RNA was extracted from whole blood and full transcriptome sequencing was performed. Bioinformatics analyses included the following steps: A) Analysis of differentially expressed genes; B) Pathway Enrichment analysis to identify biological pathways that are over-represented in the set of differentially expressed genes

**Results**: The results of our bioinformatics analysis suggest that patients who resolved pain from acute LBP episode at 3 months of follow-up exhibited a substantial change of their corresponding gene expression profiles compared to those who transitioned to chronic LBP. Importantly, our pathway analysis showed a significant role of the innate and adaptive immune system in the modulation of pain outcomes.

**Conclusions**: mRNA expression level alterations could provide insights on molecular remodeling dynamics in chronic low back pain. Validation in animal models would consolidate our findings.

##### Reduced Physical Performance Assessed with Objective Measurements Detects Kinesiophobia After Spine Surgery in Adolescents

Diana-Luk Ye^a^, Sheila Bote^b^, Neil Saran^c^, Jean A. Ouellet^d^ and Catherine E. Ferland^e^

^a^McGill University, Experimental Surgery, Montreal, Quebec, Canada; ^b^Shriners Hospital for Children-Canada, McGill Scoliosis & Spine Group, Clinical Research – Orthopaedic Surgery, Montreal, Quebec, Canada; ^c^Shriners Hospital for Children-Canada, McGill University Health Centre, Pediatric Surgery, Montreal, Quebec, Canada; ^d^Shriners Hospital for Children-Canada, McGill University Health Centre, Orthopedic Surgery, Montreal, Quebec, Canada; ^e^Shriners Hospitals for Children-Canada, McGill University Health Centre, Anesthesia, Montreal, Quebec, Canada

**CONTACT** Diana-Luk Ye diana-luk.ye@mail.mcgill.ca

© 2018 Diana-Luk Ye, Sheila Bote, Neil Saran, Jean A. Ouellet and Catherine E. Ferland. Published with license by Taylor & Francis Group, LLC.

This is an Open Access article distributed under the terms of the Creative Commons Attribution License (http://creativecommons.org/licenses/by/4.0/), which permits unrestricted use, distribution, and reproduction in any medium, provided the original work is properly cited.

**Introduction/Aim:** Spinal fusion surgery with instrumentation is an invasive and extensive surgery that predisposes children to high risks of developing kinesiophobia (fear of movement) and morbidities like disability. Currently, kinesiophobia is measured by self-reporting. To improve surgical recovery, an objective detection is needed. The aims of the study were to identify kinesiophobia with the Tampa Scale for Kinesiophobia (TSK) questionnaire and to detect kinesiophobia with an objective physical assessment, and to associate the objective tests to the subjective measure.

**Methods:** Twenty 10-19 year-old patients with Adolescent Idiopathic Scoliosis scheduled to undergo surgery were enrolled. Before and six weeks after surgery, patients answered the TSK questionnaire and performed a physical assessment comprised of sit&reach, supine bridge and prone alternating arm&leg raises tests. Via objective grading component, a higher score indicated kinesiophobia presence. Correlation, two-way ANOVA and paired t-tests analyses were performed.

**Results:** After surgery, an increase was observed in the TSK score and sub-score activity avoidance (p=0.007 and 0.002 respectively). A diminished physical performance was observed after surgery with the total score of all physical assessments (p<0.001), the scores for supine bridge (p=0.02) and arm&leg raise (p<0.001). An association between the objective physical assessments and the subjective TSK score was observed when the cohort was divided according to the objective assessments’ median score of 4. The sub-group who performed worse after surgery was also having greater activity avoidance (p=0.008).

**Discussion/Conclusions:** Results suggest that a physical assessment performed in the clinic could help identify who is at risk of developing kinesiophobia after surgery to improve recovery.

##### Improving working memory and pain inhibition in healthy older persons using neuromodulation of left dorsolateral prefrontal cortex

Zoha Deldar^a^, Nabi Rustamov^a^, Isabelle Blanchette^b^ and Mathieu Piche^a^

^a^Chiropractic, Trois-Rivières, Québec, Canada, and Groupe de Recherche en Cognition, Neuroscience, Affect et Comportement (CogNAC), Université du Québec à Trois-Rivières, Trois-Rivières, Canada; ^b^Psychology, Trois-Rivières, Québec, Canada. and Groupe de Recherche en Cognition, Neuroscience, Affect et Comportement (CogNAC), Université du Québec à Trois-Rivières, Trois-Rivières, Canada

**CONTACT** Mathieu Piche Zoha.deldar@uqtr.ca Chiropractic, Trois-Rivières, Québec, Canada, and Groupe de Recherche en Cognition, Neuroscience, Affect et Comportement (CogNAC), Université du Québec à Trois-Rivières, Trois-Rivières, Québec, Canada

© 2018 Zoha Deldar, Nabi Rustamov, Isabelle Blanchette and Mathieu Piche. Published with license by Taylor & Francis Group, LLC.

This is an Open Access article distributed under the terms of the Creative Commons Attribution License (http://creativecommons.org/licenses/by/4.0/), which permits unrestricted use, distribution, and reproduction in any medium, provided the original work is properly cited.

The aim of the present study was to examine whether transcranial Direct Current Stimulation (tDCS) of the left dorsolateral prefrontal cortex could enhance pain inhibition by improving working memory in older persons. Fifteen volunteers (7 women, 8 men; mean age: 64 ± 4.41 y.o.) participated in two tDCS sessions during which the n-back task was performed with two levels of working memory load. To evoke nociceptive flexion reflex, painful stimulation was delivered at the ankle during n-back task. The experiment included five counterbalanced conditions (pain alone and 0-back or 2-back with or without pain) performed twice during each session. Compared with the pre-tDCS baseline, anodal tDCS decreased response times and improved pain inhibition by working memory in the 2-back tasks (p < 0.01), while sham tDCS produced no significant effect (all p > 0.3). In addition, anodal tDCS and sham tDCS did not significantly change nociceptive flexion reflex amplitude. These results indicate that neuromodulation of the left dorsolateral prefrontal cortex enhances pain inhibition in older persons by improving working memory, independently of descending inhibition of spinal nociceptive activity.

**Introduction/Aim**: The aim of the present study was to examine whether transcranial Direct Current Stimulation (tDCS) of the left dorsolateral prefrontal cortex could enhance pain inhibition by improving working memory in older persons.

**Methods**: Fifteen volunteers (7 women, 8 men; mean age: 64 ± 4.41 y.o.) participated in two tDCS sessions during which a n-back task was performed with two levels of working memory load. To evoke nociceptive flexion reflex, painful stimulation was delivered at the ankle during n-back task. The experiment included five counterbalanced conditions (pain alone and 0-back or 2-back with or without pain) performed twice during each session.

**Results**: Compared with the pre-tDCS baseline, anodal tDCS decreased response times and improved pain inhibition by working memory in the 2-back tasks (p < 0.01), while sham tDCS produced no significant effect (all p > 0.3). In addition, anodal tDCS and sham tDCS did not significantly change nociceptive flexion reflex amplitude.

**Discussion/Conclusions**: These results indicate that neuromodulation of the left dorsolateral prefrontal cortex enhances pain inhibition in older persons by improving working memory, independently of descending inhibition of spinal nociceptive activity.

**Funding**: This study was funded by the Natural Science and Engineering Research Council (NSERC) of Canada. The contribution of Zoha Deldar was supported by the Département d’anatomie of UQTR and the Centre en Neuropsychologie et Cognition (CERNEC). The contribution of Nabi Rustamov was supported by Fonds de Recherche du Québec en Nature et Technologie and Fondation de Recherche en Chiropratique du Québec. The contribution of Mathieu Piché was supported by Fonds de Recherche du Québec en Santé and the UQTR Research Chair in Pain Neurophysiology.

##### A systematic review of olfactive stimulation interventions to manage procedural pain in preterm and full-term neonates

Gwenaelle De Clifford-Faugère^a^, Andréane Lavallée^a^, Christelle Khadra^a^ and Marilyn Aita^a^

Faculty of Nursing, University of Montreal, Montreal, QC, Canada

**CONTACT** Gwenaelle De Clifford-Faugère gwenaelle.de.clifford@umontreal.ca Faculty of Nursing, University of Montreal, Montreal, QC, Canada.

© 2018 Gwenaelle De Clifford-Faugère, Andréane Lavallée, Christelle Khadra and Marilyn Aita. Published with license by Taylor & Francis Group, LLC.

This is an Open Access article distributed under the terms of the Creative Commons Attribution License (http://creativecommons.org/licenses/by/4.0/), which permits unrestricted use, distribution, and reproduction in any medium, provided the original work is properly cited.

**Introduction/Aim**: Preterm and full-term neonates undergo many painful procedures during their hospitalization in the neonatal intensive care unit. Unrelieved and repeated pain can have important repercussions on their motor and intellectual development. Still, pain management interventions are limited for neonates. To our knowledge, no systematic review has evaluated the effectiveness of olfactive stimulation interventions on pain of preterm and full-term neonates. Aim: to evaluate the effectiveness of olfactive stimulation interventions for procedural pain management in preterm and full-term neonates.

**Methods**: An electronic search was conducted in PubMed, Medline, CINAHL, EMBASE, PsycInfo, Web of Science and CENTRAL from inception until June 2017. Selected studies had a RCT or a quasi-experimental design, included preterm or full-term neonates who underwent a painful procedure, evaluated a natural or an artificial odor during the painful procedure. Multisensory interventions were excluded. Selection of studies, data extraction and assessment of risk of bias by the Cochrane’s tool, were done by two independent researchers. Consensus was reached by discussion.

**Results**: Of 3089 screened studies, 15 studies met the inclusion criteria. Ten studies reported evaluating an olfactive stimulation intervention to manage pain in full-term infants and five in preterm infants. To date, data extraction and risk of bias assessment have been completed. Data analysis is ongoing.

**Discussion/Conclusions**: Findings of this systematic review will improve pain management by informing clinical practice and neonatology research on the effectiveness of olfactory stimulation interventions. The optimal goal is to prevent the consequences of repeated and untreated pain in preterm and full-term neonates.

##### Olfactive stimulation intervention with breast milk for managing procedural pain in preterm neonates: acceptability and feasibility

Gwenaelle De Clifford-Faugère^a^, Marilyn Aita^a^, Marjolaine Héon^a^ and Sylvie Le May^a^

Faculty of Nursing, University of Montreal, Montreal, Canada

**CONTACT** Gwenaëlle De Clifford-Faugère gwenaelle.de.clifford@umontreal.ca

© 2018 Gwenaelle De Clifford-Faugère, Marilyn Aita, Marjolaine Héon and Sylvie Le May. Published with license by Taylor & Francis Group, LLC.

This is an Open Access article distributed under the terms of the Creative Commons Attribution License (http://creativecommons.org/licenses/by/4.0/), which permits unrestricted use, distribution, and reproduction in any medium, provided the original work is properly cited.

**Introduction/Aim**: Repeated and untreated pain has consequences for the preterm neonates such as a hypersensitivity to pain, as well as important repercussions on their motor and intellectual development. The use of pharmacological and non-pharmacological interventions is very limited, hence the importance of investigating the effect of a new intervention: the breast milk odor. This pilot study aims to assess feasibility and acceptability of an olfactive stimulation intervention with breast milk on the pain response of preterm neonates.

**Methods**: A pilot study with a single group was conducted with preterm, mothers and nurses. Twelve preterm infants were familiarized to the breast milk odor during the nine preceding hours to heel prick and in addition to standard care during the painful procedure. Questionnaires were administered to mothers (n = 11) and nurses (n = 20).

**Results**: All eligible mothers agreed to participate in the study. The olfactive stimulation intervention was feasible and acceptable for more than 80% of mothers (i. e. all mothers came the day before heel prick to express their milk at the neonatal unit, it was acceptable to use their milk to manage pain). For nurses, acceptability rate ranged from 70% to 100 and feasibility was more than 80% (i. e. quantity of milk, keep the gauze at the desired distance, change it every three hours).

**Discussion/Conclusions**: This research will improve pain management in preterm neonates by leading the conduct a full-scale randomized clinical trial with the purpose of evaluating the effectiveness of this intervention, thus guiding the clinical practice and research in neonatology.

##### Expression of somatostatin (SST) and STTR 2 by masseter muscle ganglion neurons and their associated satellite glial cells

Jason Liu^a^, Ujendra Kumar^a^ and Brian Cairns^a^

Faculty of Pharmaceutical Sciences, University of British Columbia, Vancouver, Canada

**CONTACT** Brian Cairns brcairns@mail.ubc.ca Faculty of Pharmaceutical Sciences, University of British Columbia, 2405 Wesbrook Mall Vancouver, BC V6T 1Z3, Canada

© 2018 Jason Liu, Ujendra Kumar and Brian Cairns. Published with license by Taylor & Francis Group, LLC.

This is an Open Access article distributed under the terms of the Creative Commons Attribution License (http://creativecommons.org/licenses/by/4.0/), which permits unrestricted use, distribution, and reproduction in any medium, provided the original work is properly cited.

**Introduction/Aim**: Systemic administration of octreotide, a peripherally restricted somatostatin (SST) analogue, may decrease the excitability of nociceptors. We tested the hypothesis that SST and SSTR2 are expressed by a subset of masseter muscle ganglion neurons and their associated satellite glial cells (SGCs).

**Methods**: Masseter muscle ganglion neurons were identified by injection of the fluorescent dye fast blue into the masseter muscle of 10 Sprague Dawley rats (5 male, 5 female). Trigeminal ganglion sections were stained with antibodies against SST14, SSTR2 and glutamine synthase (a marker of SGCs). Sections were examined with a Leica confocal microscope. Statistical significance was assessed with Mann-Whitney tests.

**Results**: SST14 and SSTR2 were expressed in 8% and 5%, respectively, of the 621 ganglion neurons examined. SSTR2 receptors were expressed by 43% of ganglion neurons that contained SST. There was a trend towards higher expression of SST14 in ganglion neurons from male rats (p = 0.056). SST14 and SSTR2 were expressed in 13% and 39%, respectively, of the SGCs surrounding masseter ganglion neurons. SSTR2 receptors were expressed by 94% of SGCs that contained SST. The expression of both SST14 and SSTR2 was significantly higher in SGCs than in ganglion neurons.

**Discussion/Conclusions**: This study shows that both masseter ganglion neurons and their associated SGCs express SST and SSTR2, but that the expression of SSTR2 is substantially higher in SGCs. One interpretation of the present results is that ganglion neurons may release SST which acts, via the SSTR2 receptor to modify SGC function. Future research will determine the effect of SSTR2 activation on SGCs function.

##### The fragility and reliability of conclusions of anesthesia and critical care randomized trials with statistically significant findings: a systematic review

François Grolleau^a,b^, Gary S. Collins^c^, Andrei Smarandache^d^, Romain Pirracchio^e,f,g^, Clément Gakuba^a,h^, Isabelle Boutron^i,j,k,l^, Jason W. Busse^m^, P. J. Devereaux^n^ and Yannick Le Manach^b^

^a^CHU Caen, Department of Anesthesiology and Critical Care Medicine, CHU Caen Côte de Nacre, Caen, France; ^b^Departments of Anesthesia & Clinical Epidemiology and Biostatistics, Michael DeGroote School of Medicine, Faculty of Health Sciences, McMaster University and the Perioperative Research Group, Population Health Research Institute, Hamilton, Canada; ^c^Centre for Statistics in Medicine, Nuffield Department of Orthopaedics, Rheumatology and Musculoskeletal Sciences, Botnar Research Centre, University of Oxford, Oxford, UK; ^d^Michael G. DeGroote School of Medicine, Faculty of Health Sciences, McMaster University, Hamilton, Canada; ^e^Department of Anesthesiology and Critical Care Medicine, Hôpital Européen Georges Pompidou, APHP, Université Paris Descartes, Sorbonne Paris Cite, Paris, France; ^f^Department of Anesthesiology and Perioperative Care, University of California San Francisco, San Francisco General Hospital and Trauma Center, San Francisco, CA; ^g^Department of Biostatistics and Medical Informatics, INSERM U-1153, team ECSTRA, Université Paris Diderot, Sorbonne Paris Cite, Paris, France; ^h^Normandie Univ, UNICAEN, INSERM, INSERM UMR-S U1237, “Physiopathology and Imaging of Neurological Disorders” PhIND, Caen, France; ^i^Université Paris Descartes, Sorbonne Paris Cité, Paris, France; ^j^Centre de Recherche Epidémiologie et Statistique, INSERM U1153, Paris, France; ^k^Cochrane France, Paris, France; ^l^Centre d’Épidémiologie Clinique, Hôpital Hôtel-Dieu, Assistance Publique-Hôpitaux de Paris, Paris, France; ^m^Departments of Anesthesia and Health Research Methods, Evidence & Impact, and the Michael G. DeGroote Institute for Pain Research and Care, McMaster University, Hamilton, Canada; ^n^Departments of Medicine & Health Evidence and Impact, Michael DeGroote School of Medicine, Faculty of Health Sciences, McMaster University and the Perioperative Research Group, Population Health Research Institute, Hamilton, Canada

**CONTACT** Yannick Le Manach yannick.lemanach@phri.ca Departments of Anesthesia & Clinical Epidemiology and Biostatistics, Michael DeGroote School of Medicine, Faculty of Health Sciences, McMaster University, Hamilton, Ontario, Canada

© 2018 François Grolleau, Gary S. Collins, Andrei Smarandache, Romain Pirracchio, Clément Gakuba, Isabelle Boutron, Jason W. Busse, P. J. Devereaux and Yannick Le Manach. Published with license by Taylor & Francis Group, LLC.

This is an Open Access article distributed under the terms of the Creative Commons Attribution License (http://creativecommons.org/licenses/by/4.0/), which permits unrestricted use, distribution, and reproduction in any medium, provided the original work is properly cited.

**KEYWORDS** Critical care; intensive care; anesthesia; randomized controlled trials; research methodology; research report

**Objectives**: The Fragility Index (FI), which represents the number of patients responsible for a statistically significant finding, has been suggested as an aid for interpreting the robustness of results from clinical trials. A small FI indicates that the statistical significance of a trial depends on only a few events. Our objectives were to calculate the FI of statistically significant results from randomized controlled trials (RCT) of anesthesia and critical care interventions and to determine the frequency of distorted presentation of results or ‘spin’.

**Data sources**: We systematically searched MEDLINE from 1 January 2007 to 22 February 2017 to identify RCTs exploring the effect of critical care medicine or anesthesia interventions.

**Study selection**: Studies were included if they randomized patients 1:1 into two parallel arms, and reported at least one statistically significant (P < 0.05) binary outcome (primary or secondary).

**Data extraction**: Two reviewers independently assessed eligibility and extracted data. The FI was determined for the chosen outcome. We assessed the level of spin in negative trials and the presence of recommendations for clinical practice in positive trials.

**Data synthesis**: We identified 166 eligible RCTs with a median sample size of 207 patients (interquartile range [IQR] 109–497). The median FI was 3 (IQR 1–7), which means that adding three events to one of the trials treatment arms eliminated its statistical significance. High spin was identified in 42% (n = 30) of negative RCTs while 21% (n = 20) of positive RCTs provided recommendations. Lower levels of spin and recommendations were associated with publication in journals with high impact factors (P < 0.001 for both).

**Conclusion**: Statistically significant results in anesthesia and critical care RCTs are often fragile, and study conclusions are frequently affected by spin. Routine calculation of the FI in medical literature may allow for better understanding of trials and therefore enhance the quality of reporting.

###### Conflicts of interest

The remaining authors have disclosed that they do not have any conflicts of interest.

###### Authors contribution

Drs Grolleau and Le Manach had full access to all of the data in the study and take responsibility for the integrity of the data and the accuracy of the data analysis.

- **Study concept and design**: Grolleau, Le Manach.
- **Acquisition of data**: Grolleau, Smarandache.
- **Analysis and interpretation of data**: Grolleau, Le Manach.
- **Drafting of the manuscript**: Grolleau.
- **Critical revision of the manuscript for important intellectual content**: Grolleau, Collins, Pirracchio, Gakuba, Boutron, Busse, Devereaux, Le Manach.

##### Learned expectations on the perception of pain using a classical conditioning paradigm

Zoey Walden^a^, Pierre-Alexandre Bourgouin^b^, Véronique Taylor^c^, Pierre Rainville^d^ and Mathieu Roy^e^

^a^Psychology, McGill University, Montréal, QC, Canada; ^b^Psychology, Université de Québec et Montréal (UQAM), Montréal, QC, Canada; ^c^Psychology, Bishop’s University, Lennoxville, QC, Canada; ^d^Stomatology & Psychology, Université de Montréal & Centre de Recherche de l’Institut Universitaire de Gériatrie de Montréal (CRIUGM), Montréal, QC, Canada; ^e^McGill & Centre de Recherche de l’Institut Universitaire de Gériatrie de Montréal (CRIUGM), Montréal, QC, Canada

**CONTACT** Zoey Walden zoey.walden@mail.mcgill.ca Psychology, McGill University, Montréal, QC, Canada

© 2018 Zoey Walden, Pierre-Alexandre Bourgouin, Véronique Taylor, Pierre Rainville and Mathieu Roy. Published with license by Taylor & Francis Group, LLC.

This is an Open Access article distributed under the terms of the Creative Commons Attribution License (http://creativecommons.org/licenses/by/4.0/), which permits unrestricted use, distribution, and reproduction in any medium, provided the original work is properly cited.

**Introduction/Aim**: Pain naturally activates learning processes in anticipation to painful stimuli; moreover, the perception of pain changes based on the predictive-value of a cue. This change is dynamic and can be modeled on a trial-by-trial basis. In a prior study we used symbols (conditioned stimuli) with one reversal; therefore, the purpose of the current study was twofold: to confirm prior findings that pain ratings decrease at the presentation of a new cue; to see if the effect is consistent across multiple introductions of a new cue-to-shock pairing.

**Methods**: We examined the association of pain to a conditioned stimulus on a trial-by-trial basis using subjective pain ratings. Conditioned visual stimuli (CS+) were faces that were presented and terminated simultaneously with painful transcutaneous stimulation applied to the sural nerve on 50% of trials (unconditioned stimuli or US). There were 5 reversals, whereby a face (CS+) that was previously associated the US became “safe” (CS extinct). At reversal, a new face (new CS+) was presented that now became associated with the US. A regression model was run using subjective pain ratings.

**Results**: We expected a new face to result in lower pain ratings since it was not predictive of pain. We found an overall effect across all reversals and that a new face presentation was associated with lower pain ratings (*t*(34) = 2.46, *p* = .002).

**Discussion/Conclusions**: Novel visual stimuli that do not predict a painful stimulus result in lower pain perceptions after presentation. This effect is robust across different studies and stimuli.

##### The chronic pain network’s (CPN) clinical research network (CRN)

Ian Gilron^a^, Bruce Dick^y^, Daniella San-Martin-Feeney^y^, Chris DeBow^b^, Norm Buckley^c^, Krista Baerg^d^, Jillian Banfield^e^, Etienne Bisson^f^, Adena Brown^g^, Fiona Campbell^h^, Manon Choiniere^i^, Scott Duggan^j^, Renee El-Gabalawy^k^, Allen Finley^l^, Lauren Harris^m^, Keith Jarvi^n^, Eve-Ling Khoo^o^, Kayla Kilborn^p^, Susan Lau^q^, Mary Lynch^r^, Aaron MacInnes^s^, Casey McMahon^t^, Allison McPeak^u^, Melanie Noel^v^, Lisa Patterson^w^, Patricia Poulin^x^, Saifee Rashiq^y^, Nivez Rasic^z^, Tiffany Rice^aa^, Cyril Schneider^bb^, Yoram Shir^cc^, Jennifer Stinson^dd^, Michelle Verrier^ee^, Marc White^ff^, Ramesh Zacharias^gg^, Jessie Dhillon^hh^ and Linda Ferguson^ii^

^a^Biomedical and Molecular Science, Queen’s University, Kingston, Ontario, Canada, gilroni@queensu.ca; ^b^Patient Partner, Chronic Pain Network, Halifax, Nova Scotia, Canada, jcdebow@gmail.com; ^c^Anesthesiology, McMaster University, Hamilton, Ontario, Canada, buckleyn@mcmaster.ca; ^d^Patient Partner, Chronic Pain Network, Vancouver, British Columbia, Canada, dr.kbaerg@usask.ca; ^e^Pain & Perioperative Medicine, Dalhousie University, Halifax, Nova Scotia, Canada, jillian.banfield@nshealth.ca; ^f^Anesthesiology andPerioperative Medicine, Queen’s University, Kingston, Ontario, Canada, etienne.bisson@kingstonhsc.ca; ^g^Anesthesia,Pain Management & Perioperative Medicine, Dalhousie University, Halifax, Nova Scotia, Canada, adena.cox@iwk.nshealth.ca; ^h^Anesthesia and Pain Medicine, The Hospital for Sick Children, Toronto, Ontario, Canada, fiona.campbell@sickkids.ca; ^i^Anesthesiology and Pain Medicine/Research Center of the Centre hospitalier de l’Université de Montréal, Université de Montréal, Montreal, Quebec, Canada, scott.duggan@kingstonhsc.ca; ^j^Anesthesiology and Perioperative Medicine, Queen’s University, Kingston, Ontario, Canada, duggans@kgh.kari.net; ^k^Anesthesia and Perioperative Medicine, University of Manitoba, Winnipeg, Manitoba, Canada, renee.el-gabalawy@umanitoba.ca; ^l^Department of Anesthesia, Dalhousie University, Halifax, Nova Scotia, Canada, allen.finley@dal.ca; ^m^Anesthesia and Pain Management, The Hospital for Sick Children, Toronto, Ontario, Canada, lauren.harris@sickkids.ca; ^n^nDivision of Urology,Department of Surgery, Mount Sinai Hospital, Toronto, Ontario, Canada, kjarvi@mtsinai.on.ca; ^o^Department of Anesthesiology and Pain Medicine, The Ottawa Hospital, Ottawa, Ontario, Canada, ekhoo@ohri.ca; ^p^Anesthesia and Perioperative Medicine, University of Manitoba, Winnipeg, Manitoba, Canada, kkilborn@exchange.hsc.mb.ca; ^q^Wasser Pain Management Centre, Mount Sinai Hospital, Toronto, Ontario, Canada, susan.lau@sinaihealthsystem.ca; ^r^Department of Anesthesia, Dalhousie University, Halifax, Nova Scotia, Canada, mary.lynch@dal.ca; ^s^Department of Anesthesiology, University of British Columbia, Vancouver, British Columbia, Canada, aaron.macinnes@fraserhealth.ca; ^t^Department of Pediatrics, University of Saskatchewan, Saskatoon, Saskatchewan, Canada, cj.mcmahon@usask.ca; ^u^Department of Anesthesia, Alberta Children’s Hospital, Calgary, Alberta, Canada, aemcpeak.uc@gmail.com; ^v^Department of Psychology, University of Calgary, Calgary, Alberta, Canada, melanie.noel@ucalgary.ca; ^w^Department of Anesthesiology, Hamilton Health Sciences, Hamilton, Ontario, Canada, pattersl@hhsc.ca; ^x^Anesthesiology and Pain Medicine, University of Ottawa, Ottawa, Ontario, Canada, ppoulin@toh.ca; ^y^Department of Anesthesiology & Pain Medicine, University of Alberta, Edmonton, Alberta, Canada, srashiq@uablerta.ca; ^z^Department of Anesthesiology, Alberta Children’s Hospital, Calgary, Alberta, Canada, nivez.rasic@ahs.ca; ^aa^Alberta Children’s Health Research Institute, Calgary, Alberta, Canada, tiffanykrice@gmail.com; ^bb^Department of Rehabilitation, Université Laval, Quebec city, Quebec, Canada, cyril.schneider@rea.ulaval.ca; ^cc^Anesthesia, McGill University, Montreal, Quebec, Canada, yoram.shir@muhc.mcgill.ca; ^dd^Anesthesia and Pain Management, The Hospital for Sick Children, Toronto, Ontario, Canada, jennifer.stinson@sickkids.ca; ^ee^Department of Anesthesiology and Pain Medicine, University of Alberta, Edmonton, Alberta, Canada, mrheault@ualberta.ca; ^ff^Work Wellness and Disability Prevention Institute, Vancouver, British Columbia, Canada, president@cirpd.org; ^gg^Department of Anesthesia, McMaster University, Hamilton, Ontario, Canada, zacharias@hhsc.ca; ^hh^Department of Anesthesiology, University of British Columbia, Vancouver, British Columbia, Canada, jessie.dhillon@ubc.ca; ^ii^Alan Edwards Pain Management Unit, McGill University, Montreal, Quebec, Canada, linda.ferguson@muhc.mcgill.ca

**CONTACT** Ian Gilron gilroni@queensu.ca

© 2018 Ian Gilron, Chris DeBow, Norm Buckley, Krista Baerg, Jillian Banfield, Etienne Bisson, Adena Brown, Fiona Campbell, Manon Choiniere, Scott Duggan, Renee El-Gabalawy, Allen Finley, Lauren Harris, Keith Jarvi, Eve-Ling Khoo, Kayla Kilborn, Susan Lau, Mary Lynch, Aaron MacInnes, Casey McMahon, Allison McPeak, Melanie Noel, Lisa Patterson, Patricia Poulin, Saifee Rashiq, Nivez Rasic, Tiffany Rice, Cyril Schneider, Yoram Shir, Jennifer Stinson, Michelle Verrier, Marc White, Ramesh Zacharias, Jessie Dhillon and Linda Ferguson. Published with license by Taylor & Francis Group, LLC.

This is an Open Access article distributed under the terms of the Creative Commons Attribution License (http://creativecommons.org/licenses/by/4.0/), which permits unrestricted use, distribution, and reproduction in any medium, provided the original work is properly cited.

**Introduction/Aim**: The aim of the CRN is to contribute to the research activities of the CPN. There is a growing number of multidisciplinary clinics across Canada. Patients referred to these facilities: A) may be ideal research participants, and B) will ultimately benefit from advances of such research. A shared infrastructure could facilitate cost-effective multicenter research. This network will provide synergies that combine: 1) skilled academic clinicians who identify new testable hypotheses, 2) experienced researchers who provide research methodology, 3) efficient high-quality clinics that enhance study participant recruitment.

**Methods**: We have established a sustainable model for the CRN that is supported by equal funding contributions from the CPN and each participating site. The Vision is for a network of real-world clinics dedicated to the research of the Canadian CPN. The path to excellence is to ensure that every network member: 1) has something valuable to offer, 2) has a vested interest in overall network success, 3) interacts effectively with the rest of the network.

**Results**: Site investigators and site research coordinators have been identified for 12 sites across Canada and three multicenter studies involving the CRN are already underway with a number of other investigations being planned.

**Discussion/Conclusions**: To date, this CRN is the largest national clinical pain research network in Canada and we are in the process of establishing a sustainability model that will allow this network to expand and continue for years to come.

##### Translational profiling in a mouse model of neuropathic pain

Sonali Uttam^a^, Calvin Wong^b^, Inês S. Amorim^c^, Seyed Mehdi Jafarnejad^d^, Shannon N. Tansley^e^, Jieyi Yang^e^, Masha Prager-Khoutorsky^e^, Jeffrey S. Mogil^f^, Christos G. Gkogkas^g^ and Arkady Khoutorsky^h^

^a^Department of Anesthesia and Division of Experimental Medicine, McGill University, Montreal, Quebec, Canada; ^b^Department of Anesthesia, McGill University, Montreal, Quebec, Canada; ^c^The University of Edinburgh, Patrick Wild Centre and Centre for Discovery Brain Sciences, Edinburgh, Scotland, United Kingdom; ^d^Department of Biochemistry and Goodman Cancer Research Centre, McGill University, Montreal, Quebec, Canada; ^e^Department of Physiology, McGill University, Montreal, Quebec, Canada; ^f^Department of Psychology, Department of Anesthesia and Alan Edwards Centre for Research on Pain, McGill University, Montreal, Quebec, Canada; ^g^The University of Edinburgh, Patrick Wild Centre and Centre for Discovery Brain Sciences, Edinburgh, Scotland, United Kingdom and Alan Edwards Centre for Research on Pain, Montreal, Quebec, Canada; ^h^Department of Anesthesia, Department, Division of Experimental Medicine and Alan Edwards Centre for Research on Pain, McGill University, Montreal, Quebec, Canada

**CONTACT** Sonali Uttam sonali.uttam@mail.mcgill.

© 2018 Sonali Uttam, Calvin Wong, Inês S. Amorim, Seyed Mehdi Jafarnejad, Shannon N. Tansley, Jieyi Yang, Masha Prager-Khoutorsky, Jeffrey S. Mogil, Christos G. Gkogkas and Arkady Khoutorsky. Published with license by Taylor & Francis Group, LLC.

This is an Open Access article distributed under the terms of the Creative Commons Attribution License (http://creativecommons.org/licenses/by/4.0/), which permits unrestricted use, distribution, and reproduction in any medium, provided the original work is properly cited.

**Introduction/Aim**: Chronic pain is a debilitating and poorly treated condition whose underlying mechanisms are not well understood. Previous studies in animal models of pain have examined changes in the transcriptome associated with chronic pain. This approach does not take into account the regulation of gene expression at the mRNA translation step, which plays a central role in determining protein levels in the cell. This study aims to identify the mRNA for which translation is altered in response to nerve injury using genome-wide ribosome profiling technique.

**Methods**: To induce neuropathic pain, we used spared nerve injury model. Dorsal root ganglion and spinal cord tissue associated with the sciatic nerve were extracted 30 days post injury and processed for ribosome profiling (Ingolia et al., 2009). Using bioinformatic analysis we calculated the mRNA abundance (proxy for transcription) and translation efficiency (proxy for translation) for all transcripts. Meta-gene analysis was carried out by Ingenuity Pathway Analysis (IPA) to identify the commonly affected pathways.

**Results**: We found subsets of genes that are differentially regulated at transcriptional and translational levels. We also identified the key functions and pathways linked to translational control and neuropathic pain.

**Discussion/Conclusions**: Our analysis of transcriptome and translatome following nerve injury showed that changes in translation efficiency and mRNA levels occur in the opposite direction for multiple mRNAs. This points toward the phenomena of translational buffering and highlights the importance of translation regulation. The IPA analysis revealed the altered cellular pathways, identifying ERK as a key regulator of both transcriptional and translational networks.
